# Synergy of triazolyl substituents at C1 and C3 of galactose for high-affinity and selective galectin-4C inhibition†

**DOI:** 10.1039/d5cb00106d

**Published:** 2025-07-18

**Authors:** Alexander Dahlqvist, Rob Marc Go, Chandan Kishor, Hakon Leffler, Helen Blanchard, Ulf J. Nilsson

**Affiliations:** a Department of Chemistry, Lund University Lund Sweden ulf.nilsson@chem.lu.se; b Institute for Biomedicine and Glycomics, Griffith University Queensland Australia helen.blanchard@universityofgalway.ie; c Division of Microbiology, Immunology and Glycobiology, Lund University Lund Sweden; d School of Biological and Chemical Sciences, University of Galway Galway H91 TK33 Ireland

## Abstract

Galectins are a family of carbohydrate-recognising proteins involved in regulation of cell adhesion and cell signaling, leading to roles in *e.g.* cancer progression, fibrosis, and ulcerative colitis. Glycomimetic galectin inhibitors based on different molecular scaffolds are known and have demonstrated effects from cell experiments to the clinic. Presented here is the synthesis and evaluation of 3-aryltriazolyl-C1-galactosyls leading to discovery of an unexpected synergy effect between C1 and C3 triazolyl substituents to give galectin-4C (C-terminal domain) inhibitors with affinities down to 9.5 μM and up to thirty-sevenfold selectivity for galectin-4C over other galectins. X-ray structural analysis of one inhibitor:galectin-4C complex revealed that both the C1 and C3 arene-substituents engage in interactions with the galectin-4C binding site. These molecules have potential as lead compounds towards discovery of galectin-4-targeting compounds addressing inflammatory conditions, such as inflammatory bowel disease and ulcerative colitis.

## Abstract 2

Galectin-4 differs from other galectins in carbohydrate-binding specificity, cellular and pathophysiological function, and roles in *e.g*. cancer progression, fibrosis, and ulcerative colitis, making potent, selective inhibitors desired.

## Graphical abstract

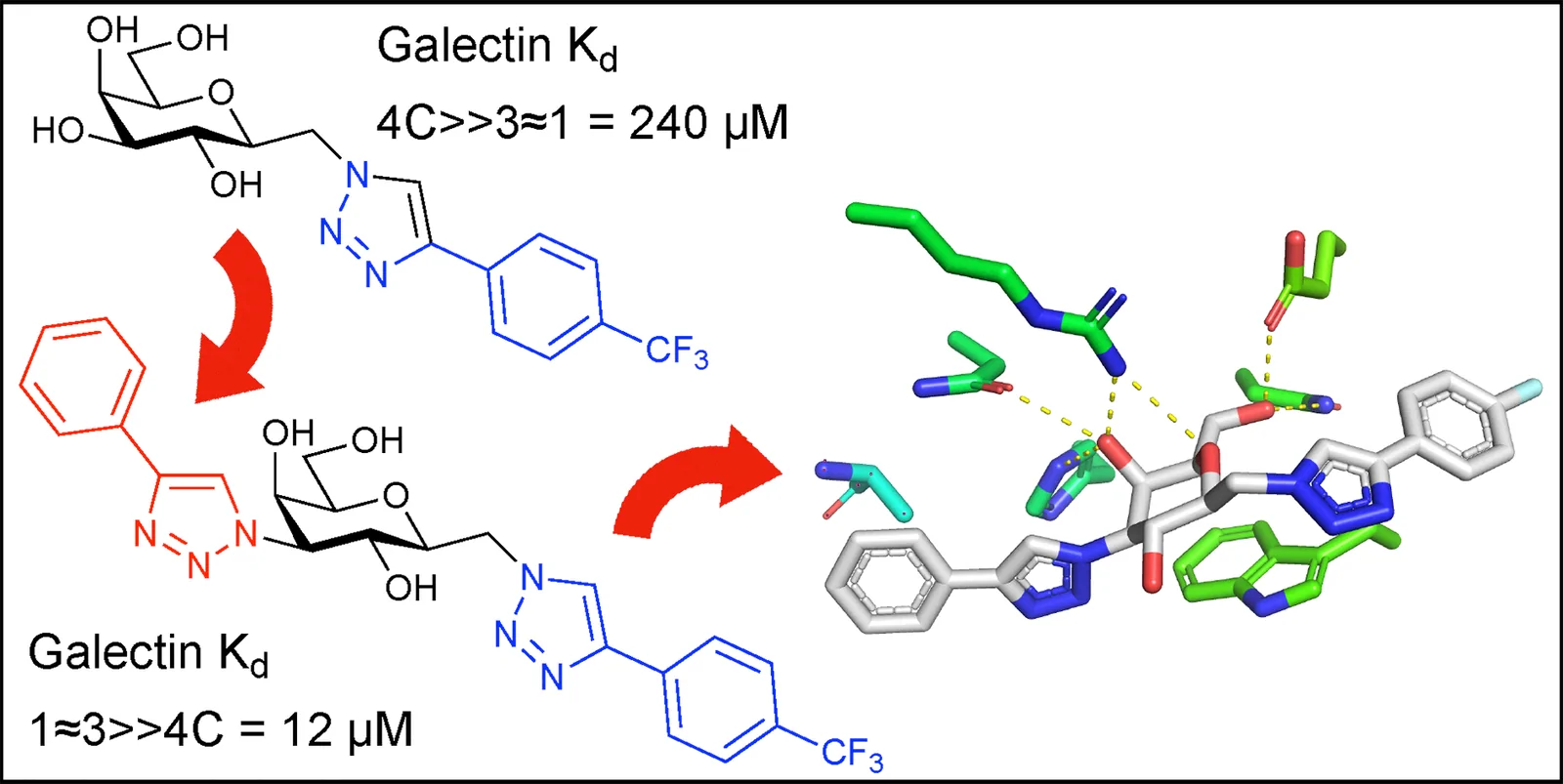

## Introduction

Carbohydrate recognition is fundamental in cell biology, from *e.g.* chaperone-assisted protein folding,^1^ the trafficking of proteins in cells,^2^ to cell surface organization^3^ and this involves carbohydrate recognition by a variety of proteins. Galectins are such a family of proteins,^4–6^ composed of three sub-categories named prototype, tandem repeat, and chimera. The prototype category includes galectin-1 and usually occur as homodimers of two identical ∼130 amino acid carbohydrate recognition domains (CRDs). Bearing two non-similar CRDs called “N” for the N-terminal CRD and “C” for the C-terminal, the tandem repeat galectins include galectins-4 and others, while the sole member of the chimera category, galectin-3, has one CRD preceded by a long intrinsically disordered collagen-like N-terminal.^7^ Galectins have the ability to bind *N*- and *O*-linked glycans for example to cluster glycoprotein receptors like vascular endothelial growth factor receptor (VEGFr)^8^ and cell adhesion proteins like integrins^9^ giving them a role in regulating the interactions of a cell with its environment. Galectin overexpression has been documented in several diseases that involve dysfunctional cell signalling such as in chronic inflammation^10–16^ and cancer.^11,14,17,18^

Inhibition of galectins has previously been shown to be an effective therapy in models of some of these diseases.^19–30^ Different classes of galectin-inhibitors have been discovered including multivalent molecules and glycan-mimicking, glycomimetic, small-molecule inhibitors.^31^ Most small-molecule inhibitors rely on decorating a galactoside scaffold with non-carbohydrate structural moieties scaffolds,^19,27,29,30,32–43^ but several groups also report on non-carbohydrate inhibitors.^44–50^ Several of these studies have reported high-affinity and selectivity, as well as sometimes optimized ADME and pharmacokinetics, of drug-like small molecule inhibitors of galectin-1, -3, and -8N inhibitors, while potent and selective small-molecule inhibitors against galectin-4, 7, and 9 are still scarce despite their medical relevance.

Herein, we report on the synthesis and biochemical and structural analysis of galactosyl derivatives equipped with heterocyclic structures at both C1 and C3 that show selectivity and μM affinity for galectin-4C, a galectin reported to be of key relevance in inflammatory bowel disease and colitis,^51^ as well as in cancer.^52,53^

## Results and discussion

### Synthesis of C1-aryltriazole galactopyranosyls

**Chart 1:**
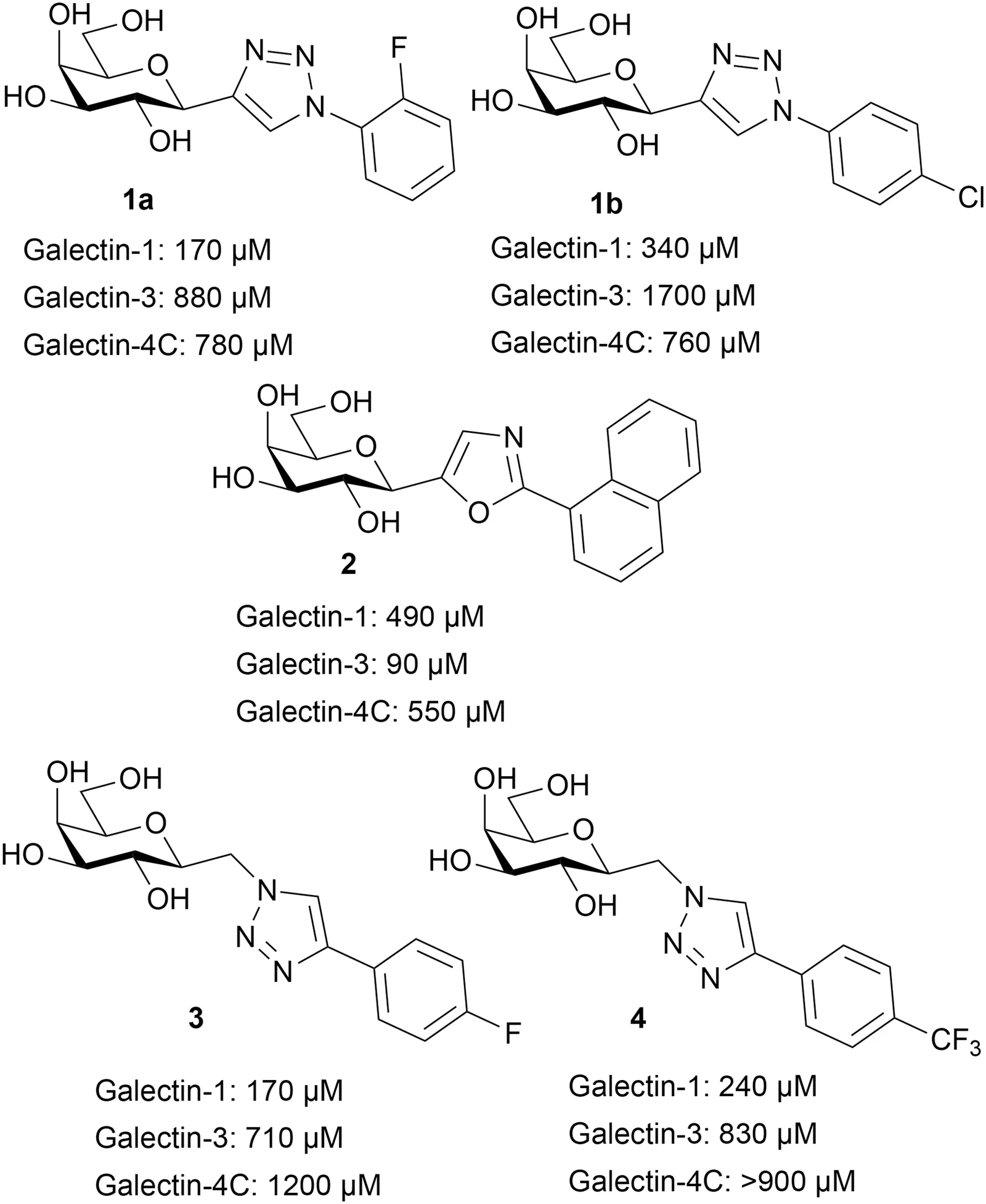
C1-Galactosyl galectin inhibitors with affinity and selectivity pattern.^54,55^

The known C1-aryltriazolyl galactopyranosyls^54^1a–b (Chart 1) possess good galectin-1 affinities and good to high selectivity, while aryloxazolyl galactopyranoside 2 is selective for galectin-3 with good affinity (90 mM). The (aryltriazolyl)methyl galactopyranosides^55^3 and 4 have good galectin-1 affinity while having slightly worse selectivities for galectin-1 over galectin-3 as compared to 1a–b. Affinities for other galectins, such as for example galectin-4N and 4C, were in general significantly weaker.

**Scheme 1:**
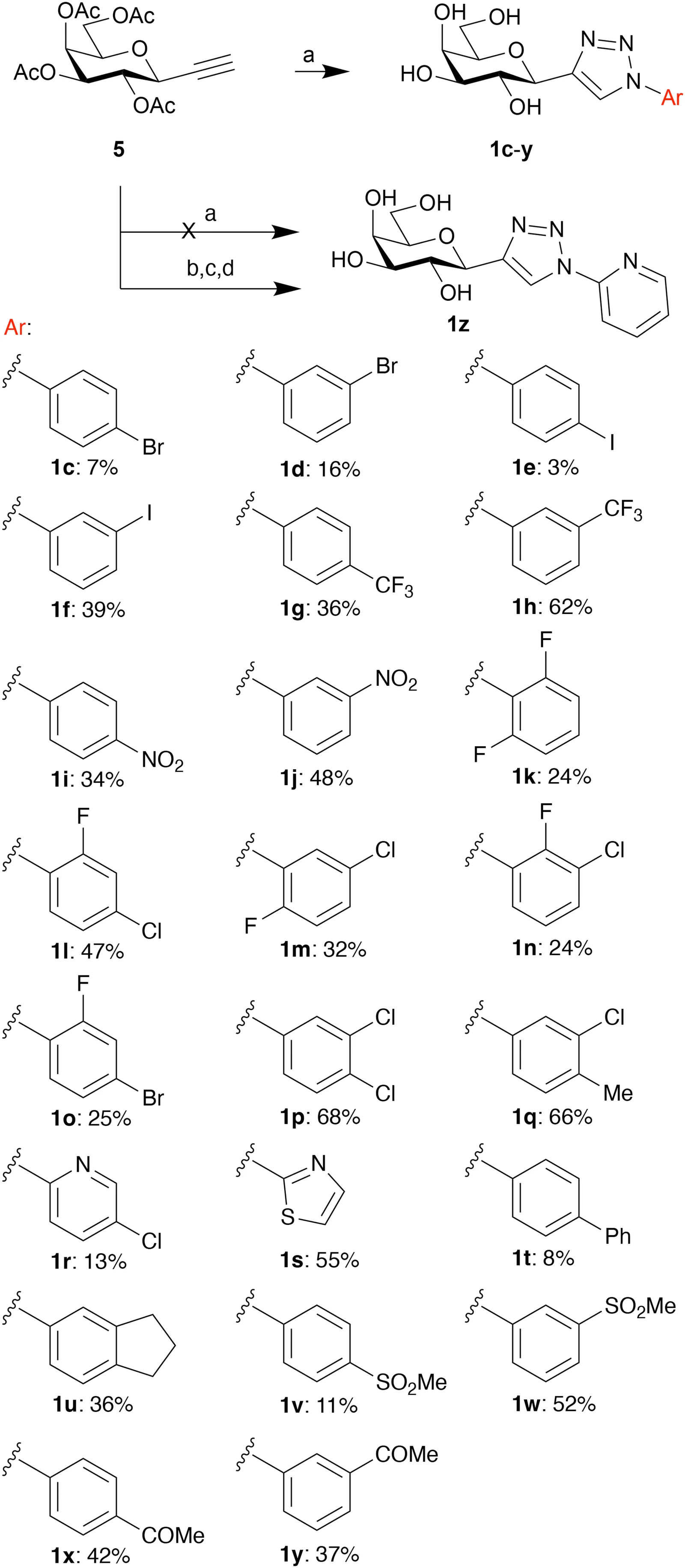
Synthesis of the aryltriazoles 1c–1y: (a) copper(i)iodide triethylamine, appropriate aryl azide, ACN (dry), r.t., 2–12 h, then sodium methoxide in dry methanol, r.t 2 h. Yields 3–62%. Synthesis of pyridinyltriazoles (1z) was unsuccessful with regular conditions and required an alternative synthesis route; (b) copper(i)iodide, triethylamine, 2-azido-5-chloropyridine, ACN (dry), r.t., 12 h. (c) Palladium hydroxide on carbon (20 wt%) cyclohexene/ethanol 2 : 1 80 °C, 12 h. (d) Sodium methoxide in dry methanol, 2 h.

To refine the SAR of the galactosyl triazoles 1a–1b, we synthesized an extensive collection of C1-aryltriazole galactopyranosyls (1c–1y) from 2,3,4,6-tetraacetyl-1-deoxy-1-ethynyl-β-d-galactopyranoside (5) *via* copper catalyzed azide alkyne cycloadditions (CuAAC)^56^ followed by sodium methoxide-mediated transesterfication in yields varying from low to intermediate (Scheme 1). Aryl azides used were selected based on commercial availability. Apart from the 2-thiazolyltriazole (1s) and the 5-chloro-pyridin-2-yltriazole (1r), no other heterocycles could be synthesized successfully and therefore an alternative route to the 2-pyridinyltriazole (1z) through dechlorination of 5-chloropyridinyl galactosyl triazole was developed. The unoptimized and varying yields of, and in one case (1z) unsuccessful, CuAAC were somewhat unexpected as CuAAC is generally considered to be an indeed reliable and efficient reaction. An explanation may be that the CuAAC is most efficient with electron-poor alkynes, while the alkyne 5 is comparatively electron-rich and possibly somewhat sterically hindered. All final compounds were purified using preparative HPLC to a minimum of 95% purity, as determined by analytical HPLC.

### Galectin affinities of C1-aryltriazole galactopyranosyls

Affinities of 1c–1z for galectin-1, -3, -4C, -4N, -7, -8C, -8N, -9C, and -9N were measured using an established fluorescence polarization assay^57^ with reported probes and conditions.^19^

Only galectins-1 and -3 showed appreciable binding for these anomeric mono-derivatized C-galactosyls and affinities for the other galectins are in the range of 1.5 mM or weaker and are not reported (Table 1). Galectin-4C is included in Table 1 to represent a weak-binding galectin towards 1a–1z C1 mono-aryl derivatives.

**Table 1:** Dissociation constants (μM) of aryltriazolyl galactopyranosyls 1c–1z and the references methyl β-d-galactopyranoside and methyl β-lactoside for galectin-1, 3, and -4C

	Galectin-1	Galectin-3	Galectin-4C
1a^54^ ADC-57	170	880	n.d.^d^
1b^54^ ADC-17	340	1700	n.d.
1c	140 ± 25^a^	1400 ± 340	1400/1500^d^
1d	1400 ± 95	1600 ± 30	>2000^b^
1e	460 ± 50	>1000^b^	n.d.
1f	1300 ± 220	>1000^b^	n.d.
1g	1300 ± 320	1200 ± 31	2300/2600^c^
1h	1500 ± 62	1300 ± 69	1500/2200^c^
1i	400 ± 5	1500 ± 67	910 ± 46
1j	1800 ± 64	1000 ± 23	>2000^b^
1k	1300 ± 190	1200 ± 90	>2000^b^
1l	300 ± 20	1200 ± 110	>2000^b^
1m	580 ± 12	870 ± 140	1300 ± 260
1n	1100 ± 48	>1000^b^	>2000^b^
1o	310 ± 29	770 ± 89	>2000^b^
1p	1300 ± 200	660 ± 53	2300/2500^c^
1q	1200 ± 240	1400 ± 38	>2000
1r	650 ± 3	490 ± 6	>2000
1s	980 ± 7	910 ± 92	>2000
1t	970 ± 25	2000 ± 270	880 ± 82
1u	2100 ± 73	1400 ± 160	2100 ± 490
1v	780 ± 43	690 ± 70	2900/3500
1w	1400 ± 85	1400 ± 70	>2000^b^
1x	970 ± 65	1200 ± 83	1900 ± 550
1y	1300 ± 260	1000 ± 200	2000/2700
1z	150 ± 17	880 ± 100	2600/3100
β-Me Gal	>10 000^58^	4400^58^	>10 000^59^
β-Me Lac	190^58^	220^58^	2900^59^

a *K*_d_ and SEM are calculated from 3–6 single point measurements at different inhibitor concentrations.

b Estimated vaue; no apparent binding at the highest inhibitor concentration.

c Only two data points revealed inhibition and the two values are shown.

d Not determined.

The known 2-fluorophenyl-(1a) and 4-chlorophenyltriazole (1b) galactopyranosyls^54^ displayed together with the 4-bromophenyl derivative 1c the highest affinities and selectivities for galectin-1. The 4-bromophenyl (1c) has somewhat better affinity (140 μM) and selectivity (tenfold) than the 4-chlorophenyl (1b), while the 4-iodophenyl (1e) has worse affinity and selectivity than both 1b and 1c. The 4-trifluoromethylphenyl compound (1g) has a much worse affinity. Two halo substituents in the 2,6-difluorophenyl 1k and the 3,4-dichlorophenyl 1p are not improving affinity but instead reverses selectivity to give a slight galectin-3 preference. This may be related to a change of conformational preference of the triazole-phenyl, which was demonstrated to be important in our previous work.^54^ Biphenyl (1t) and indanyl (1u) both have poor affinities and selectivities, showing that such extensions of the aryl system is not viable. Combinations between the substituent patterns reveal no clear cooperativity; 2-fluoro-4-chlorophenyl (1l) and 2-fluoro-4-bromophenyl (1o) are both worse in affinity and selectivity than the singly substituted phenyls. The 2-fluoro-3-chlorophenyl (1n) analog has poor affinity for both galectin-1 and -3, 2-fluoro-5-chlorophenyl (1m) has worse affinity and significantly worse selectivity than the parent monosubstituted phenyls. Other substituents, the methyl sulfonylphenyls (1v, 1w), the acetylphenyls (1x, 1y) and the nitrophenyls (1i, 1j), all have millimolar affinities and poor selectivities.

Heterocycle substituents give interesting results as the thiazolyl (1s), the only five-membered ring, has close to millimolar affinity and no selectivity, while the 2-pyridyl (1z) has good affinity (150 μM) and selectivity for galectin-1 over galectin-3. Additionally, the lack of cooperativity between substituents seen for the phenyl halogens also holds true for the heterocycles as the 5-chloro-2-pyridyl (1r) not only has poorer affinity than the parent monosubstituted aryls but reverses the selectivity with a slight preference for galectin-3 over galectin-1. Taken together, the anomeric mono-derivatized C_galactosyls 1a–1z display selectivity for galectin-1 and -3, albeit with only mediocre affinities.

### Synthesis of C3-derivatized C1-galactosyls

**Scheme 2:**
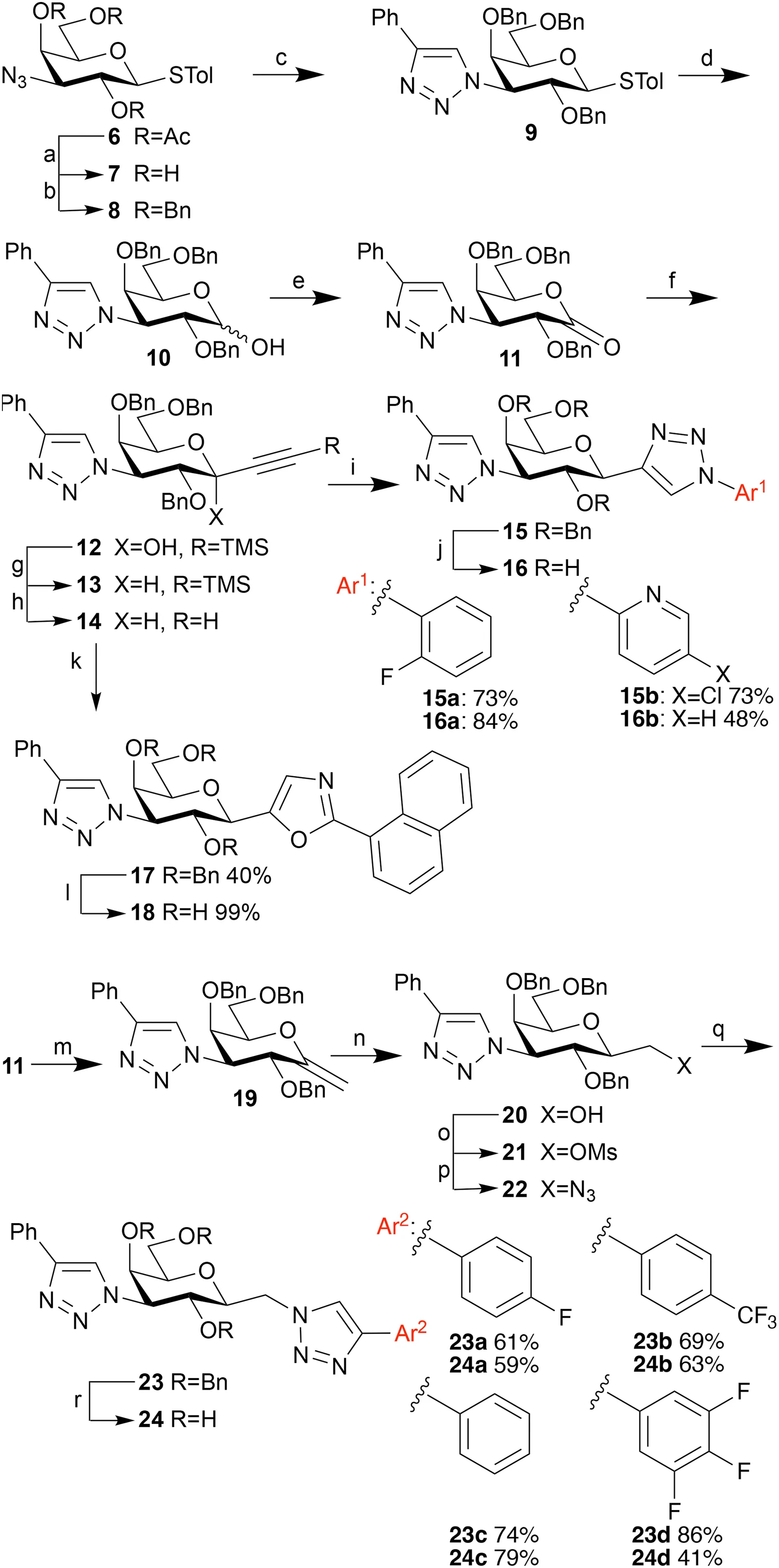
Synthesis of 1,3-dideoxy-1-(aryltriazolyl)-3-(phenyltriazolyl)-β-d-galactopyranosides 15a–b, 1,3-dideoxy-1-(1-naphthyloxazolyl)-3-(phenyltriazolyl)-β-d-galactopyranoside 17 and 1-deoxy-3-(phenyltriazolyl)-1-(aryltriazolyl)methyl-β-d-galactopyranosides 24a–d. (a) Sodium methoxide, methanol (dry), r.t, 2 h, 95%. (b) Sodium hydride, benzyl bromide, THF (dry), reflux 18 h, 84%. (c) Phenylacetylene, copper(i) iodide, triethylamine, ACN (dry), r.t, 18 h, 84%. (d) *N*-Bromosuccinimide, acetone/water, r.t, 10 min, 92%. (e) Dess–Martin periodinane, DCM (dry), r.t, 18 h, 99%. (f) Cerium(iii) dichloride trimethylsilyl acetylide, THF (dry), −78 °C to r.t, 8 h, 78%. (g) Triethylsilane, boron trifluoride etherate, ACN/DCM (dry) 0 °C, 8 h, 60%. (h) Tetrabutylammonium fluoride, THF, r.t, 10 min, 91%. (i) Aryl azide, Copper(i) iodide, triethylamine, ACN (dry), r.t, 18 h. (j) Palladium hydroxide on carbon, cyclohexene/ethanol, reflux. (k) 1-Cyanonaphthalene, [Bis(trifluoromethanesulfonyl)imidate] (triphenylphosphine)gold(i), 8-methylquinoline *N*-oxide, 55 °C, sealed tube under nitrogen. (l) Palladium hydroxide on carbon, cyclohexene/ethanol, reflux. (m) Petasis reagent, toluene, 80 °C, 48 h, 63%. (n) Borane dimethyl sulfide, THF (dry), 0 °C, 12 h, then hydrogen peroxide, sodium hydroxide, THF/water, 0 °C, 2 h, 62%. Diastereoselectivity 4 : 1 β : α. (o) Mesyl chloride, pyridine, 0 °C, 2 h, 95%. (p) Sodium azide, DMF (dry), 95 °C, 2 h, 92%. (q) Aryl acetylene, copper(i) iodide, triethylamine, ACN (dry), rt, 18 h. (r) Palladium hydroxide on carbon, cyclohexene/ethanol, 80 °C, 18 h.

Earlier studies have demonstrated that affinity and selectivity can be improved by introducing galactose C3-substituents on the galactopyranose for both galectin-1^27,28,30^ and galectin-3.^19,28,29,42,60,61^ Hence, we combined selected anomeric moieties of 1a, 1r, 1z, 2, 3, and 4 with one selected galactose C3 bi-aryl, 3-(4-pheny)-triazolyl, to enhance galectin affinities. In addition, three more anomeric groups were included: a 2-fluorophenyl-triazole, a phenyl-triazolylmethyl, and a (3,4,5-trifluorophenyl)-triazolylmethyl (Scheme 2).

De-*O*-acetylation of compound 6 with sodium methoxide in methanol gave the deprotected compound 7^28,62^ in a 95% yield (Scheme 1). Refluxing 7 in THF with sodium hydride and benzyl bromide to give the benzylated azidosugar 8 in yields up to 84%. A copper catalyzed azide–alkyne cycloaddition of 8 with phenylacetylene gave thiophenyl phenyltriazolyl derivative 9 (84% yield), which was hydrolyzed to compound 10 with *N*-bromosuccinimide in acetone/water with a yield of 92%. Compound 10 was oxidized to lactone 11 using Dess–Martin periodinane (99%), followed by an addition reaction using trimethylsilyl ethynyl cerium(iii)^54^ dichloride to give the alkyne sugar 12 (78% yield). Reduction of 12 using triethyl silane and boron trifluoride etherate gave C1-alkynyl derivative 13 in 60% yield, which was subsequently desilylated with tetrabutylammonium fluoride to give 14 (91%).

From here the synthesis branches; the C1-aryltriazolyl derivatives 15a–b were obtained using a second copper catalyzed azide–alkyne cycloaddition in yields of 73% for both 15a and 15b, followed by debenzylation with palladium hydroxide on carbon to give final compounds 16a–b. C1-Naphthyloxazolyl 17 was synthesized from 14 using a recently reported gold catalysis method^54,63^ utilizing (triphenylphosphine)gold(i) triflimidate, 8-methylquinoline *N*-oxide and 1-cyanonaphthalene, with a yield of 40%. Deprotection of 17 to 18 using palladium hydroxide on carbon proceeded smoothly with a yield of 99%.

Synthesis of the C1-(aryltriazolyl)methyl derivatives 24a–b branches from lactone 11. Heating of 11 in dry toluene with Petasis reagent^64,65^ gave the exomethylene ether 19 in a yield of 63%. Diastereoselective hydroboration–oxidation of 19 using borane dimethyl sulfide followed by hydrogen peroxide and sodium hydroxide^55^ gave 2-deoxy-3-phenyltriazolyl galactoheptulose 20 in a yield of 62% and with a diastereoselectivty of 4 : 1 favoring the desired β diastereomer (based on HPLC). Subsequently, mesylation with mesyl chloride in pyridine gave mesylate 21 (95% yield), which was converted to the azide 22 with sodium azide in DMF (92% yield). Finally, the protected final compounds were obtained using yet another copper catalyzed azide–alkyne cycloaddition with arylacetylene, copper(i) iodide and triethylamine to give 23a–d in yields of 61–86%, followed by deprotection with palladium hydroxide on carbon in cyclohexane/ethanol to give compounds 24a–d in yields of 41–63%. The synthesis of other exomethylene triazoles, like the 1-naphthyltriazole and the 1-trimethylsilyl triazole failed during the deprotection, a de-arylation resulted in only debenzylated 20 being recovered for these reactions. Final compounds were purified with preparative HPLC to a purity of 95% or higher as determined using HPLC/MS with UV/vis detector prior to galectin-binding evaluations.

### Galectin affinities of C3-derivatized C1-galactosyls

The doubly derivatized compounds 16a–b, 18 and 24a–b were tested for affinity towards galectins-1, -3, 4C, 4N, 7, 8C, 8N, 9C, and 9N in the fluorescence polarization assay.^57^ Affinity constants (*K*_d_) for galectins-1, -3 -4C, and -4N are listed in Table 2, while data for other galectins has been omitted for clarity as all compounds showed only a high micromolar or millimolar affinity for these galectins.

**Table 2:** Dissociation constants (μM) of 16a–b, 18 and 24a–d towards galectins-1, -3, -4C

	Galectin-1	Galectin-3	Galectin-4C	Galectin-4N
16a	280 ± 39^a^	34 ± 1	18 ± 2.0	90 ± 17
16b	180 ± 1	34 ± 3	9.6 ± 2.3	240 ± 54
18	530 ± 73	8.6 ± 0.4	2.3 ± 0.4	44 ± 13
24a	220 ± 10	200 ± 32	9.5 ± 1.3	130 ± 20
24b	440 ± 68	720 ± 78	12 ± 1.7	760 ± 32
24c	140 ± 5	90 ± 13	19 ± 2.9	290 ± 32
24d	240 ± 5	91 ± 2	22 ± 2.8	130 ± 23

a *K*_d_ and SEM are calculated from 3–6 single point measurements at different inhibitor concentrations.

Designed to have increased affinity and selectivity towards galectin-1, compounds 16a–b were a disappointment since galectin-1 affinity decreased for both compounds, while affinity increased for galectin-3 instead, reversing the selectivity compared to aryltriazoles 1a–b. To our surprise, however, affinity towards the C-terminal CRD of galectin-4 (galectin-4C) greatly increased up to as much as 79-fold better for 16b compared to 1a–b. This resulted in positively unexpected selectivity for galectin-4C over galectin-3, providing a promising starting point for the development of even more selective galectin-4C inhibitors.

Furthermore, the 1-naphthyloxazolyl derivative 18 is an even better galectin-4C inhibitor with a 4-fold selectivity over galectin-3 and an affinity of 2.3 μM despite the parent compound 2 relatively poor galectin-4C affinity of 550 μM. This SAR is further supported with the data of (aryltriazolyl)methyl galactopyranosyls 24a–d. Compared to the galectin-1 selective parent compounds lacking the 3-phenyltriazolyl substituent (3 and 4), affinities towards galectin-1 are slightly worse and selectivity is gone while galectin-4C affinities are enhanced to give dissociation constants of 9.5 μM and 12 μM for 24a and 24b, respectively. Importantly, the affinity for galectin-3 is still relatively poor, leading to a pronounced galectin-4C selectivity of 24a and 24b of at least twenty-onefold and up to thirty-sevenfold. The unsubstituted phenyl 24c and 3,4,5-trifluorophenyl derivative 24d show worse affinities towards galectin-4C and higher affinity towards galectins -1 and -3, resulting in worse selectivity and thus demonstrating selectivity-inducing effect the 4-fluoro and 4-trifluoromethyl groups of 24a and 24b.

### Atomic structure of the galectin-4C:24a complex

To understand the binding and selectivity of galectin-4C CRD for compounds 24a and 24b, X-ray crystallography was pursued to provide detailed atomic structural information. The X-ray crystallographic structure of human galectin-4C (amino acids 185–323) with 24a was determined at 2.28 Å resolution (Table S1, ESI†). The structure exhibited a space group of *P*2_1_ and a crystallographic asymmetric unit that contains four monomers (A–D) of galectin-4C CRD. Monomers showed some variation in electron density definition particularly monomer B where loops S1-F5 (amino acids 199–206), and S5-S6 (amino acids 252–255) had weak electron density, and the loop S3-S4 (amino acids 228–232) had no discernible electron density and hence the S3-S4 loop amino acids were omitted in the structure refinement. The S3-S4 loop in monomer D also exhibited weak electron density, only indicating the positions of the backbone atoms in this loop. Monomers A and B have well-defined electron density for the bound 24a whereas crystal packing interactions affected the binding of the ligand to monomers C and D, with no clear ligand electron density observed in these binding sites. This is due to crystallographic packing effects whereby a loop from a symmetry related molecule inserts into the carbohydrate binding site of monomers C and D, thereby preventing occupation by 24a.

### Galectin-4C interactions with 24a

**Fig. 1:**
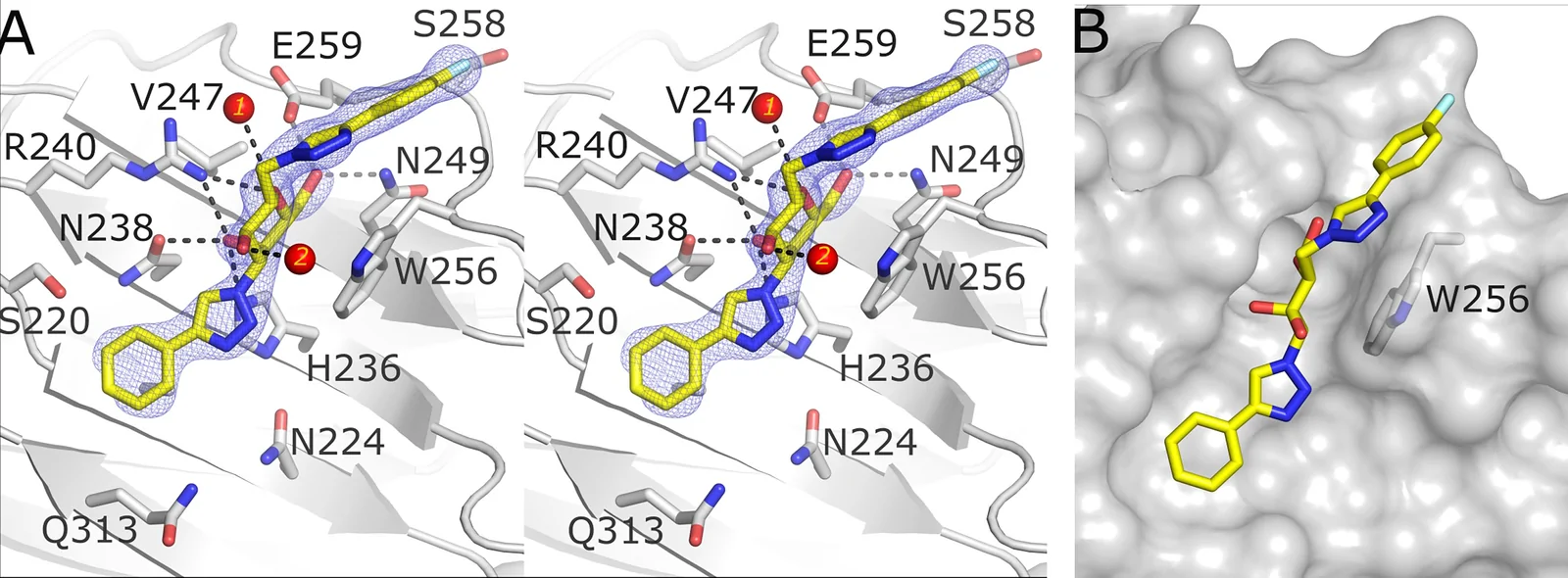
(A) Stereo figure. 2|*F*_o_| − |*F*_c_| *α*_c_ map at 1*σ* (blue mesh) depicting 24a (stick model; atoms: carbon (yellow), nitrogen (blue); oxygen (red); fluorine (cyan)) bound to galectin-4C CRD (monomer A) binding site (grey ribbon and stick), with hydrogen bond interactions shown as black dashed lines, and water molecules depicted as red spheres. (B) Surface representation of 24a in the binding site groove of galectin-4C.

In the monomer A and B CRDs the electron density was unambiguous for the galactose scaffold and comparable to previously reported galectin-galactose-based ligand crystal structures.^42,43,66^ Additional density extending from the C1 and C3 positions confirmed that ligand exchange with lactose had occurred successfully. The triazole rings from the C1 and C3 positions on the galactose scaffold orient themselves into positions similar to galectin-ligand complexes, as previously reported.^42,66^ As seen in Fig. 1A, the galactose moiety forms typical interactions with the galectin binding site.^42,43,66^ The C6 hydroxyl forms hydrogen bond interactions with Asn249 and Glu259, and Arg240 forms hydrogen bond interactions with the galactose ring oxygen and with the C4 hydroxyl. The C4 hydroxyl is further stabilized by His236 and Asn238. Several water molecules also interact with 24a. In particular, the galactose ring oxygen and the C2 hydroxyl group interact with neighbouring water molecules (numbered 1 and 2, Fig. 1A) in monomer A. In monomer B, there is an additional water molecule that enables water-mediated interaction that connects a nitrogen of the C3-triazolyl ring to Asn224. The equivalent site in monomer A reveals very weak electron density but indicates a propensity for a water molecule to be located at that site. The 3-phenyltriazolyl substituent fits nicely into a shallow groove in galectin-4C (Fig. 1B), while the C1-substituted 4-fluorophenyl-triazole group is accommodated within the loop between strands S5 and S6 with the triazole ring forming a slightly tilted T-stacking with the electron-poor rim of the Trp256 indole and where the fluorine atom has potential to interact with the backbone carbonyls of Trp256 and Gly257. Binding affinity assays reveal that 24a exhibits a 20-fold stronger affinity for the galectin-4C (*K*_d_ = 9.5 μM) compared to the galectin-1 (*K*_d_ = 220 μM) and galectin-3 (*K*_d_ = 200 μM) CRDs.

**Fig. 2:**
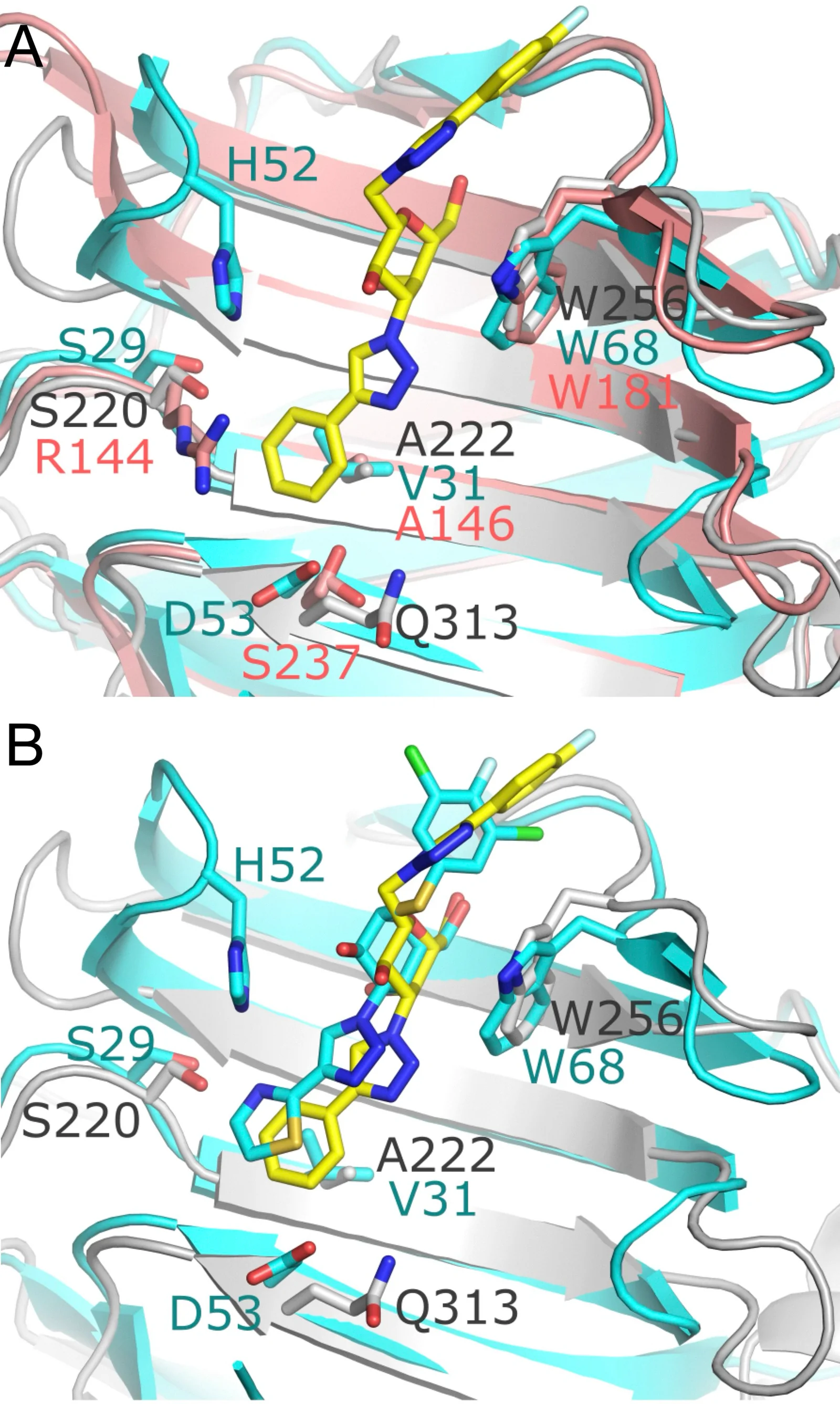
(A) Superimposition of the galectin-4C-24a complex (grey ribbon, PDB: 6WAB) with galectin-1 (cyan ribbon, PDB: 8OJP) and galectin-3 CRD (light pink ribbon, PDB: 7ZQX). Compound 24a is shown as a stick model (carbon (yellow), nitrogen (blue), oxygen (red)). Ser237 in the galectin-3 CRD exhibits a split side chain conformation, with 80% occupancy directed towards the carbohydrate binding site and 20% occupancy flipped away. (B) Superimposition of the galectin-4C-24a complex (grey ribbon, PDB: 6WAB) with the galectin-1-4c complex (cyan ribbon, PDB: 8OJP). Compound 24a is depicted as yellow sticks, while compound 4c is shown as cyan sticks.

To consider how the selectivity of 24a towards galectin-4C is attained, we compared the CRDs of galectin-1 and galectin-3 with galectin-4C to identify subtle differences between these binding sites. Several variations in amino acids within the extended binding site may play a crucial role in the selectivity of 24a towards galectin-4C (Fig. 2A). One notable difference is the substitution of Ala222 in galectin-4C with Val31 in galectin-1. The hydrophobic side chain of valine may alter the binding conformation of the inhibitor within this site. Structural alignment of the galectin-4-24a complex and the galectin-1 in complex with 4c (GB1490, PDB ID 8OJP), a high affinity α-thiophenyl galactoside derivative optimized for galectin-1 binding,^30^ indicates that the thiazole moiety of 4c moved upwards by 1.2 Å compared to the phenyltriazolyl of compound 24a (Fig. 2B). A similar alteration was observed in the 4-fluorophenyl-triazolyl moiety of the TD139 complex with galectin-1 (PDB ID 4Y24).^67^ In galectin-1, the protrusion of His52 from the S4-S5 loop could interfere with the binding of 24a, resulting in weaker binding affinity. In galectin-3 the presence an arginine (Arg144), which instead is a serine in galectin-4C and galectin-1, has potential for the long side chain of Arg144 to interfere with the binding of 24a to galectin-3 CRD also resulting in weaker binding affinity. In the region close to the phenyl group of 24a, it is noted that Asp53 in galectin-1 is one carbon shorter than the corresponding residue Gln313 in galectin-4C. Similarly for galectin-3 the amino acid (Ser237) located at this site has a short side chain and potentially leads to a closer direct hindrance of the phenyl group. The longer side chain of Gln313 is able to swivel to position the sidechain end to sit more distal from the ligand's phenyl group, therefore in galectin-4C there is less restriction on the spatial accommodation of 24a.

## Conclusions

The synthesis of a C1-aryltriazole galactopyranosyl compound panel allowed a detailed galectin-1 and 3 SAR to be established for ligand interactions in sub-sites near the galactose anomeric position. Compounds were discovered (1c and 1z) with similar affinities to, and in one case (1c) better selectivities than, previously reported 1a–1b. However, combining different galectin-1 and galectin-3 selective C1-substituents and known affinity-enhancing 4-aryl-triazolyl substituents at galactose C3 instead lead to surprisingly potent inhibitors of galectin-4C. Rather than the predicted additive affinity and selectivity patterns, an unexpected crosstalk effects between the C1 and C3 moieties was observed. Compounds designed to be selective for galectin-1 (16a–b and 24a–b) turned out to have much stronger galectin-4C affinities, with 24a–b, having particularly good affinities of 9.5 μM and 12 μM, respectively, and up to thirty-sevenfold selectivity for galectin-4C over other galectins. These unexpected affinity and selectivity patterns are probably due to negative cooperativity between the substituents for galectins-1 and -3, but positive cooperativity in the case of galectin-4C. Taken together, these compounds are high affinity and highly selective galectin-4C inhibitors that are the most selective chemical biology tool compounds to date, as well as promising leads for the development of drugs targeting galectin-4-related inflammatory conditions, such as inflammatory bowel disease (IBD) and ulcerative colitis. It remains however to be proven that inhibition of only one domain of the tandem-repeat galectin-4 suffice to block activity in a biological setting. Nevertheless, this is not unlikely, as it has been proven for other tandem-repeat galectins that inhibition^41^ of site-directed mutagenesis^68,69^ of only one is enough to significantly attenuate biological activity.

## Experimental

### General procedures

Chemicals were obtained from Sigma-Aldrich unless otherwise stated and used without further purification, unless stated in the procedure. NMR spectra were collected on a Bruker Ultrashield Plus/Avance II 400 MHz spectrometer. ^1^H spectra were recorded at 400 MHz and ^13^C spectra at 100 MHz with residual solvent signal as references. All final compounds were purified using preparative HPLC on an Agilent 1260 Infinity system with a SymmetryPrep C18 5 μM 19 × 100 mm column using a gradient (water with 0.1% formic acid and acetonitrile); 0–20 minutes 10–100% acetonitrile, 20–23 minutes 100% acetonitrile. Monitoring and collection based on UV/vis absorbance at 210 and 254 nm. Purity analysis and diastereomer ratio measurement was performed using UPLC/MS with UV/vis detection on a Waters Acquity UPLC + Waters XEVO-G2 system using a Waters Acquity CSH C18, 1.7 μm, 2.1 × 100 mm column. Samples were run using a gradient with water (0.1% formic acid) and acetonitrile using a flow rate of 0.50 mL min^−1^ and a column temperature 60 °C. Gradient parameters: 0–0.7 min: 40% acetonitrile, 0.7–10.0 min: 40–99% acetonitrile, 10.0–11.0 min 99% acetonitrile, 11.0–11.1 min 99–40% acetonitrile, 11.1–13 min 40% acetonitrile, 3 or 6 μL injection, detection 190–300 nm. MS parameters: cap voltage 3.0 kV, cone voltage 40 kV, Ext 4, source temp 120 °C, desolvation temp 500 °C, cone gas 50, desolvation gas 800, centroid resolution mode, *m/z* interval 50–1200, lockspray. Calibration: Leu-Enkephalin *m/z* 556.2771, 0.25 s every 30 s, average three scans. For optical rotation measurements, samples were dissolved in an appropriate solvent to a concentration of 2–10 mg mL^−1^. Polarimetry was performed on a PerkinElmer Model 341 polarimeter using a sodium lamp and measuring at 589 nM with a 90 mm long 1 mL cell at 20 °C.

### Representative procedure for synthesis of 1c–y

#### 4-(1-Deoxy-β-d-galactopyranosyl)-1-(4-bromophenyl)-1*H*-1,2,3-triazole 1c

Compound 2 (25 mg, 0.070 mmol) was dissolved in dry acetonitrile (2 mL) with copper(i) iodide (3 mg, 0.014 mmol) followed by addition of 4-bromophenyl azide in *tert*-butyl methyl ether (0.5 M, 0.15 mL, 0.077 mmol) and triethylamine (20 μL, 0.140 mmol). The reaction was heated to 50 °C under nitrogen for 30 minutes, then poured into ethyl acetate (20 mL) and washed with brine (20 mL). The brine was extracted twice with ethyl acetate (20 mL), the organic phases pooled, dried with anhydrous sodium sulfate and evaporated. The crude was dissolved in 2 mL of dry methanol with sodium methoxide (23 mg, 0.420 mmol) and left for 1 hour under nitrogen. The reaction was quenched by addition of Amberlite IR-120 (H^+^ form) until pH 7, filtered and evaporated. The crude was purified by column chromatography (5 : 1 dichloromethane/methanol) followed by prep-HPLC (20 minute gradient from 10% acetonitrile/90% water with 0.1% formic acid to 100% acetonitrile) to give 1c (2 mg, 7%) as a clear solid product. [*α*]_D_^20^ = 25° (*c* = 0.1, methanol). ^1^H NMR (MeOD-d4): 8.59 (s, 1H), 7.85–7.75 (m, 4H), 4.45 (d, *J* = 10.2 Hz, H1), 4.01–3.94 (m, 2H), 3.80 (dd, *J* = 13.1 Hz, 8.5 Hz, 1H, H6), 3.76–3.70 (m, 2H), 3.64 (dd, *J* = 9.7 Hz, 3.4 Hz, 1H, H3). ^13^C NMR (MeOD-d4): 147.4, 132.7, 122.0, 121.8, 79.7, 74.9, 74.8, 70.9, 69.5, 61.5. HRMS: M + H (C_14_H_17_BrN_3_O_5_); 386.0359 found, 386.0352 calculated. Purity by HPLC (UV/vis detector 254 nm): 99.9%.

#### 4-Methylphenyl 3-azido-2,4,6-tri-*O*-benzyl-1,3-dideoxy-1-tolylthio-β-d-galactopyranoside 8

Compound 7^28,62^ (1.57 g, 5.04 mmol) was dissolved of dry tetrahydrofuran (70 mL) and cooled to 0 °C under nitrogen and sodium hydride (60% in oil, 0.805 g, 20.20 mmol) was added in portions. After the end of hydrogen evolution, benzyl bromide (2.39 mL, 20.20 mmol) was added, the reaction was heated to reflux for 48 h. After completion the reaction was poured into dichloromethane (200 mL) and washed with saturated ammonium chloride solution (200 mL), which was extracted once with dichloromethane (200 mL). The pooled organics were washed with brine, which was extracted once with dichloromethane (200 mL). All organics were pooled, dried with sodium sulfate, filtered and evaporated. Flash chromatography (5 : 1 heptane/ethyl acetate) gave 8 (2.79 g, yield 95%). The product is a slightly yellow very viscous oil. [*α*]_D_^20^ = −34° (*c* = 1.4 in acetonitrile). ^1^H NMR (CDCl_3_): 7.52–7.45 (m, 4H), 7.43–7.26 (m, 13H), 7.07–7.01 (m, 2H), 4.95 (d, *J* = 10.0 Hz, 1H), 4.92 (d, *J* = 11.5 Hz, 1H), 4.72 (d, *J* = 9.8 Hz, 1H), 4.65–4.57 (m, 2H), 4.52–4.42 (m, 2H), 3.94 (d, *J* = 2.9 Hz, 1H, H4), 3.89 (t, *J* = 9.8 Hz, 1H, H2), 3.66 (s, 3H), 3.56 (dd, *J* = 9.8, 2.9 Hz, 1H, H3), 2.34 (s, 3H). ^13^C NMR (CDCl_3_): 138.13, 137.74, 137.54, 132.64, 132.15, 129.88, 129.72, 128.70, 128.47, 128.45, 128.35, 128.27, 128.06, 127.92, 127.91, 127.88, 127.69, 88.39, 77.56, 77.36, 76.50, 75.74, 75.44, 75.20, 75.01, 73.59, 73.40, 68.50, 68.31, 67.23, 21.14. HRMS: [M + H^+^] (C_34_H_36_N_3_O_4_S): 582.2430 found, 582.2421 expected.

#### 4-Methylphenyl 2,4,6-tri-*O*-benzyl-1,3-dideoxy-3-(4-(phenyl)-1*H*-1,2,3-triazolyl)-1-thio-β-d-galactopyranoside 9

Compound 8 (1.00 g, 1.719 mmol) and copper(i) iodide (66 mg, 0.344 mmol) were dissolved in of dry acetonitrile (35 mL) under nitrogen. Phenylacetylene (225 μL, 2.063 mmol) triethylamine and (478 μL, 3.438 mmol) were added, after which the reaction mixture left overnight. After completion the reaction was poured into ethyl acetate (100 mL) and washed with brine (100 mL) which was extracted twice with ethyl acetate (100 mL). The organics were pooled, dried with sodium sulfate, filtered and evaporated. The crude was purified with column chromatography (3 : 1 heptane/ethyl acetate) which gave 9 (0.99 g, yield 84%. The product is a white solid. [*α*]_D_^20^ = 79° (*c* = 0.97 in acetonitrile). ^1^H NMR: 7.66–7.60 (m, 3H), 7.58–7.53 (m, 2H), 7.44–7.28 (m, 8H), 7.19–7.04 (m, 6H), 6.96–6.91 (m, 2H), 4.97 (dd, *J* = 10.5, 3.2 Hz, 1H, H3), 4.81–4.73 (m, 2H), 4.60–4.48 (m, 2H), 4.45 (d, *J* = 10.9 Hz, 1H), 4.26–4.16 (m, 2H), 4.13 (d, *J* = 2.8 Hz, 1H, H4), 3.98 (d, *J* = 10.4 Hz, 1H), 3.93 (t, *J* = 6.8 Hz, 1H, H2), 3.82–3.76 (m, 2H), 2.38 (s, 3H). ^13^C NMR: 147.61, 137.87, 137.43, 137.12, 136.78, 132.31, 130.48, 129.86, 129.56, 129.23, 128.68, 128.53, 128.49, 128.47, 128.28, 128.06, 128.00, 127.95, 127.92, 125.70, 121.82, 119.02, 88.91, 77.56, 76.39, 75.28, 74.94, 74.49, 73.64, 67.83, 66.25, 21.20. HRMS: [M + H^+^] (C_42_H_42_N_3_O_4_): 684.2908 found, 684.2891 expected.

#### 2,4,6-Tri-*O*-benzyl-3-deoxy-3-(4-(phenyl)-1*H*-1,2,3-triazolyl)-β-d-galactopyranoside 10

Compound 9 (0.99 g, 0.219 mmol) was dissolved in 22 : 1 acetone/water (23 mL) and *N*-bromosuccinimide (0.77 g, 0.658 mmol) was added in one portion. The solution immediately went from clear to orange, the solution gradually losing color over 10 minutes after which the reaction was complete. The reaction was poured into ethyl acetate (100 mL) and washed with saturated sodium bicarbonate solution (100 mL), which was extracted once with ethyl acetate (100 mL). The ethyl acetate was pooled and washed with brine (200 mL) which was extracted once with ethyl acetate (100 mL). The ethyl acetate was pooled again, dried with sodium sulfate, filtered and evaporated. The crude was purified with column chromatography (1 : 1 heptane/ethyl acetate) to give 10 (0.77 g, yield 92%). Product is a white solid that is a 1 : 1 mixture of anomers. [*α*]_D_^20^ = 111° (*c* = 0.44 in acetonitrile). ^1^H NMR: 7.70–7.66 (m, 2H), 7.65–7.61 (m, 2H), 7.55 (s, 1H), 7.46–7.39 (m, 5H), 7.38–7.28 (m, 13H), 7.26–7.18 (m, 9H), 7.14–7.04 (m, 8H), 7.03–6.99 (m, 2H), 5.52 (d, *J* = 3.4, H2), 5.27 (dd, *J* = 11.6, 3.1 Hz, 1H, H3′), 4.94–4.88 (m, 2H), 4.79 (d, *J* = 11.6 Hz, 1H), 4.60–4.42 (m, 8H), 4.35 (dd, *J* = 11.3, 3.3 Hz, 1H, H3), 4.27–4.21 (m, 2H), 4.13 (dd, *J* = 3.1, 0.7 Hz, 1H, H4′), 4.13 (dd, *J* = 3.2, 0.6 Hz, 1H, H4), 4.01 (dd, *J* = 10.6, 7.3 Hz,1H, H6), 3.94 (t, *J* = 6.6 Hz, 1H, H5), 3.90–3.85 (m, 2H), 3.74–3.61 (m, 4H). ^13^C NMR: 137.33, 128.71, 128.65, 128.55, 128.51, 128.49, 128.46, 128.38, 128.33, 128.29, 128.09, 128.03, 127.95, 127.92, 127.89, 125.71, 125.67, 119.26, 119.16, 90.41, 77.35, 76.34, 75.58, 75.41, 74.20, 74.02, 73.58, 72.67, 72.20, 68.69, 68.11, 68.04, 64.30, 60.34. HRMS: [M + H^+^] (C_35_H_36_N_3_O_5_): 578.2657 found, 578.2649 expected.

#### 2,4,6-Tri-*O*-benzyl-3-deoxy-3-(4-(phenyl)-1*H*-1,2,3-triazolyl)-β-d-galactonolactone 11

Compound 10 (768 mg, 1.329 mmol) and Dess–Martin periodinane (732 mg, 1.728 mmol) were dissolved in dry dichloromethane (40 mL) and left overnight. The reaction mixture was evaporated on silica powder (5 g) and purified with column chromatography (2 : 1 heptane/ethyl acetate) to give 11 (765 mg, yield of 99%). The product is a white, fluffy solid. [*α*]_D_^20^ = 180° (*c* = 0.26 in acetonitrile). ^1^H NMR(CDCl_3_): 7.66–7.61 (m, 2H), 7.47–7.41 (m, 2H), 7.40–7.23 (m, 10H), 7.19–7.14 (m, 3H), 7.13–7.09 (m, 2H), 7.07–7.02 (m, 2H), 5.18 (dd, *J* = 10.9, 2.7 Hz, 1H, H3), 5.03 (d, *J* = 11.1 Hz, 1H), 4.81 (d, *J* = 4.5 Hz, 1H), 4.78 (d, *J* = 4.5 Hz, 1H, H2), 4.71 (td, *J* = 7.2, 1.6 Hz, 1H, H5), 4.62–4.51 (m, 2H), 4.67 (d, *J* = 10.9 Hz, 1H), 4.43 (t, *J* = 1.8 Hz, 1H, H4), 4.12 (d, *J* = 11.6 Hz, 1H), 3.83–3.78 (m, 2H). ^13^C NMR(CDCl_3_): 168.37, 136.85, 135.78, 130.14, 129.00, 128.73, 128.65, 128.58, 128.49, 128.46, 128.35, 128.28, 128.08, 128.04, 125.70, 118.51, 77.90, 77.34, 75.68, 74.83, 74.41, 73.83, 71.80, 66.68, 63.05. HRMS: [M + H^+^] (C_35_H_34_N_3_O_5_): 576.2505 found, 576.2493 expected.

#### 2,4,6-Tri-*O*-benzyl-3-deoxy-1-*C*-((trimethylsilyl)-ethynyl)-3-(4-(phenyl)-1*H*-1,2,3-triazolyl)-α-d-galactopyranose 12

Cerium(iii) chloride heptahydrate (990 mg, 2.658 mmol) was dried under vacuum overnight at 110 °C, after which it was cooled to 0 °C, flushed with nitrogen and dry tetrahydrofuran (7.5 mL) was added. The mixture was left to warm to room temperature over two hours. Ethynyltrimethylsilane (411 μL, 2.924 mmol) was dissolved in dry tetrahydrofuran (4 mL) under nitrogen, cooled to −78 °C after which *n*-butyllithium solution in hexanes (2.5 M, 1.17 mL, 2.924 mmol) was added slowly. The reaction was left for 1 h to generate lithium trimethylsilyl acetyllide. The cerium mixture was cooled to −78 °C and the lithium trimethylsilyl acetylide was added slowly, after which the mixture was left for 1 h at −78 °C. 11 (765 mg, 1.329 mmol) was dissolved in dry tetrahydrofuran (7.5 mL) and added slowly to the cerium mixture. The reaction was left to warm to room temperature over 12 hours. The reaction mixture was poured into ethyl acetate (100 mL) and washed with brine (100 mL) (5M hydrochloric acid added in small portions until pH ∼5). The brine was extracted two times with ethyl acetate (100 mL). The organics were pooled, dried with sodium sulfate, filtrated and evaporated. Column chromatography (2 : 1 heptane/ethyl acetate) gave 12 (694 mg, yield of 78%). The product is a clear, amorphous solid that is a 3 : 2 mixture of the anomers. [*α*]_D_^20^ = 148° (*c* = 0.45 in acetonitrile). ^1^H NMR (CDCl_3_): 7.68–7.57 (m, 3H major + 2H minor), 7.46–7.18 (m, 11H major + 12H minor), 7.17–7.07 (m, 5H major + 5H minor), 7.05–7.01 (m, 2H minor), 7.00–6.96 (m, 2H major), 5.15 (dd, *J* = 11.0, 3.3 Hz, 1H, H3 major), 5.09 (dd, *J* = 11.2, 3.2 Hz, 1H minor), 4.91 (d, *J* = 5.3 Hz, 1H minor), 4.88 (d, *J* = 4.6 Hz, 1H major), 4.70 (d, *J* = 11.9 Hz, 1H), 4.63–4.29 (m, 5H major + 6H minor), 4.26 (d, *J* = 10.8 Hz, 1H minor), 4.16 (d, *J* = 11.5 Hz, 1H minor), 4.11 (dd, *J* = 3.3, 1.0 Hz, 1H major), 4.09 (dd, *J* = 3.2, 1.0 Hz, 1H minor), 3.92 (d, *J* = 10.6 Hz, 1H major), 3.83–3.67 (m, 4H major + 1H minor), 0.29 (s, 9H minor), 0.24 (s, 9H major). ^13^C NMR: 147.45, 137.43, 137.07, 136.93, 136.82, 136.54, 130.55, 128.77, 128.67, 128.60, 128.51, 128.47, 128.36, 128.34, 128.19, 128.14, 128.10, 127.95, 127.83, 127.61, 125.74, 125.69, 119.33, 103.35, 98.97, 96.31, 94.60, 91.00, 90.32, 77.23, 76.49, 76.33, 76.04, 75.50, 75.29, 75.13, 73.71, 73.58, 73.47, 73.28, 70.10, 67.73, 67.49, 63.55, 61.55, −0.23, −0.46. HRMS: [M + H^+^] (C_40_H_44_N_3_O_5_Si): 674.3046 found, 674.3045 expected.

#### 2,4,6-Tri-*O*-benzyl-1,3-dideoxy-1-*C*-(trimethylsilyl)-ethynyl-3-(4-(phenyl)-1*H*-1,2,3-triazolyl)-β-d-galactopyranosyl 13

Compound 12 (107 mg, 0.159 mmol) was dissolved in dry 7 : 3 acetonitrile/dichloromethane (2 mL) under nitrogen and cooled to 0 °C. Triethylsilane (33 μL, 0.207 mmol) dissolved in dry 2 : 1 acetonitrile/dichloromethane (0.5 mL) was added, followed by slow addition of of boron trifluoride diethyl etherate (30 μL, 0.477 mmol) dissolved in of dry 2 : 1 acetonitrile/dichloromethane (0.5 mL). After addition, the reaction mixture was allowed to warm to room temperature and left for 16 hours. After completion, the reaction mixture was poured into dichloromethane (10 mL) & washed with saturated sodium bicarbonate (10 mL) which was extracted with dichloromethane (10 mL). The pooled organics were then washed with brine which was extracted with dichloromethane (10 mL), pooled again, dried with sodium sulfate, filtered and evaporated. The crude was purified using column chromatography (3 : 1 heptane/ethyl acetate) to give 13 (63 mg, 60% yield). [*α*]_D_^20^ = 104° (*c* = 0.59 in acetonitrile). ^1^H NMR (CDCl_3_): 7.67–7.60 (m, 3H), 7.45–7.39 (m, 2H), 7.38–7.22 (m, 9H), 7.16–7.07 (m, 5H), 7.01–6.96 (m, 2H), 4.90–4.83 (m, 2H), 4.59–4.46 (m, 3H), 4.37 (d, *J* = 10.7 Hz, 1H), 4.33–4.22 (m, 2H), 4.12 (d, *J* = 2.8 Hz, 1H, H4), 3.91–3.82 (m, 2H), 3.76–3.71 (m, 2H), 0.23 (s, 9H). ^13^C NMR(CDCl_3_): 147.46, 137.33, 136.83, 136.74, 130.48, 128.67, 128.53, 128.47, 128.43, 128.31, 128.20, 128.10, 128.05, 128.03, 127.98, 125.70, 119.17, 101.82, 92.49, 76.36, 75.58, 75.38, 74.63, 73.63, 71.42, 67.53, 65.43, −0.34. HRMS: [M + H^+^] (C_40_H_44_N_3_O_4_Si): 658.3105 found, 658.3096 expected.

#### 2,4,6-Tri-*O*-benzyl-1,3-dideoxy-1-*C*-ethynyl-3-(4-(phenyl)-1*H*-1,2,3-triazolyl)-β-d-galactopyranosyl 14

Compound 13 (275 mg, 0.408 mmol) was dissolved in tetrahydrofuran (10 mL) and tetrabutylammonium fluoride in tetrahydrofuran (1 M, 0.82 mL, 0.817 mmol) was added. After 10 minutes, the reaction was completed, after which it was poured into dichloromethane (20 mL) and washed with brine (20 mL) which was extracted with dichloromethane (20 mL). The organics were pooled, dried with anhydrous sodium sulfate, filtered and evaporated. The crude was purified using column chromatography (2 : 1 heptane/ethyl acetate) which gave 14 (219 mg, yield 91%). The product is a white solid. [*α*]_D_^20^ = 126° (*c* = 0.26 in acetonitrile). ^1^H NMR (CDCl_3_): 7.69–7.61 (m, 3H), 7.46–7.39 (m, 2H), 7.39–7.22 (m, 9H), 7.17–7.05 (m, 5H), 7.02–6.96 (m, 2H), 4.88 (dd, *J* = 10.3, 3.1 Hz, 1H, H3), 4.81 (d, *J* = 10.8 Hz, 1H), 4.60–4.47 (m, 2H), 4.44 (d, *J* = 10.8 Hz, 1H), 4.39 (d, *J* = 10.8 Hz, 1H), 4.31 (t, *J* = 9.4 Hz, 1H, H2), 4.24 (dd, *J* = 9.0, 2.2 Hz, 1H, H1), 4.12 (d, *J* = 2.7 Hz, 1H, H4), 3.95–3.84 (m, 2H), 3.77–3.70 (m, 2H). ^13^C NMR (CDCl_3_): 137.29, 136.83, 136.61, 130.47, 128.68, 128.54, 128.48, 128.32, 128.30, 128.10, 128.04, 127.97, 125.69, 80.55, 77.44, 76.34, 75.54, 75.35, 74.78, 73.65, 70.81, 67.58, 65.40. HRMS: [M + H^+^] (C_37_H_36_N_3_O_4_): 586.2710 found, 586.2700 expected.

#### 2,4,6-Tri-*O*-benzyl-1,3-dideoxy-1-(1-(2-fluorophenyl)-1*H*-1,2,3-triazol-4-yl)-3-(4-(phenyl)-1*H*-1,2,3-triazolyl)-β-d-galactopyranosyl 15a

Compound 14 (50 mg, 0.761 mmol) and copper(i) iodide (3 mg, 0.015 mmol) was dissolved in dry acetonitrile (10 mL) after which 2-fluorophenyl azide in *tert*-butyl methyl ether (0.5 M, 183 μL, 0.913 mmol) and triethylamine (21 μL, 0.152 mmol) was added. The reaction was left under nitrogen for 48 h, after which the reaction was poured into dichloromethane (25 mL) and washed with brine (25 mL), which was extracted with dichloromethane (25 mL). The organics were pooled and dried with anhydrous sodium sulfate, filtered and evaporated. The crude was purified using column chromatography (3 : 2 heptane/ethyl acetate) to give 15a (40 mg, yield 73%). The product is a white, powdery solid. [*α*]_D_^20^ = 79° (*c* = 0.52 in acetonitrile). ^1^H NMR (CDCl_3_): 8.12 (d, 2.6 Hz, 1H), 7.98 (td, *J* = 7.9, 1.6 Hz, 1H), 7.91 (s, 1H), 7.73–7.68 (m, 2H), 7.53–7.25 (m, 14 H), 7.21–7.15 (m, 2H), 7.08–6.98 (m, 3H), 6.77–6.72 (m, 2H), 5.15 (dt, *J* = 7.7, 2.8 Hz, 1H, H3), 4.85–4.77 (m, 2H), 4.60–4.44 (m, 3H), 4.24 (d, *J* = 3.3 Hz, 1H, H4), 4.15–4.06 (m, 2H), 4.02 (d, *J* = 10.8 Hz, 1H), 3.92 (d, *J* = 10.8 Hz, 1H), 3.81–3.71 (m, 2H). ^13^C NMR (CDCl_3_): 137.38, 130.46, 128.73, 128.52, 128.35, 128.16, 128.09, 127.96, 127.86, 125.70, 125.36, 125.04, 119.49, 116.98, 77.85, 76.71, 75.80, 75.61, 75.06, 74.37, 73.63, 67.87, 66.17. HRMS: [M + H^+^] (C_43_H_40_FN_6_O_4_): 723.3100 found, 723.3090 expected.

#### 2,4,6-Tri-*O*-benzyl-1,3-dideoxy-1-(1-(4-chloro-2-pyridyl)-1*H*-1,2,3-triazol-4-yl)-3-(4-(phenyl)-1*H*-1,2,3-triazolyl)-β-d-galactopyranosyl 15b

Compound 14 (50 mg, 0.085 mmol), 2-azido-5-chloropyridine (16 mg, 0.103 mmol) and copper(i) iodide (3 mg, 0.017 mmol) was dissolved in dry acetonitrile (10 mL) after which triethylamine (24 μL, 0.171 mmol) was added. The reaction was heated to 55 °C under nitrogen and left for 48 hours. Upon completion, the reaction was poured into dichloromethane (25 mL) and washed with brine (25 mL), which was extracted with dichloromethane (25 mL). The organics were pooled, dried with anhydous sodium sulfate, filtered and evaporated. The crude was purified using column chromatography (3 : 2 heptane/ethyl acetate) to give 15b (46 mg, yield 73%). The product is a white solid. [*α*]_D_^20^ = 72° (*c* = 0.51 in acetonitrile). ^1^H NMR (CDCl_3_): 8.63 (s, 1H), 8.49 (dd, *J* = 2.6, 0.7 Hz, 1H), 8.22 (dd, *J* = 8.7, 0.7 Hz, 1H), 7.93 (dd, *J* = 8.5, 2.6 Hz, 1H), 7.84 (s, 1H), 7.72–7.67 (m, 2H), 7.46–7.39 (m, 2H), 7.38–7.24 (m, 11H), 7.21–7.14 (m, 2H), 7.04–6.95 (m, 3H), 6.78–6.73 (m, 2H), 5.15 (dd, *J* = 9.9, 3.3, 1H, H3), 4.84–4.72 (m, 2H), 4.60–4.43 (m, 3H), 4.24 (d, *J* = 3.1 Hz, 1H, H4), 4.13–4.05 (m, 2H), 4.02 (d, 11.0 Hz, 1H), 3.93 (d, *J* = 10.7 Hz, 1H), 3.80–3.70 (m, 2H).^13^C NMR (CDCl_3_): 147.62, 147.40, 145.91, 138.98, 137.37, 136.97, 131.66, 130.47, 128.73, 128.52, 128.33, 128.12, 128.08, 127.98, 127.95, 127.81, 125.70, 121.00, 119.46, 114.68, 77.78, 75.88, 75.59, 75.09. 74.29, 73.63, 67.85, 66.12. HRMS: [M + H^+^] (C_42_H_40_N_7_O_4_): 740.2764 found, 740.2747 expected.

#### 1,3-Dideoxy-1-(1-(2-fluorophenyl)-1*H*-1,2,3-triazol-4-yl)-3-(4-(phenyl)-1*H*-1,2,3-triazolyl)-β-d-galactopyranosyl 16a

Compound 15a (36 mg, 0.050 mmol) was dissolved in 4.5 mL of 2 : 1 cyclohexene/ethanol and palladium hydroxide on carbon (20 wt%, 21 mg, 0.026 mmol) was added. The reaction was refluxed under nitrogen in a sealed tube overnight. Upon completion, he reaction mixture was filtered through Celite, which was washed with methanol, and the evaporated. The crude was purified with column chromatography (11 : 1 dichloromethane/methanol) and then prep-HPLC (10 minute gradient from 10% acetonitrile/90% water with 0.1% formic acid to 100% acetonitrile) to give 16a (19 mg, yield 84%). The product is a white solid. [*α*]_D_^20^ = 53° (*c* = 0.38 in methanol) ^1^H NMR (MeOD-d4): 8.55 (d, *J* = 2.4, 1H), 8.51 (s, 1H), 7.93–7.84 (m, 3H), 7.64–7.57 (m, 1H), 7.51–7.41 (m, 4H), 7.36 (tt, *J* = 7.4, 2.0 Hz, 1H), 5.03 (dd, *J* = 9.7, 2.9 Hz, 1H, H3), 4.81–4.71 (m, 2H), 4.27 (d, *J* = 2.9 Hz, 1H, H4), 4.02 (t, *J* = 6.1, 1H, H5), 3.85 (dd, *J* = 11.3, 6.7 Hz, 1H, H6), 3.77 (dd, *J* = 11.4, 5.2, 1H, H6). ^13^C NMR (MeOD-d4): 155.41, 152.92, 146.99, 146.35, 130.99, 130.91, 128.59, 127.86, 125.26, 125.18, 125.15, 125.01, 124.95, 120.54, 116.90, 116.70, 79.90, 75.79, 68.72, 67.81, 67.44, 61.24. HRMS: [M + H^+^] (C_22_H_22_FN6O_4_): 453.1696 found, 453.1681 expected. HPLC purity by UV/vis detector (210, 254 nm): 99.7%.

#### 1,3-Dideoxy-1-(1-(2-pyridyl)-1*H*-1,2,3-triazol-4-yl)-3-(4-(phenyl)-1*H*-1,2,3-triazolyl)-β-d-galactopyranosyl 16b

Compound 15b (39 mg, 0.053 mmol) was dissolved in 2 : 1 cyclohexene/ethanol (6 mL) and palladium hydroxide on carbon (23 mg, 0.028 mmmol) was added. The reaction was refluxed in a sealed tube under nitrogen overnight. Upon completion the reaction mixture was filtered through Celite, which was washed with methanol, and evaporated. The crude was purified using column chromatography (7 : 1 dichloromethane/methanol) and prep-HPLC (10 minute gradient from 10% acetonitrile/90% water with 0.1% formic acid to 100% acetonitrile) to give 16b (11 mg, 48%) as a white solid. [*α*]_D_^20^ = 48° (*c* = 0.14, methanol). ^1^H NMR (DMSO-d6): 8.95 (s, 1H), 8.66–8.60 (m, 2H), 8.19–8.12 (m, 2H), 7.92–7.87 (m, 2H), 7.60–7.55 (m, 1H), 7.50–7.43 (m, 2H), 7.34 (tt, *J* = 7.5, 1.3 Hz, 1H), 4.95 (dd, *J* = 10.0, 3.0 Hz, 1H, H3), 4.79–4.65 (m, 2H), 4.05 (dd, *J* = 6.7, 2.7 Hz, 1H, H4), 3.89 (t, *J* = 6.2 Hz, 1H, H5), 3.58–3.50 (m, 2H). ^13^C NMR (DMSO-d6): 149.48, 149.00, 147.20, 145.98, 131.67, 129.38, 128.09, 125.46, 124.82, 121.53, 114.12, 79.81, 75.54, 68.47, 67.56, 66.95, 60.95. HRMS: [M + H^+^] (C_21_H_22_N_7_O_4_): 436.1736 found, 436.1728 expected. HPLC purity by UV/vis detector (210, 254 nm): 99.6%.

#### 2,4,6-Tri-*O*-benzyl-1,3-dideoxy-1-(2-(1-naphthoyl)-oxazo-5-yl)-1*H*-1,2,3-triazol-4-yl)-3-(4-(phenyl)-1*H*-1,2,3-triazolyl)-β-d-galactopyranosyl 17

Compound 14 (164 mg, 0.280 mmol), 8-methylquinoline *N*-oxide (67 mg, 0.420 mmol) [Bis(trifluoromethanesulfonyl)imidate](triphenylphosphine)gold(i) (2 : 1) toluene adduct (22 mg, 0.028 mmol) and 1-cyanonaphthalene (172 mg, 1.120 mmol) were sealed in a tube under nitrogen and heated to 55 °C to create a neat melt. The reaction was left for 72 hours, after which it was poured into ethyl acetate (50 mL) and washed with brine (50 mL) which was extracted with ethyl acetate (50 mL). The organics were pooled, dried with anhydrous sodium sulfate, filtered and evaporated. The crude was purified using column chromatography (2 : 1 heptane/ethyl acetate) to give 17 (85 mg, yield of 40%). The product is a lightly yellow solid. [*α*]_D_^20^ = 78° (*c* = 0.087 in acetonitrile). ^1^H NMR (CDCl_3_): 9.32 (d, *J* = 8.6 Hz, 1H), 8.26 (dd, *J* = 7.3, 1.2 Hz, 1H), 8.01 (d, *J* = 8.2 Hz, 1H), 7.95 (d, *J* = 7.8 Hz, 1H), 7.86 (s, 1H), 7.74–7.69 (m, 2H), 7.67 (td, *J* = 7.9, 1.5 Hz, 1H), 7.63–7.56 (m, 2H), 7.49 (s, 1H), 7.46–7.40 (m, 2H), 7.39–7.25 (m, 8H), 7.24–7.17 (m, 3H), 7.01–6.96 (m, 3H), 6.73–6.67 (m. 2H), 5.12 (dd, *J* = 10.4, 3.0 Hz, 1H, H3), 4.71 (d, *J* = 9.2 Hz, 1H, H1), 4.66–4.45 (m, 4H), 4.25 (d, *J* = 3.0, 1H, H4), 4.12–4.00 (m, 3H), 3.93 (d, *J* = 11.0 Hz, 1H), 3.82–3.72 (m, 2H). ^13^C NMR (CDCl_3_): 137.31, 136.16, 133.95, 131.59, 130.43, 128.74, 128.61, 128.55, 128.35, 128.29, 128.27, 128.16, 128.06, 127.98, 127.73, 126.39, 126.11, 125.71, 124.89, 119.31, 77.96, 77.34, 75.50, 74.88, 74.36, 74.29, 73.68, 67.74, 66.07. HRMS: [M + H^+^] (C_48_H_43_N_4_O_5_): 755.3270 found, 755.3228 expected.

#### 1,3-Dideoxy-1-(2-(1-naphthoyl)-oxazo-5-yl)-1*H*-1,2,3-triazol-4-yl)-3-(4-(phenyl)-1*H*-1,2,3-triazolyl)-β-d-galactopyranosyl 18

Compound 17 (7 mg, 0.009 mmol) was dissolved in 2 : 1 cyclohexene/ethanol (3 mL) and palladium hydroxide on carbon (20 wt%, 8 mg, 0.011 mmol) was added. The reaction was refluxed in a sealed tube under nitrogen for 48 hours. Upon completion, the reaction mixture was filtered through Celite, which was washed with methanol and evaporated. The crude was purified using column chromatography (9 : 1 dichloromethane/methanol) and prep-HPLC (10 minute gradient from 10% acetonitrile/90% water with 0.1% formic acid to 100% acetonitrile) to give 18 (2 mg, yield of 45%). The product is a very slightly yellow solid. [*α*]_D_^20^ = 24° (*c* = 0.10 in methanol). ^1^H NMR (MeOD-d4): 9.1 (d, *J* = 8.7 Hz, 1H), 8.54 (s, 1H), 8.27 (dd, *J* = 7.3, 1.2 Hz, 1H), 8.08 (d, *J* = 8.2 Hz, 1H), 7.91–7.86 (m, 2H), 7.70–7.58 (m. 3H), 7.54 (s, 1H), 7.50–7.43 (m, 2H), 7.36 (tt, *J* = 7.4, 1.2 Hz, 1H), 5.03 (dd, *J* = 10.3, 3.0 Hz, 1H, H3), 4.86-4.75 (m, 2H), 4.28 (dd, *J* = 3.0, 0.6 Hz, 1H, H4), 4.03 (tt, *J* = 6.1, 1.0 Hz, 1H, H5), 3.86 (dd, *J* = 11.4, 6.7 Hz, H6), 3.78 (dd, *J* = 11.4, 5.4 Hz, H6). ^13^C NMR (MeOD-d4): 149.35, 134.08, 131.23, 130.55, 128.58, 128.31, 127.93, 127.87, 127.19, 127.12, 126.11, 125.52, 125.27, 124.69, 120.54, 79.93, 75.01, 68.57, 67.83, 65.99, 61.16. HRMS: [M + H^+^] (C_27_H_25_N_4_O_5_): 485.1825 found, 485.1819 expected. HPLC purity by UV/vis detector (210, 254 nm): 96.9.

#### 1-((4-(1-Phenyl-1*H*-1,2,3-triazol-4-yl)-1,3-dideoxy-β-d-galactopyranosyl)methyl)-4-(4-fluorophenyl)-1*H*-1,2,3-triazole 24a

Compound 23a (89 mg, 0.121 mmol) was dissolved in 2 : 1 cyclohexene/ethanol (4 mL) palladium hydroxide on carbon (20 wt%, 67 mg, 0.084 mmol) and refluxed overnight. The reaction mixture was filtered through Celite which was washed with methanol and evaporated. Column chromatography (9 : 1 dichloromethane/methanol) gave 24a (33 mg yield 59%). Product is a white powdery solid. 9 mg was purified using prep-HPLC (10 minute gradient from 10% acetonitrile/90% water with 0.1% formic acid to 100% acetonitrile) before affinity testing. [*α*]_D_^20^ = −1° (*c* = 0.63 in methanol). ^1^H NMR (CDCl_3_): 8.45–8.42 (m, 2H), 7.91–7.83 (m, 4H), 7.48–7.42 (m, 2H), 7.36 (tt, *J* = 7.4 Hz, 1.3 Hz, 1H), 7.23–7.18 (m, 2H), 5.03 (dd, *J* = 14.3 Hz, 2.3 Hz, 1H, H1), 4.71 (dd, *J* = 14.3 Hz, 7.8 Hz, 1H, H1), 4.27 (t, *J* = 10.0 Hz, 1H, H3), 4.16 (d, *J* = 2.8 Hz, 1H, H5), 3.91–3.89 (m, 1H, H2), 3.84–3.69 (m, 3H). ^13^C NMR (CDCl_3_): 164.0, 161.5, 147.0, 146.5, 130.5, 128.6, 127.9, 127.3, 127.2, 126.90, 126.86, 125.3, 125.2, 120.5, 115.5, 115.3, 79.6, 79.2, 68.5, 67.7, 65.2, 61.2, 51.3 HRMS: [M + H^+^] (C_23_H_24_FN_6_O_4_): 467.1837 found, 467.1838 expected. HPLC purity by UV/vis detector (210, 254 nm): 99.5%.

#### 1-((4-(1-Phenyl-1*H*-1,2,3-triazol-4-yl)-1,3-dideoxy-β-d-galactopyranosyl)methyl)-4-(4-(trifluoromethyl)phenyl)-1*H*-1,2,3-triazole 24b

Compound 23b (45 mg, 0.057 mmol) was dissolved in 2 : 1 cyclohexene/ethanol (4 mL) with palladium hydroxide on carbon (20 wt%, 32 mg, 0.046 mmol) and refluxed overnight. The reaction mixture was filtered through Celite which was washed with methanol and evaporated. Column chromatography (9 : 1 dichloromethane/methanol) gave 24b (19 mg, yield 63%). Product is a white amorphous solid. 8 mg was purified using prep-HPLC (10 minute gradient from 10% acetonitrile/90% water with 0.1% formic acid to 100% acetonitrile) before affinity testing. [*α*]_D_^20^ = −5° (*c* = 0.25 in methanol). ^1^H NMR (MeOD-d4): 8.60 (s, 1H), 8.45 (s, 1H), 8.09-8.04 (m, 2H), 7.88–7.83 (m, 2H), 7.79–7.74 (m, 2H), 7.49–7.42 (m, 2H), 7.36 (tt, *J* = 7.4 Hz, 2.1 Hz, 1H), 5.05 (dd, *J* = 14.5 Hz, 2.2 Hz, 1H, H1), 4.74 (dd, *J* = 14.4 Hz, 7.6 Hz, 1H, H1), 4.28 (t, *J* = 10.0 Hz, 1H, H3), 4.17 (d, *J* = 2.7 Hz, 1H, H5), 3.94–3.86 (m, 1H, H2), 3.85–3.70 (m, 3H). ^13^C NMR (MeOD-d4): 147.0, 145.9, 134.3, 130.5, 128.6, 127.9, 125.7, 125.52, 125.48, 125.3, 123.4, 120.5, 79.5, 79.3, 68.5, 67.7, 65.2, 61.2, 51.3. HRMS: [M + H^+^] (C_24_H_24_F_3_N_6_O_4_): 517.1810 found, 517.1806 expected. HPLC purity by UV/vis detector (210, 254 nm): 99.5%.

#### 1-((4-(1-Phenyl-1*H*-1,2,3-triazol-4-yl)-1,3-dideoxy-β-d-galactopyranosyl)methyl)-4-phenyl-1*H*-1,2,3-triazole 24c

Compound 23c (29 mg, 0.040 mmol) was dissolved in 2 : 1 cyclohexene/ethanol (4 mL) palladium hydroxide on carbon (20 wt%, 23 mg, 0.032 mmol) and refluxed overnight. The reaction mixture was filtered through Celite which was washed with methanol and evaporated. Column chromatography (9 : 1 dichloromethane/methanol) followed by prep-HPLC (10 minute gradient from 10% acetonitrile/90% water with 0.1% formic acid to 100% acetonitrile) gave 24c (14 mg yield 79%). Product is a white powdery solid. [*α*]_D_^20^ = −1° (*c* = 0.10 in methanol). ^1^H NMR (MeOD-d4): 8.47 (s, 1H), 8.44 (s, 1H), 7.89–7.83 (m, 4H), 7.49–7.42 (m, 4H), 7.40–7.33 (m, 2H), 5.03 (dd, *J* = 14.3 Hz, 2.3 Hz, 1H, H1), 4.93–4.87 (H4, solvent obscured), 4.71 (dd, *J* = 14.2 Hz, 7.7 Hz, 1H, H1), 4.28 (t, *J* = 10.4 Hz, 1H, H3), 4.16 (d, *J* = 2.0 Hz, 1H, H5), 3.91–3.89 (m, 1H, H2), 3.85–3.69 (m, 3H). ^13^C NMR (MeOD-d4): 147.5, 147.0, 130.5, 130.4, 128.58, 128.57, 127.92, 127.86, 125.34, 125.27, 122.3, 120.5, 79.6, 79.2, 68.5, 67.7, 65.3, 61.2, 51.3 HRMS: [M + H^+^] (C_23_H_25_N_6_O_4_): 449.1942 found, 449.1932 expected. HPLC purity by UV/vis detector (210, 254 nm): 99.3%.

#### 1-((4-(1-Phenyl-1*H*-1,2,3-triazol-4-yl)-1,3-dideoxy-β-d-galactopyranosyl)methyl)-4-(3,4,5-trifluorophenyl)-1*H*-1,2,3-triazole 24d

Compound 23d (45 mg, 0.058 mmol) was dissolved in 2 : 1 cyclohexene/ethanol (4 mL) palladium hydroxide on carbon (20 wt%, 33 mg, 0.047 mmol) and refluxed overnight. The reaction mixture was filtered through Celite which was washed with methanol and evaporated. Column chromatography (9 : 1 dichloromethane/methanol) followed by prep-HPLC (10 minute gradient from 10% acetonitrile/90% water with 0.1% formic acid to 100% acetonitrile) gave 24d (12 mg yield 41%). Product is a white powdery solid. [*α*]_D_^20^ = −4° (*c* = 0.12 in methanol). ^1^H NMR (MeOD-d4): 8.53 (s, 1H), 8.43 (s, 1H), 7.88–7.82 (m, 2H), 7.70–7.61 (m, 2H), 7.49–7.42 (m, 2H), 7.36 (tt, *J* = 7.4 Hz, 1.3 Hz), 5.03 (dd, *J* = 14.2 Hz, 2.2 Hz, 1H, H1), 4.92–4.87 (H4, solvent obscured), 4.72 (dd, *J* = 14.2 Hz, 7.7 Hz, 1H, H1), 4.26 (t, *J* = 10.1 Hz, 1H, H3), 4.16 (d, *J* = 2.8 Hz, 1H, H5), 3.90–3.69 (m, 4H). ^13^C NMR (MeOD-d4): 147.1, 130.5, 128.6, 127.9, 125.3, 123.2, 120.5, 109.6, 109.5, 109.42, 109.35, 79.5, 79.3, 68.5, 67.7, 65.2, 61.2, 51.3 HRMS: [M + H^+^] (C_23_H_22_F_3_N_6_O_4_): 503.1655 found, 503.1649 expected. HPLC purity by UV/vis detector (210, 254 nm): 99.7%.

### Expression and purification of galectin-4C

Recombinant human galectin-4 C-terminal carbohydrate recognition domain was expressed and purified as previously reported.^57,70^ Briefly, BL-21 (DE3) *E. coli* cells containing galectin-4C (amino acid sequence 185–323) was expressed, lysed by sonication and purified by affinity chromatography on a lactosyl-Sepharose column. Galectin-4C protein was eluted using 50 mM β-lactose in PBS, pH 7.4. Galectin-4C was then further gel purified using a Superdex 200/60 column (GE Healthcare) previously equilibrated in PBS, pH 7.4. The peak fractions collected containing galectin-4C were pooled and concentrated to 6.5 mg mL^−1^ using an AmiconUltra 3K molecular weight cut-off centrifugal concentrator (Millipore, Billerica, MA, USA) and used directly in crystallisation studies.

### Crystallisation of galectin-4C and protein–ligand complex formation

Crystals were obtained under similar conditions as previously described, with slight changes to the concentration of ammonium sulfate.^70^ Briefly, 2 μL of galectin-4C CRD protein (6.5 mg mL^−1^ in PBS, pH 7.4, with 50 mM lactose) was mixed with 2 μL of reservoir solution (1.6 M ammonium sulfate, 4% (v/v) polyethylene glycol (PEG) 400, 0.1 M HEPES pH 7.0). Crystallisation drops were set up over 500 μL reservoir solution. Crystals appeared within 14–30 days and grew to a typical size of 0.35 mm × 0.2 mm × 0.2 mm in 14–60 days. The use of DMSO to solubilize the ligand 24a led to crystal structures with no observed ligand electron density in the galectin-4C binding site. However, 24a was successfully solubilised in 50% (v/v) PEG400 which led to the determination of the herein reported crystal structure. Galectin-4C lactose co-crystals were transferred to 5 μL drops of reservoir solution and allowed to sit overnight to remove excess lactose. The galectin-4C crystals were then transferred to a drop containing 1 mM 24a ligand and a lower (1.2 M, instead of 1.6 M) concentration of ammonium sulfate, 4% (v/v) polyethylene glycol (PEG) 400, 0.1 M HEPES pH 7.0, sealed over 200 μL of reservoir solution for 180 mins for ligand exchange.

### X-ray diffraction analysis and structure determination

The soaked galectin-4C crystals with 24a were dipped in a cryoprotectant solution (reservoir solution, 20% (v/v) glycerol and 0.5 mM of 24a in PEG 400) for ∼10 s before being flash cooled in liquid nitrogen. X-ray diffraction sets were collected at 100 K at the Australian Synchrotron, MX2 beamline.^71^ The data were integrated using XDS,^72^ scaled and merged using AIMLESS^73^ as implemented in the CCP4 crystallographic suite.^74^ The structures were solved by molecular replacement (residue number 186-323, sequence from PDB ID: 4YM2^70^) using PHASER^75^ and refined using REFMAC5.^76^ The S3-S4 loop of monomer B showed no electron density and the amino acids 228-232 were omitted in the structure refinement. Well-defined electron density for the ligand 24a was revealed in protein monomers A and B. Visualization of the electron density and model building was performed using COOT.^77,78^ The ligand geometry of 24a was generated using PRODRG2 server.^79^ Model analysis and validation was performed using MOLPROBITY. Figures were created using PyMOL (The PyMOL Molecular Graphics System, Version 2.5 Schrödinger, LLC).

## Author contributions

AD designed compounds, performed synthesis, characterized compounds, analysed protein binding data, and wrote the manuscript. RMG and CK performed galectin-4C X-ray data collection, refinement, and analysis. HL developed the protein binding assay and analysed the data. HB analysed the X-ray structure and wrote the manuscript. UJN conceptualized the study, acquired funding, administered the project, analysed the data, and wrote the manuscript. All authors reviewed and edited the manuscript.

## Conflicts of interest

HL and UJN are shareholders in Galecto Biotech Inc. The other authors have no conflicts to declare.

## Supplementary Material

CB-006-D5CB00106D-s001

## Data Availability

Crystallographic data for galectin-4C in complex with 24a has been deposited at the PBD repository under 6WAB and can be obtained from https://www.rcsb.org.
