# Supplementary material for: Synergy of triazolyl substituents at C1 and C3 of galactose for high-affinity and selective galectin-4C inhibition
Source: RSC Chem Biol. 2025 Jul 18;6(9):1437–50. doi: 10.1039/d5cb00106d (PMC12302229; doi:10.1039/d5cb00106d)
Supplement: CB-006-D5CB00106D-s001 [file CB-006-D5CB00106D-s001.pdf]

Supplementary Information (SI) for RSC Chemical Biology.
This journal is © The Royal Society of Chemistry 2025

Supporting information

Synergy of triazolyl substituents at C1 and C3 of galactose for high-affinity and selective galectin-4C inhibition

Alexander Dahlqvist[1], Rob Marc Go[2], Chandan Kishor[2], Hakon Leffler[3], Helen Blanchard[2,4*], Ulf J. Nilsson[1*]

[1]Department of Chemistry, Lund University, Lund, Sweden

[2]Institute for Biomedicine and Glycomics, Griffith University, Queensland, Australia

[3]Division of Microbiology, Immunology and Glycobiology, Lund University, Lund, Sweden

[4]School of Biological and Chemical Sciences, University of Galway, Galway, H91 TK33, Ireland

*Corresponding author: helen.blanchard@universityofgalway.ie, ulf.nilsson@chem.lu.se

General procedures S2

Chemicals S2

NMR procedure S2

Purification of final compounds S2

Purity analysis and HRMS S2

Optical rotation S3

Fluorescence polarization experiments S3

Synthetic procedures and analytical data for **1d-1y** S4

References S25

^{1}H- and ^{13}C-NMR spectra of compounds **8-24,** NOESY of **20** S26

LC-MS analyses of **1c-1z**, **16a-b**, **18**, **24a-d** S128

Table S1. Crystallographic data and refinement statistics S159

General procedures

Chemicals

Chemicals were obtained from Sigma-Aldrich except aryl azides, which were obtained from Enamine and 2,3,4,6-tetrabenzyl-D-galactopyranose, which was obtained from Carbosynth. All chemicals were used without further purification, unless otherwise stated in the procedure.

NMR procedure

NMR spectra were collected on a Bruker Ultrashield Plus/Avance II 400 MHz spectrometer. ^{1}H spectra were recorded at 400 MHz and ^{13}C spectra at 100 MHz with residual solvent signal as references.

Purification of final compounds

All final compounds were purified using preparative HPLC on an Agilent 1260 Infinity system with a SymmetryPrep C18 5 µM 19x100 mm column using a gradient (water with 0.1% formic acid and acetonitrile); 0-20 minutes 10-100% acetonitrile, 20-23 minutes 100% acetonitrile. Monitoring and collection based on UV/VIS absorbance at 210 and 254 nm.

Purity analysis and HRMS

Purity analysis was performed using UPLC/MS with UV/VIS detection on a Waters Acquity UPLC + Waters XEVO-G2 system using a Waters Acquity CSH C18, 1.7 um, 2.1 x100 mm column. Samples were run using a gradient with water

(0.1% formic acid) and acetonitrile using a flow rate of 0.50 mL/min and a column temperature 60°C. Gradient parameters: 0-0.7min: 40% acetonitrile, 0.7-10.0 min: 40-99% acetonitrile, 10.0-11.0 min 99% acetonitrile, 11.0-11.1 min 99-40% acetonitrile, 11.1-13 min 40% acetonitrile, 3 or 6 uL injection, detection 190-300 nm. MS parameters: Cap voltage 3.0 kV, Cone voltage 40 kV, Ext 4, Source temp 120°C, Desolvation temp 500°C, cone gas 50, desolvation gas 800, Centroid resolution mode, m/z interval 50-1200, Lockspray. Calibration: Leu-Enkephalin m/z 556.2771, 0.25 s every 30 s, average 3.

Optical rotation

Samples were dissolved in an appropriate solvent to a concentration of 2-10 mg/mL. Polarimetry was performed on a PerkinElmer Model 341 Polarimeter using a sodium lamp and measuring at 589 nM with a 90 mm long 1 mL cell at 20°C.

Fluorescence polarization experiments

Human galectin-1[1] and galectin-3[2] were expressed and purified as earlier described. Fluorescence polarization experiments were performed on a PheraStarFS plate reader with software PHERAstar Mars version 2.10 R3 (BMG, Offenburg, Germany). Specific experimental conditions were a galectin-1 concentration of 0.5 µM together with the fluorescent probe (3,3'-dideoxy-3-[4-(fluorescein-5-yl-carbonylaminomethyl)-1*H*-1,2,3-triazol-1-yl]-3'-(3,5-di-methoxybenzamido)-1,1'-sulfanediyl-di-β-D-galactopyranoside[1] concentration of 20 nM and a galectin-3 concentration of 0.2 µM together with the fluorescent probe (3,3'-dideoxy-3-[4-(fluorescein-5-yl-carbonylaminomethyl)-1*H*-1,2,3-

triazol-1-yl]-3'-(3,5-di-methoxybenzamido)-1,1'-sulfanediyl-di-β-D-galactopyranoside) concentration of 20 nM, respectively. Inhibitors were dissolved in dimethylsulfoxide (analytical grade) to a concentration of 20 mM, diluted with PBS to 3–6 different concentrations, and tested in duplicate twice for galectin affinity using a competitive fluorescence polarization assay as earlier described.[3] The highest inhibitor concentrations tested were 1 mM due to solubility limitations at higher concentration. Dissociation constants average and SEM were calculated from two to eight single point measurements showing between 20–80% inhibition.

4-(1-Deoxy-β-D-galactopyranosyl)-1-(3-bromophenyl)-1*H*-1,2,3-triazole 1d

Compound **5** (2,3,4,6-tetra-O-acetyl-1-deoxy-1-ethynyl-galactose) (25 mg, 0.070 mmol) was dissolved in dry acetonitrile (2 mL) with copper(I) iodide (3 mg, 0.014 mmol), 3-bromophenyl azide (0.15 ml 0.5 mol/L in tetrabutyl methyl ether, 0.077 mmol) and triethylamine (20 µl, 0.140 mmol) was added. The reaction was heated to 50°C under nitrogen for 30 minutes, then poured into ethyl acetate (20 mL) and washed with brine (20 mL). The brine was extracted twice with ethyl acetate (20 ml), the organic phases pooled, dried with anhydrous sodium sulfate and evaporated. The crude was dissolved in dry methanol (2 mL) with sodium methoxide (23 mg, 0.420 mmol) and left for 1 hour under nitrogen. The reaction was quenched by addition of Amberlite IR 120 (H^+-form) until pH ~7, filtered and evaporated. The crude was purified by column chromatography (5:1 dichloromethane/methanol) followed by prep-HPLC (20 minute gradient from 10% acetonitrile/90% water with 0.1% formic

acid to 100% acetonitrile) to give **1d** (4 mg, 16%). Product is a clear solid. $[\alpha]^{20}_{D}$= 18° (*c*=0.20 in methanol).^{1}H-NMR(MeOD-d4): 8.62 (s, 1H), 8.12 (t, *J*=2.0 Hz, 1H), 7.89 (ddd, *J*= 8.14 Hz, 2.1 Hz, 0.9 Hz, 1H), 7.69 (dq, *J*=8.0 Hz, 0.9 Hz, 1H), 7.53 (t, *J*= 8.1 Hz, 1H), 4.45 (d, *J*=9.7 Hz, **H^{1}**), 4.01-3.94 (m, 2H), 3.81 (dd, *J*=12.2 Hz, 8.1 Hz, 1H, **H^{6}**), 3.77-3.70 (m, 2H), 3.65 (dd, *J*=9.5 Hz, 3.4 Hz, 1H, **H^{3}**) ^{13}C-NMR(MeOD-d4): 147.4, 131.6, 131.2, 123.1, 122.8, 121.6, 118.8, 79.7, 74.9, 74.8, 70.8, 69.6, 61.5. HRMS for $C_{14}H_{17}BrN_3O_5$ [M+H$^+$]; 386.0359 found, 386.0346 calculated. Purity by HPLC (UV/VIS detector 254 nm): 98.9%

4-(1-Deoxy-β-D-galactopyranosyl)-1-(4-iodophenyl)-1*H*-1,2,3-triazole 1e

Compound **5** (2,3,4,6-tetra-O-acetyl-1-deoxy-1-ethynyl-galactose) (25 mg, 0.070 mmol) was dissolved in dry acetonitrile (2 mL) with copper(I) iodide (3 mg, 0.014 mmol), 4-iodophenyl azide (19 mg, 0.077 mmol) and triethylamine (20 µl, 0.140 mmol) was added. The reaction was heated to 50°C under nitrogen for 30 minutes, then poured into ethyl acetate (20 mL) and washed with brine (20 mL). The brine was extracted twice with ethyl acetate (20 ml), the organic phases pooled, dried with anhydrous sodium sulfate and evaporated. The crude was dissolved in dry methanol (2 mL) with sodium methoxide (23 mg, 0.420 mmol) and left for 1 hour under nitrogen. The reaction was quenched by addition of Amberlite IR 120 (H$^+$-form) until pH ~7, filtered and evaporated. The crude was purified by column chromatography (5:1 dichloromethane/methanol) followed by prep-HPLC (20 minute gradient from 10% acetonitrile/90% water with 0.1% formic acid to 100% acetonitrile) to give **1e** (1 mg, 3%). Product is a clear solid. $[\alpha]^{20}_{D}$= 48° (*c*=0.08 in methanol).

^{1}H-NMR(MeOD-d4): 8.58 (s, 1H), 7.99-7.94 (m, 2H), 7.71-7.66 (m, 2H), 4.45 (d, *J*=9.8 Hz, **H^1**), 4.01-3.93 (m, 2H), 3.80 (dd, *J*=12.2 Hz, 8.3, 1H, **H^6**), 3.76-3.70 (m, 2H), 3.64 (dd, *J*=9.4 Hz, 3.4 Hz, 1H, **H^3**). ^{13}C-NMR(MeOD-d4): 138.8, 121.8, 79.7, 74.9, 74.9, 70.9, 69.6, 61.5. HRMS for $C_{14}H_{17}IN_3O_5$ [M+H$^+$]; 434.0223 found, 434.0207 calculated. Purity by HPLC (UV/VIS detector 254 nm): 99.0%

4-(1-Deoxy-β-D-galactopyranosyl)-1-(3-iodophenyl)-1*H*-1,2,3-triazole 1f

Compound **5** (2,3,4,6-tetra-O-acetyl-1-deoxy-1-ethynyl-galactose) (25 mg, 0.070 mmol) was dissolved in dry acetonitrile (2 mL) with copper(I) iodide (3 mg, 0.014 mmol), 3-iodophenyl azide (19 mg, 0.077 mmol) and triethylamine (20 μl, 0.140 mmol) was added. The reaction was heated to 50°C under nitrogen for 30 minutes, then poured into ethyl acetate (20 mL) and washed with brine (20 mL). The brine was extracted twice with ethyl acetate (20 ml), the organic phases pooled, dried with anhydrous sodium sulfate and evaporated. The crude was dissolved in dry methanol (2 mL) with sodium methoxide (23 mg, 0.420 mmol) and left for 1 hour under nitrogen. The reaction was quenched by addition of Amberlite IR 120 (H$^+$-form) until pH ~7, filtered and evaporated. The crude was purified by column chromatography (5:1 dichloromethane/methanol) followed by prep-HPLC (20 minute gradient from 10% acetonitrile/90% water with 0.1% formic acid to 100% acetonitrile) to give **1f** (12 mg, 39%). Product is a clear solid. $[\alpha]^{20}_{D}$= 5° (*c*=0.11 in methanol).

^{1}H-NMR(MeOD-d4): 8.60 (d, *J*=2.4 Hz,1H), 8.28 (t, *J*=1.9 Hz, 1H), 7.91-7.86 (m, 1H), 7.36 (t, *J*=8.1 Hz, 1H), 4.45 (d, *J*=9.9 Hz, **H^1**), 4.01-3.94 (m, 2H), 3.81 (dd, *J*=11.2 Hz, 8.6, 1H, **H^6**), 3.76-3.70 (m, 2H), 3.65 (dd, *J*=9.6 Hz, 3.3 Hz, 1H, **H^3**). ^{13}C-NMR(MeOD-d4): 147.4, 137.8, 137.7, 131.1, 128.9, 121.6, 119.4, 93.7, 79.7, 74.9,

74.8, 70.8, 69.6, 61.5. HRMS for $C_{14}H_{17}IN_3O_5$ [M+H$^+$]; 434.0220 found, 434.0207 calculated. Purity by HPLC (UV/VIS detector 254 nm): 98.8%

4-(1-Deoxy-β-D-galactopyranosyl)-1-(4-trifluoromethylphenyl)-1*H*-1,2,3-triazole 1g

Compound **5** (2,3,4,6-tetra-O-acetyl-1-deoxy-1-ethynyl-galactose) (25 mg, 0.070 mmol) was dissolved in dry acetonitrile (2 mL) with copper(I) iodide (3 mg, 0.014 mmol), 4-(trifluoromethyl)phenyl azide (15 mg, 0.077 mmol) and triethylamine (20 µl, 0.140 mmol) was added. The reaction was heated to 50°C under nitrogen for 30 minutes, then poured into ethyl acetate (20 mL) and washed with brine (20 mL). The brine was extracted twice with ethyl acetate (20 ml), the organic phases pooled, dried with anhydrous sodium sulfate and evaporated. The crude was dissolved in dry methanol (2 mL) with sodium methoxide (23 mg, 0.420 mmol) and left for 1 hour under nitrogen. The reaction was quenched by addition of Amberlite IR 120 (H$^+$-form) until pH ~7, filtered and evaporated. The crude was purified by column chromatography (5:1 dichloromethane/methanol) followed by prep-HPLC (20 minute gradient from 10% acetonitrile/90% water with 0.1% formic acid to 100% acetonitrile) to give **1g** (10 mg, 36%). Product is a clear solid. $[\alpha]^{20}_{D}$= 21° (*c*=0.26 in methanol). ^{1}H-NMR(MeOD-d4): 8.70 (s, 1H), 8.14-8.10 (m, 2H), 7.96-7.91 (m, 2H), 4.48 (d, *J*=9.7 Hz, **H^1**), 4.03-3.96 (m, 2H), 3.81 (dd, *J*=12.2 Hz, 8.2, 1H, **H^6**), 3.77-3.72 (m, 2H), 3.66 (dd, *J*=9.4 Hz, 3.3 Hz, 1H, **H^3**). ^{13}C-NMR(MeOD-d4): 147.7, 126.9, 126.8, 121.6, 120.4, 79.7, 74.9, 74.8, 70.9, 69.6, 61.5. HRMS for $C_{15}H_{17}F_3N_3O_5$ [M+H$^+$]; 376.1125 found, 376.1115 calculated. Purity by HPLC (UV/VIS detector 254 nm): 99.6%

4-(1-Deoxy-β-D-galactopyranosyl)-1-(3-trifluoromethylphenyl)-1*H*-1,2,3-triazole 1h

Compound **5** (2,3,4,6-tetra-O-acetyl-1-deoxy-1-ethynyl-galactose) (25 mg, 0.070 mmol) was dissolved in dry acetonitrile (2 mL) with copper(I) iodide (3 mg, 0.014 mmol), 3-(trifluoromethyl)phenyl azide (15 mg, 0.077 mmol) and triethylamine (20 µl, 0.140 mmol) was added. The reaction was heated to 50°C under nitrogen for 30 minutes, then poured into ethyl acetate (20 mL) and washed with brine (20 mL). The brine was extracted twice with ethyl acetate (20 ml), the organic phases pooled, dried with anhydrous sodium sulfate and evaporated. The crude was dissolved in dry methanol (2 mL) with sodium methoxide (23 mg, 0.420 mmol) and left for 1 hour under nitrogen. The reaction was quenched by addition of Amberlite IR 120 (H^+-form) until pH ~7, filtered and evaporated. The crude was purified by column chromatography (5:1 dichloromethane/methanol) followed by prep-HPLC (20 minute gradient from 10% acetonitrile/90% water with 0.1% formic acid to 100% acetonitrile) to give **1h** (16 mg, 62%). Product is a clear solid. $[\alpha]^{20}_{D}$= 22° (*c*=0.19 in methanol). ^{1}H-NMR(MeOD-d4): 8.71 (s, 1H), 8.24-8.21 (m, 1H), 8.20-8.15 (m, 1H), 7.85-7.79 (m, 2H), 4.48 (d, *J*=9.6 Hz, **H^{1}**), 4.03-3.96 (m, 2H), 3.81 (dd, *J*=12.4 Hz, 8.3, 1H, **H^{6}**), 3.77-3.71 (m, 2H), 3.66 (dd, *J*=9.6 Hz, 3.6 Hz, 1H, **H^{3}**). ^{13}C-NMR(MeOD-d4): 163.2, 147.6, 137.5, 130.8, 125.2, 123.6, 121.7, 116.89, 116.86, 79.7, 74.9, 74.8, 70.9, 69.6, 61.4. HRMS for $C_{15}H_{17}F_3N_3O_5$ [M+H$^+$]; 376.1115 found, 376.1115 calculated. Purity by HPLC (UV/VIS detector 254 nm): 99.1%

4-(1-Deoxy-β-D-galactopyranosyl)-1-(4-nitrophenyl)-1*H*-1,2,3-triazole 1i

Compound **5** (2,3,4,6-tetra-O-acetyl-1-deoxy-1-ethynyl-galactose) (25 mg, 0.070 mmol) was dissolved in dry acetonitrile (2 mL) with copper(I) iodide (3 mg, 0.014 mmol), 4-nitrophenyl azide (13 mg, 0.077 mmol) and triethylamine (20 µl, 0.140 mmol) was added. The reaction was heated to 50°C under nitrogen for 30 minutes, then poured into ethyl acetate (20 mL) and washed with brine (20 mL). The brine was extracted twice with ethyl acetate (20 ml), the organic phases pooled, dried with anhydrous sodium sulfate and evaporated. The crude was dissolved in dry methanol (2 mL) with sodium methoxide (23 mg, 0.420 mmol) and left for 1 hour under nitrogen. The reaction was quenched by addition of Amberlite IR 120 (H^+-form) until pH ~7, filtered and evaporated. The crude was purified by column chromatography (5:1 dichloromethane/methanol) followed by prep-HPLC (20 minute gradient from 10% acetonitrile/90% water with 0.1% formic acid to 100% acetonitrile) to give **1i** (9 mg, 34%). Product is a clear solid. $[\alpha]^{20}_{D}$= 41° (*c*=0.39 in methanol).

^{1}H-NMR(DMSO-d6): 8.77 (s, 1H), 8.52-8.47 (m, 2H), 8.22-8.17 (m, 2H), 4.48 (d, *J*=9.4 Hz, **H^1**), 4.03-3.96 (m, 2H), 3.81 (dd, *J*=12.2 Hz, 8.2, 1H, **H^6**), 3.77-3.71 (m, 2H), 3.66 (dd, *J*=9.1 Hz, 3.1 Hz, 1H, **H^3**). ^{13}C-NMR(DMSO-d6): 148.5, 147.1, 141.5, 126.1, 122.9, 120.9, 80.1, 75.13, 75.10, 70.5, 69.2, 61.3. HRMS for $C_{14}H_{17}N_4O_7$ [M+H$^+$]; 353.1098 found, 353.1092 calculated. Purity by HPLC (UV/VIS detector 254 nm): 95.3%

4-(1-Deoxy-β-D-galactopyranosyl)-1-(3-nitrophenyl)-1*H*-1,2,3-triazole 1j

Compound **5** (2,3,4,6-tetra-O-acetyl-1-deoxy-1-ethynyl-galactose) (25 mg, 0.070 mmol) was dissolved in dry acetonitrile (2 mL) with copper(I) iodide (3 mg, 0.014 mmol), 3-nitrophenyl azide (13 mg, 0.077 mmol) and triethylamine (20 µl,

0.140 mmol) was added. The reaction was heated to 50°C under nitrogen for 30 minutes, then poured into ethyl acetate (20 mL) and washed with brine (20 mL). The brine was extracted twice with ethyl acetate (20 ml), the organic phases pooled, dried with anhydrous sodium sulfate and evaporated. The crude was dissolved in dry methanol (2 mL) with sodium methoxide (23 mg, 0.420 mmol) and left for 1 hour under nitrogen. The reaction was quenched by addition of Amberlite IR 120 (H^+-form) until pH ~7, filtered and evaporated. The crude was purified by column chromatography (5:1 dichloromethane/methanol) followed by prep-HPLC (20 minute gradient from 10% acetonitrile/90% water with 0.1% formic acid to 100% acetonitrile) to give **1j** (12 mg, 48%). Product is a clear solid. $[\alpha]^{20}_{D}$= 31° (*c*=0.32 in methanol).

^{1}H-NMR(MeOD-d4): 8.79-8.75 (m, 2H), 8.38 (ddd, *J*=8.3 Hz, 2.3 Hz, 0.9 Hz, 1H), 8.33 (ddd, *J*=8.2 Hz, 2.1 Hz, 0.9 Hz, 1H), 7.88 (t, *J*=8.3 Hz, 1H), 4.48 (d, *J*=9.9 Hz, **H^1**), 4.04-3.97 (m, 2H), 3.82 (dd, *J*=13.1 Hz, 8.4, 1H, **H^6**), 3.78-3.71 (m, 2H), 3.66 (dd, *J*=9.4 Hz, 3.3 Hz, 1H, **H^3**). ^{13}C-NMR(MeOD-d4): 149.1, 147.8, 137.8, 131.0, 125.7, 122.9, 121.8, 113.9, 79.7, 74.9, 74.8, 70.8, 69.6, 61.5. HRMS for $C_{14}H_{17}N_4O_7$ [M+H$^+$]; 353.1090 found, 353.1092 calculated. Purity by HPLC (UV/VIS detector 254 nm): 95.0%

4-(1-Deoxy-β-D-galactopyranosyl)-1-(2,6-difluorophenyl)-1*H*-1,2,3-triazole 1k

Compound **5** (2,3,4,6-tetra-O-acetyl-1-deoxy-1-ethynyl-galactose) (25 mg, 0.070 mmol) was dissolved in dry acetonitrile (2 mL) with copper(I) iodide (3 mg, 0.014 mmol), 2,6-difluorophenyl azide (12 mg, 0.077 mmol) and triethylamine (20 μl, 0.140 mmol) was added. The reaction was heated to 50°C under nitrogen

for 30 minutes, then poured into ethyl acetate (20 mL) and washed with brine (20 mL). The brine was extracted twice with ethyl acetate (20 ml), the organic phases pooled, dried with anhydrous sodium sulfate and evaporated. The crude was dissolved in dry methanol (2 mL) with sodium methoxide (23 mg, 0.420 mmol) and left for 1 hour under nitrogen. The reaction was quenched by addition of Amberlite IR 120 (H^+-form) until pH ~7, filtered and evaporated. The crude was purified by column chromatography (5:1 dichloromethane/methanol) followed by prep-HPLC (20 minute gradient from 10% acetonitrile/90% water with 0.1% formic acid to 100% acetonitrile) to give **1k** (6 mg, 24%). Product is a clear solid. $[\alpha]^{20}_{D}$= 24° (*c*=0.27 in methanol).

^{1}H-NMR(MeOD-d4): 8.36 (s, 1H), 7.73-7.64 (m, 1H), 7.37-7-29 (m, 2H), 4.50 (d, *J*=9.7 Hz, **H^1**), 4.04-3.96 (m, 2H), 3.82 (dd, *J*=13.5 Hz, 9.0 Hz, 1H, **H^6**), 3.77-3.70 (m, 2H), 3.65 (dd, *J*=9.5 Hz, 3.1 Hz, 1H, **H^3**). ^{13}C-NMR(MeOD-d4): 163.1, 158.3, 155.8, 132.3, 132.2, 132.1, 126.3, 112.41, 112.37, 112.21, 112.17, 79.7, 74.9, 74.8, 70.7, 69.6, 61.6. HRMS for $C_{14}H_{16}F_2N_3O_5$ [M+H$^+$]; 344.1067 found, 344.1053 calculated. Purity by HPLC (UV/VIS detector 254 nm): 100%

4-(1-Deoxy-β-D-galactopyranosyl)-1-(2-fluoro-4-chlorophenyl)-1*H*-1,2,3-triazole 1l

Compound **5** (2,3,4,6-tetra-O-acetyl-1-deoxy-1-ethynyl-galactose) (25 mg, 0.070 mmol) was dissolved in dry acetonitrile (2 mL) with copper(I) iodide (3 mg, 0.014 mmol), 4-chloro-2-fluorophenyl azide (13 mg, 0.077 mmol) and triethylamine (20 µl, 0.140 mmol) was added. The reaction was heated to 50°C under nitrogen for 30 minutes, then poured into ethyl acetate (20 mL) and washed with brine (20 mL). The brine was extracted twice with ethyl acetate (20

ml), the organic phases pooled, dried with anhydrous sodium sulfate and evaporated. The crude was dissolved in dry methanol (2 mL) with sodium methoxide (23 mg, 0.420 mmol) and left for 1 hour under nitrogen. The reaction was quenched by addition of Amberlite IR 120 (H^+-form) until pH ~7, filtered and evaporated. The crude was purified by column chromatography (5:1 dichloromethane/methanol) followed by prep-HPLC (20 minute gradient from 10% acetonitrile/90% water with 0.1% formic acid to 100% acetonitrile) to give **1l** (12 mg, 47%). Product is a clear solid. $[\alpha]^{20}_{D}$= 18° (*c*=0.48 in methanol). ^{1}H-NMR(MeOD-d4): 8.47 (d, *J*=2.4 Hz,1H), 7.90 (t, *J*= 8.3 Hz, 1H), 7.61 (dd, *J*=10.6 Hz, 2.3 Hz, 1H), 7.51-7.47 (m, 1H), 4.48 (d, *J*=9.8 Hz, **H^{1}**), 4.02-3.95 (m, 2H), 3.81 (dd, *J*=11.2 Hz, 8.3, 1H, **H^{6}**), 3.77-3.70 (m, 2H), 3.65 (dd, *J*=9.3 Hz, 2.8 Hz, 1H, **H^{3}**). ^{13}C-NMR(MeOD-d4): 135.7, 135.6, 126.2, 125.6, 125.5, 124.64, 124.58, 117.6, 117.4, 79.7, 74.8, 70.8, 69.6, 61.5. HRMS for $C_{14}H_{16}ClFN_3O_5$ [M+H$^+$]; 360.0765 found, 360.0757 calculated. Purity by HPLC (UV/VIS detector 254 nm): 99.7%

4-(1-Deoxy-β-D-galactopyranosyl)-1-(5-chloro-2-fluorophenyl)-1*H*-1,2,3-triazole 1m

Compound **5** (2,3,4,6-tetra-O-acetyl-1-deoxy-1-ethynyl-galactose) (25 mg, 0.070 mmol) was dissolved in dry acetonitrile (2 mL) with copper(I) iodide (3 mg, 0.014 mmol), 5-chloro-2-fluorophenyl azide (13 mg, 0.077 mmol) and triethylamine (20 µl, 0.140 mmol) was added. The reaction was heated to 50°C under nitrogen for 30 minutes, then poured into ethyl acetate (20 mL) and washed with brine (20 mL). The brine was extracted twice with ethyl acetate (20 ml), the organic phases pooled, dried with anhydrous sodium sulfate and evaporated. The crude was dissolved in dry methanol (2 mL) with sodium

methoxide (23 mg, 0.420 mmol) and left for 1 hour under nitrogen. The reaction was quenched by addition of Amberlite IR 120 (H^+-form) until pH ~7, filtered and evaporated. The crude was purified by column chromatography (5:1 dichloromethane/methanol) followed by prep-HPLC (20 minute gradient from 10% acetonitrile/90% water with 0.1% formic acid to 100% acetonitrile) to give **1m** (8 mg, 32%). Product is a clear solid. $[\alpha]^{20}_{D}$= 27° (*c*=0.25 in methanol). ^{1}H-NMR(MeOD-d4): 8.50 (d, *J*=2.2 Hz,1H), 7.98 (dd, *J*= 6.4 Hz, 2.7 Hz, 1H), 7.63-7.57 (m, 1H), 7.49 (dd, *J*= 10.4 Hz, 9.4 Hz, 1H), 4.48 (d, *J*=9.7 Hz, $\mathbf{H^1}$), 4.02-3.94 (m, 2H), 3.81 (dd, *J*=12.2 Hz, 8.2, 1H, $\mathbf{H^6}$), 3.77-3.70 (m, 2H), 3.65 (dd, *J*=9.1 Hz, 3.1 Hz, 1H, $\mathbf{H^3}$). ^{13}C-NMR(MeOD-d4): 147.2, 130.5, 130.4, 123.0, 124.7, 124.6, 124.5, 118.5, 118.3, 79.7, 74.9, 74.8, 70.9, 69.6, 61.5. HRMS for $C_{14}H_{16}ClFN_3O_5$ [M+H^+]; 360.0756 found, 360.0757 calculated. Purity by HPLC (UV/VIS detector 254 nm): 98.8%

4-(1-Deoxy-β-D-galactopyranosyl)-1-(3-chloro-2-fluorophenyl)-1*H*-1,2,3-triazole 1n

Compound **5** (2,3,4,6-tetra-O-acetyl-1-deoxy-1-ethynyl-galactose) (25 mg, 0.070 mmol) was dissolved in dry acetonitrile (2 mL) with copper(I) iodide (3 mg, 0.014 mmol), 3-chloro-2-fluorophenyl azide (13 mg, 0.077 mmol) and triethylamine (20 µl, 0.140 mmol) was added. The reaction was heated to 50°C under nitrogen for 30 minutes, then poured into ethyl acetate (20 mL) and washed with brine (20 mL). The brine was extracted twice with ethyl acetate (20 ml), the organic phases pooled, dried with anhydrous sodium sulfate and evaporated. The crude was dissolved in dry methanol (2 mL) with sodium methoxide (23 mg, 0.420 mmol) and left for 1 hour under nitrogen. The reaction

was quenched by addition of Amberlite IR 120 (H^+-form) until pH ~7, filtered and evaporated. The crude was purified by column chromatography (5:1 dichloromethane/methanol) followed by prep-HPLC (20 minute gradient from 10% acetonitrile/90% water with 0.1% formic acid to 100% acetonitrile) to give **1n** (6 mg, 24%). Product is a clear solid. $[\alpha]^{20}_{D}$= 17° (*c*=0.33 in methanol). ^{1}H-NMR(MeOD-d4): 8.50 (d, *J*=2.4 Hz,1H), 7.84 (m, 1H, H6), 7.71 (m, 1H, H4), 7.43 (td, *J*=8.2 Hz, 1.7 Hz, 1H, H5), 4.49 (d, *J*=9.7 Hz, **H^1**), 4.02-3.96 (m, 2H), 3.81 (dd, *J*=12.1 Hz, 8.3, 1H, **H^6**), 3.77-3.70 (m, 2H), 3.65 (dd, *J*=9.4 Hz, 3.5 Hz, 1H, **H^3**). ^{13}C-NMR(MeOD-d4): 131.2, 125.4, 124.7, 124.0, 79.2, 74.9, 70.8, 69.6, 61.5. HRMS for $C_{14}H_{16}ClFN_3O_5$ [M+H$^+$]; 360.0759 found, 360.0757 calculated. Purity by HPLC (UV/VIS detector 254 nm): 95.0%

4-(1-Deoxy-β-D-galactopyranosyl)-1-(4-bromo-2-fluorophenyl)-1*H*-1,2,3-triazole 1o

Compound **5** (2,3,4,6-tetra-O-acetyl-1-deoxy-1-ethynyl-galactose) (25 mg, 0.070 mmol) was dissolved in dry acetonitrile (2 mL) with copper(I) iodide (3 mg, 0.014 mmol), 4-bromo-2-fluorophenyl azide (17 mg, 0.077 mmol) and triethylamine (20 µl, 0.140 mmol) was added. The reaction was heated to 50°C under nitrogen for 30 minutes, then poured into ethyl acetate (20 mL) and washed with brine (20 mL). The brine was extracted twice with ethyl acetate (20 ml), the organic phases pooled, dried with anhydrous sodium sulfate and evaporated. The crude was dissolved in dry methanol (2 mL) with sodium methoxide (23 mg, 0.420 mmol) and left for 1 hour under nitrogen. The reaction was quenched by addition of Amberlite IR 120 (H^+-form) until pH ~7, filtered and evaporated. The crude was purified by column chromatography (5:1

dichloromethane/methanol) followed by prep-HPLC (20 minute gradient from 10% acetonitrile/90% water with 0.1% formic acid to 100% acetonitrile) to give **1o** (7 mg, 25%). Product is a clear solid. $[\alpha]^{20}_{D}$= 30° (*c*=0.40 in methanol). ^{1}H-NMR(MeOD-d4): 8.47 (d, *J*=2.2 Hz,1H), 7.84 (t, *J*=8.1 Hz, 1H $\mathbf{H_A}^{\mathbf{6}}$), 7.75 (dd, *J*=10.5 Hz, 2.1 Hz, 1H, $\mathbf{H_A}^{\mathbf{3}}$), 7.63 (ddd, *J*=8.7 Hz, 2.1 Hz, 1.2 Hz, 1H, $\mathbf{H_A}^{\mathbf{5}}$), 4.47 (d, *J*=9.8 Hz, $\mathbf{H}^{\mathbf{1}}$), 4.02-3.94 (m, 2H), 3.81 (dd, *J*=12.1 Hz, 8.6, 1H, $\mathbf{H}^{\mathbf{6}}$), 3.76-3.70 (m, 2H), 3.64 (dd, *J*=9.5 Hz, 3.3 Hz, 1H, $\mathbf{H}^{\mathbf{3}}$). ^{13}C-NMR(MeOD-d4): 155.1, 152.6, 128.6, 128.5, 126.4, 124.7, 123.0, 122.9, 120.6, 120.3, 79.7, 74.9, 70.8, 69.6, 61.5. HRMS for $C_{14}H_{16}BrFN_3O_5$ [M+H$^+$]; 404.0255 found, 404.0252 calculated. Purity by HPLC (UV/VIS detector 254 nm): 98.8%

4-(1-Deoxy-β-D-galactopyranosyl)-1-(3,4-dichlorophenyl)-1*H*-1,2,3-triazole 1p

Compound **5** (2,3,4,6-tetra-O-acetyl-1-deoxy-1-ethynyl-galactose) (25 mg, 0.070 mmol) was dissolved in dry acetonitrile (2 mL) with copper(I) iodide (3 mg, 0.014 mmol), 3,4-dichlorophenyl azide (12 mg, 0.077 mmol) and triethylamine (20 µl, 0.140 mmol) was added. The reaction was heated to 50°C under nitrogen for 30 minutes, then poured into ethyl acetate (20 mL) and washed with brine (20 mL). The brine was extracted twice with ethyl acetate (20 ml), the organic phases pooled, dried with anhydrous sodium sulfate and evaporated. The crude was dissolved in dry methanol (2 mL) with sodium methoxide (23 mg, 0.420 mmol) and left for 1 hour under nitrogen. The reaction was quenched by addition of Amberlite IR 120 (H$^+$-form) until pH ~7, filtered and evaporated. The crude was purified by column chromatography (5:1 dichloromethane/methanol) followed by prep-HPLC (20 minute gradient from 10% acetonitrile/90% water

with 0.1% formic acid to 100% acetonitrile) to give **1p** (18 mg, 68%). Product is a clear solid. $[\alpha]^{20}_{D}$= 18° (*c*=0.41 in methanol).

^{1}H-NMR(MeOD-d4): 8.63 (s, 1H), 8.13 (d, *J*= 2.3 Hz, 1H), 7.86 (dd, *J*= 8.8 Hz, 2.6 Hz, 1H), 7.76 (d, *J*=8.8 Hz, 1H), 4.45 (d, *J*=9.7 Hz, **H^{1}**), 4.02-3.95 (m, 2H), 3.81 (dd, *J*=12.2 Hz, 8.2, 1H, **H^{6}**), 3.76-3.71 (m, 2H), 3.65 (dd, *J*=9.1 Hz, 3.1 Hz, 1H, **H^{3}**). ^{13}C-NMR(MeOD-d4): 147.6, 136.4, 133.3, 132.3, 131.5, 121.9, 121.6, 119.6, 79.7, 74.9, 74.8 , 70.8, 69.5, 61.5. HRMS for $C_{14}H_{16}Cl_2N_3O_5$ [M+H^{+}]; 376.0471 found, 376.0462 calculated. Purity by HPLC (UV/VIS detector 254 nm): 98.8%

4-(1-Deoxy-β-D-galactopyranosyl)-1-(3-chloro-4-methylphenyl)-1*H*-1,2,3-triazole 1q

Compound **5** (2,3,4,6-tetra-O-acetyl-1-deoxy-1-ethynyl-galactose) (25 mg, 0.070 mmol) was dissolved in dry acetonitrile (2 mL) with copper(I) iodide (3 mg, 0.014 mmol), 3-chloro-4-methylphenyl azide (12 mg, 0.077 mmol) and triethylamine (20 µl, 0.140 mmol) was added. The reaction was heated to 50°C under nitrogen for 30 minutes, then poured into ethyl acetate (20 mL) and washed with brine (20 mL). The brine was extracted twice with ethyl acetate (20 ml), the organic phases pooled, dried with anhydrous sodium sulfate and evaporated. The crude was dissolved in dry methanol (2 mL) with sodium methoxide (23 mg, 0.420 mmol) and left for 1 hour under nitrogen. The reaction was quenched by addition of Amberlite IR 120 (H^{+}-form) until pH ~7, filtered and evaporated. The crude was purified by column chromatography (5:1 dichloromethane/methanol) followed by prep-HPLC (20 minute gradient from 10% acetonitrile/90% water with 0.1% formic acid to 100% acetonitrile) to give **1q** (17 mg, 66%). Product is a clear solid. $[\alpha]^{20}_{D}$= 12° (*c*=0.36 in methanol).

^{1}H-NMR(MeOD-d4): 8.57 (s, 1H), 7.93 (d, *J*=2.1 Hz, 1H), 7.72 (dd, *J*= 8.1 Hz, 2.1 Hz, 1H), 7.53 (d, *J*= 8.6 Hz, 1H), 4.45 (d, *J*=9.9 Hz, $\mathbf{H^1}$), 4.01-3.94 (m, 2H), 3.81 (dd, *J*=12.2 Hz, 8.2, 1H, $\mathbf{H^6}$), 3.76-3.70 (m, 2H), 3.65 (dd, *J*=9.1 Hz, 3.1 Hz, 1H, $\mathbf{H^3}$), 2.47 (s, 3H). ^{13}C-NMR(MeOD-d4): 147.3, 136.8, 131.8, 121.5, 120.5, 118.4, 79.7, 74.9, 74.8, 70.8, 69.6, 61.5, 18.3. HRMS for $C_{15}H_{19}ClN_3O_5$ [M+H$^+$]; 356.1014 found, 356.1008 calculated. Purity by HPLC (UV/VIS detector 254 nm): 99.6%

4-(1-Deoxy-β-D-galactopyranosyl)-1-(4-chloropyrid-2-yl)-1*H*-1,2,3-triazole 1r

Compound **5** (2,3,4,6-tetra-O-acetyl-1-deoxy-1-ethynyl-galactose) (25 mg, 0.070 mmol) was dissolved in dry acetonitrile (2 mL) with copper(I) iodide (3 mg, 0.014 mmol), 2-azido-5-chloropyridine (11 mg, 0.077 mmol) and triethylamine (20 µl, 0.140 mmol) was added. The reaction was heated to 50°C under nitrogen for 30 minutes, then poured into ethyl acetate (20 mL) and washed with brine (20 mL). The brine was extracted twice with ethyl acetate (20 ml), the organic phases pooled, dried with anhydrous sodium sulfate and evaporated. The crude was dissolved in dry methanol (2 mL) with sodium methoxide (23 mg, 0.420 mmol) and left for 1 hour under nitrogen. The reaction was quenched by addition of Amberlite IR 120 (H$^+$-form) until pH ~7, filtered and evaporated. The crude was purified by column chromatography (5:1 dichloromethane/methanol) followed by prep-HPLC (20 minute gradient from 10% acetonitrile/90% water with 0.1% formic acid to 100% acetonitrile) to give **1r** (2 mg, 13%). Product is a clear solid. $[\alpha]^{20}_{D}$= 25° (*c*=0.13 in methanol).

^{1}H-NMR(MeOD-d4): 8.83 (s, 1H), 8.60 (d, *J*=2.5 Hz, 1H, pyridine $\mathbf{H^6}$), 8.21 (dd, *J*=8.7 Hz, 0.6 Hz, 1H, pyridine $\mathbf{H^3}$), 8.13 (dd, *J*= 8.8 Hz, 2.5 Hz, 1H, pyridine $\mathbf{H^4}$),

4.47 (d, *J*=9.7 Hz, **H^1**), 4.02-3.95 (m, 2H), 3.81 (dd, *J*=12.6 Hz, 8.8, 1H, **H^6**), 3.77-3.70 (m, 2H), 3.64 (dd, *J*=9.4 Hz, 3.4 Hz, 1H, **H^3**). ^{13}C-NMR(MeOD-d4): 147.3, 139.2, 128.1, 120.4, 114.5, 79.7, 74.88, 74.87, 70.8, 69.6, 61.5. HRMS for $C_{13}H_{16}ClN_4O_5$ [M+H$^+$]; 343.0817 found, 343.0804 calculated. Purity by HPLC (UV/VIS detector 254 nm): 98.9%

4-(1-Deoxy-β-D-galactopyranosyl)-1-(2-thiazolyl)-1*H*-1,2,3-triazole 1s

Compound **5** (2,3,4,6-tetra-O-acetyl-1-deoxy-1-ethynyl-galactose) (25 mg, 0.070 mmol) was dissolved in dry acetonitrile (2 mL) with copper(I) iodide (3 mg, 0.014 mmol), 2-azidothiazole (11 mg, 0.077 mmol) and triethylamine (20 µl, 0.140 mmol) was added. The reaction was heated to 50°C under nitrogen for 30 minutes, then poured into ethyl acetate (20 mL) and washed with brine (20 mL). The brine was extracted twice with ethyl acetate (20 ml), the organic phases pooled, dried with anhydrous sodium sulfate and evaporated. The crude was dissolved in dry methanol (2 mL) with sodium methoxide (23 mg, 0.420 mmol) and left for 1 hour under nitrogen. The reaction was quenched by addition of Amberlite IR 120 (H$^+$-form) until pH ~7, filtered and evaporated. The crude was purified by column chromatography (5:1 dichloromethane/methanol) followed by prep-HPLC (20 minute gradient from 10% acetonitrile/90% water with 0.1% formic acid to 100% acetonitrile) to give **1s** (2 mg, 8%). Product is a clear solid. $[\alpha]^{20}_{D}$= 15° (*c*=0.17 in methanol).

^{1}H-NMR(MeOD-d4): 8.75 (s, 1H), 7.78 (d, *J*=3.5 Hz, 1H, thiazole **H^4**), 7.60 (d, *J*=3.5 Hz, 1H, thiazole **H^5**), 4.47 (d, *J*=9.8 Hz, **H^1**), 4.01-3.94 (m, 2H), 3.81 (dd, *J*=12.0 Hz, *J*=8.5 Hz, 1H, **H^6**), 3.76-3.70 (m, 2H), 3.64 (dd, *J*=9.6 Hz, *J*=3.2 Hz, 1H, **H^3**). ^{13}C-NMR(MeOD-d4): 157.2, 147.6, 140.4, 120.7, 118.6, 79.8, 74.80, 74.77, 70.8, 69.6,

61.5. HRMS for $C_{11}H_{15}N_4O_5S$ [M+H+]; 315.0764 found, 315.0758 calculated. Purity by HPLC (UV/VIS detector 254 nm): 99.3%

4-(1-Deoxy-β-D-galactopyranosyl)-1-(4-biphenyl)-1*H*-1,2,3-triazole 1t

Compound **5** (2,3,4,6-tetra-O-acetyl-1-deoxy-1-ethynyl-galactose) (25 mg, 0.070 mmol) was dissolved in dry acetonitrile (2 mL) with copper(I) iodide (3 mg, 0.014 mmol), 4-azido-1,1'-biphenyl (13 mg, 0.077 mmol) and triethylamine (20 µl, 0.140 mmol) was added. The reaction was heated to 50°C under nitrogen for 30 minutes, then poured into ethyl acetate (20 mL) and washed with brine (20 mL). The brine was extracted twice with ethyl acetate (20 ml), the organic phases pooled, dried with anhydrous sodium sulfate and evaporated. The crude was dissolved in dry methanol (2 mL) with sodium methoxide (23 mg, 0.420 mmol) and left for 1 hour under nitrogen. The reaction was quenched by addition of Amberlite IR 120 (H^+-form) until pH ~7, filtered and evaporated. The crude was purified by column chromatography (5:1 dichloromethane/methanol) followed by prep-HPLC (20 minute gradient from 10% acetonitrile/90% water with 0.1% formic acid to 100% acetonitrile) to give **1t** (2 mg, 8%). Product is a clear solid. $[\alpha]^{20}_{D}$= 31° (*c*=0.11 in methanol).

^{1}H-NMR(MeOD-d4): 8.62 (s, 1H), 7.98-7.93 (m, 2H), 7.90-7.84 (m, 2H), 7.74-7.69 (m, 2H), 7.53-7.47 (m, 2H), 7.41 (tt, *J*=7.42 Hz, 2.0 Hz, 1H), 4.48 (d, *J*=9.8 Hz, $\mathbf{H^1}$), 4.03-3.97 (m, 2H), 3.82 (dd, *J*=12.8 Hz, 8.2 Hz, 1H, $\mathbf{H^6}$), 3.78-3.72 (m, 2H), 3.66 (dd, *J*=9.4 Hz, 3.3 Hz, 1H, $\mathbf{H^3}$). ^{13}C-NMR(MeOD-d4): 147.3, 141.8, 139.5, 136.1, 128.7, 128.0, 127.6, 126.63, 121.4, 120.5, 79.7, 75.0, 74.9, 70.9, 69.6, 61.5. HRMS for $C_{20}H_{22}N_3O_5$ [M+H+]; 384.1559 found, 384.1554 calculated. Purity by HPLC (UV/VIS detector 254 nm): 99.7%

4-(1-Deoxy-β-D-galactopyranosyl)-1-(5-2,3-dihydro-1*H*-indenyl)-1H-1,2,3-triazole 1u

Compound **5** (2,3,4,6-tetra-O-acetyl-1-deoxy-1-ethynyl-galactose) (25 mg, 0.070 mmol) was dissolved in dry acetonitrile (2 mL) with copper(I) iodide (3 mg, 0.014 mmol), 5-azido-2,3-dihydro-1H-indene (13 mg, 0.077 mmol) and triethylamine (20 µl, 0.140 mmol) was added. The reaction was heated to 50°C under nitrogen for 30 minutes, then poured into ethyl acetate (20 mL) and washed with brine (20 mL). The brine was extracted twice with ethyl acetate (20 ml), the organic phases pooled, dried with anhydrous sodium sulfate and evaporated. The crude was dissolved in dry methanol (2 mL) with sodium methoxide (23 mg, 0.420 mmol) and left for 1 hour under nitrogen. The reaction was quenched by addition of Amberlite IR 120 (H^+-form) until pH ~7, filtered and evaporated. The crude was purified by column chromatography (5:1 dichloromethane/methanol) followed by prep-HPLC (20 minute gradient from 10% acetonitrile/90% water with 0.1% formic acid to 100% acetonitrile) to give **1u** (9 mg, 36%). Product is a clear solid. $[\alpha]^{20}_{D}$= 20° (*c*=0.47 in methanol). ^{1}H-NMR(MeOD-d4): 8.49 (s, 1H), 7.67 (s, 1H), 7.57 (dd, *J*=8.1 Hz, 2.1 Hz, 1H), 7.41 d, *J*=8.0 Hz, 1H), 4.45 (d, *J*=9.7 Hz, **H^1**), 4.01-3.95 (m, 2H), 3.81 (dd, *J*=12.2 Hz, 8.4 Hz, 1H, **H^6**), 3.77-3.70 (m, 2H), 3.64 (dd, *J*=9.1 Hz, 3.6 Hz, 1H, **H^3**), 3.07-2.97 (m, 2H), 2.18 (dt, *J*=14.8 Hz, 7.5 Hz, 2H). ^{13}C-NMR(MeOD-d4): 147.0, 146.1, 145.3, 135.5, 124.9, 121.6, 118.4, 116.4, 79.6, 75.0, 74.9, 70.9, 69.6, 61.5, 32.4, 32.0, 25.3. HRMS for $C_{17}H_{22}N_3O_5$ [M+H$^+$]; 348.1560 found, 348.1554 calculated. Purity by HPLC (UV/VIS detector 254 nm): 99.8%

4-(1-Deoxy-β-D-galactopyranosyl)-1-(4-methylsulfonylphenyl)-1*H*-1,2,3-triazole 1v

Compound **5** (2,3,4,6-tetra-O-acetyl-1-deoxy-1-ethynyl-galactose) (25 mg, 0.070 mmol) was dissolved in dry acetonitrile (2 mL) with copper(I) iodide (3 mg, 0.014 mmol), 4-methylsulfonylphenyl azide (13 mg, 0.077 mmol) and triethylamine (20 µl, 0.140 mmol) was added. The reaction was heated to 50°C under nitrogen for 30 minutes, then poured into ethyl acetate (20 mL) and washed with brine (20 mL). The brine was extracted twice with ethyl acetate (20 ml), the organic phases pooled, dried with anhydrous sodium sulfate and evaporated. The crude was dissolved in dry methanol (2 mL) with sodium methoxide (23 mg, 0.420 mmol) and left for 1 hour under nitrogen. The reaction was quenched by addition of Amberlite IR 120 (H^+-form) until pH ~7, filtered and evaporated. The crude was purified by column chromatography (5:1 dichloromethane/methanol) followed by prep-HPLC (20 minute gradient from 10% acetonitrile/90% water with 0.1% formic acid to 100% acetonitrile) to give **1v** (3 mg, 11%). Product is a clear solid. $[\alpha]^{20}_{D}$= 52° (*c*=0.21 in dimethylsulfoxide).

^{1}H-NMR(DMSO-d6): 8.98 (s, 1H), 8.26-8.21 (m, 2H), 8.17.8.13 (m, 2H), 4.29 (d, *J*=9.9 Hz, 1H, H1), 3.91 (t, *J*=9.7 Hz, 1H, H2), 3.80 (d, *J*=2.5 Hz, 1H, H4), 3.58-3.43 (m, 4H, partially solvent obscured), 3.31 (s, 3H). ^{13}C-NMR(DMSO-d6): 148.3, 140.7, 140.5, 129.6, 122.8, 120.8, 80.0, 75.14, 75.09, 70.4, 69.2, 61.3, 43.9. HRMS for $C_{15}H_{20}N_3O_7S$ [M+H$^+$]; 386.1018 found, 386.1016 calculated. Purity by HPLC (UV/VIS detector 254 nm): 99.9%

4-(1-Deoxy-β-D-galactopyranosyl)-1-(3-methylsulfonylphenyl)-1*H*-1,2,3-triazole 1w

Compound **5** (2,3,4,6-tetra-O-acetyl-1-deoxy-1-ethynyl-galactose) (25 mg, 0.070 mmol) was dissolved in dry acetonitrile (2 mL) with copper(I) iodide (3 mg, 0.014 mmol), 3-methylsulfonylphenyl azide (13 mg, 0.077 mmol) and triethylamine (20 µl, 0.140 mmol) was added. The reaction was heated to 50°C under nitrogen for 30 minutes, then poured into ethyl acetate (20 mL) and washed with brine (20 mL). The brine was extracted twice with ethyl acetate (20 ml), the organic phases pooled, dried with anhydrous sodium sulfate and evaporated. The crude was dissolved in dry methanol (2 mL) with sodium methoxide (23 mg, 0.420 mmol) and left for 1 hour under nitrogen. The reaction was quenched by addition of Amberlite IR 120 (H^+-form) until pH ~7, filtered and evaporated. The crude was purified by column chromatography (5:1 dichloromethane/methanol) followed by prep-HPLC (20 minute gradient from 10% acetonitrile/90% water with 0.1% formic acid to 100% acetonitrile) to give **1w** (14 mg, 52%). Product is a clear solid. $[\alpha]^{20}_{D}$= 15° (*c*=0.42 in dimethylsulfoxide).

^{1}H-NMR(DMSO-d6): 9.00 (s, 1H), 8.95 (t, *J*=2.0 Hz, 1H), 8.31 (ddd, *J*=8.1 Hz, 2.2 Hz, 0.9 Hz, 1H), 8.03 (ddd, *J*=7.8 Hz, 1.8 Hz, 1.0 Hz, 1H), 7.90 (t, *J*=8.3 Hz, 1H), 4.30 (d, *J*=9.2 Hz, 1H, H1), 3.90 (t, *J*=9.7 Hz, 1H, H2), 3.80 (s, 1H, H4), 3.58-3.43 (m, 4H), 3.35 (s, 3H, partially solvent obscured).^{13}C-NMR(DMSO-d6): 148.3, 143.0, 137.6, 131.8, 127.1, 125.1, 122.8, 118.8, 80.0, 75.2, 75.1, 70.6, 69.2, 61.3, 43.7. HRMS for $C_{15}H_{20}N_3O_7S$ [M+H$^+$]; 386.1020 found, 386.1016 calculated. Purity by HPLC (UV/VIS detector 254 nm): 99.9%

4-(1-Deoxy-β-D-galactopyranosyl)-1-(4-acetylphenyl)-1*H*-1,2,3-triazole 1x

Compound **5** (2,3,4,6-tetra-O-acetyl-1-deoxy-1-ethynyl-galactose) (25 mg, 0.070 mmol) was dissolved in dry acetonitrile (2 mL) with copper(I) iodide (3 mg, 0.014 mmol), 1-(4-azidophenyl)ethan-1-one (12 mg, 0.077 mmol) and triethylamine (20 µl, 0.140 mmol) was added. The reaction was heated to 50°C under nitrogen for 30 minutes, then poured into ethyl acetate (20 mL) and washed with brine (20 mL). The brine was extracted twice with ethyl acetate (20 ml), the organic phases pooled, dried with anhydrous sodium sulfate and evaporated. The crude was dissolved in dry methanol (2 mL) with sodium methoxide (23 mg, 0.420 mmol) and left for 1 hour under nitrogen. The reaction was quenched by addition of Amberlite IR 120 (H^+-form) until pH ~7, filtered and evaporated. The crude was purified by column chromatography (5:1 dichloromethane/methanol) followed by prep-HPLC (20 minute gradient from 10% acetonitrile/90% water with 0.1% formic acid to 100% acetonitrile) to give **1x** (10 mg, 24%). Product is a clear solid. $[\alpha]^{20}_{D}$= 36° (*c*=0.49 in dimethylsulfoxide).

^{1}H-NMR(DMSO-d6): 8.95 (s, 1H), 8.18 (dt, *J*=8.9 Hz, 2.0 Hz, 2H), 8.11 (dt, *J*=8.9 Hz, 2.0 Hz, 2H), 4.29 (d, *J*=9.7 Hz, 1H, H1), 3.91 (t, *J*=9.3 Hz, 1H, H2), 3.80 (d, *J*=3.1 Hz, 1H, H4), 3.58-3.43 (m, 4H, partially solvent obscured), 2.65 (s, 3H). ^{13}C-NMR(DMSO-d6): 197.4, 148.2, 140.2, 136.7, 130.6, 122.6, 120.1, 80.1, 75.21, 75.15, 70.5, 69.2, 61.3, 27.3. HRMS for $C_{16}H_{20}N_3O_6$ [M+H$^+$]; 350.1354 found, 350.1347 calculated. Purity by HPLC (UV/VIS detector 254 nm): 99.9%

4-(1-Deoxy- β-D-galactopyranosyl)-1-(3-acetylphenyl)-1*H*-1,2,3-triazole 1y

Compound **5** (2,3,4,6-tetra-O-acetyl-1-deoxy-1-ethynyl-galactose) (25 mg, 0.070 mmol) was dissolved in dry acetonitrile (2 mL) with copper(I) iodide (3 mg, 0.014 mmol), 1-(3-azidophenyl)ethan-1-one (12 mg, 0.077 mmol) and triethylamine (20 µl, 0.140 mmol) was added. The reaction was heated to 50°C under nitrogen for 30 minutes, then poured into ethyl acetate (20 mL) and washed with brine (20 mL). The brine was extracted twice with ethyl acetate (20 ml), the organic phases pooled, dried with anhydrous sodium sulfate and evaporated. The crude was dissolved in dry methanol (2 mL) with sodium methoxide (23 mg, 0.420 mmol) and left for 1 hour under nitrogen. The reaction was quenched by addition of Amberlite IR 120 (H^+-form) until pH ~7, filtered and evaporated. The crude was purified by column chromatography (5:1 dichloromethane/methanol) followed by prep-HPLC (20 minute gradient from 10% acetonitrile/90% water with 0.1% formic acid to 100% acetonitrile) to give **1y** (9 mg, 37%). Product is a clear solid. $[\alpha]^{20}_{D}$= 24° (*c*=0.67 in dimethylsulfoxide).

^{1}H-NMR(DMSO-d6): 8.97 (s, 1H), 8.42 (t, *J*=1.8 Hz, 1H), 8.21 (ddd, *J*=8.1 Hz, 2.3 Hz, 1.0 Hz, 1H), 8.05 (dt, *J*=8.1 Hz, 1.6 Hz, 1H), 7.77 (t, *J*=8.0 Hz, 1H), 4.29 (d, *J*=9.9 Hz, 1H, H1), 3.91 (t, *J*=9.4 Hz, 1H, H2), 3.80 (d, *J*=3.1 Hz, 1H, H4), 3.58-3.43 (m, 4H), 2.69 (s, 3H). ^{13}C-NMR(DMSO-d6): 197.7, 148.1, 138.7, 137.5, 131.0, 128.4, 124.7, 122.6, 119.8, 80.0, 75.3, 75.2, 70.5, 69.2, 61.3, 27.5. HRMS for $C_{16}H_{20}N_3O_6$ [M+H$^+$]; 350.1351 found, 350.1347 calculated. Purity by HPLC (UV/VIS detector 254 nm): 98.8%

References

1. E. Salomonsson, A. Larumbe, J. Tejler, E. Tullberg, H. Rydberg, A. Sundin, A. Khabut, T. Frejd, Y. D. Lobsanov, J. M. Rini, U. J. Nilsson and H. Leffler, *Biochemistry*, 2010, **49**, 9518-9532.
2. S. M. Massa, D. N. Cooper, H. Leffler and S. H. Barondes, *Biochemistry*, 1993, **32**, 260-267.
3. P. Sorme, B. Kahl-Knutsson, M. Huflejt, U. J. Nilsson and H. Leffler, *Anal. Biochem.*, 2004, **334**, 36-47.

1c ^{1}H-NMR

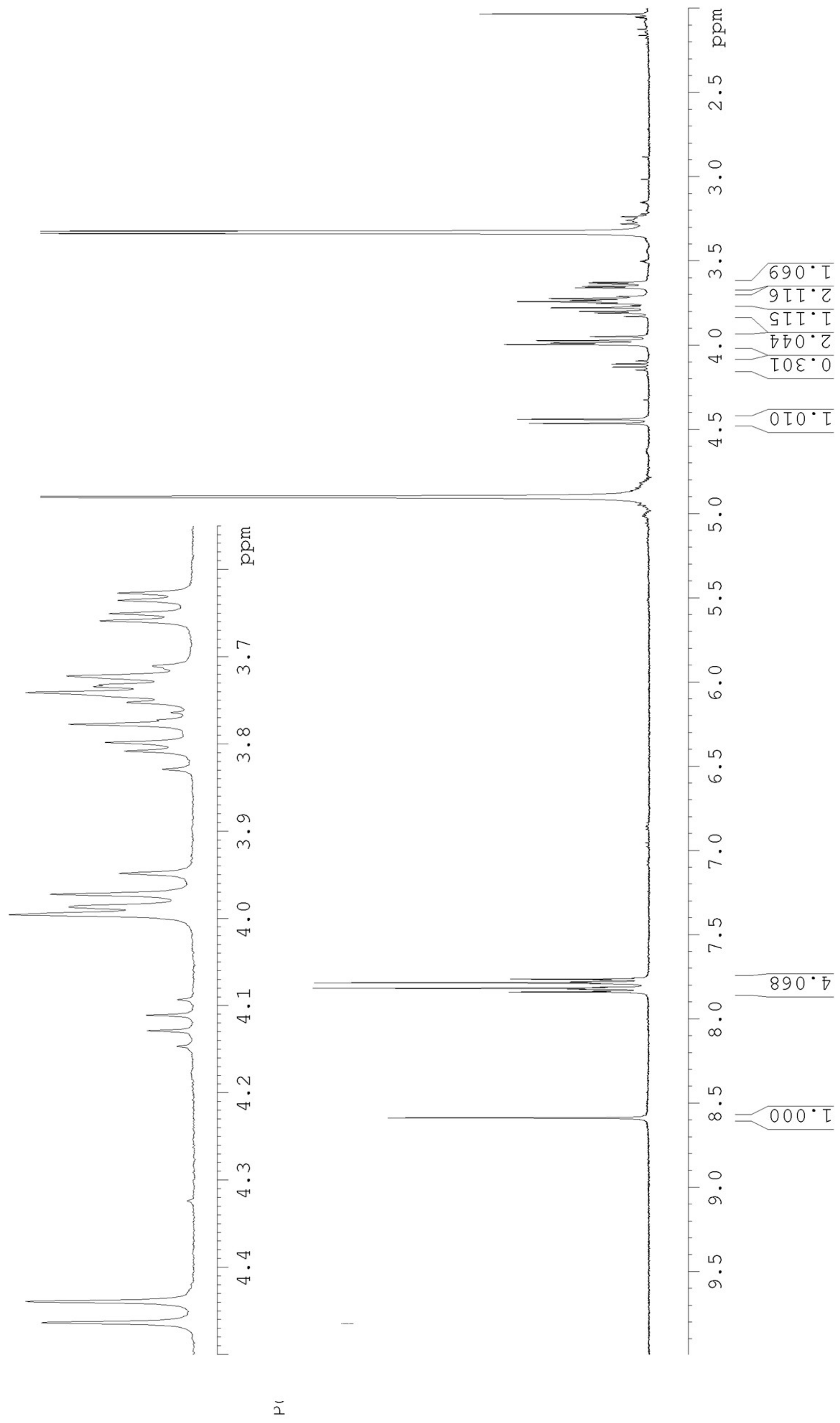

1c ^{13}C-NMR

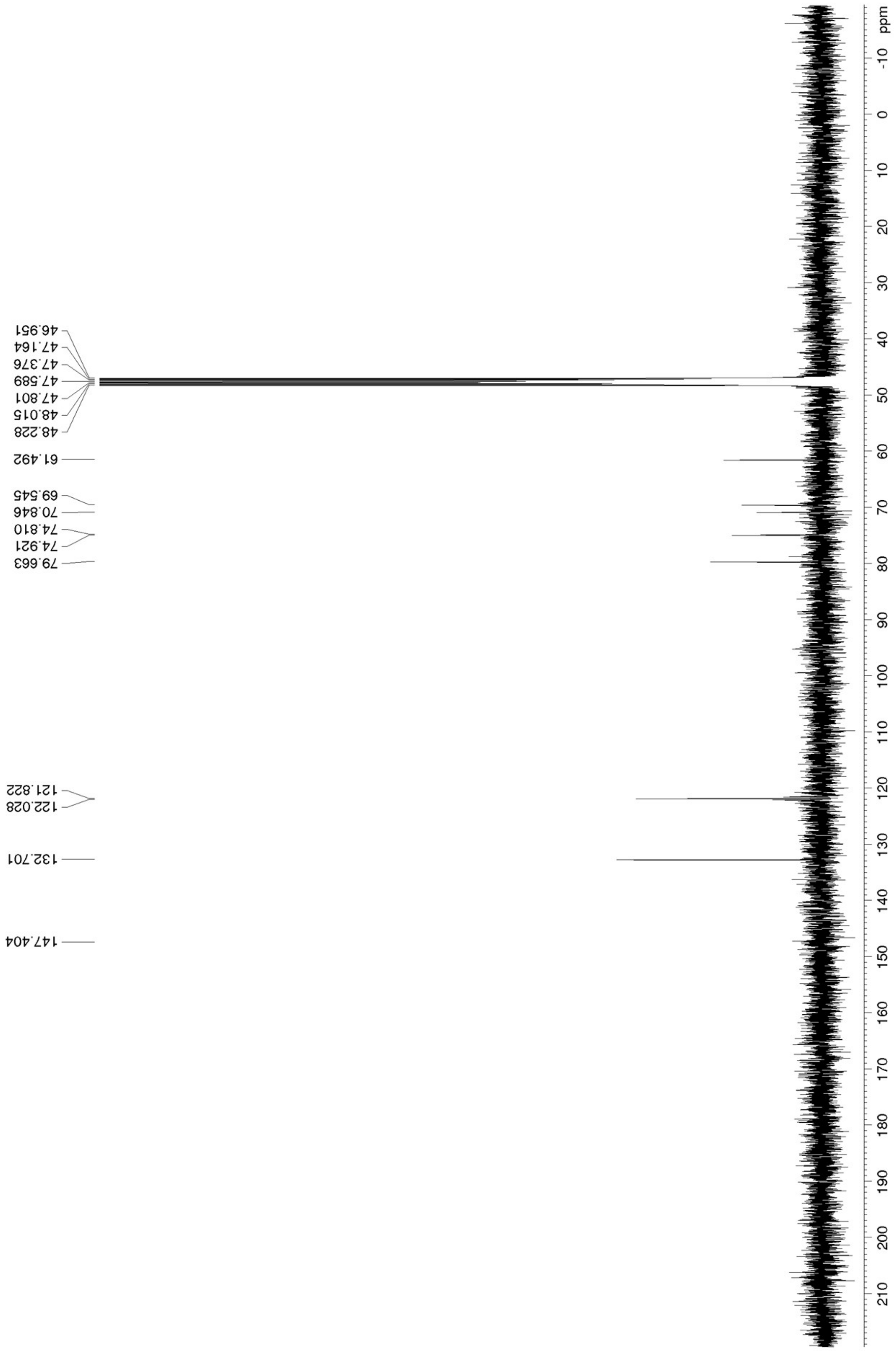

46.951
47.164
47.376
47.589
47.801
48.015
48.228
61.492
69.545
70.846
74.810
74.921
79.663
121.822
122.028
132.701
147.404
ppm

1d ^{1}H-NMR

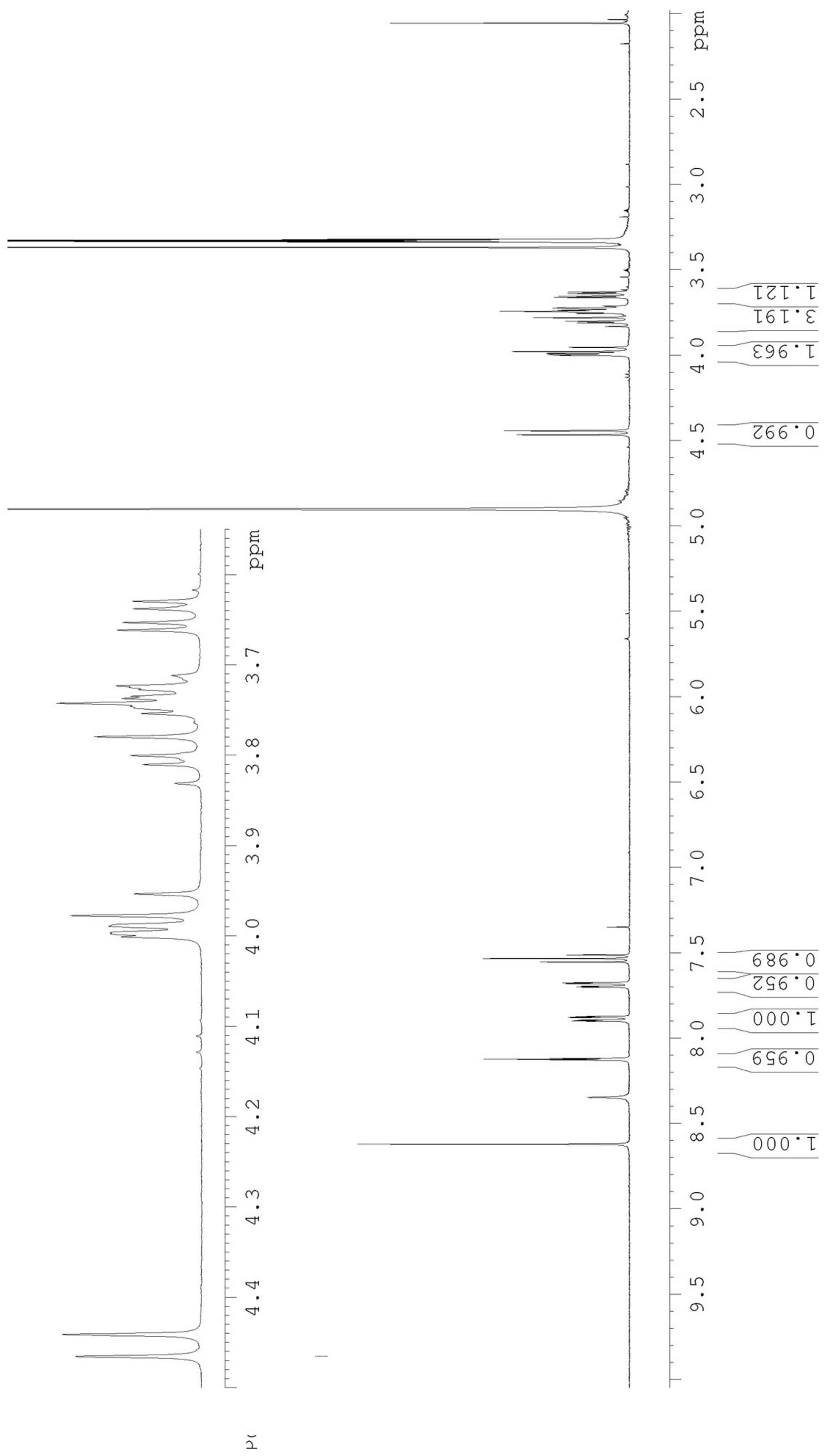

1d ^{13}C-NMR

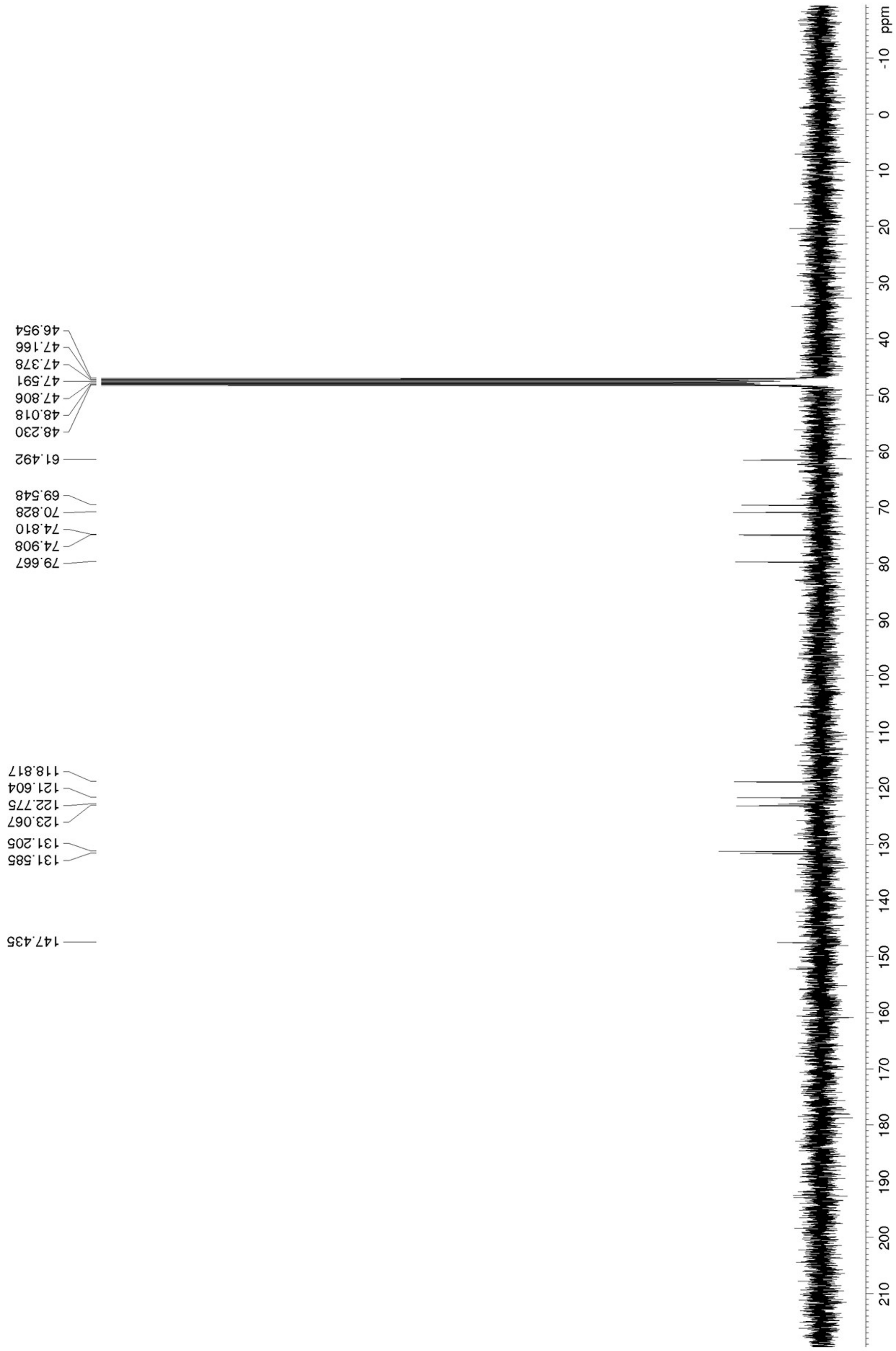
46.954
47.166
47.378
47.591
47.806
48.018
48.230
61.492
69.548
70.828
74.810
74.908
79.667
118.817
121.604
122.775
123.067
131.205
131.585
147.435
ppm

1e ^{1}H-NMR

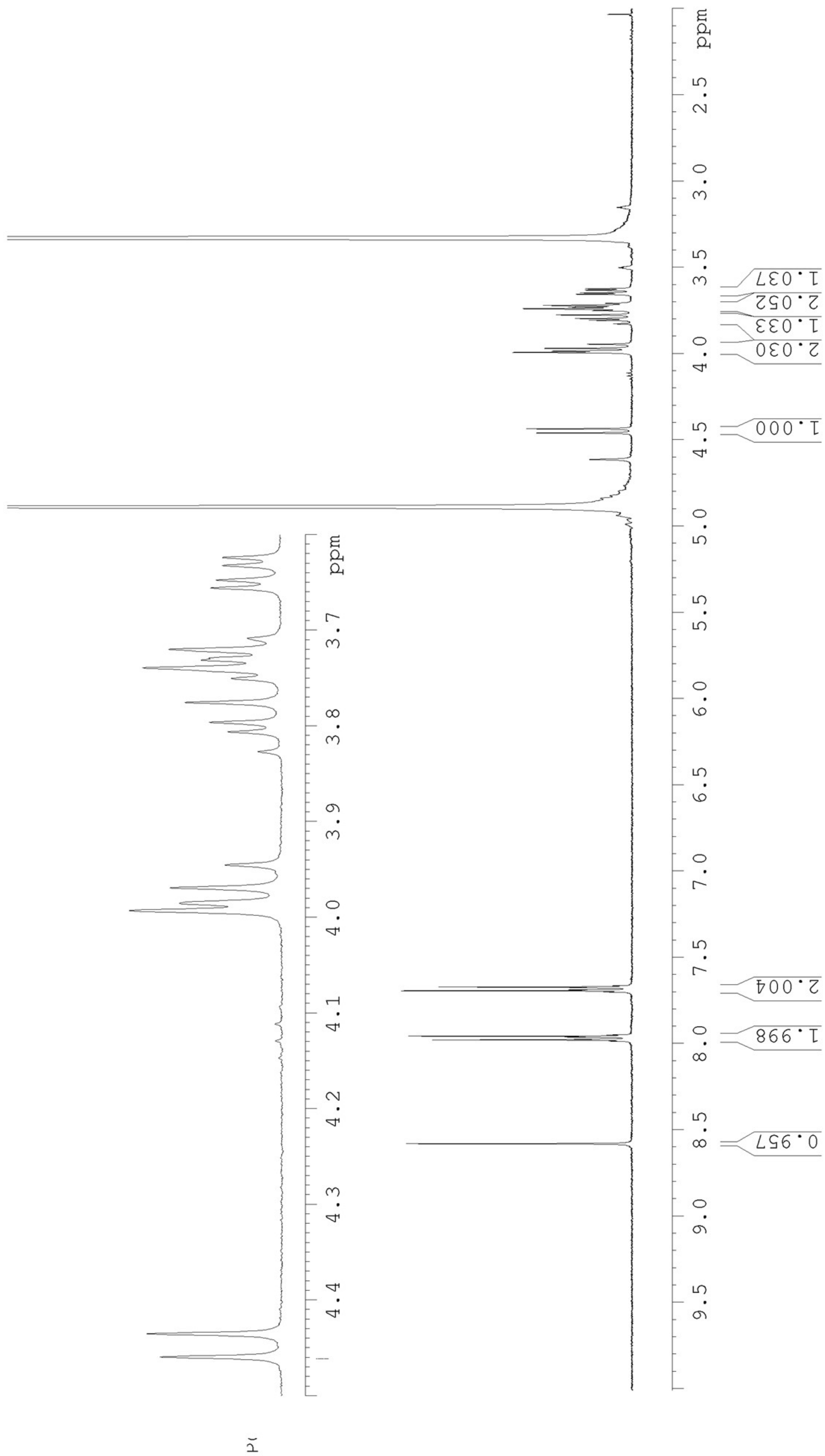

ppm
2.5
3.0
3.5
4.0
4.5
5.0
5.5
6.0
6.5
7.0
7.5
8.0
8.5
9.0
9.5
1.037
2.052
1.033
2.030
1.000
2.004
1.998
0.957
ppm
3.7
3.8
3.9
4.0
4.1
4.2
4.3
4.4

1e ^{13}C-NMR

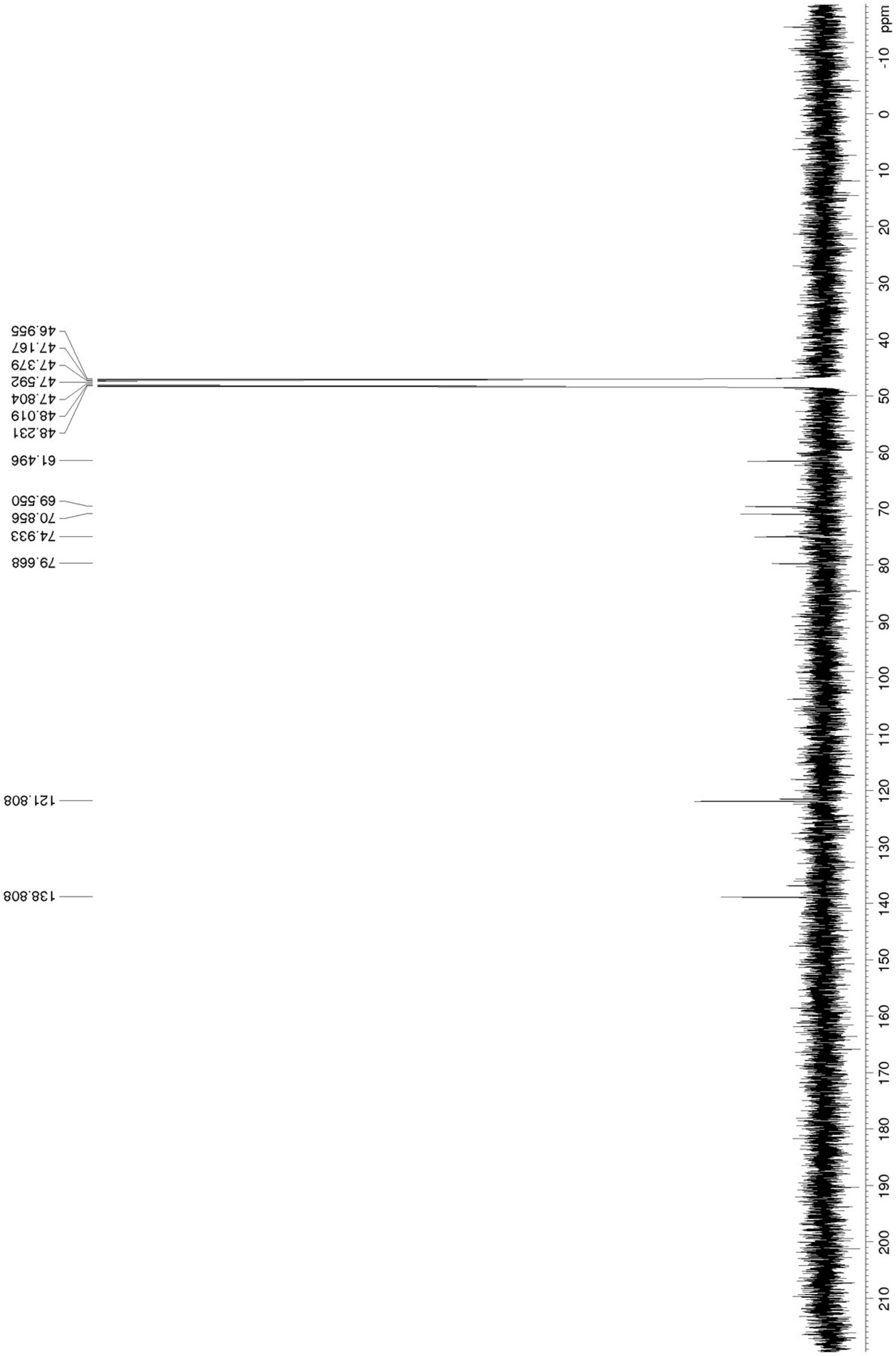
46.955
47.167
47.379
47.592
47.804
48.019
48.231
61.496
69.550
70.856
74.933
79.668
121.808
138.808
ppm

1f ^{1}H-NMR

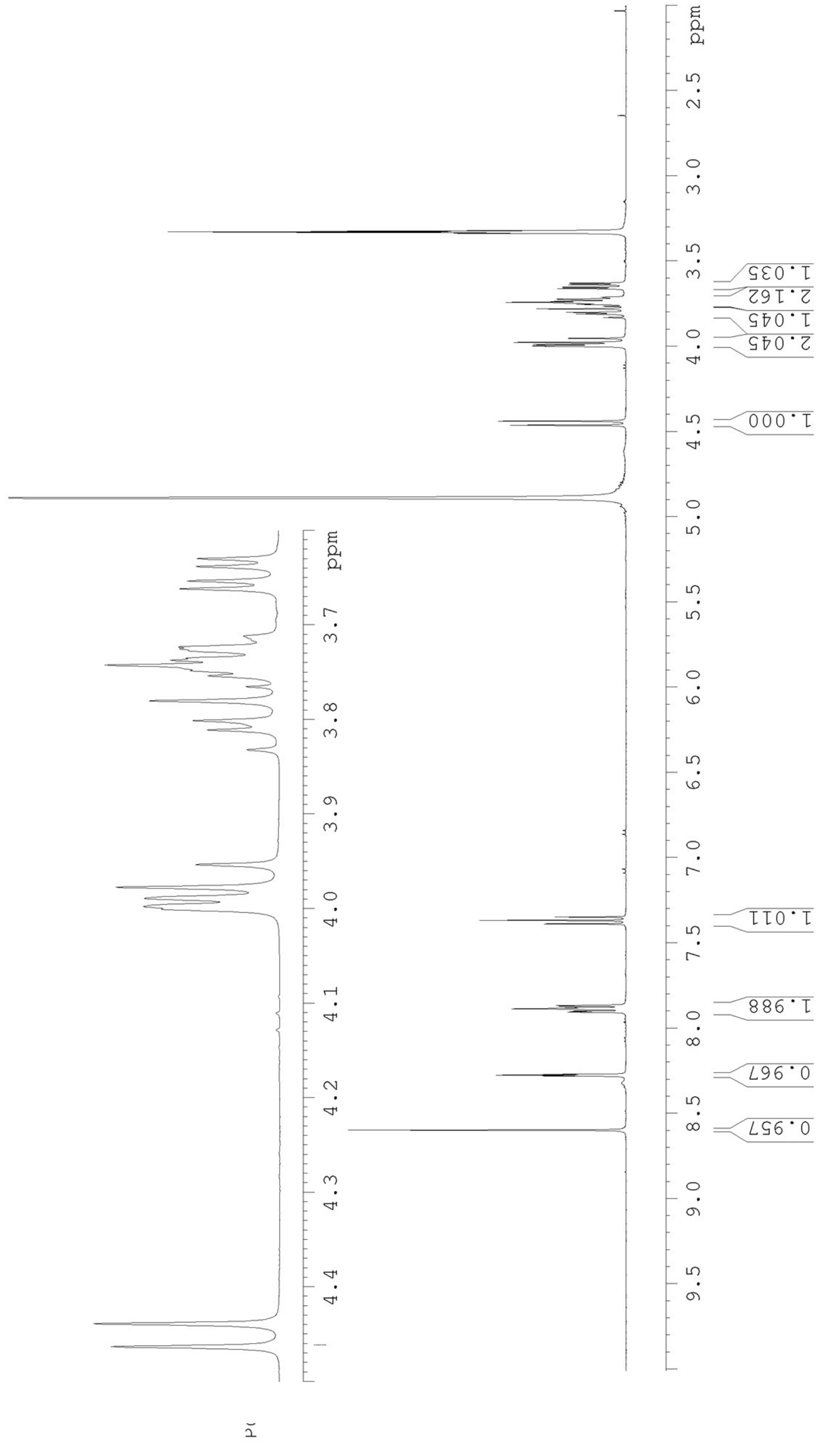

1f ^{13}C-NMR

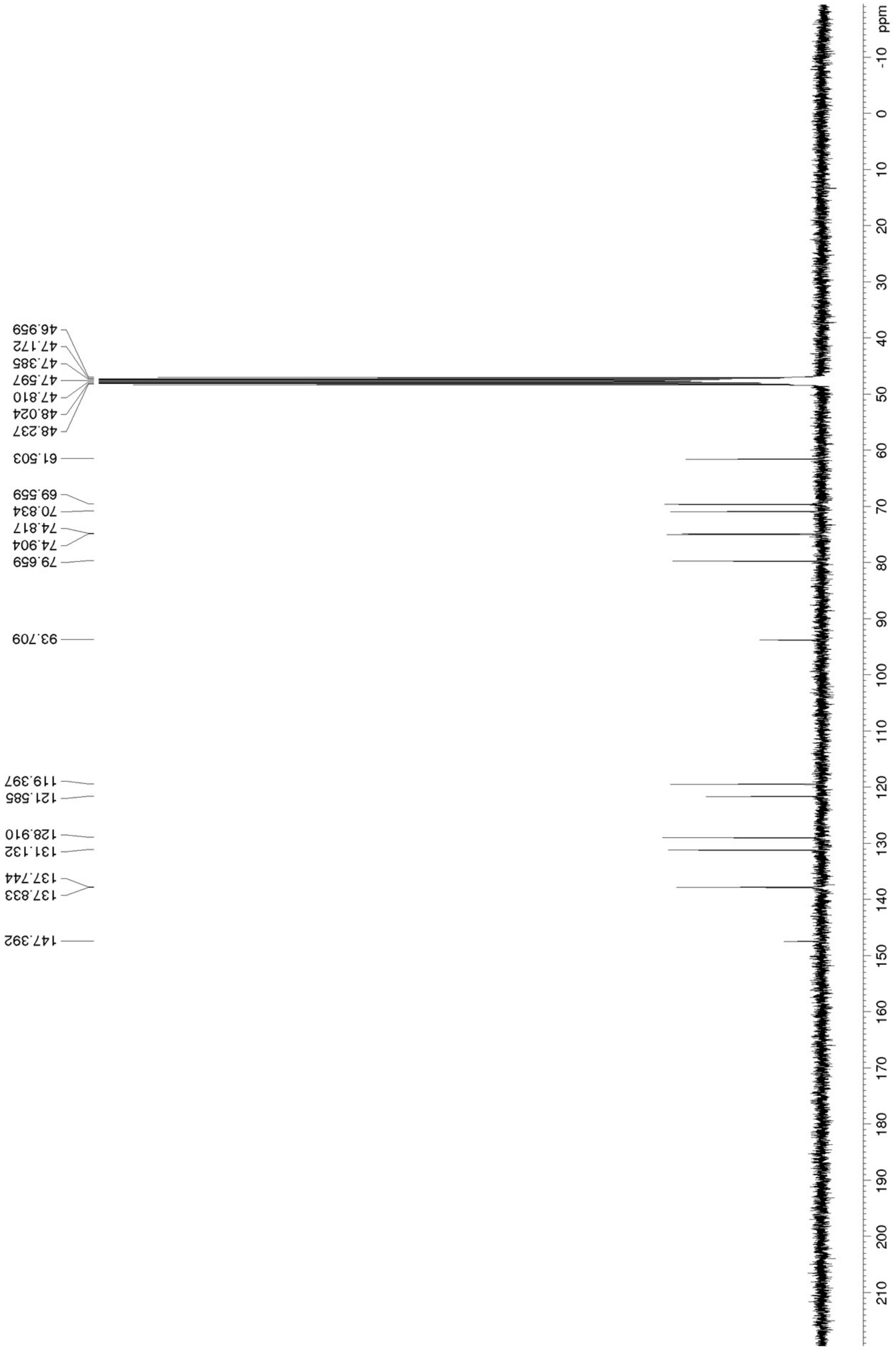
46.959
47.172
47.385
47.597
47.810
48.024
48.237
61.503
69.559
70.834
74.817
74.904
79.659
93.709
119.397
121.585
128.910
131.132
137.744
137.833
147.392
ppm

1g ^{1}H-NMR

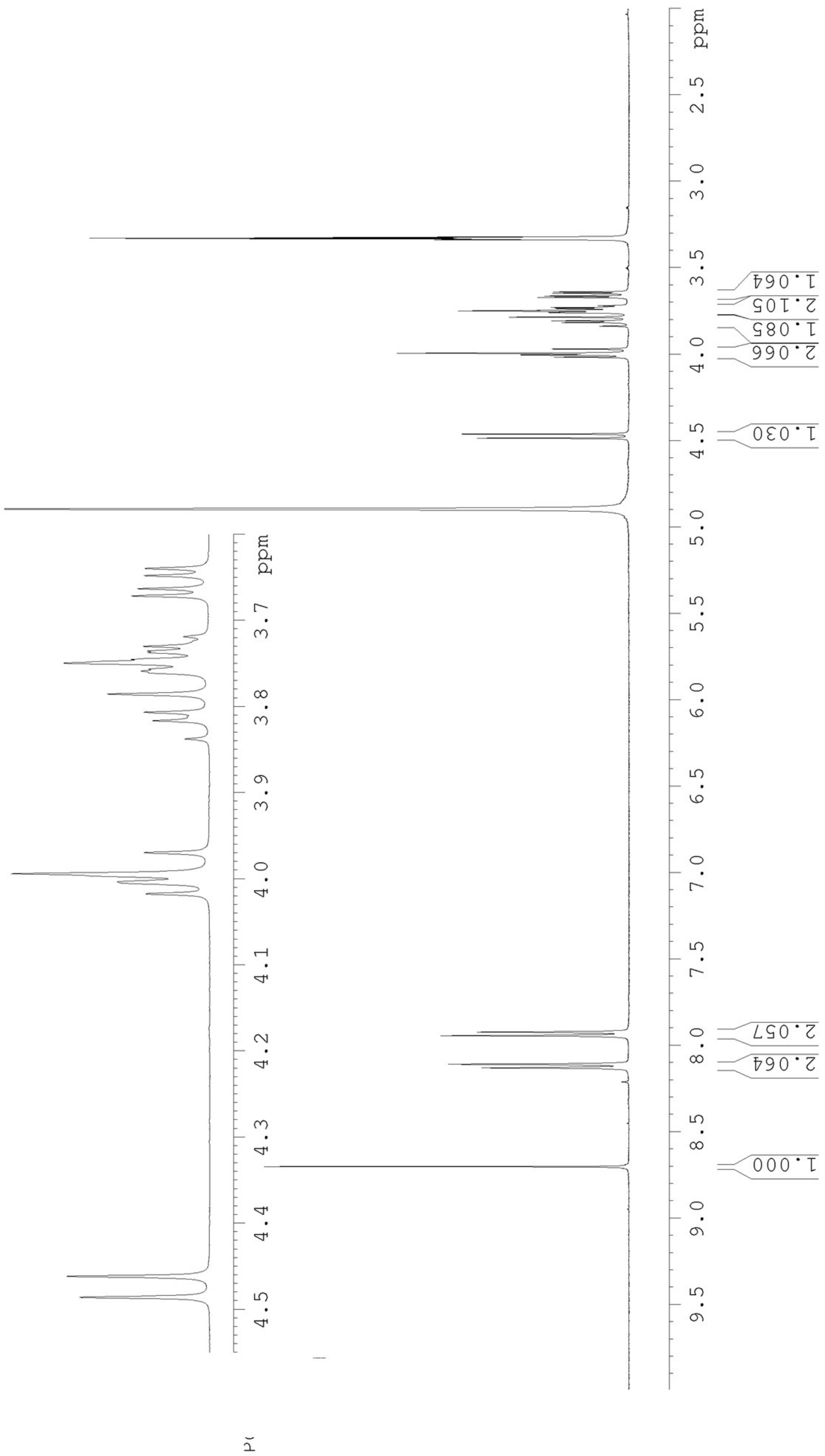

1g ^{13}C-NMR

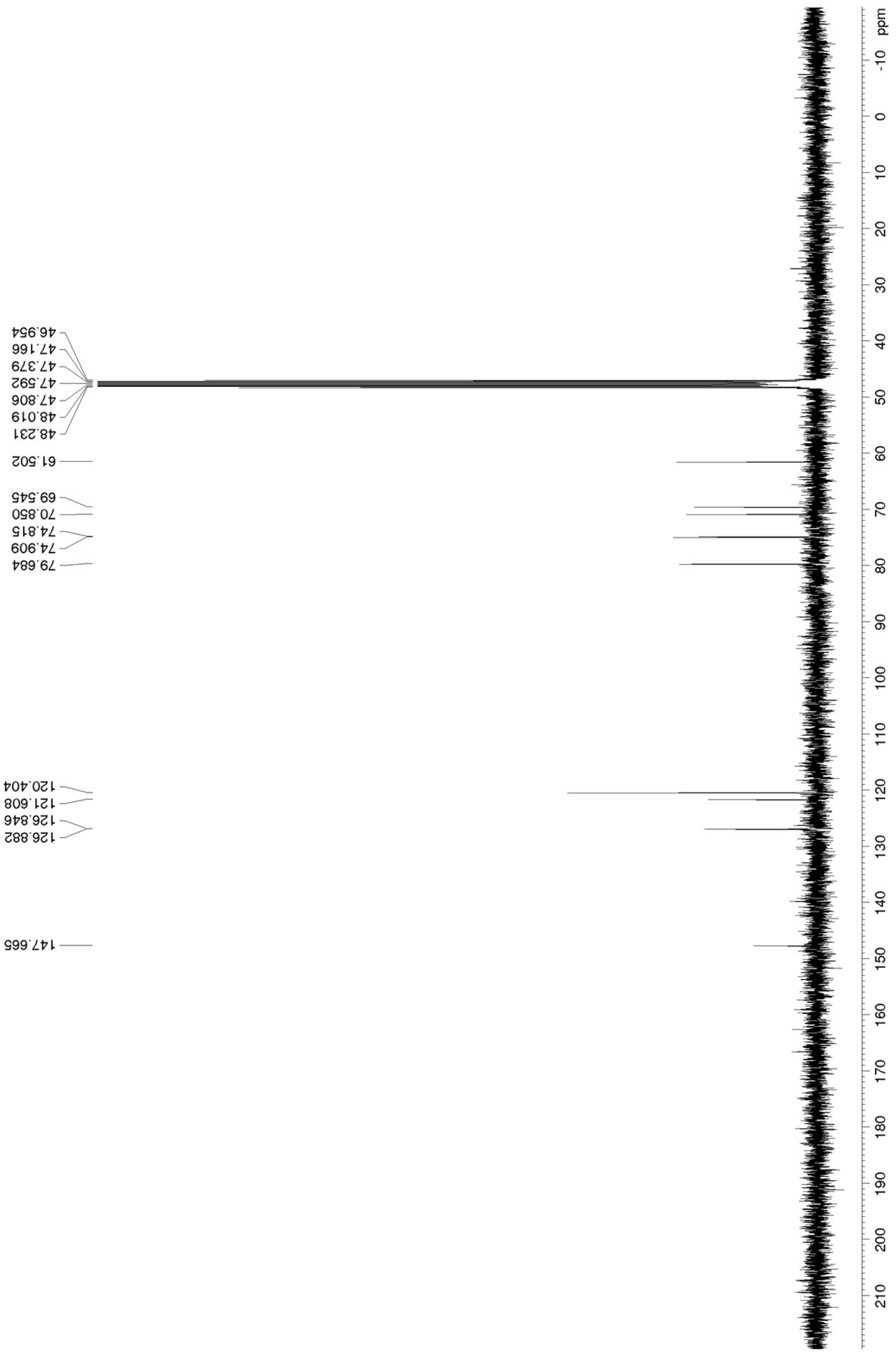
46.954
47.166
47.379
47.592
47.806
48.019
48.231
61.502
69.545
70.850
74.815
74.909
79.684
120.404
121.608
126.846
126.882
147.665
ppm

1h ^{1}H-NMR

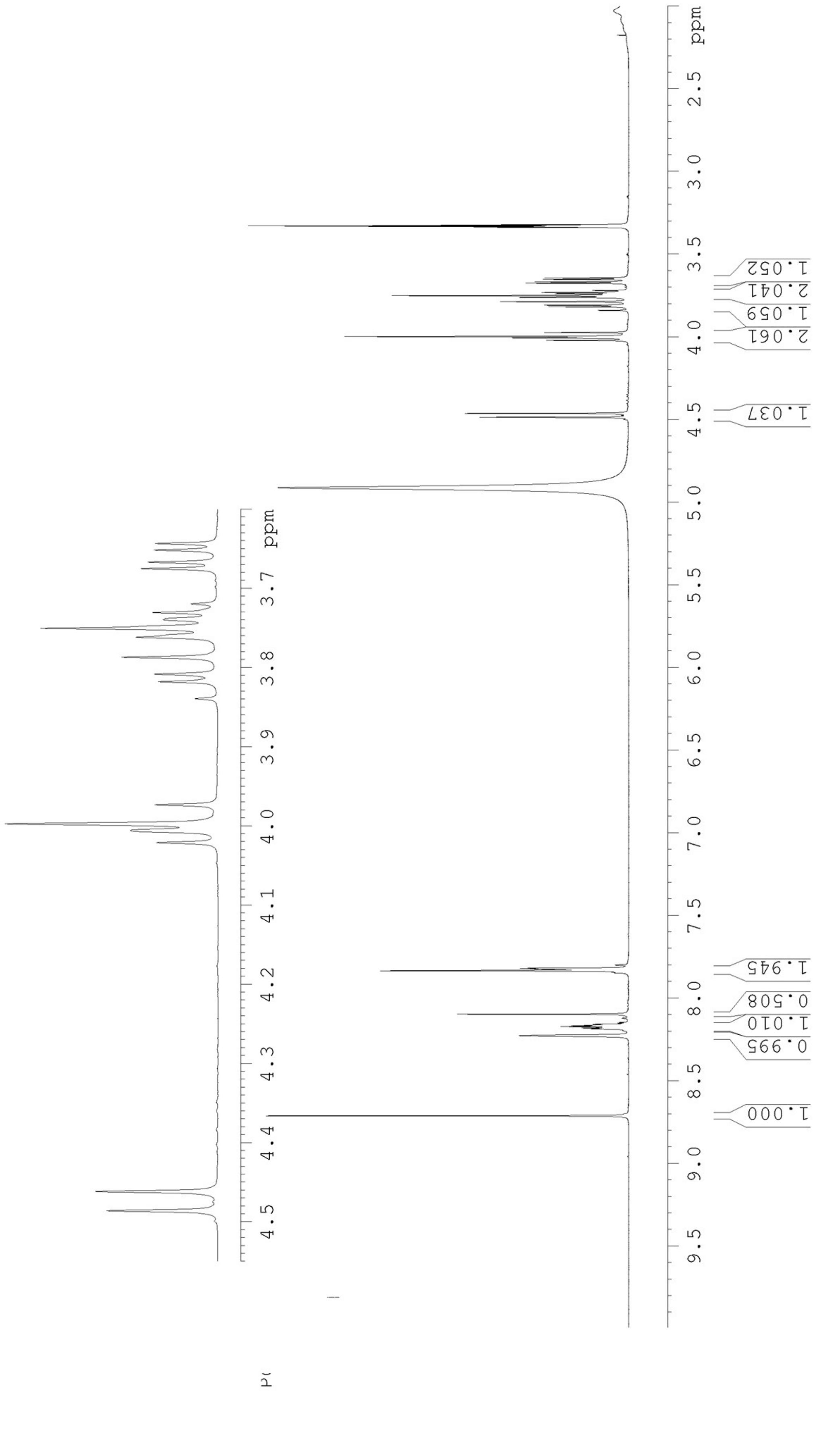

1h ^{13}C-NMR

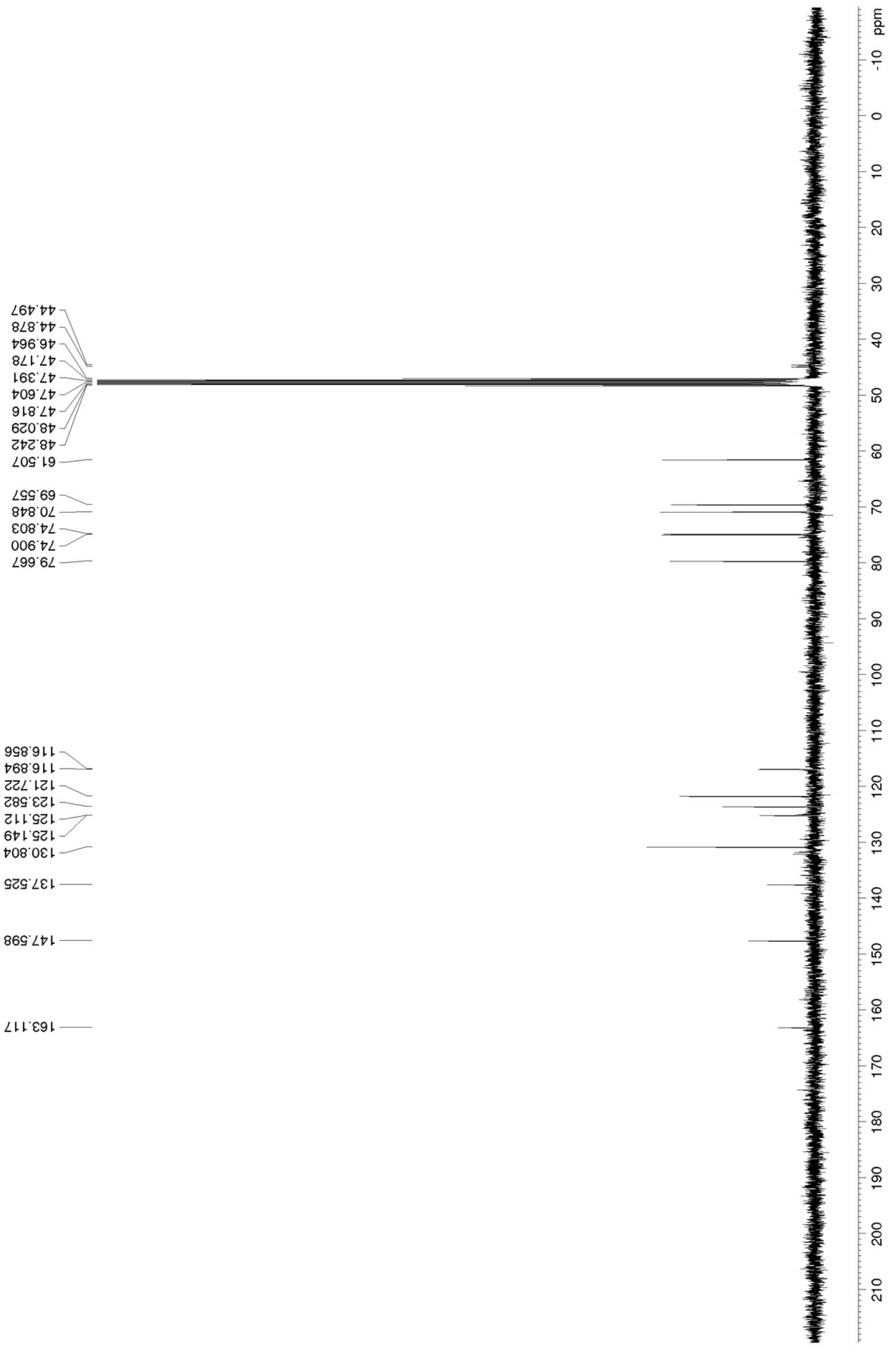
44.497
44.878
46.964
47.178
47.391
47.604
47.816
48.029
48.242
61.507
69.557
70.848
74.803
74.900
79.667
116.856
116.894
121.722
123.582
125.112
125.149
130.804
137.525
147.598
163.117
ppm

1i ^{1}H-NMR

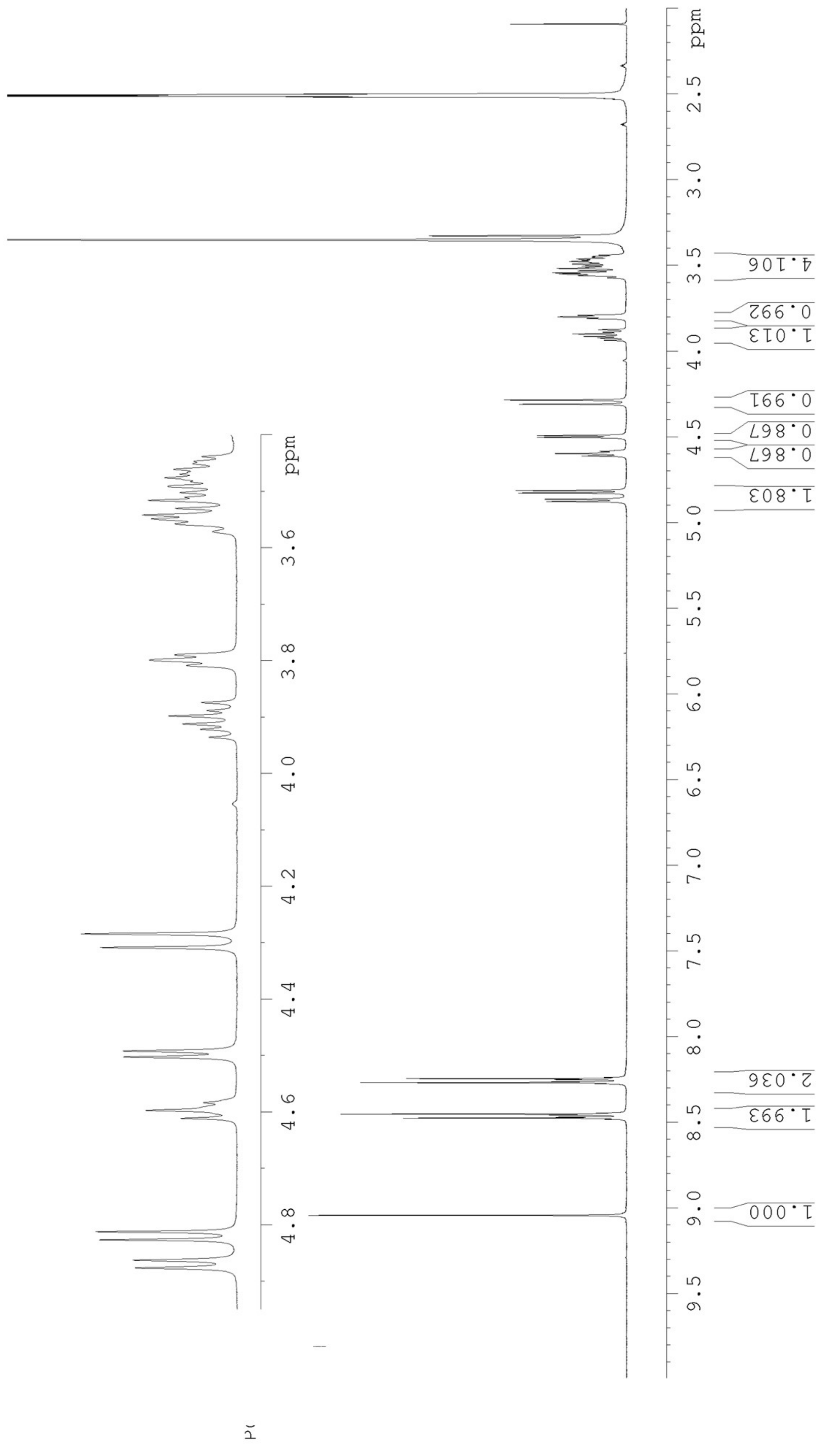
ppm
2.5
3.0
3.5
4.0
4.5
5.0
5.5
6.0
6.5
7.0
7.5
8.0
8.5
9.0
9.5
4.106
0.992
1.013
0.991
0.867
0.867
1.803
2.036
1.993
1.000
ppm
3.6
3.8
4.0
4.2
4.4
4.6
4.8

1i ^{13}C-NMR

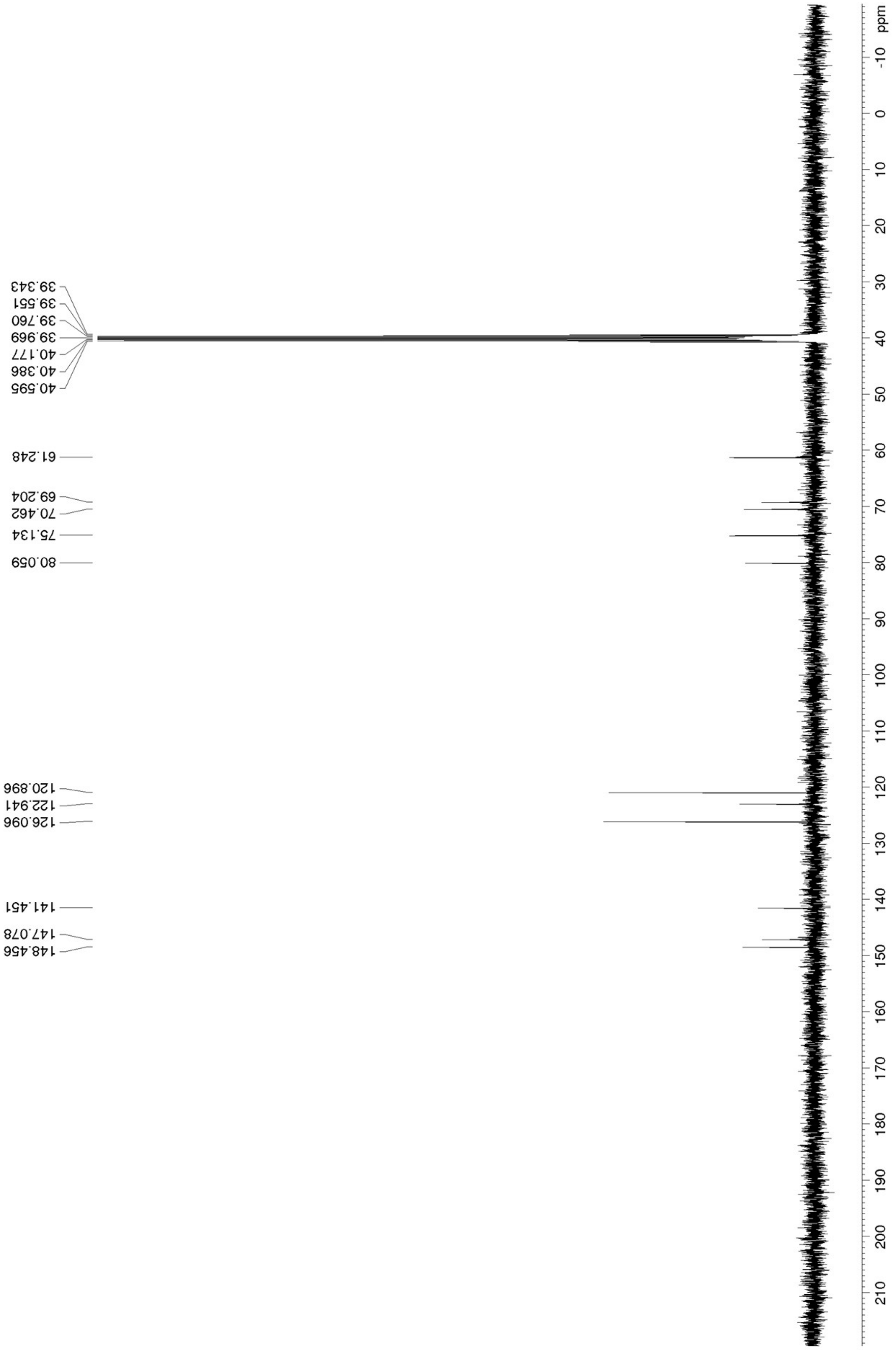
39.343
39.551
39.760
39.969
40.177
40.386
40.595
61.248
69.204
70.462
75.134
80.059
120.896
122.941
126.096
141.451
147.078
148.456
ppm

1j ^{1}H-NMR

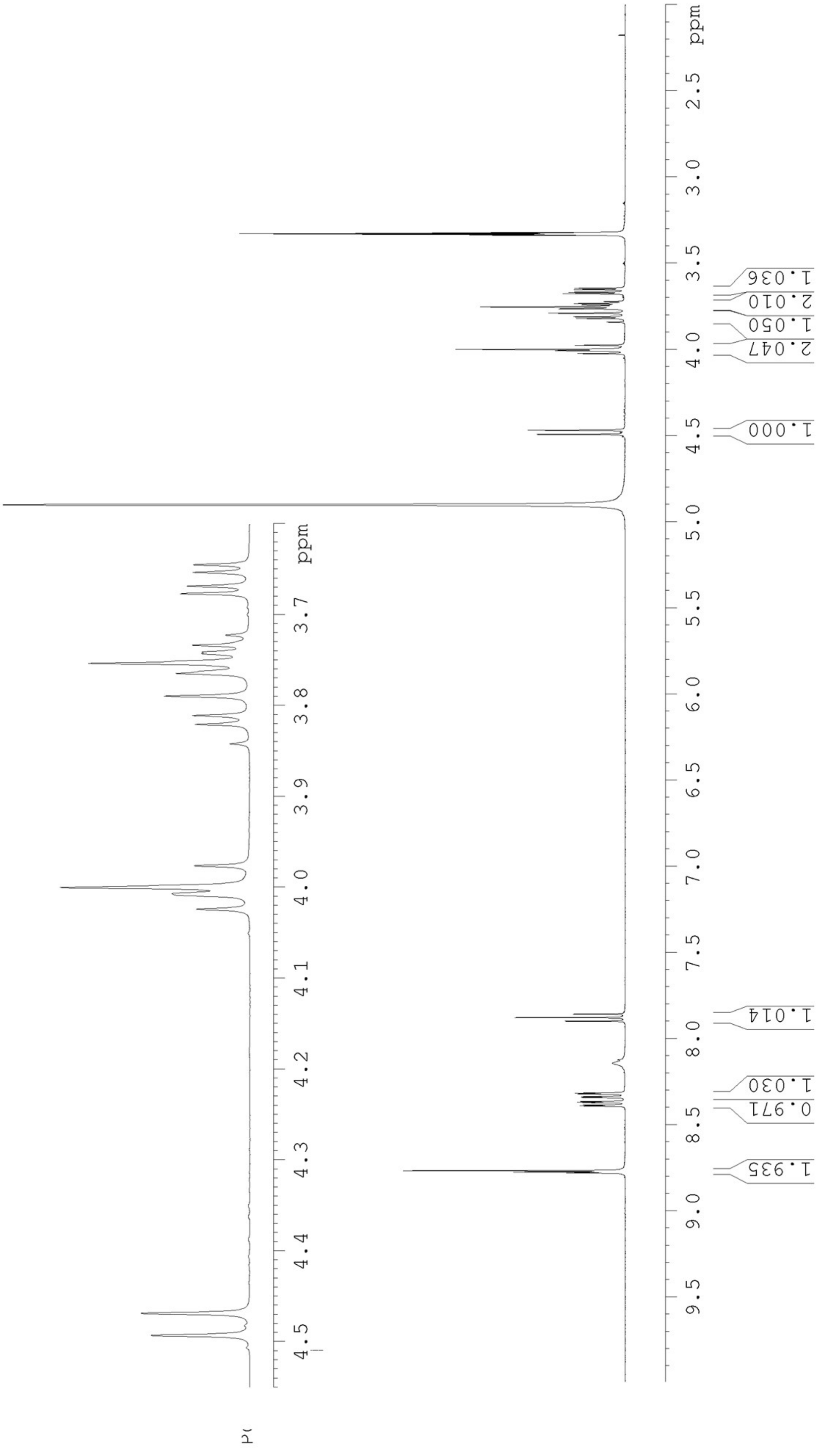
ppm
2.5
3.0
3.5
4.0
4.5
5.0
5.5
6.0
6.5
7.0
7.5
8.0
8.5
9.0
9.5
1.036
2.010
1.050
2.047
1.000
1.014
1.030
0.971
1.935
ppm
3.7
3.8
3.9
4.0
4.1
4.2
4.3
4.4
4.5

1j ^{13}C-NMR

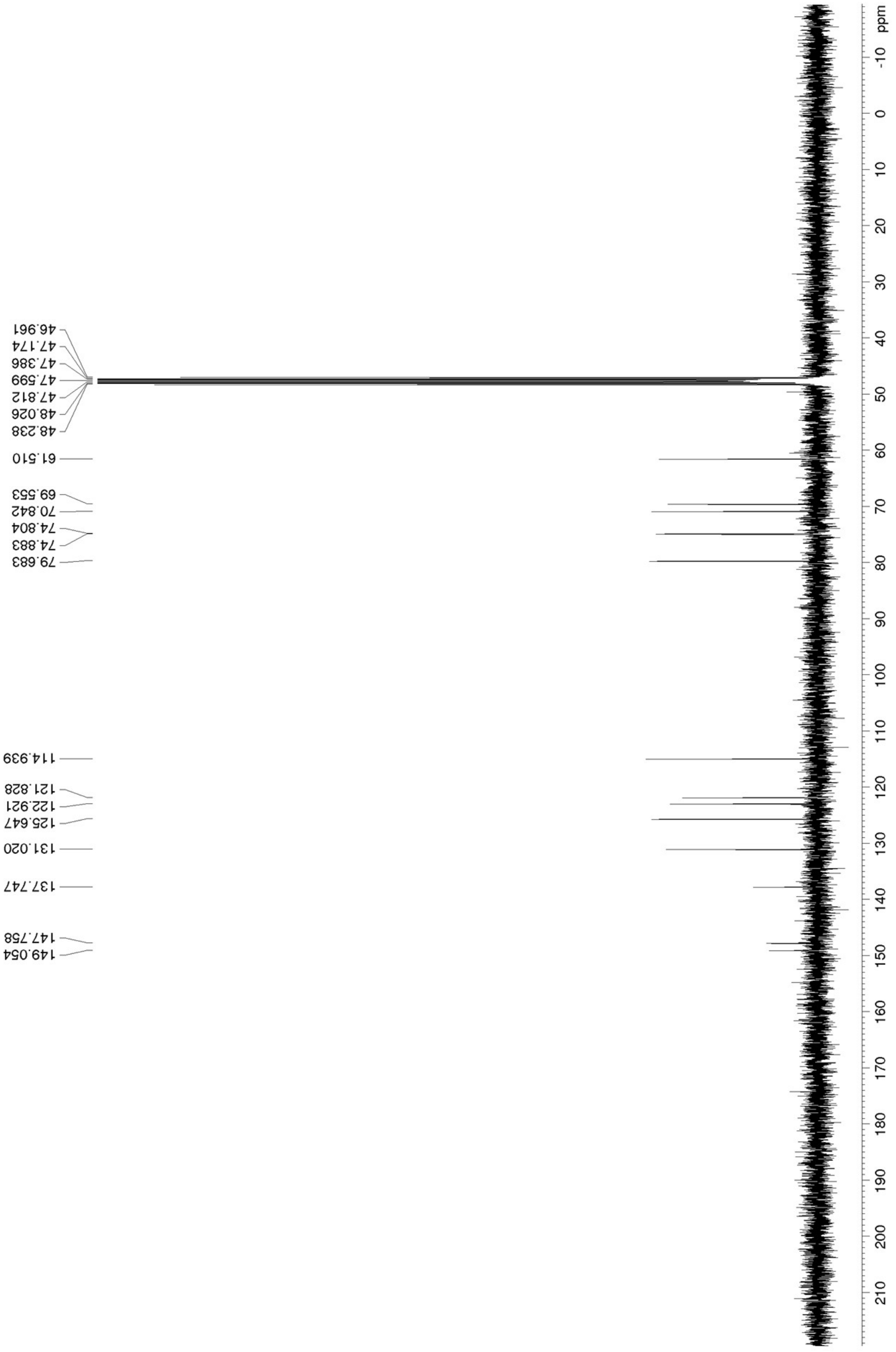
46.961
47.174
47.386
47.599
47.812
48.026
48.238
61.510
69.553
70.842
74.804
74.883
79.683
114.939
121.828
122.921
125.647
131.020
137.747
147.758
149.054
ppm

1k ^{1}H-NMR

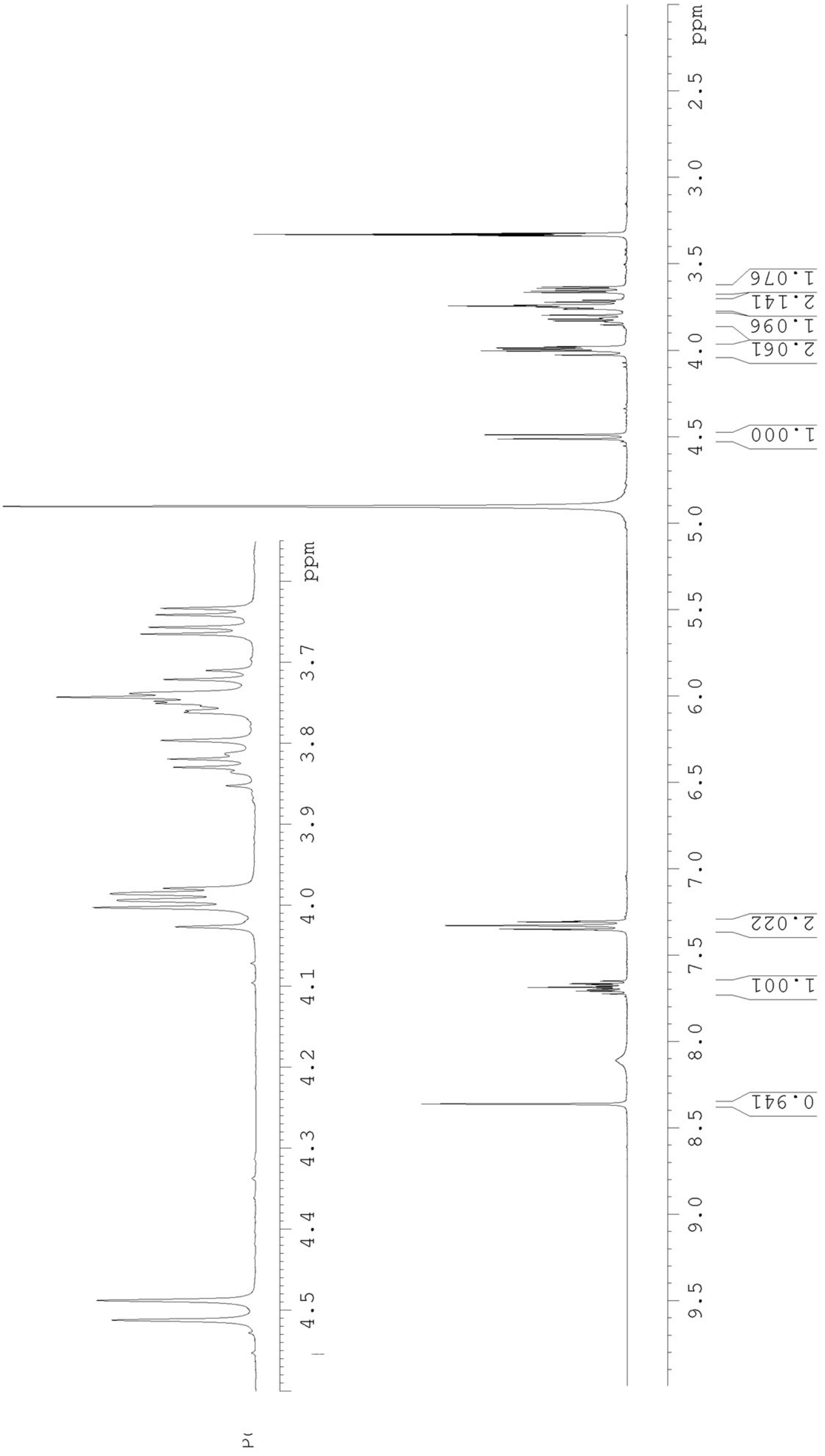

1k ^{13}C-NMR

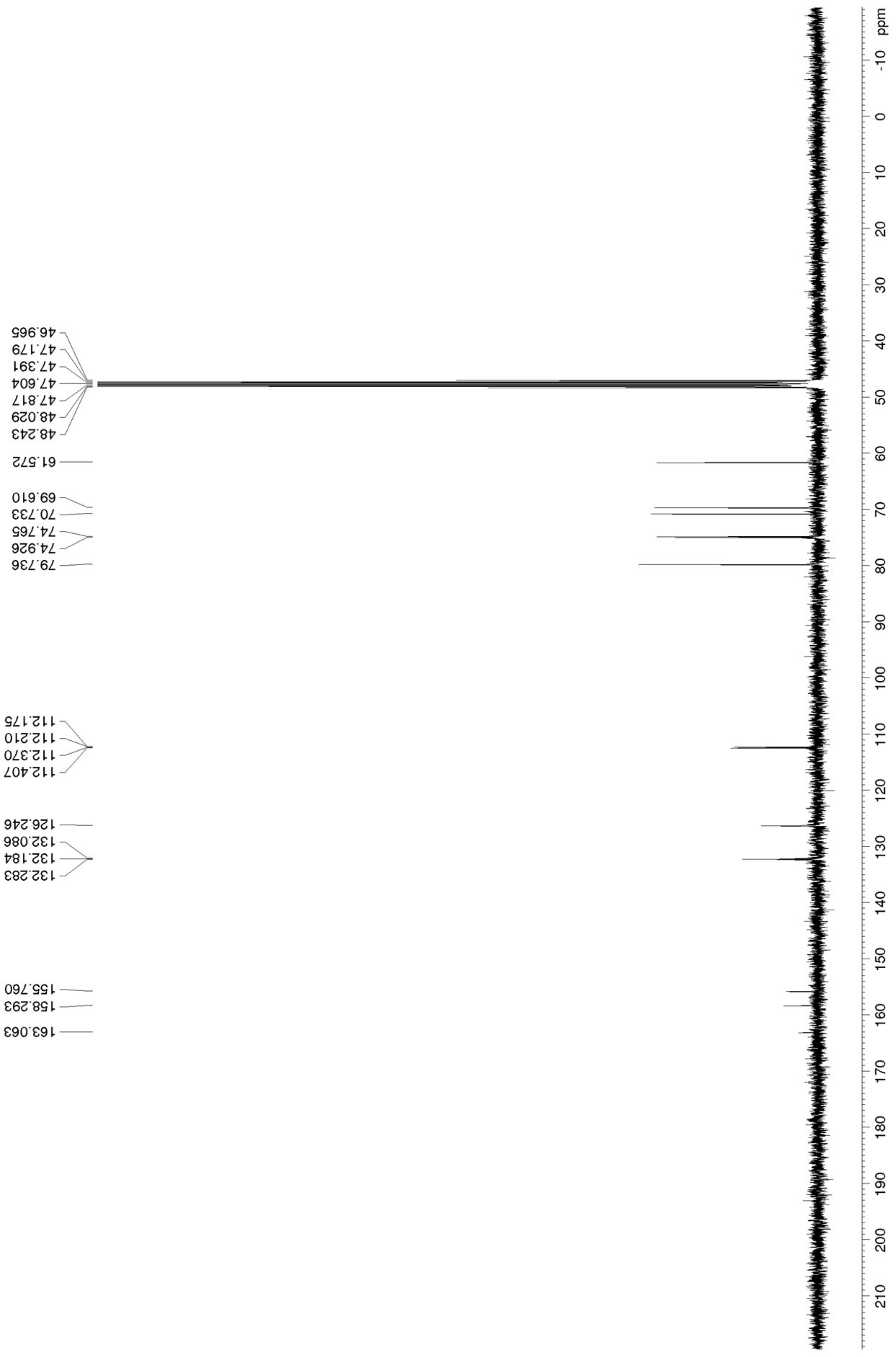
46.965
47.179
47.391
47.604
47.817
48.029
48.243
61.572
69.610
70.733
74.765
74.926
79.736
112.175
112.210
112.370
112.407
126.246
132.086
132.184
132.283
155.760
158.293
163.063
ppm

1l ^{1}H-NMR

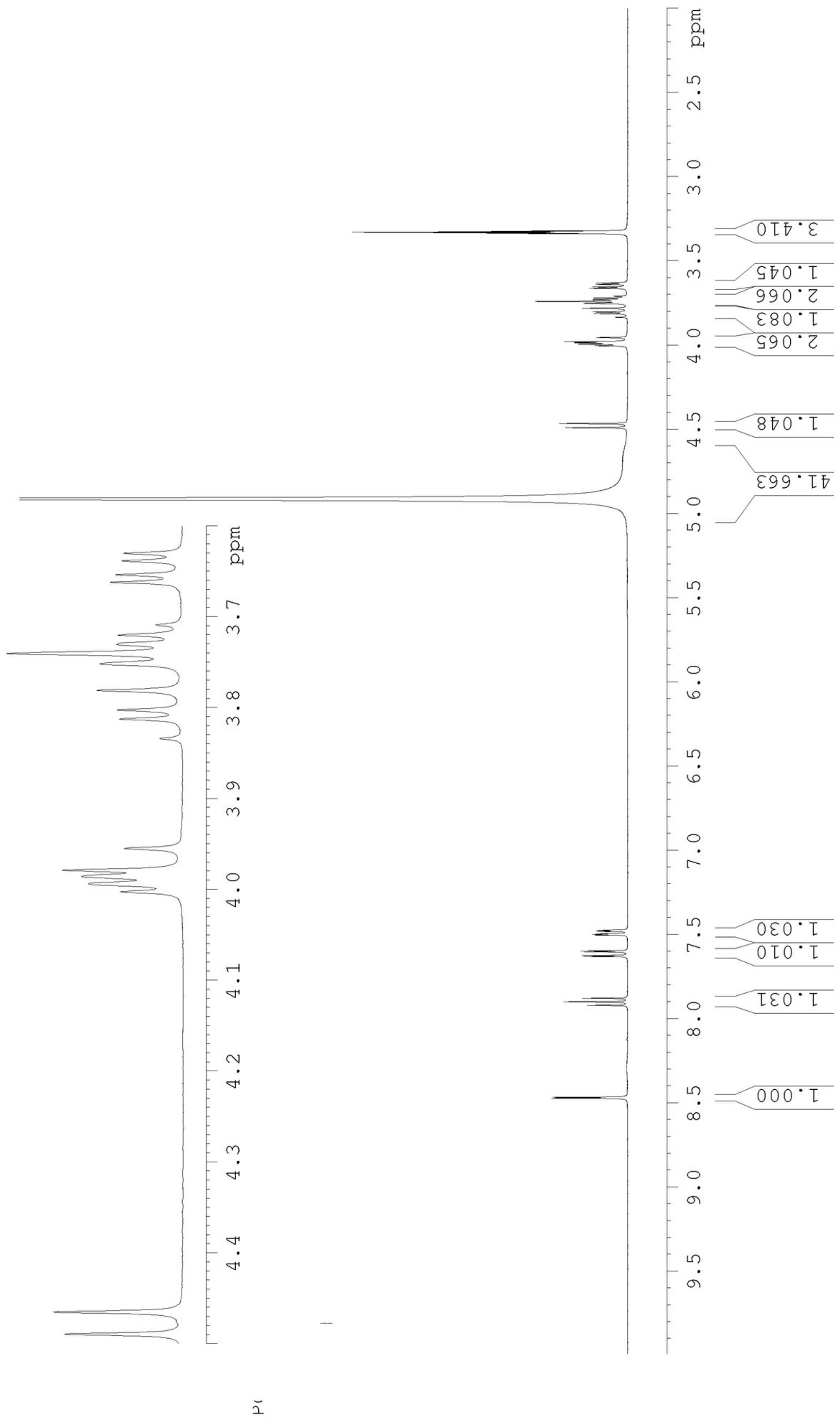

1l ^{13}C-NMR

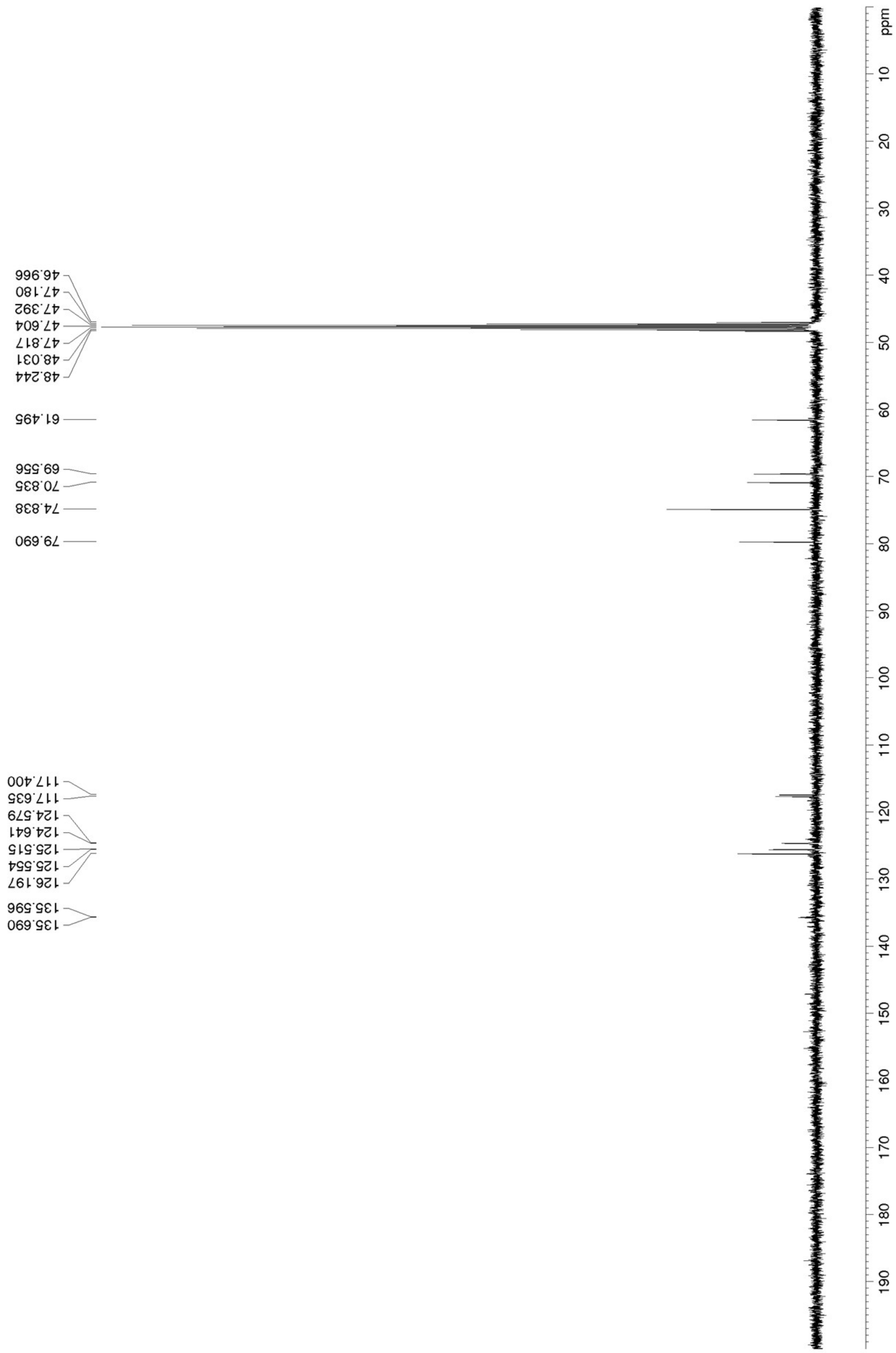
46.966
47.180
47.392
47.604
47.817
48.031
48.244
61.495
69.556
70.835
74.838
79.690
117.400
117.635
124.579
124.641
125.515
125.554
126.197
135.596
135.690
ppm

1m ^{1}H-NMR

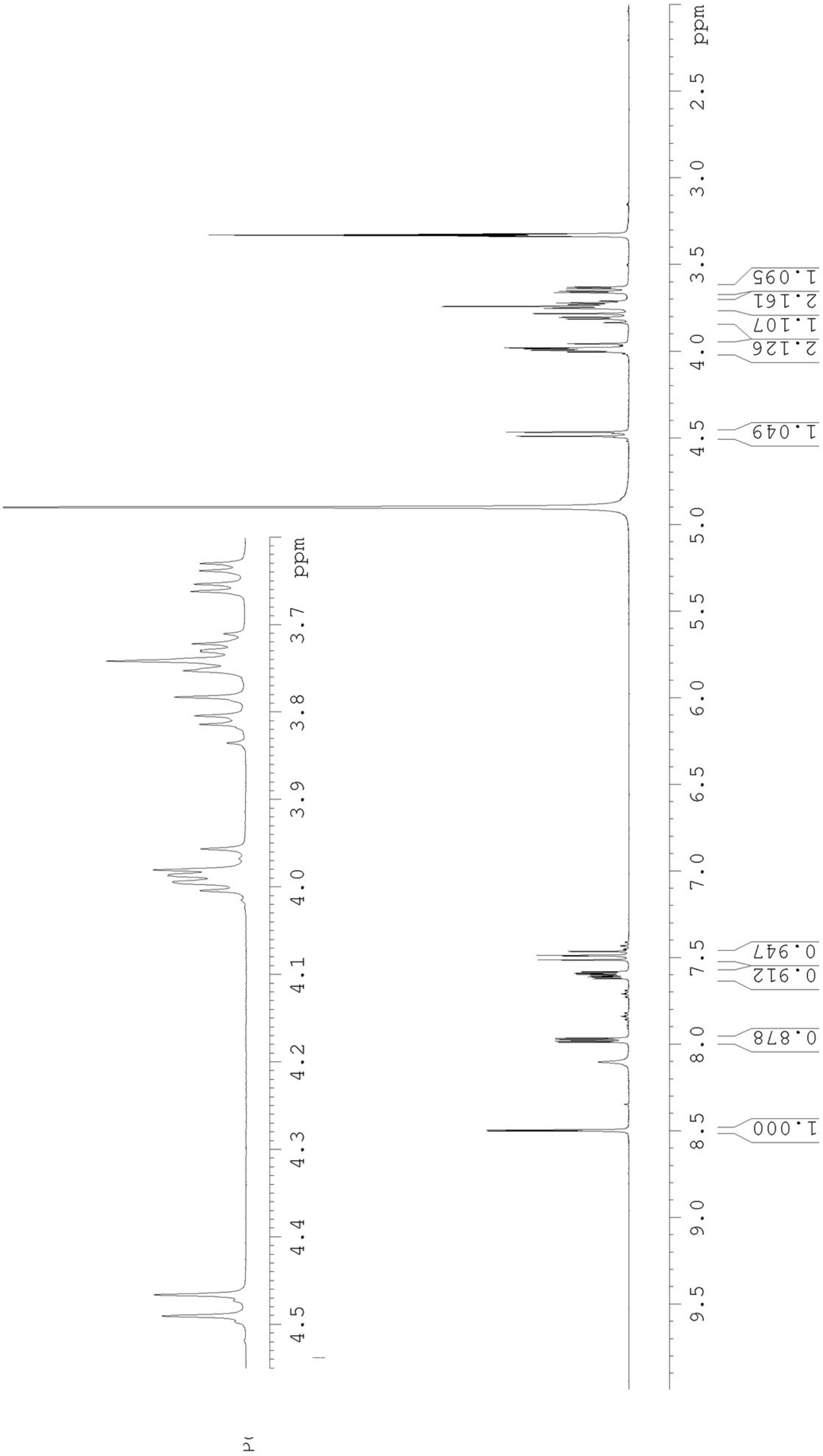

1m ^{13}C-NMR

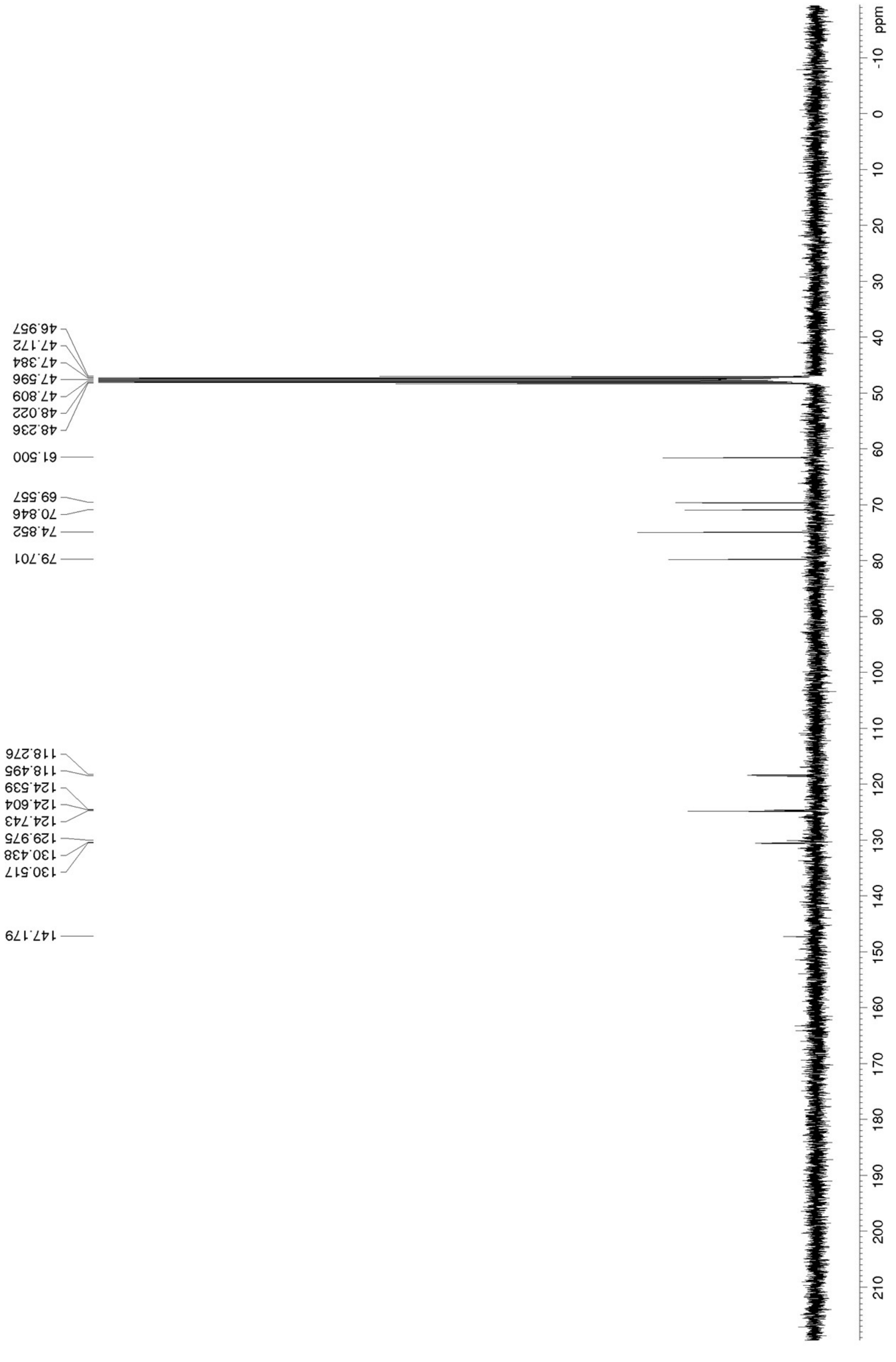
46.957
47.172
47.384
47.596
47.809
48.022
48.236
61.500
69.557
70.846
74.852
79.701
118.276
118.495
124.539
124.604
124.743
129.975
130.438
130.517
147.179
ppm

1n ^{1}H-NMR

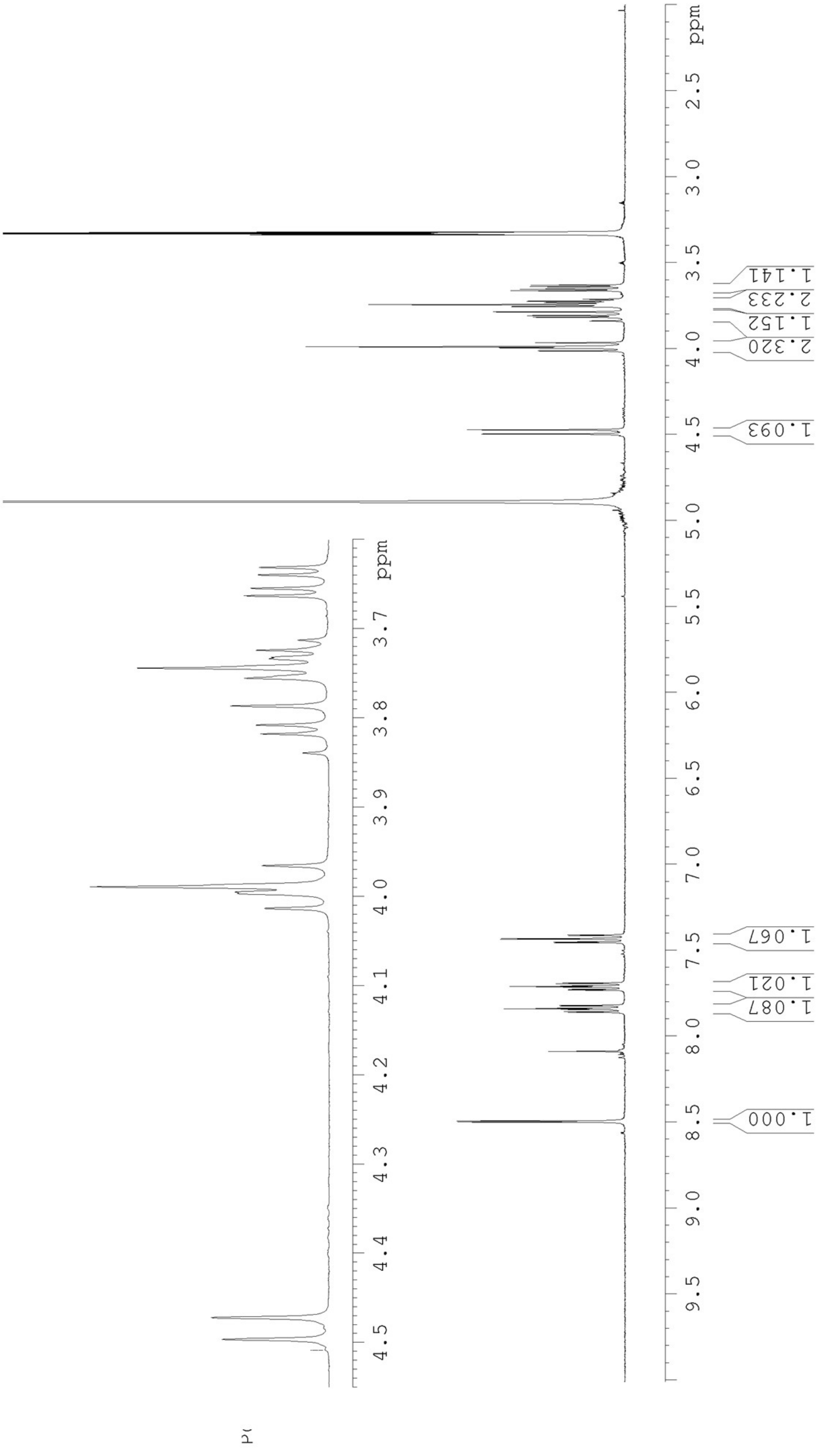

1n ^{13}C-NMR

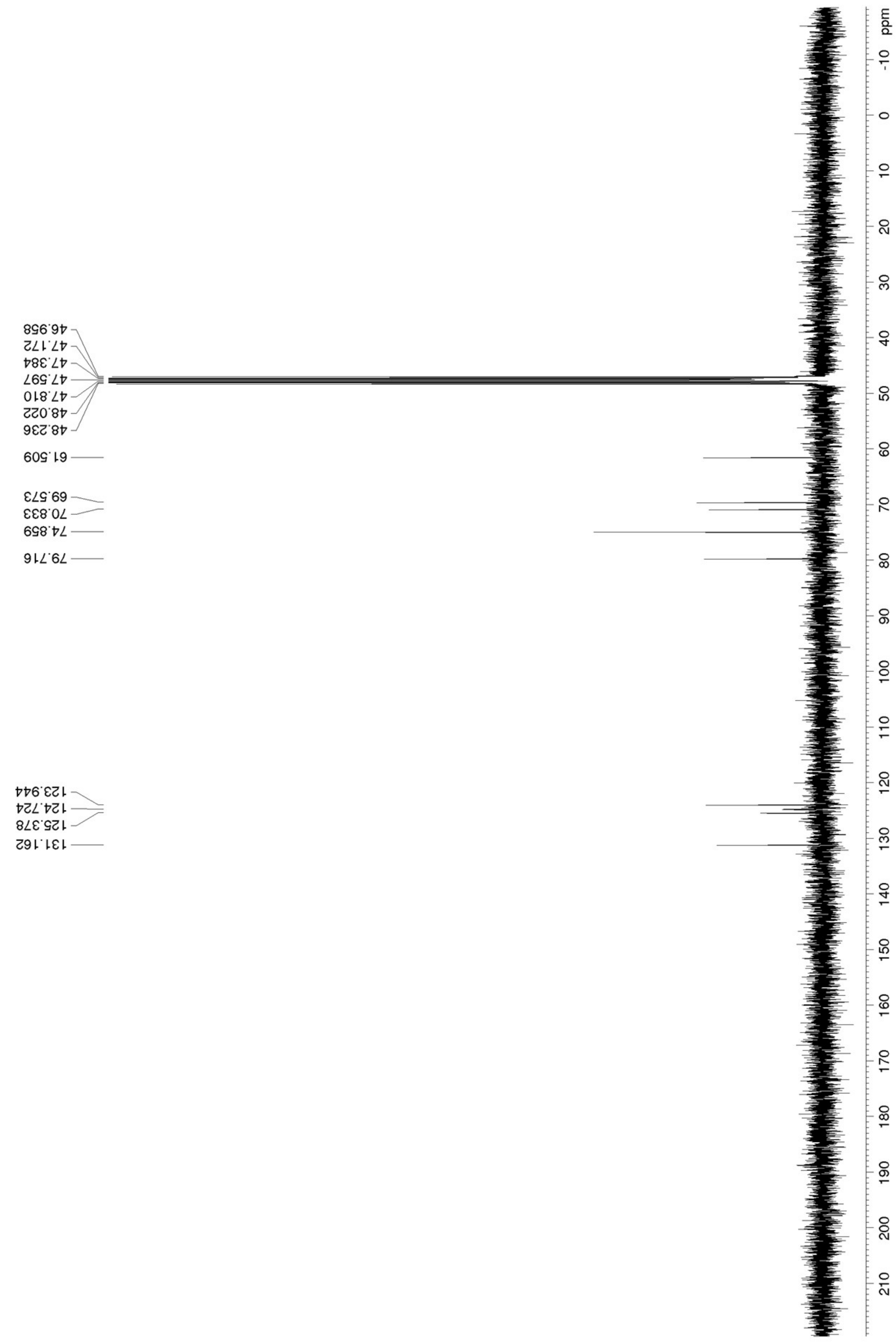
46.958
47.172
47.384
47.597
47.810
48.022
48.236
61.509
69.573
70.833
74.859
79.716
123.944
124.724
125.378
131.162
ppm

1o ^{1}H-NMR

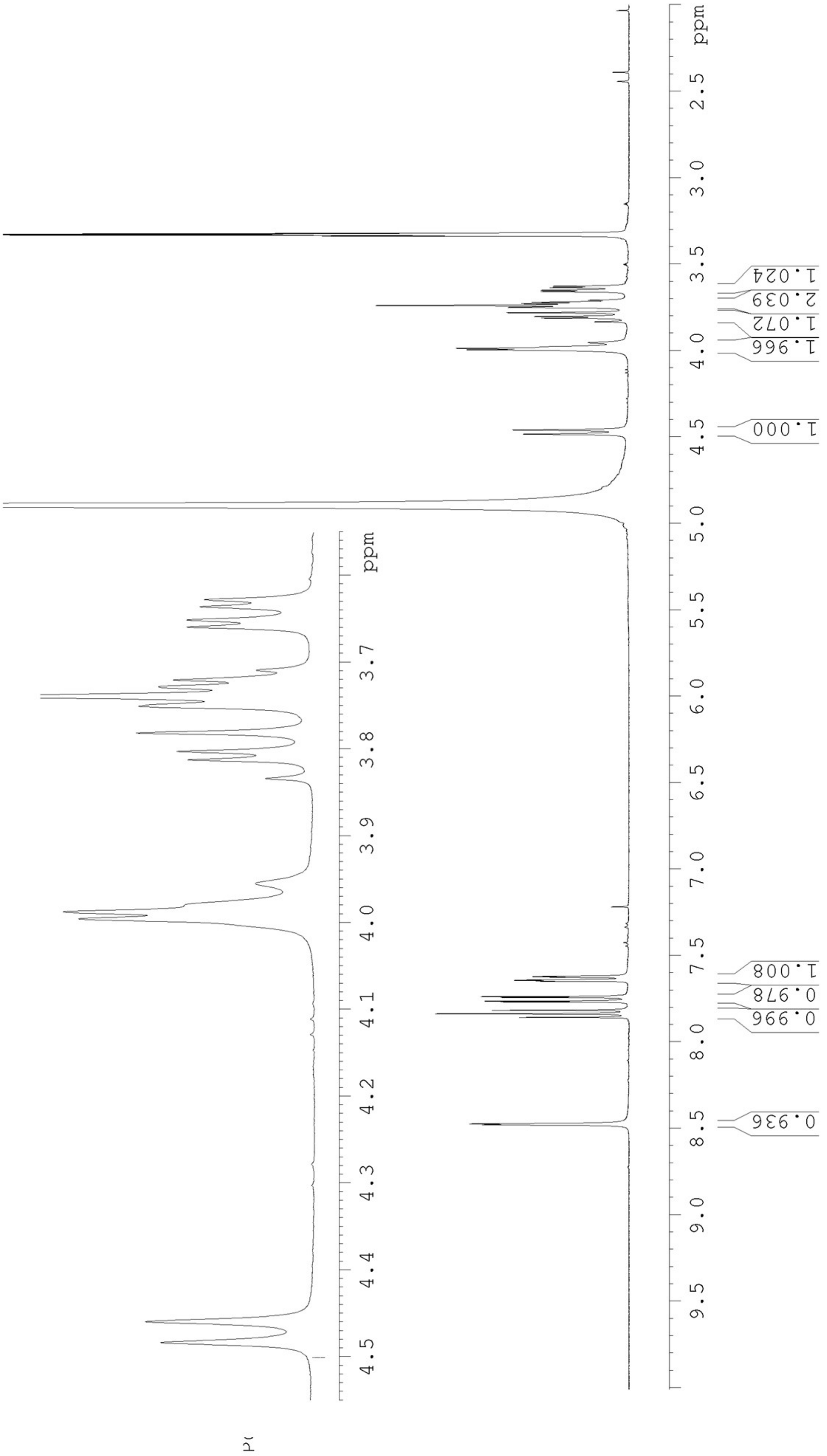
ppm
2.5
3.0
3.5
4.0
4.5
5.0
5.5
6.0
6.5
7.0
7.5
8.0
8.5
9.0
9.5
1.024
2.039
1.072
1.966
1.000
1.008
0.978
0.996
0.936
ppm
3.7
3.8
3.9
4.0
4.1
4.2
4.3
4.4
4.5

1o ^{13}C-NMR

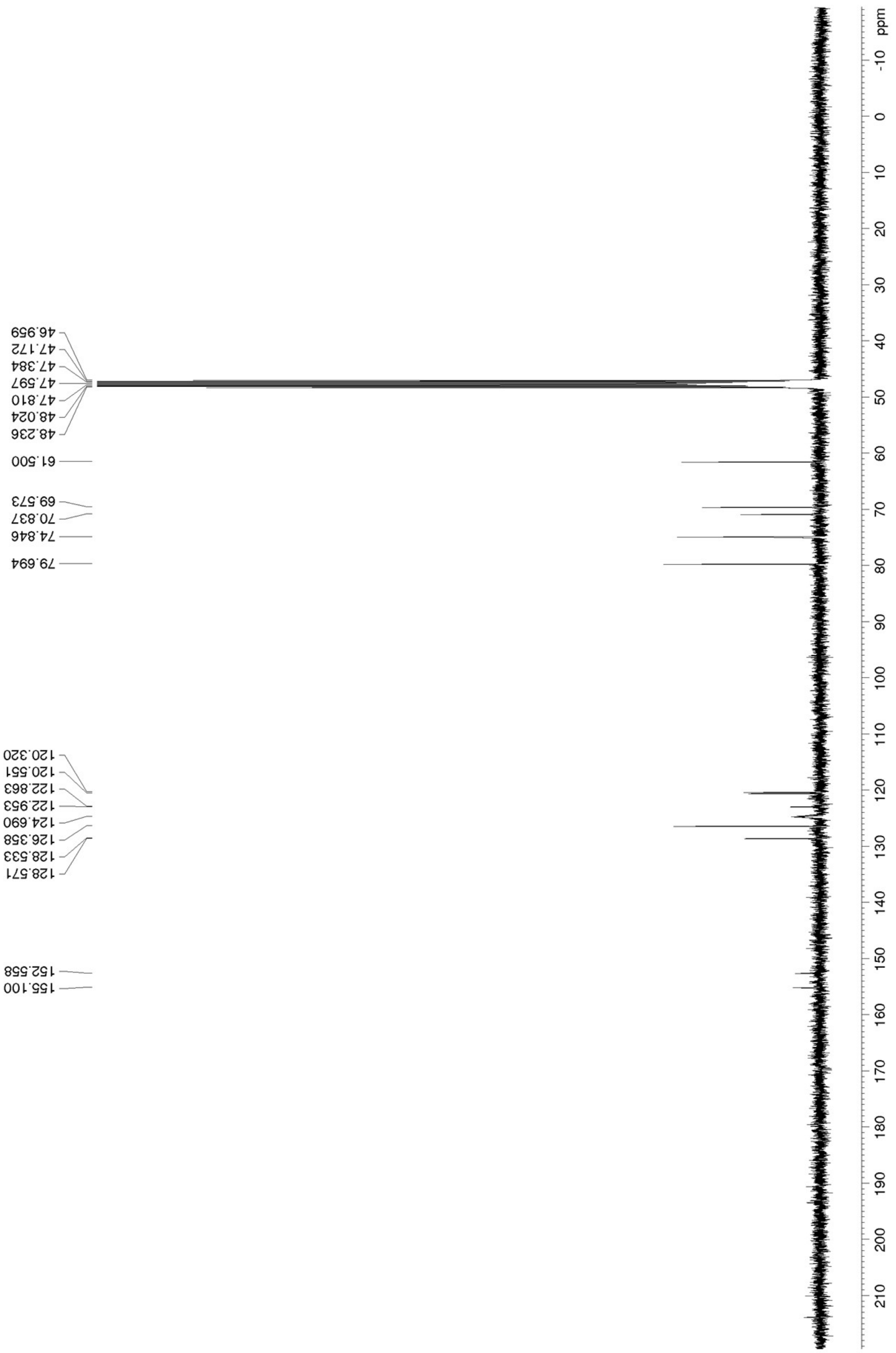
46.959
47.172
47.384
47.597
47.810
48.024
48.236
61.500
69.573
70.837
74.846
79.694
120.320
120.551
122.863
122.953
124.690
126.358
128.533
128.571
152.558
155.100
ppm

1p ^{1}H-NMR

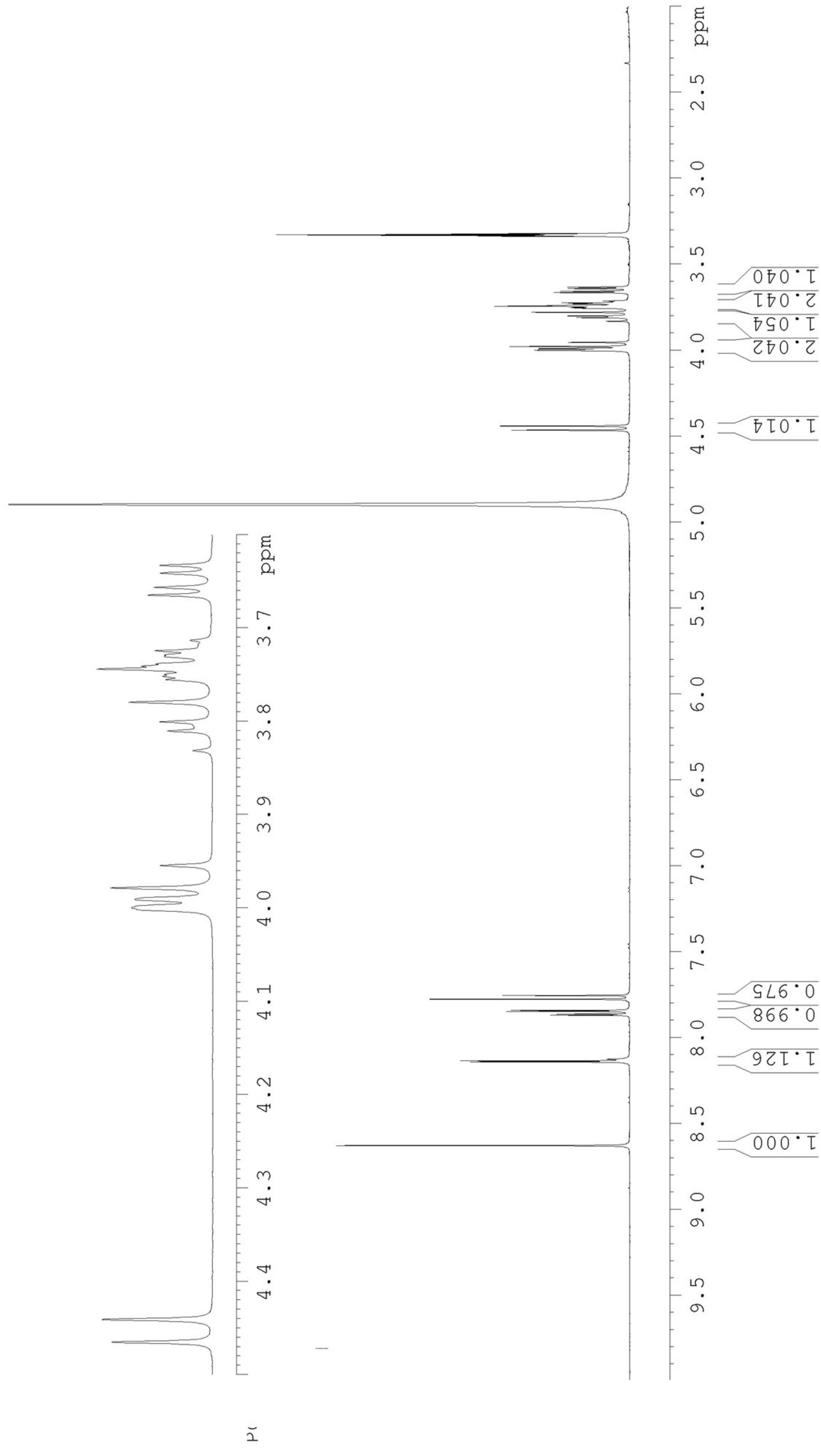
ppm
2.5
3.0
3.5
4.0
4.5
5.0
5.5
6.0
6.5
7.0
7.5
8.0
8.5
9.0
9.5
1.040
2.041
1.054
2.042
1.014
0.975
0.998
1.126
1.000
ppm
3.7
3.8
3.9
4.0
4.1
4.2
4.3
4.4

1p ^{13}C-NMR

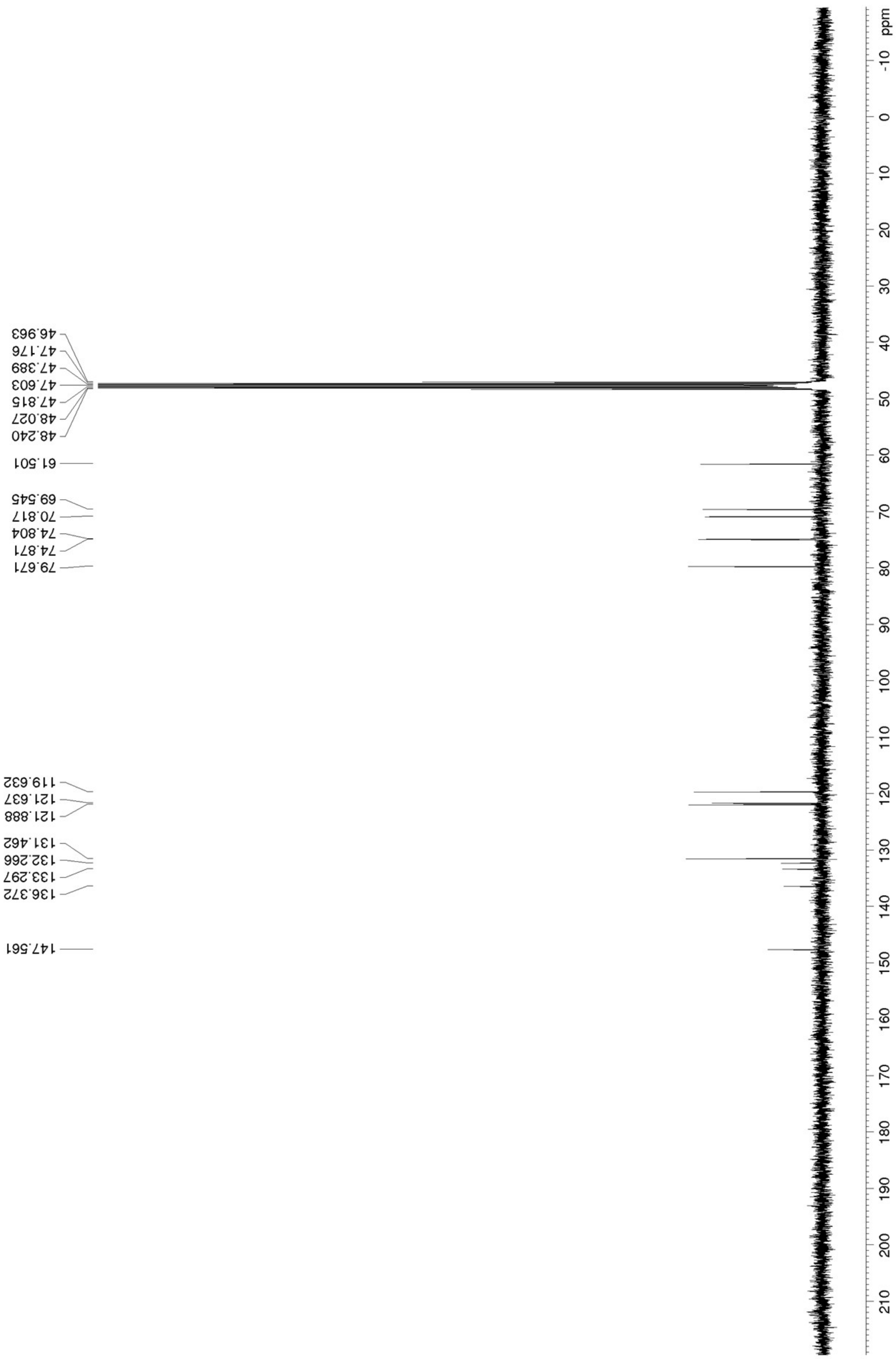
46.963
47.176
47.389
47.603
47.815
48.027
48.240
61.501
69.545
70.817
74.804
74.871
79.671
119.632
121.637
121.888
131.462
132.266
133.297
136.372
147.561
ppm

1q ^{1}H-NMR

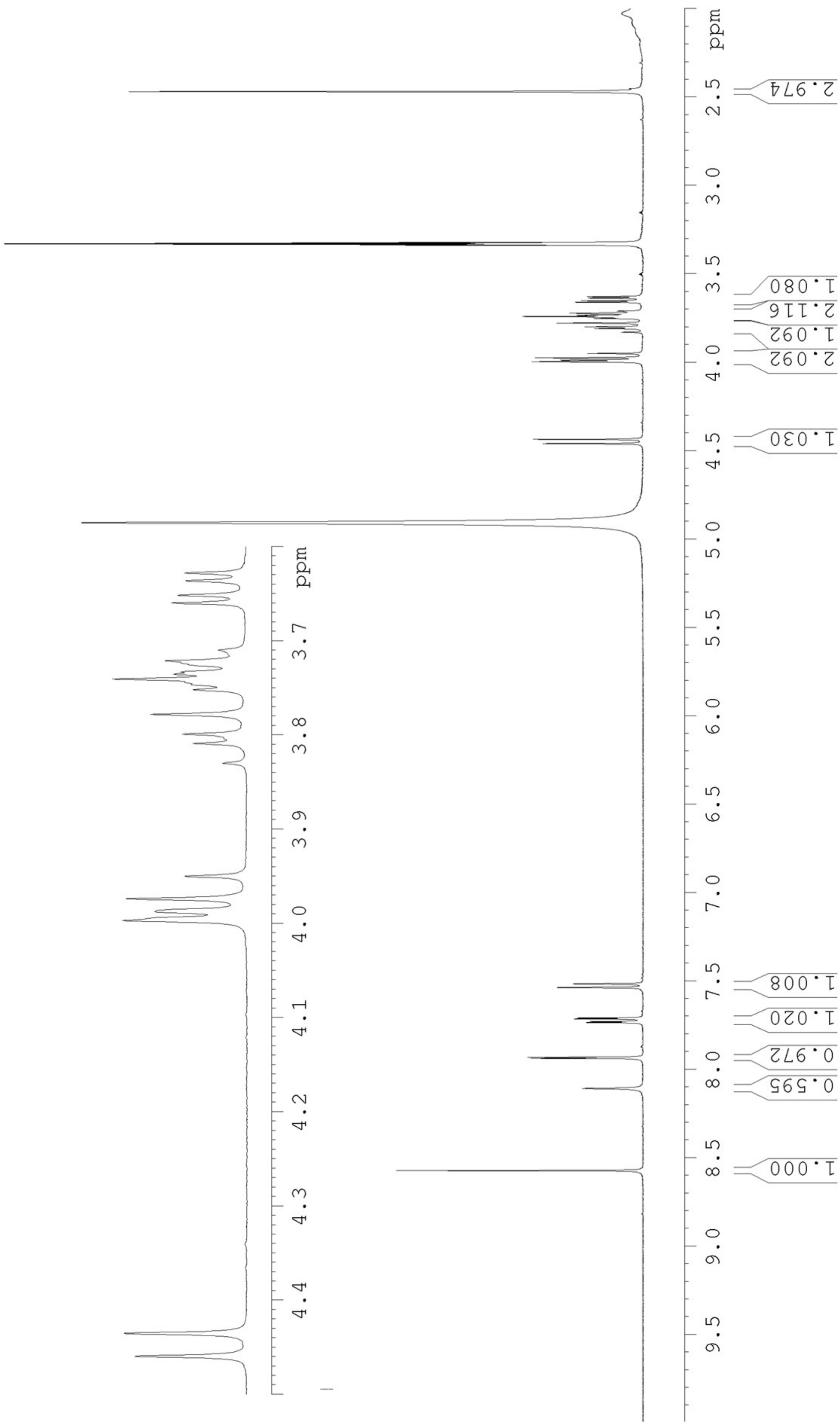
ppm
2.5
3.0
3.5
4.0
4.5
5.0
5.5
6.0
6.5
7.0
7.5
8.0
8.5
9.0
9.5
2.974
1.080
2.116
1.092
2.092
1.030
1.008
1.020
0.972
0.595
1.000
ppm
3.7
3.8
3.9
4.0
4.1
4.2
4.3
4.4

1q ^{13}C-NMR

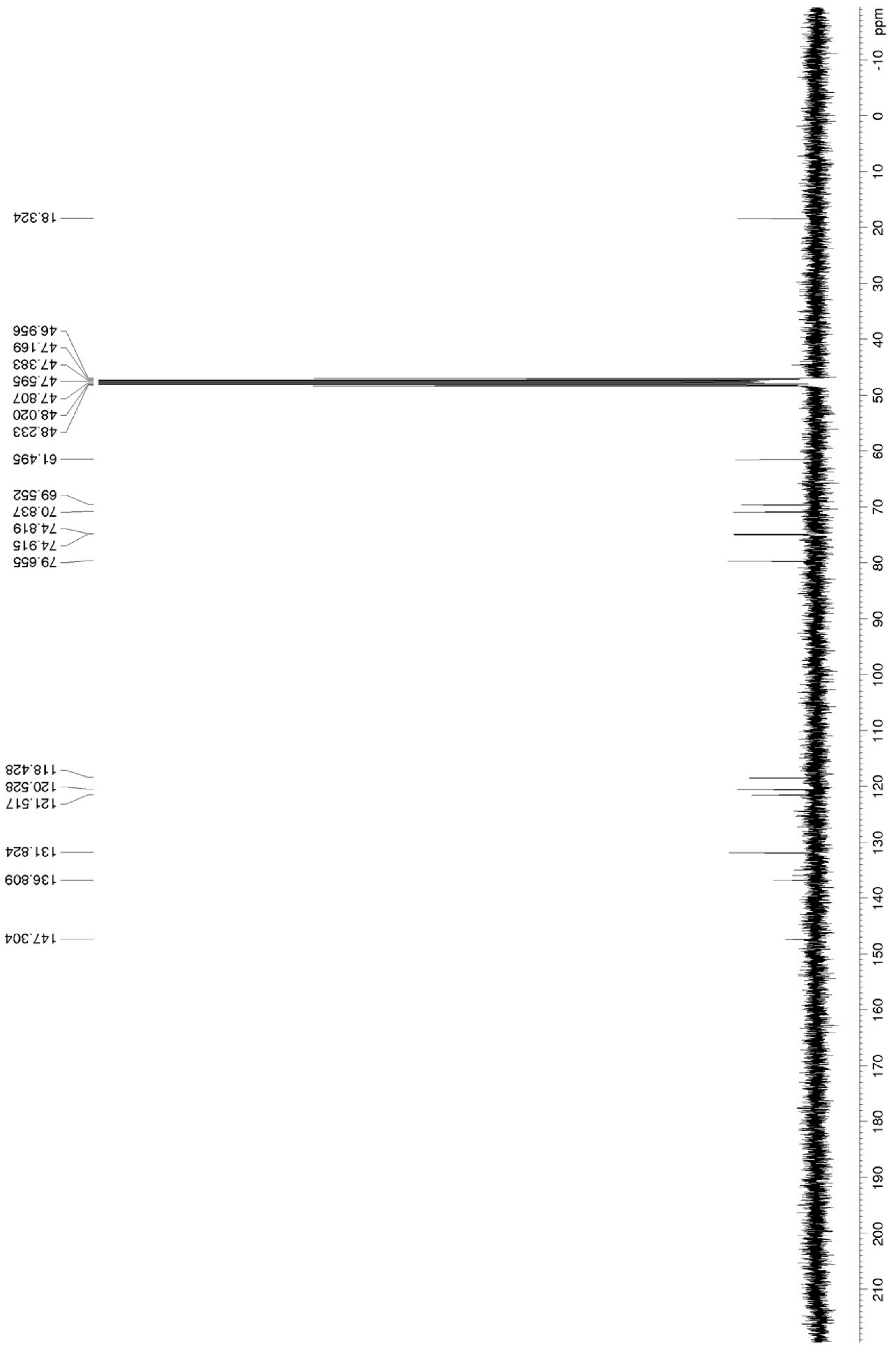
18.324
46.956
47.169
47.383
47.595
47.807
48.020
48.233
61.495
69.552
70.837
74.819
74.915
79.655
118.428
120.528
121.517
131.824
136.809
147.304
ppm

1r ^{1}H-NMR

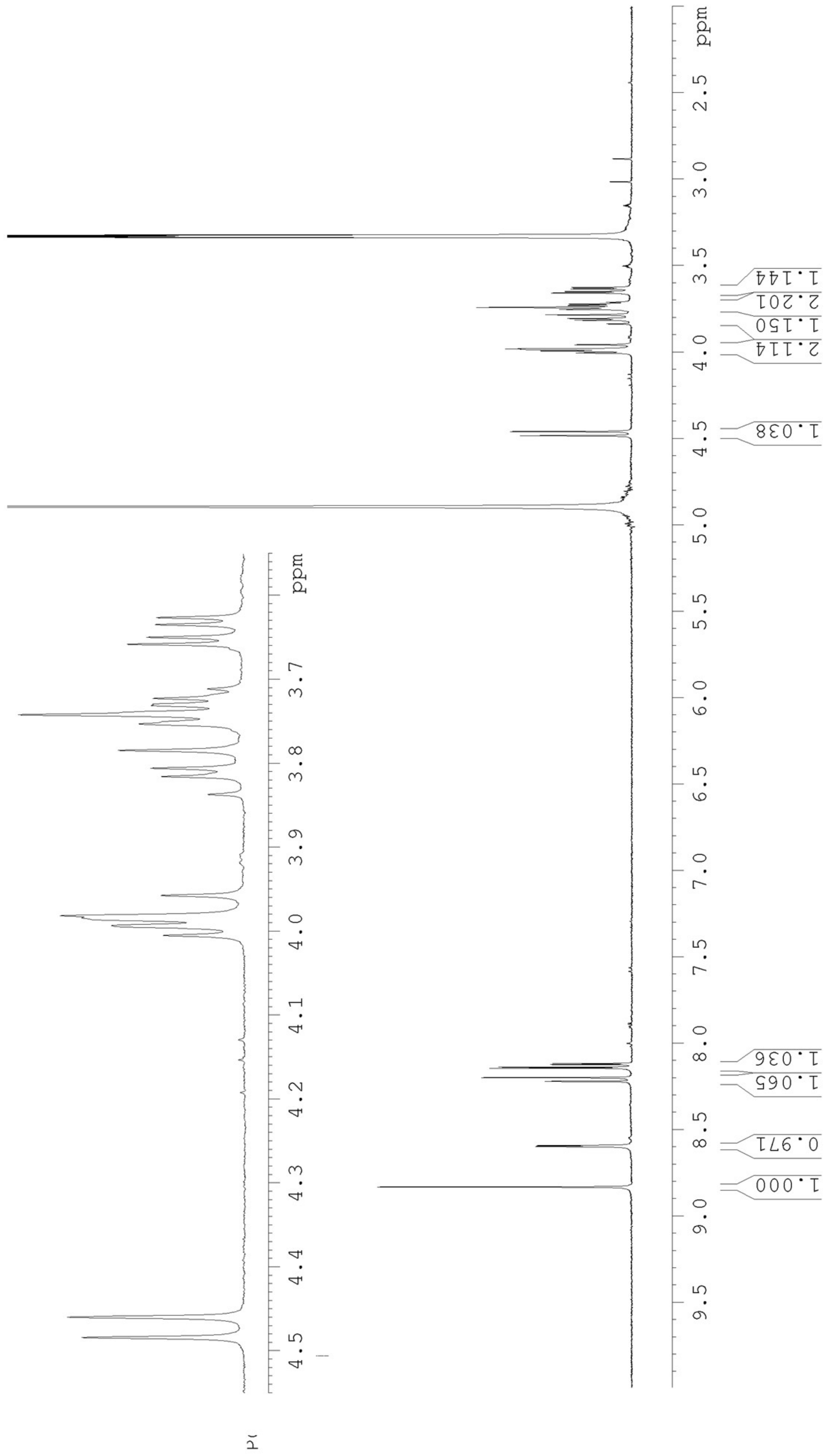

1r ^{13}C-NMR

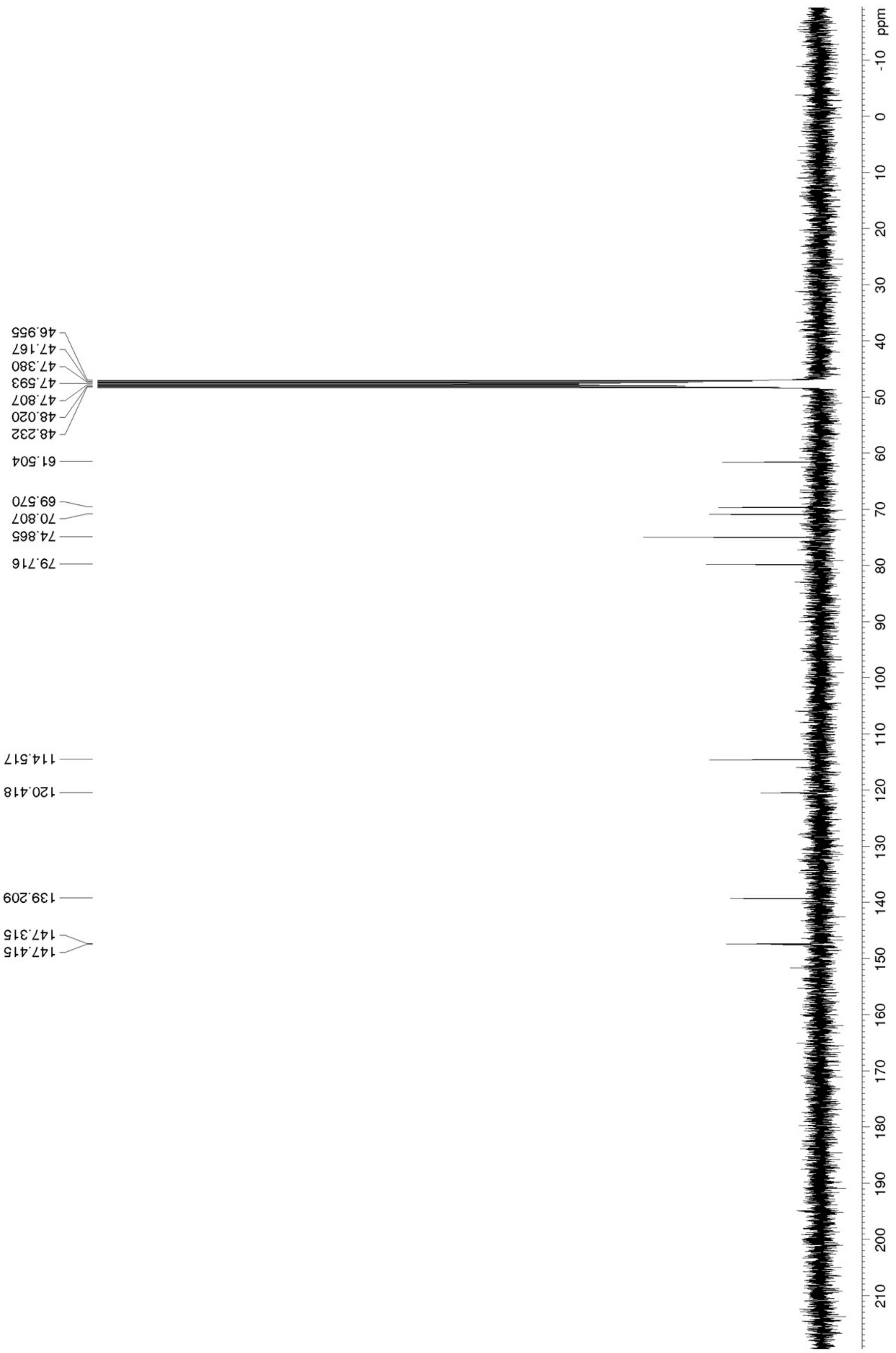
46.955
47.167
47.380
47.593
47.807
48.020
48.232
61.504
69.570
70.807
74.865
79.716
114.517
120.418
139.209
147.315
147.415
ppm

1s ^{1}H-NMR

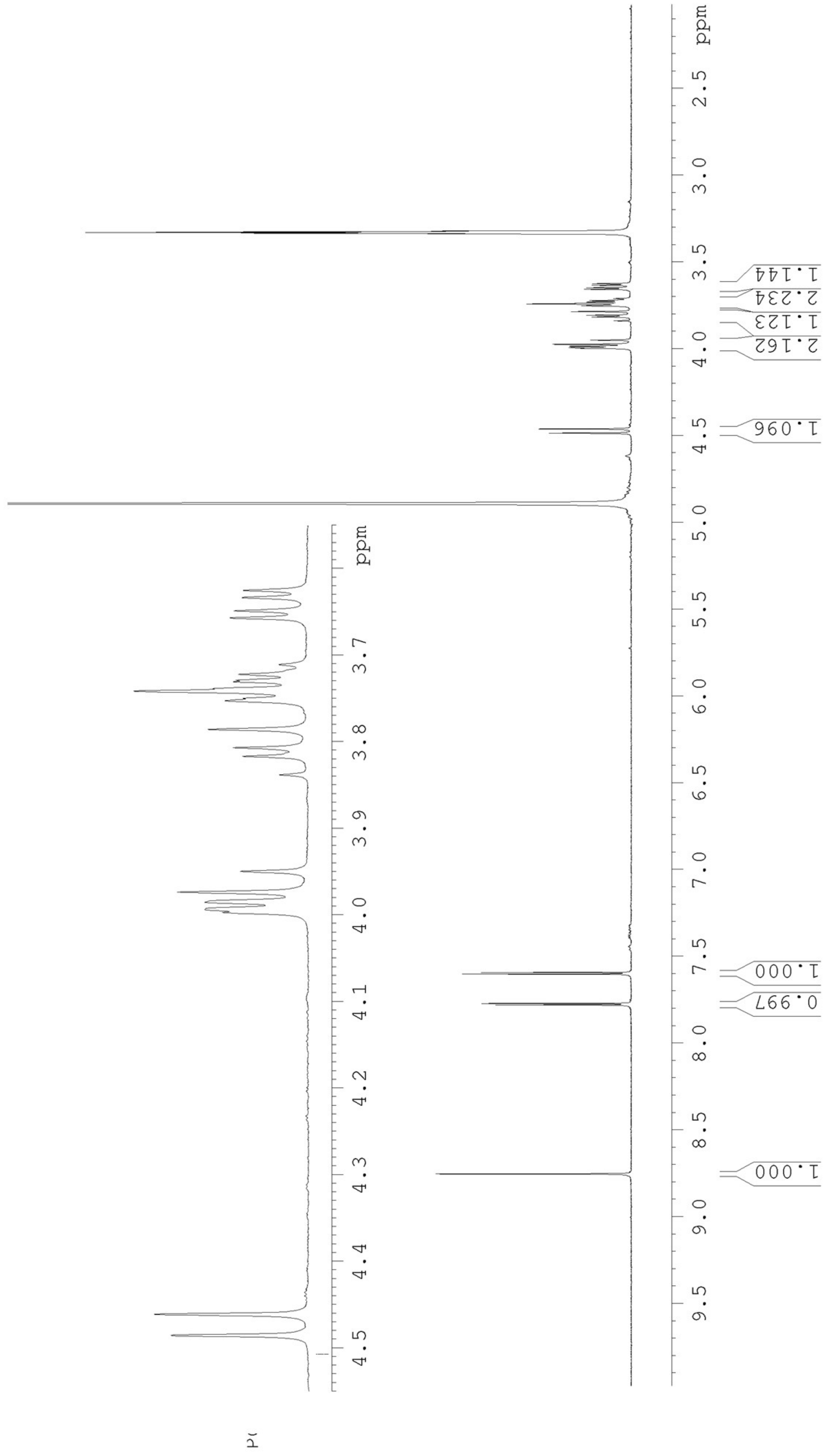

1s ^{13}C-NMR

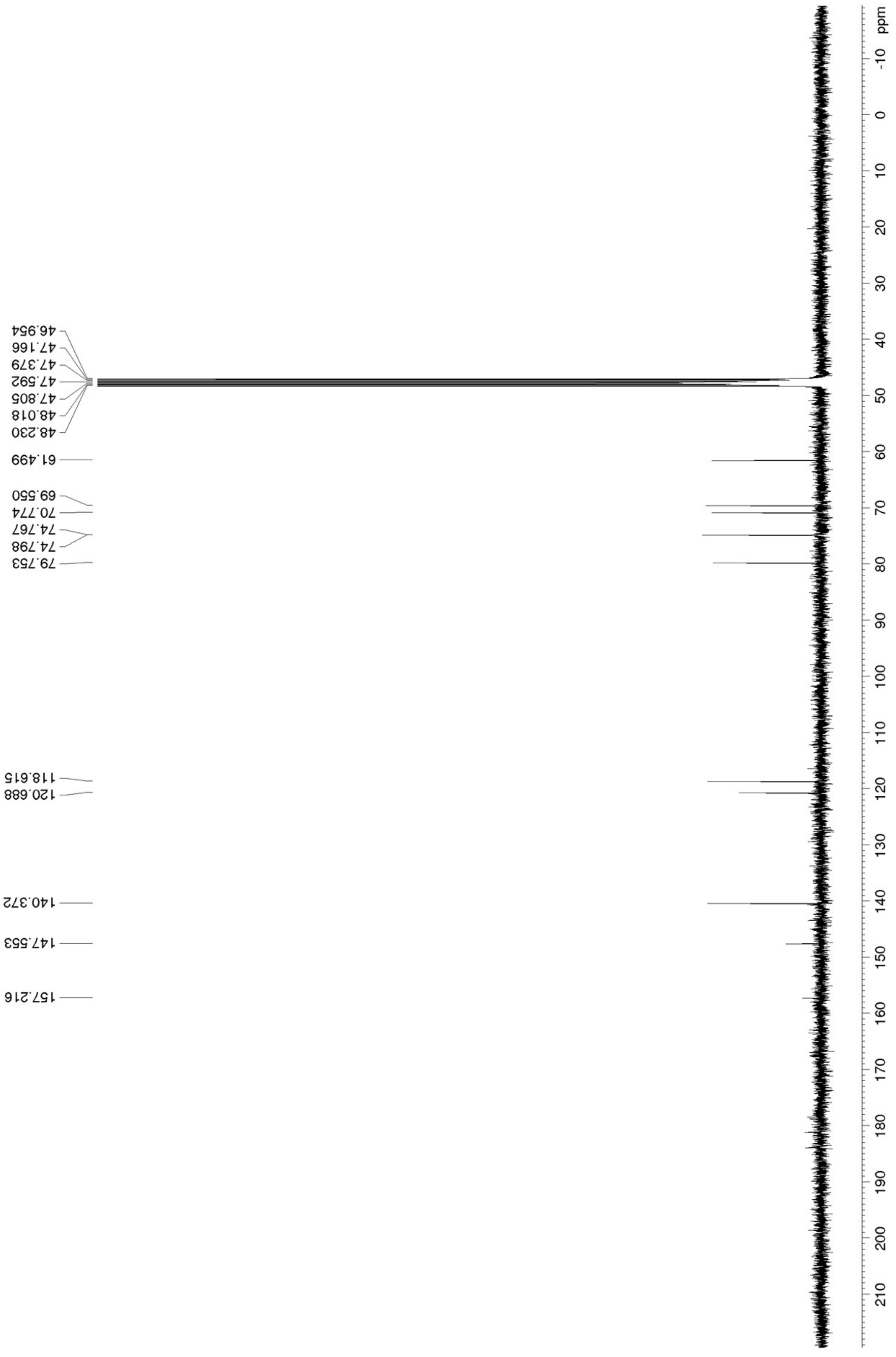

46.954
47.166
47.379
47.592
47.805
48.018
48.230
61.499
69.550
70.774
74.767
74.798
79.753
118.615
120.688
140.372
147.553
157.216
ppm

1t ^{1}H-NMR

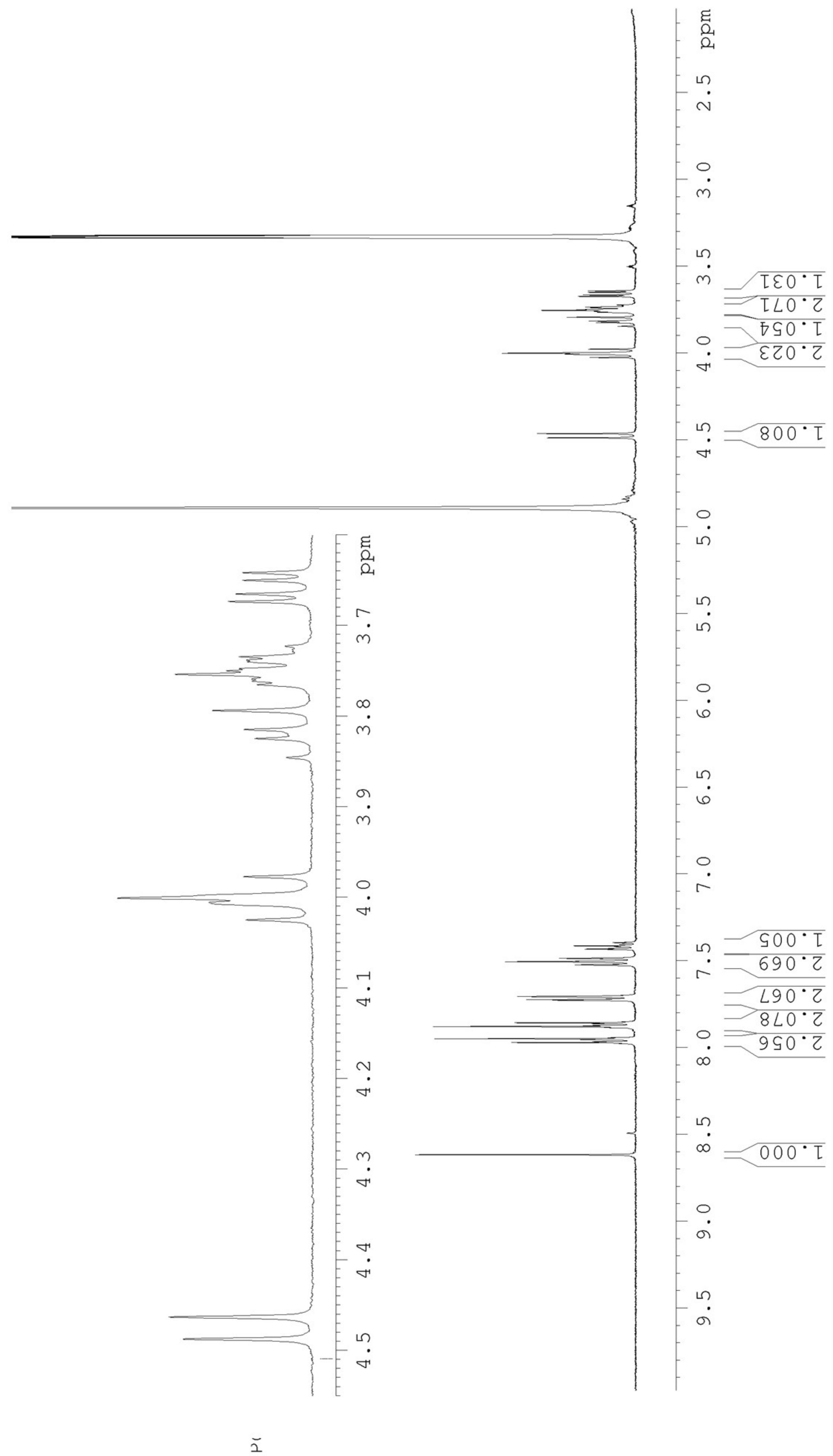

1t ^{13}C-NMR

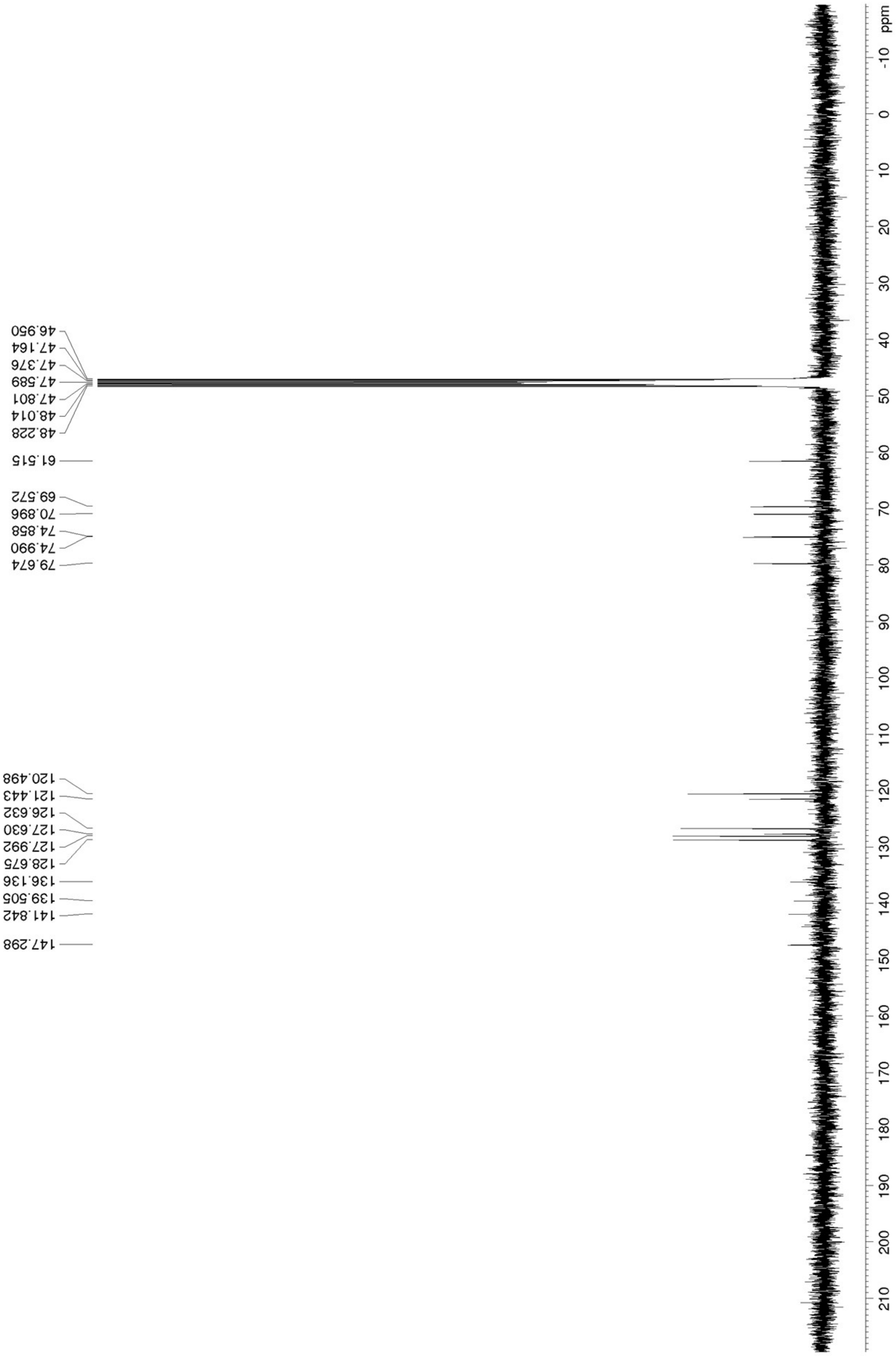

46.950
47.164
47.376
47.589
47.801
48.014
48.228
61.515
69.572
70.896
74.858
74.990
79.674
120.498
121.443
126.632
127.630
127.992
128.675
136.136
139.505
141.842
147.298
ppm

1u ^{1}H-NMR

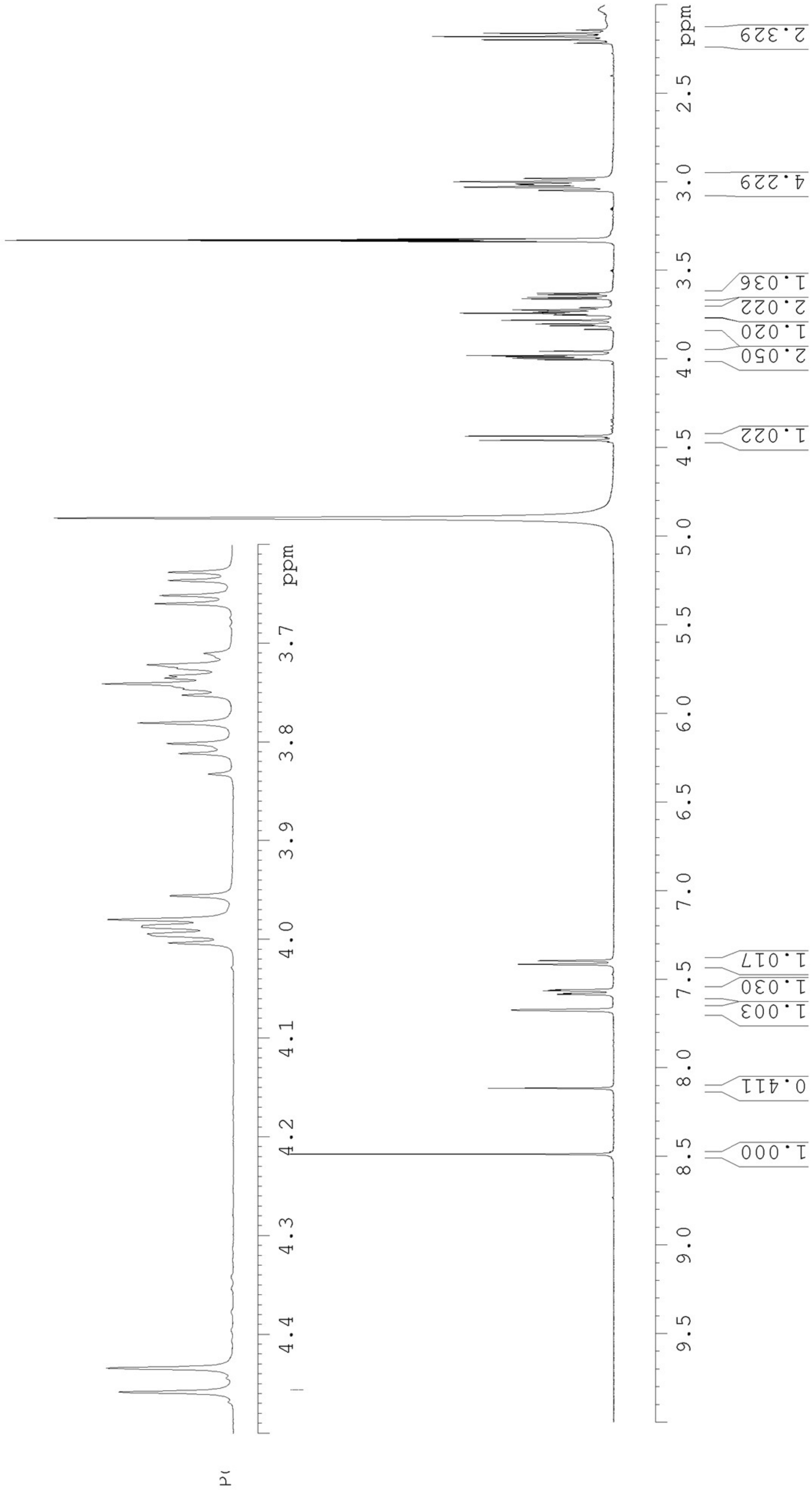

1u ^{13}C-NMR

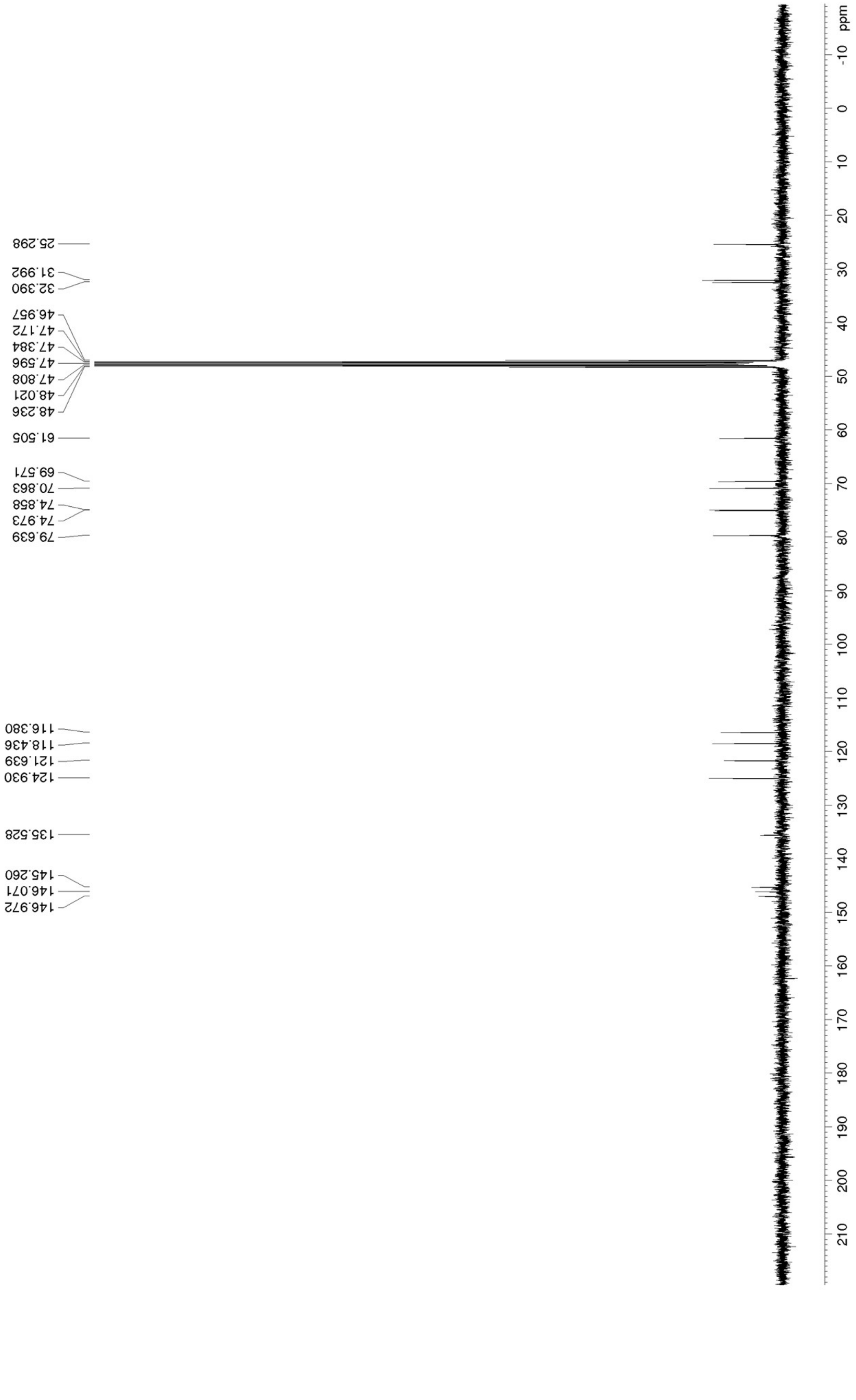
25.298
31.992
32.390
46.957
47.172
47.384
47.596
47.808
48.021
48.236
61.505
69.571
70.863
74.858
74.973
79.639
116.380
118.436
121.639
124.930
135.528
145.260
146.071
146.972
ppm

1v ^{1}H-NMR

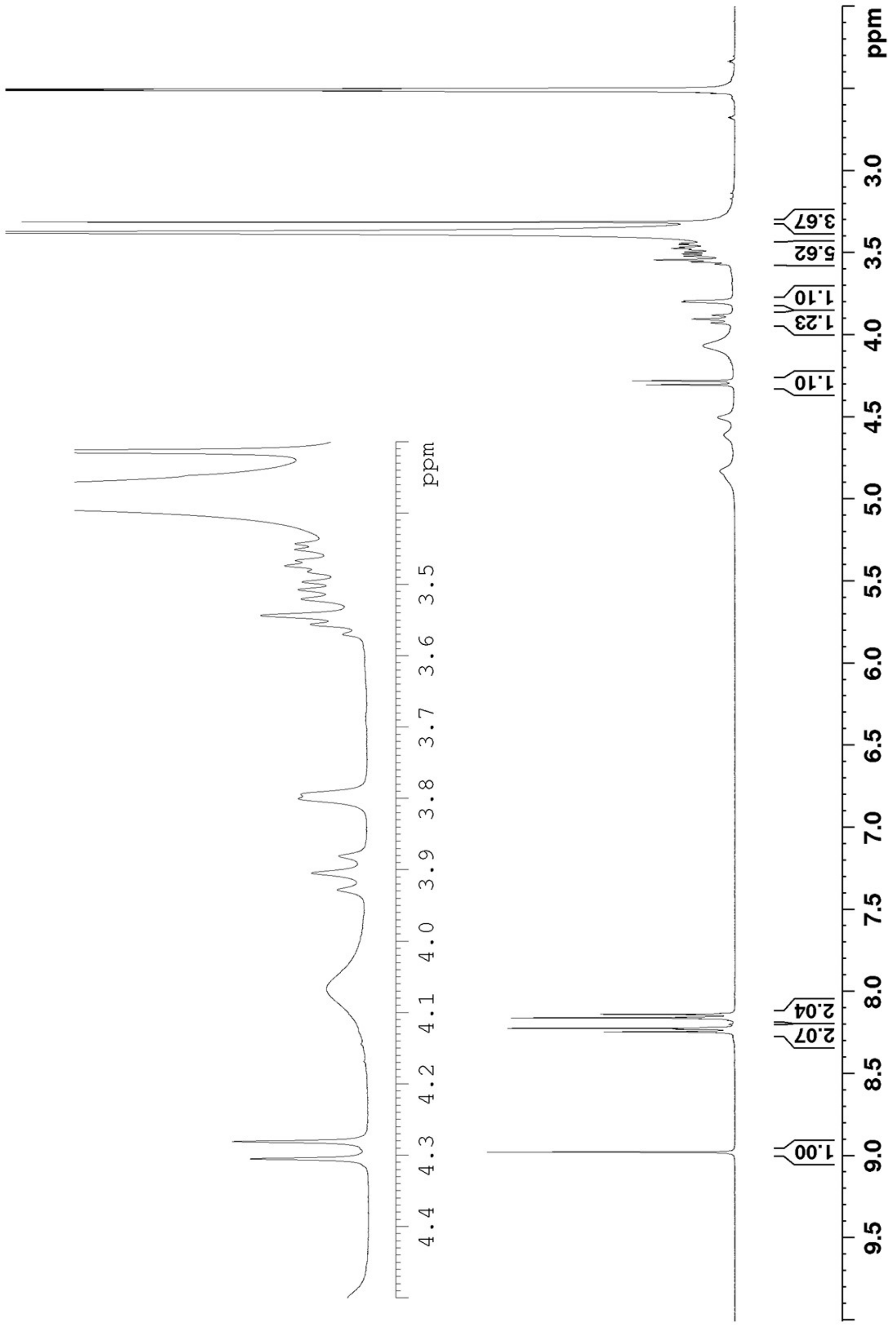

ppm
3.0
3.5
4.0
4.5
5.0
5.5
6.0
6.5
7.0
7.5
8.0
8.5
9.0
9.5
3.67
5.62
1.10
1.23
1.10
2.04
2.07
1.00
ppm
3.5
3.6
3.7
3.8
3.9
4.0
4.1
4.2
4.3
4.4

1v ^{13}C-NMR

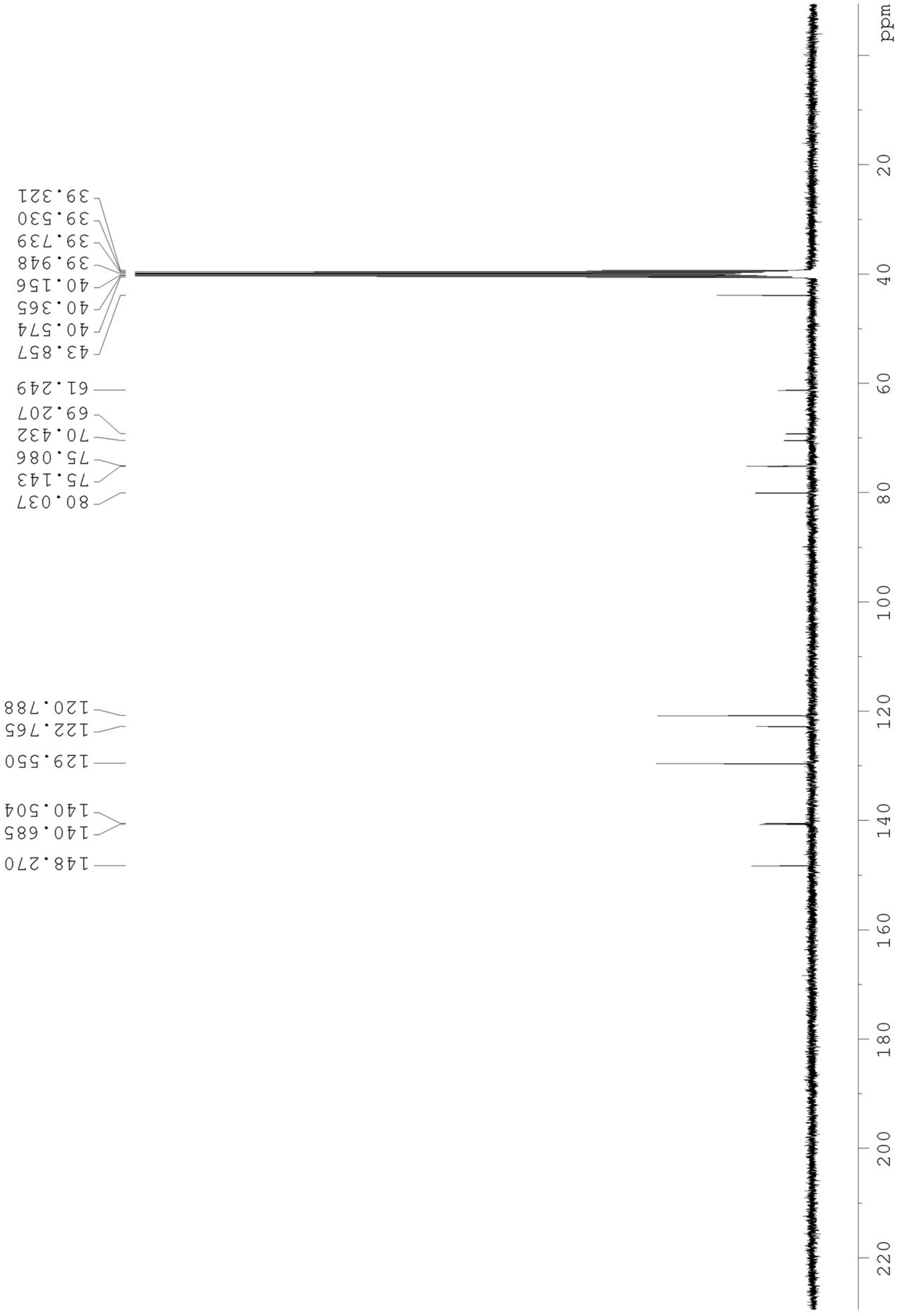
39.321
39.530
39.739
39.948
40.156
40.365
40.574
43.857
61.249
69.207
70.432
75.086
75.143
80.037
120.788
122.765
129.550
140.504
140.685
148.270
ppm
20
40
60
80
100
120
140
160
180
200
220

1w ^{1}H-NMR

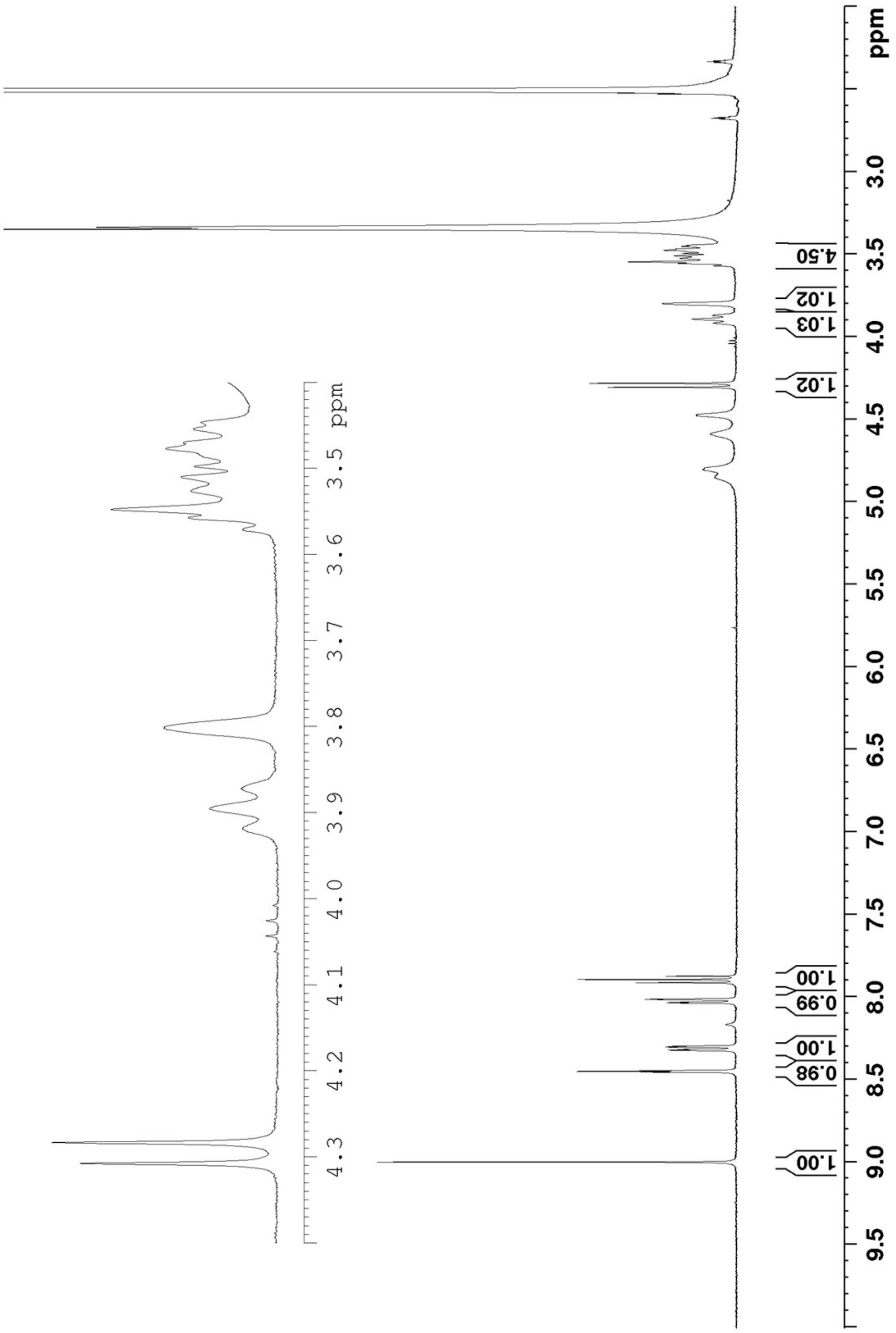

ppm
3.0
3.5
4.0
4.5
5.0
5.5
6.0
6.5
7.0
7.5
8.0
8.5
9.0
9.5
4.50
1.02
1.03
1.02
1.00
0.99
1.00
0.98
1.00
3.5
3.6
3.7
3.8
3.9
4.0
4.1
4.2
4.3

1w ^{13}C-NMR

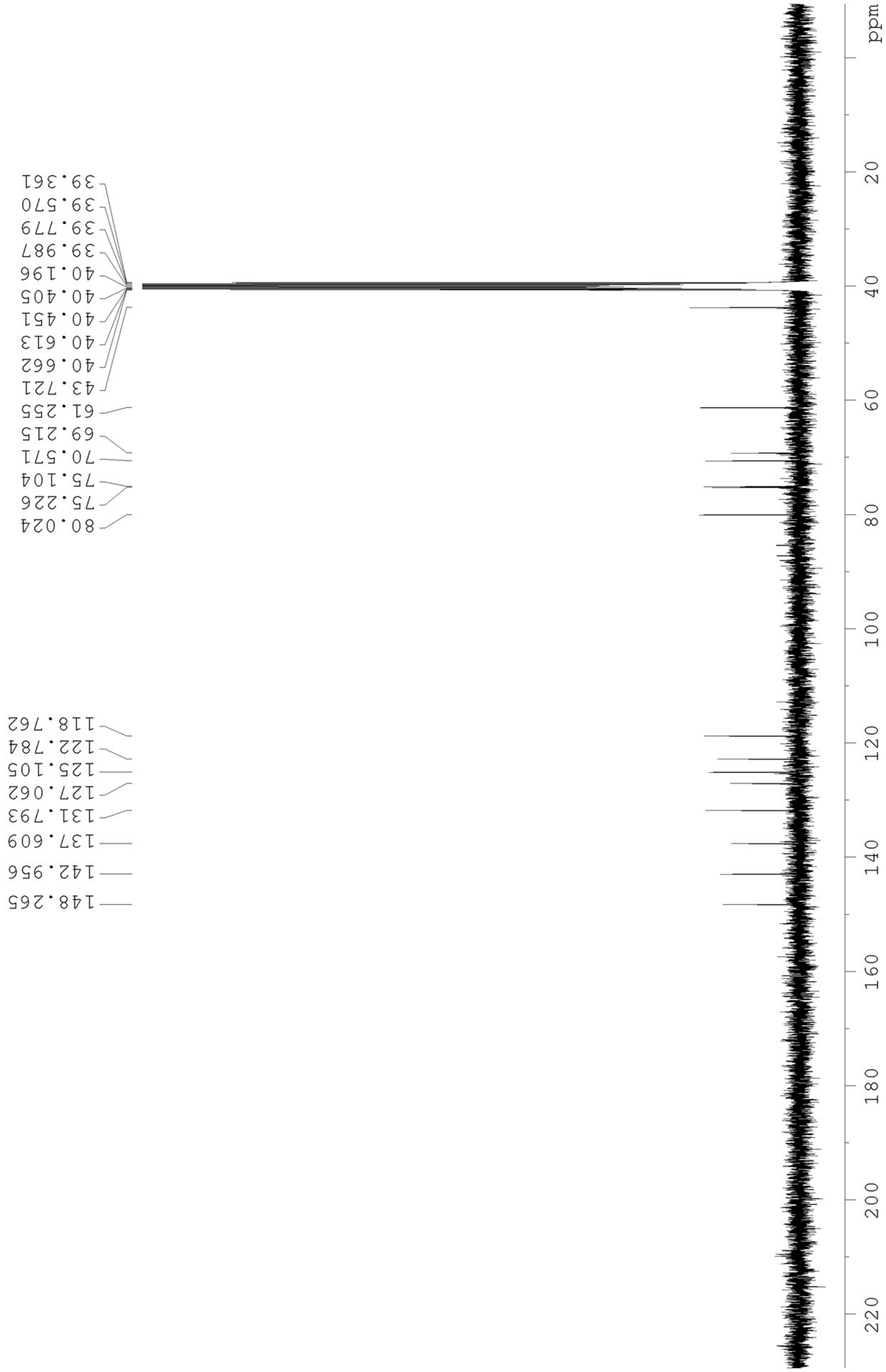
39.361
39.570
39.779
39.987
40.196
40.405
40.451
40.613
40.662
43.721
61.255
69.215
70.571
75.104
75.226
80.024
118.762
122.784
125.105
127.062
131.793
137.609
142.956
148.265
ppm
20
40
60
80
100
120
140
160
180
200
220

1x ^{1}H-NMR

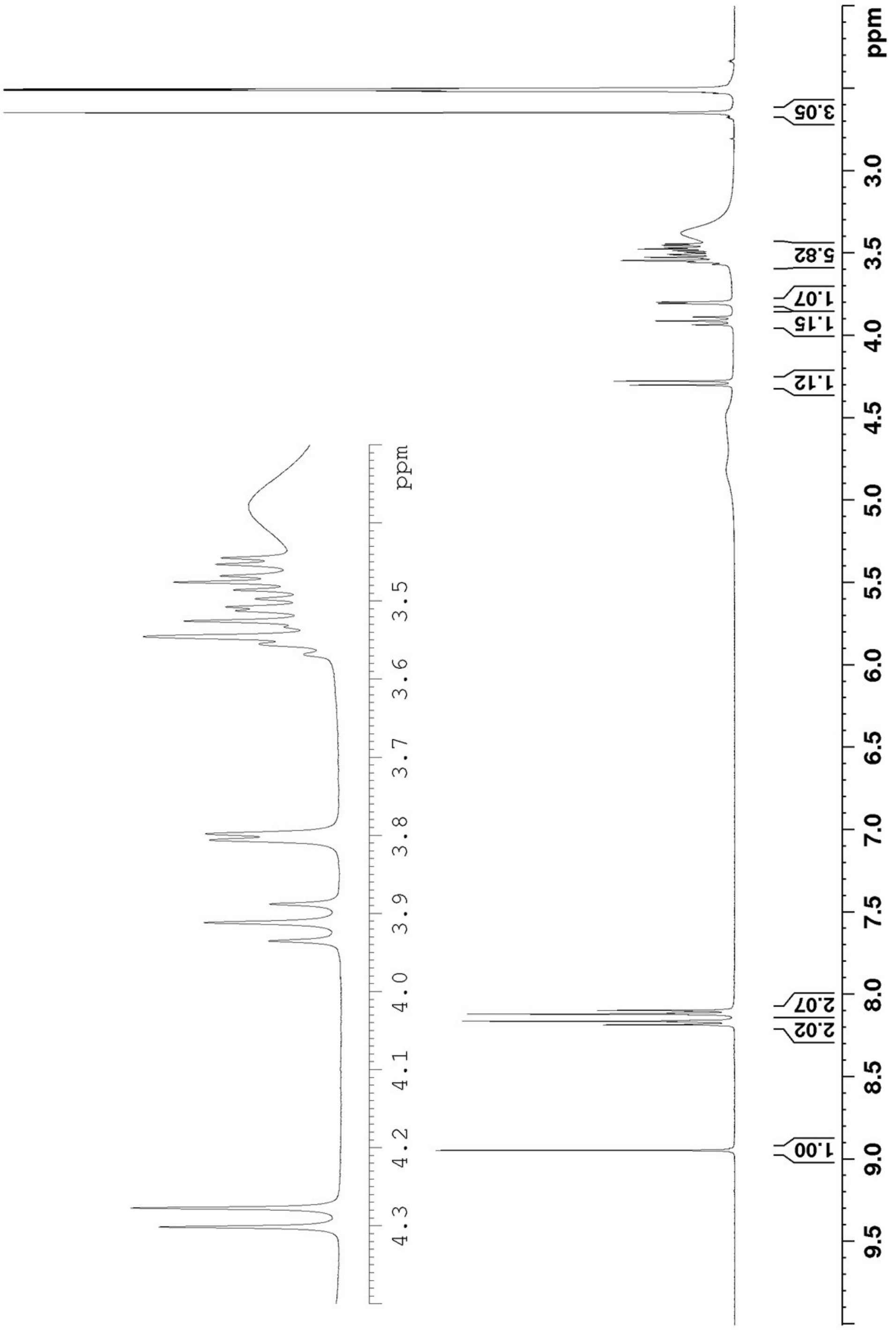
ppm
3.0
3.5
4.0
4.5
5.0
5.5
6.0
6.5
7.0
7.5
8.0
8.5
9.0
9.5
3.05
5.82
1.07
1.15
1.12
2.07
2.02
1.00
ppm
3.5
3.6
3.7
3.8
3.9
4.0
4.1
4.2
4.3

1x ^{13}C-NMR

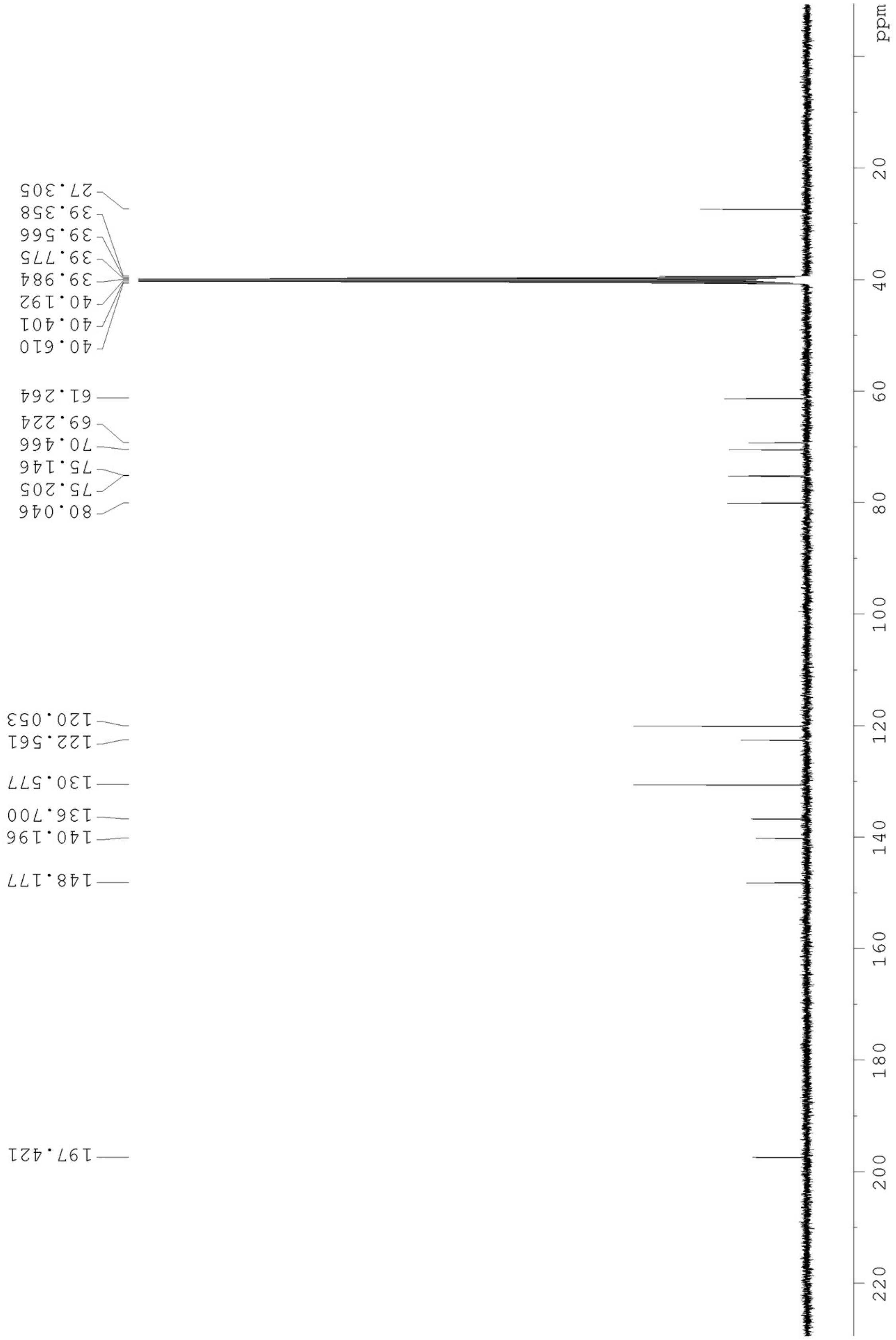
27.305
39.358
39.566
39.775
39.984
40.192
40.401
40.610
61.264
69.224
70.466
75.146
75.205
80.046
120.053
122.561
130.577
136.700
140.196
148.177
197.421
ppm
20
40
60
80
100
120
140
160
180
200
220

1y ^{1}H-NMR

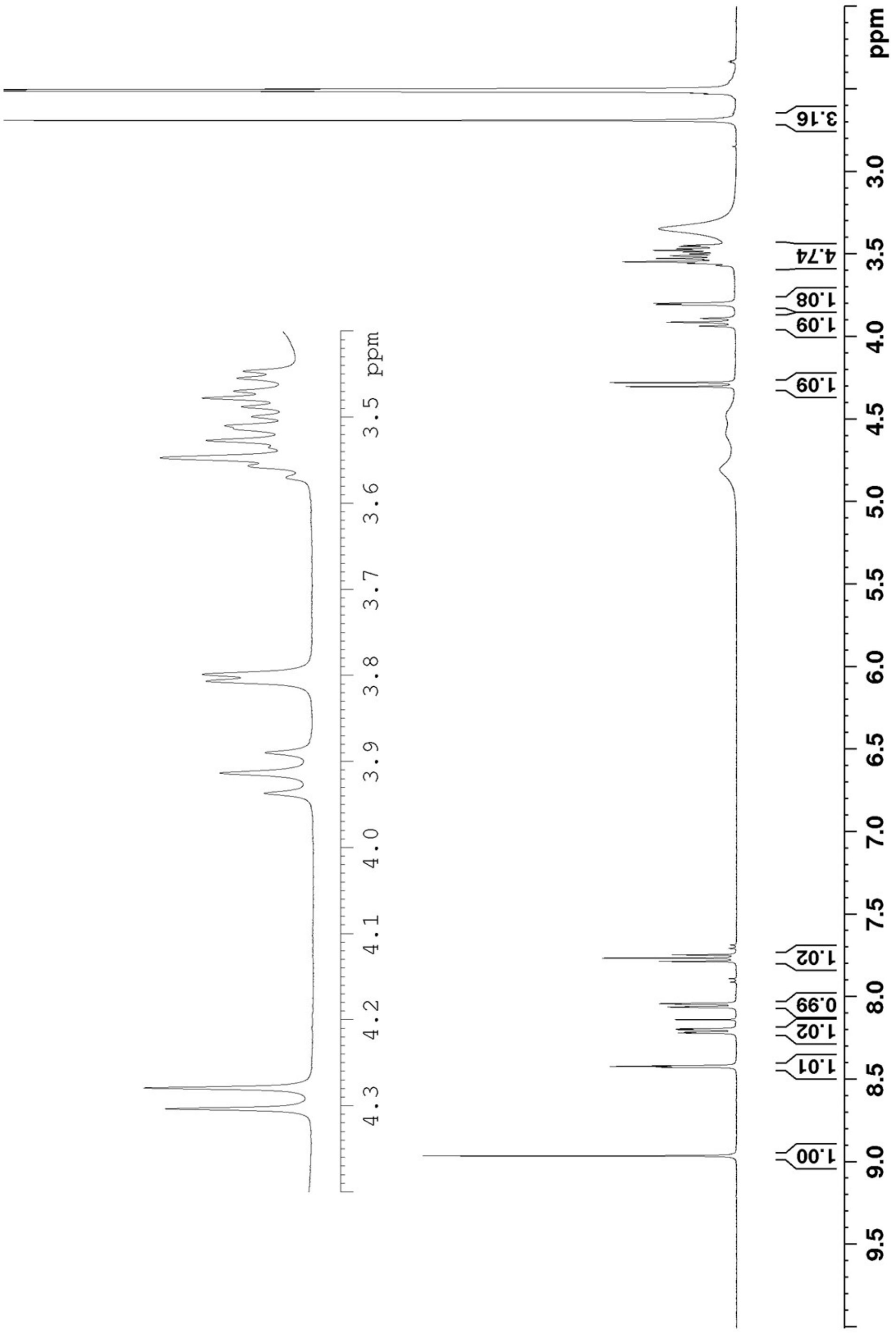
ppm
3.16
4.74
1.08
1.09
1.09
1.02
0.99
1.02
1.01
1.00
3.0
3.5
4.0
4.5
5.0
5.5
6.0
6.5
7.0
7.5
8.0
8.5
9.0
9.5
3.5
3.6
3.7
3.8
3.9
4.0
4.1
4.2
4.3

1y ^{13}C-NMR

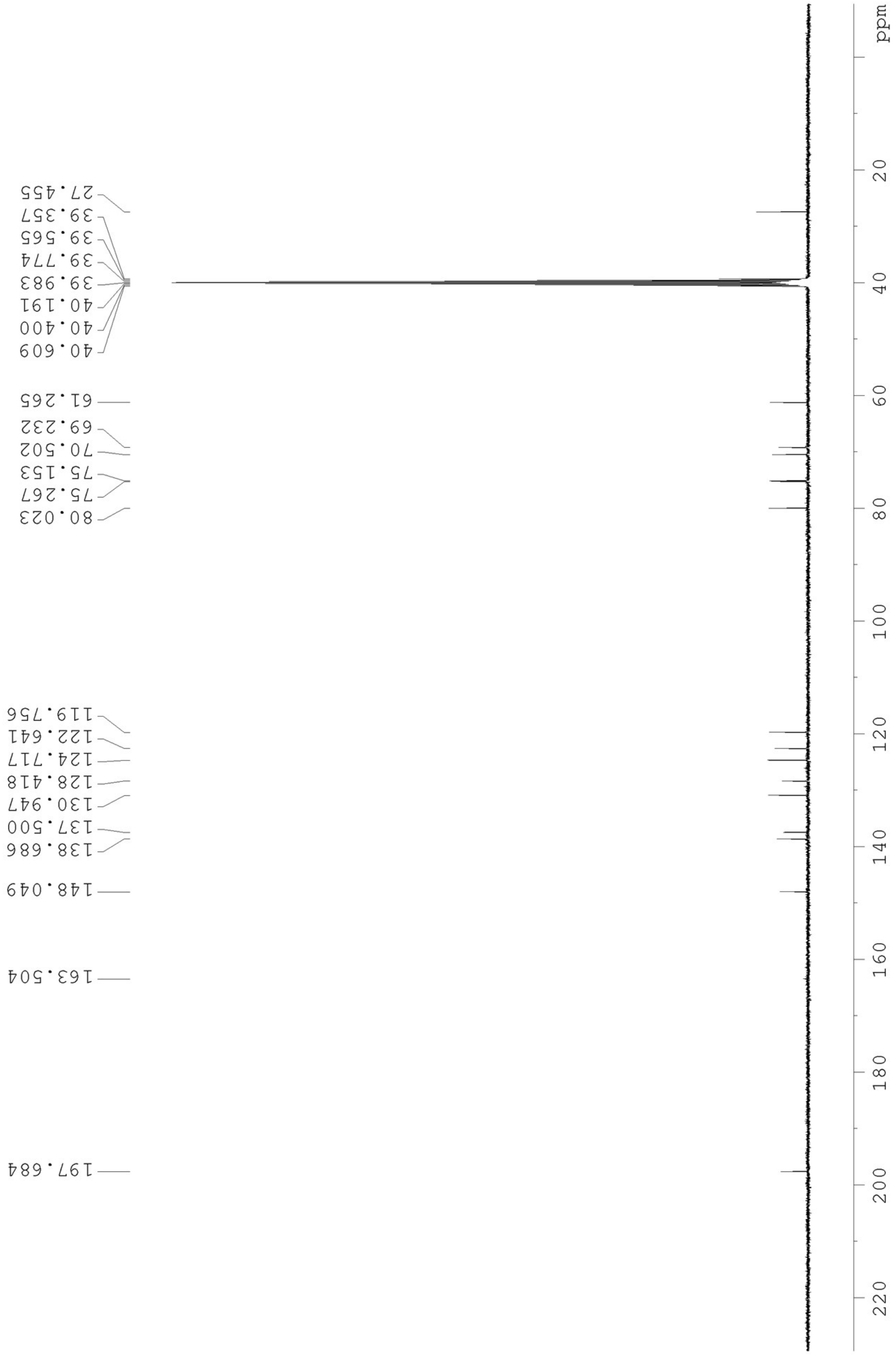
27.455
39.357
39.565
39.774
39.983
40.191
40.400
40.609
61.265
69.232
70.502
75.153
75.267
80.023
119.756
122.641
124.717
128.418
130.947
137.500
138.686
148.049
163.504
197.684
ppm
20
40
60
80
100
120
140
160
180
200
220

1z ^{1}H-NMR

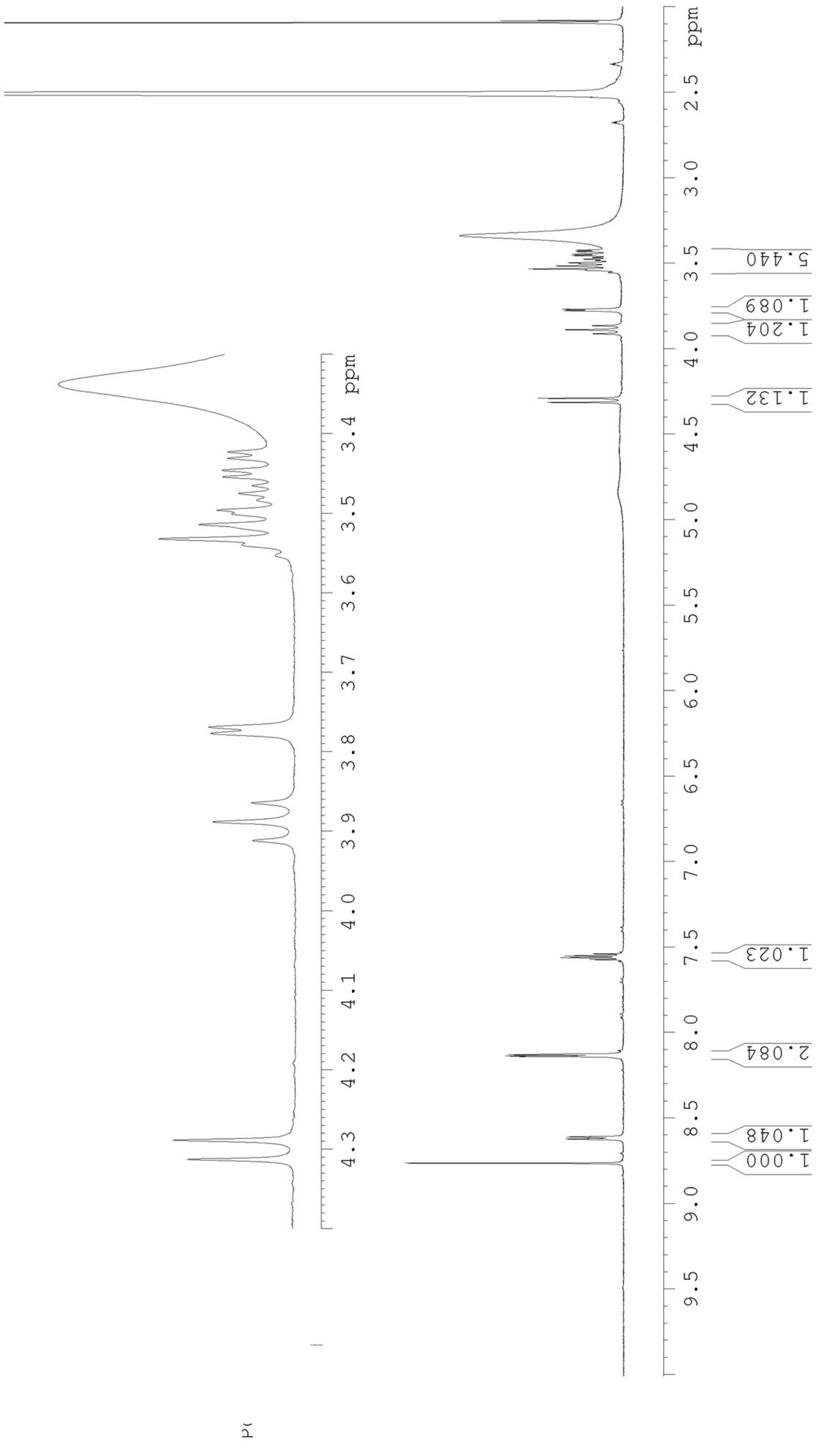

1z ^{13}C-NMR

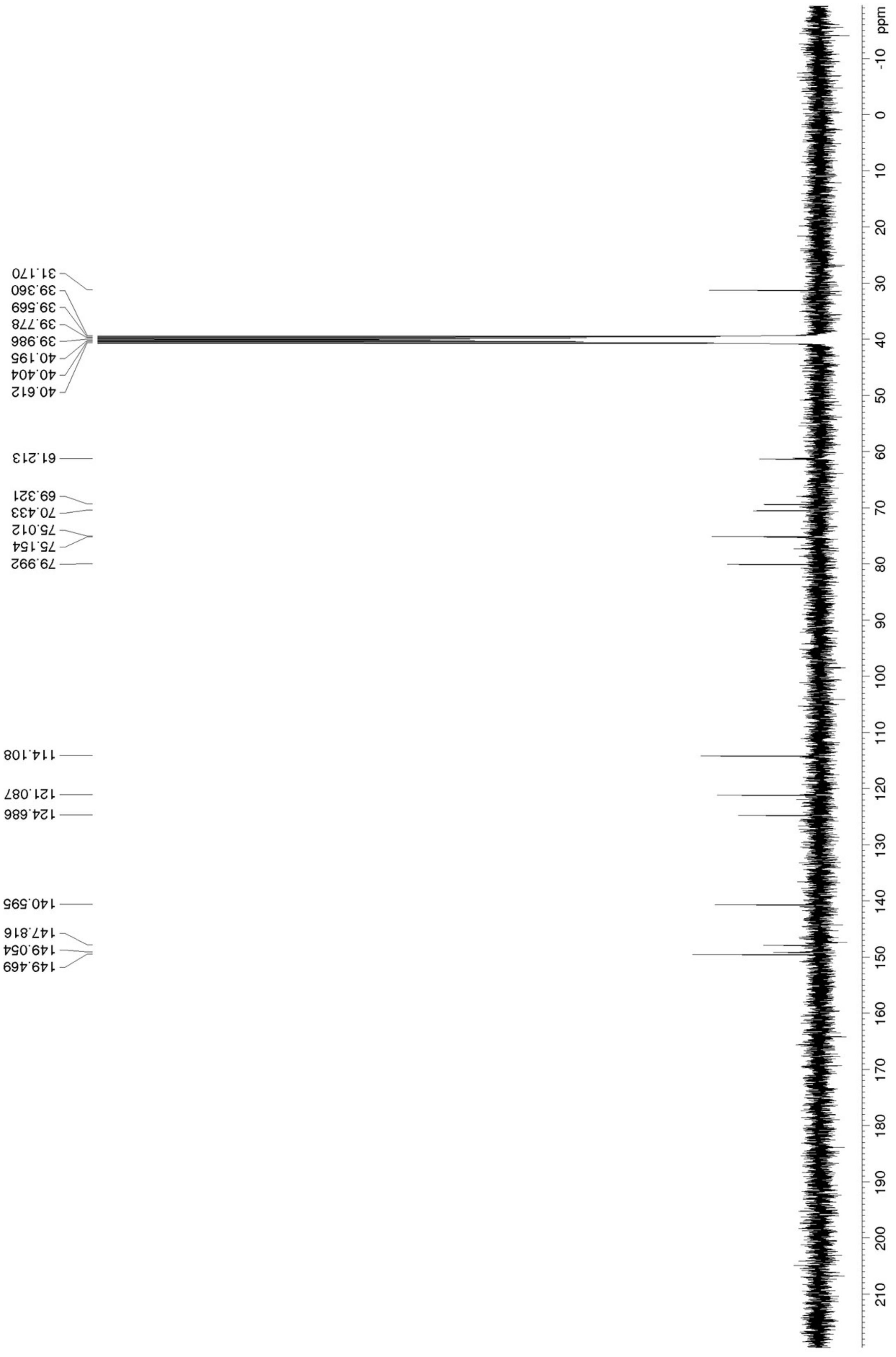
31.170
39.360
39.569
39.778
39.986
40.195
40.404
40.612
61.213
69.321
70.433
75.012
75.154
79.992
114.108
121.087
124.686
140.595
147.816
149.054
149.469
ppm

3 ^{1}H-NMR

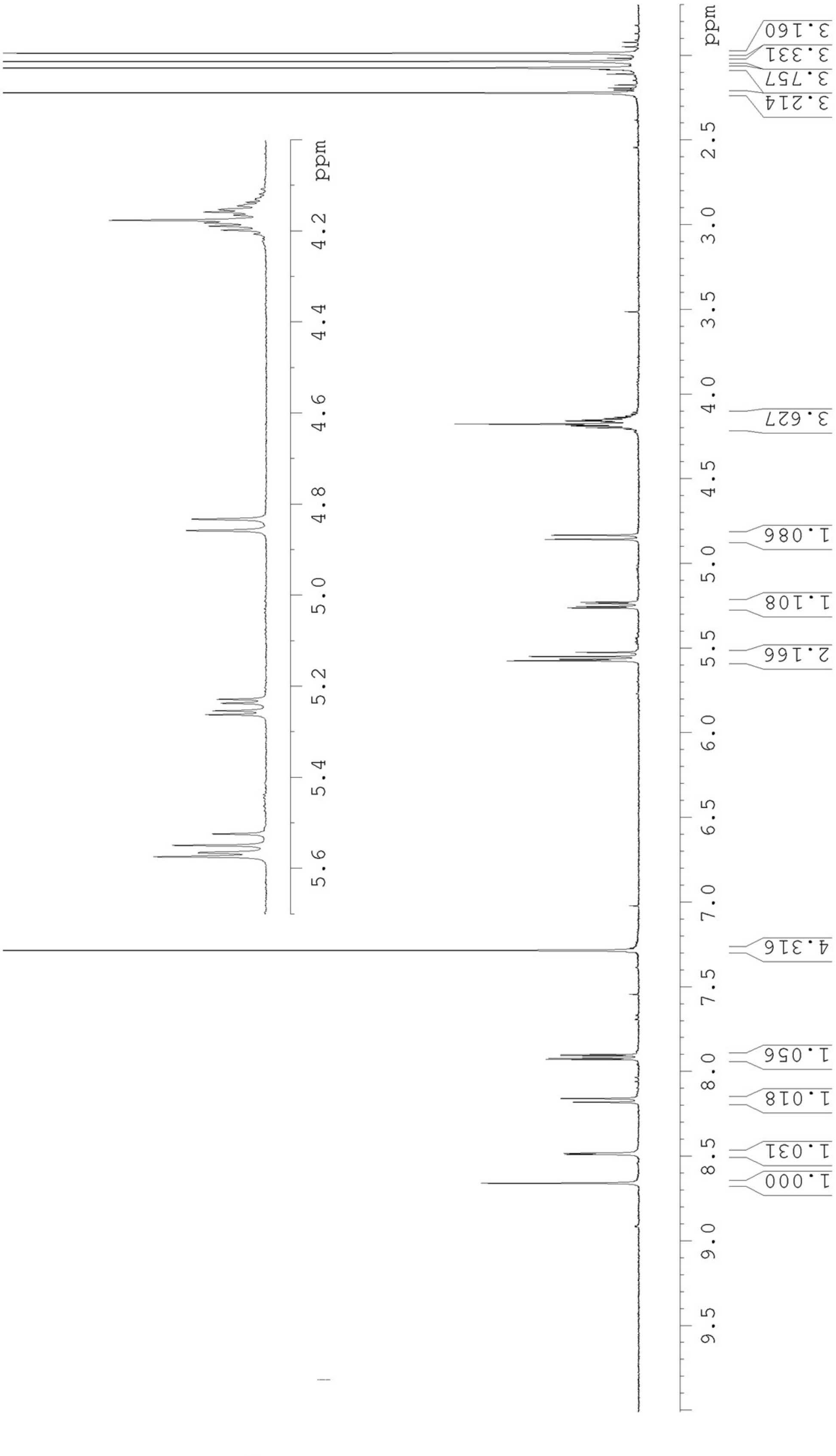
ppm
2.5
3.0
3.5
4.0
4.5
5.0
5.5
6.0
6.5
7.0
7.5
8.0
8.5
9.0
9.5
3.160
3.331
3.757
3.214
3.627
1.086
1.108
2.166
4.316
1.056
1.018
1.031
1.000
ppm
4.2
4.4
4.6
4.8
5.0
5.2
5.4
5.6

3 ^{13}C-NMR

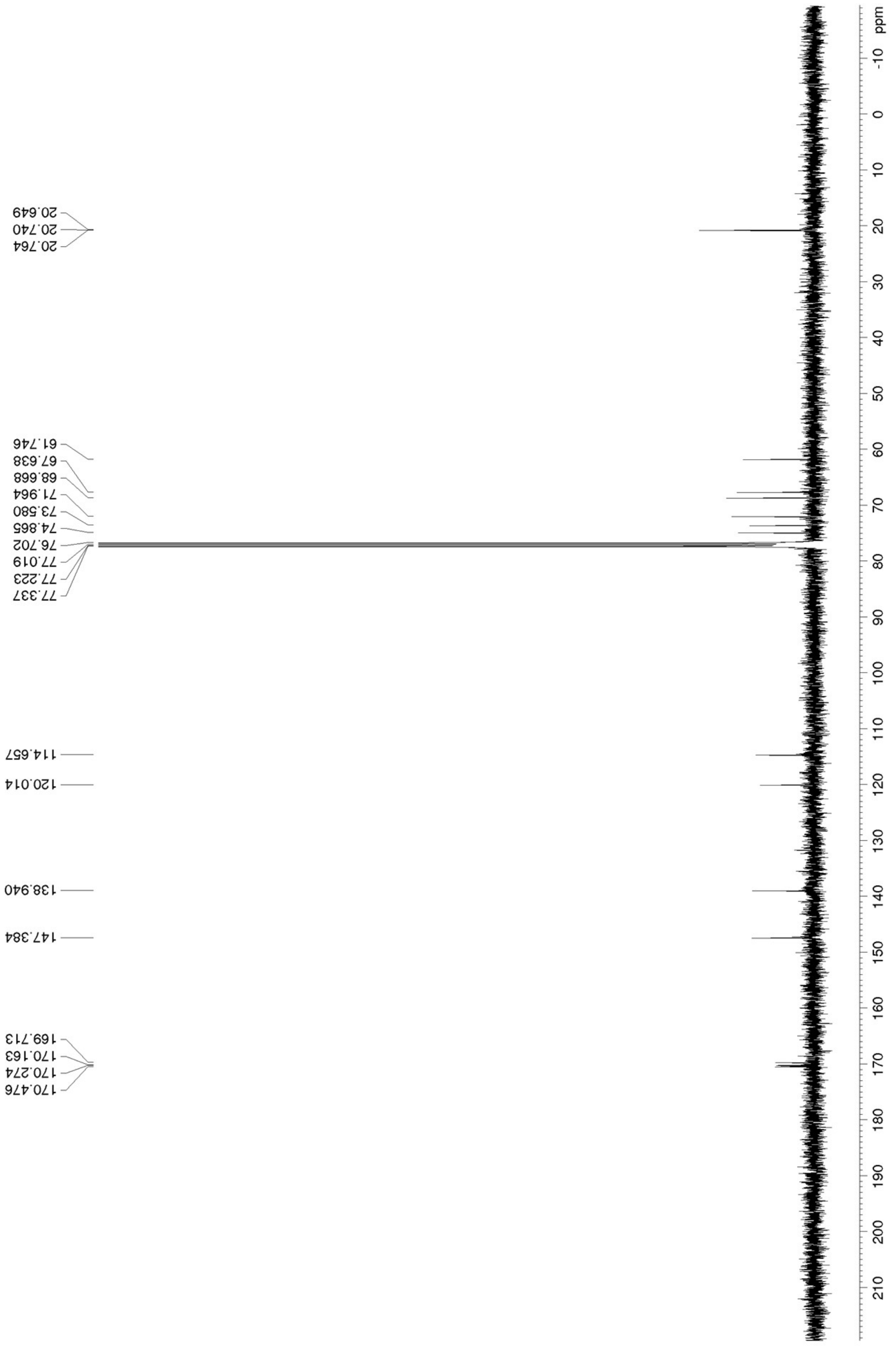
20.649
20.740
20.764
61.746
67.638
68.668
71.964
73.580
74.865
76.702
77.019
77.223
77.337
114.657
120.014
138.940
147.384
169.713
170.163
170.274
170.476
ppm

Compound **8** ^{1}H-NMR

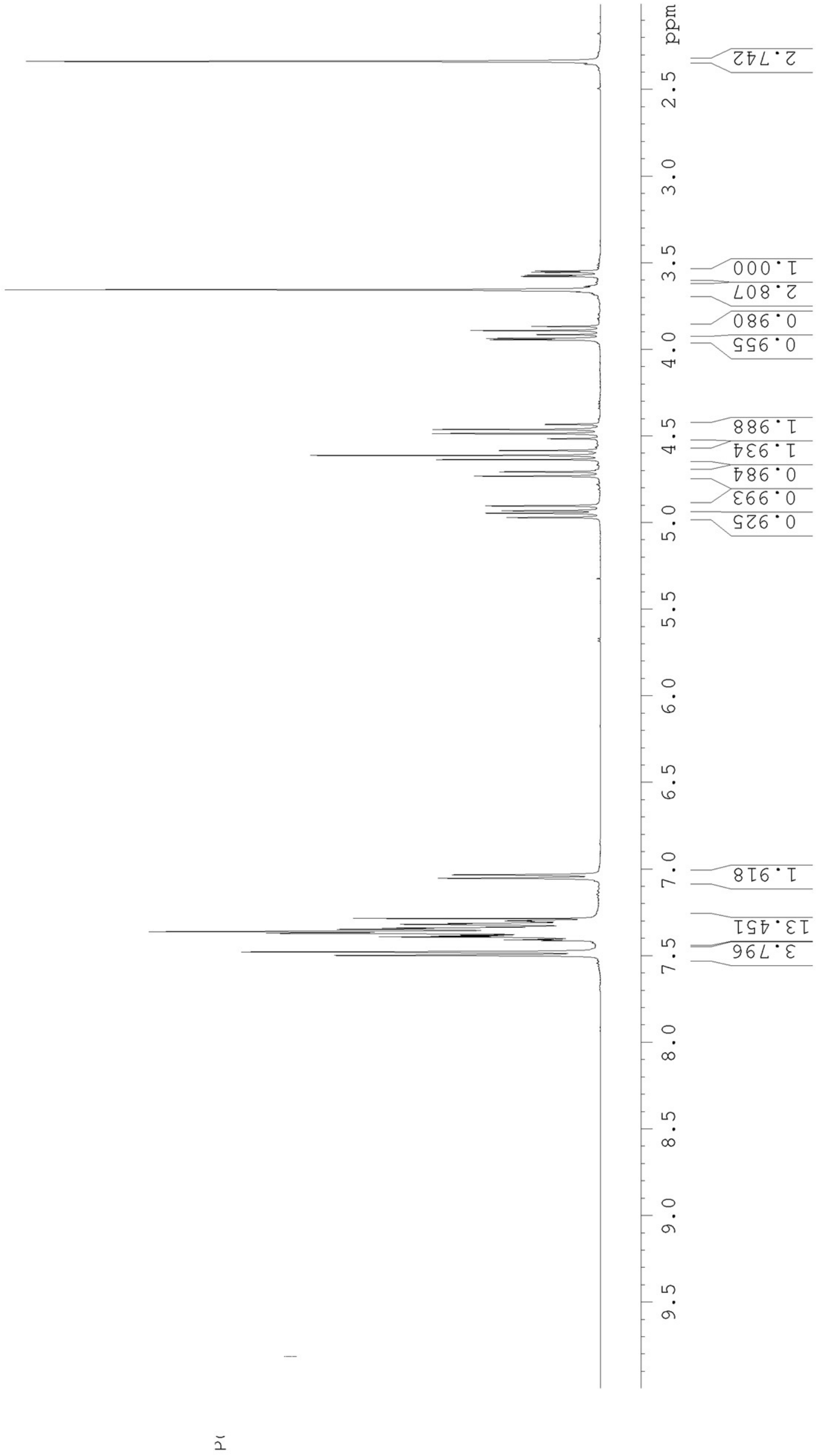

Compound **8** ^{13}C-NMR

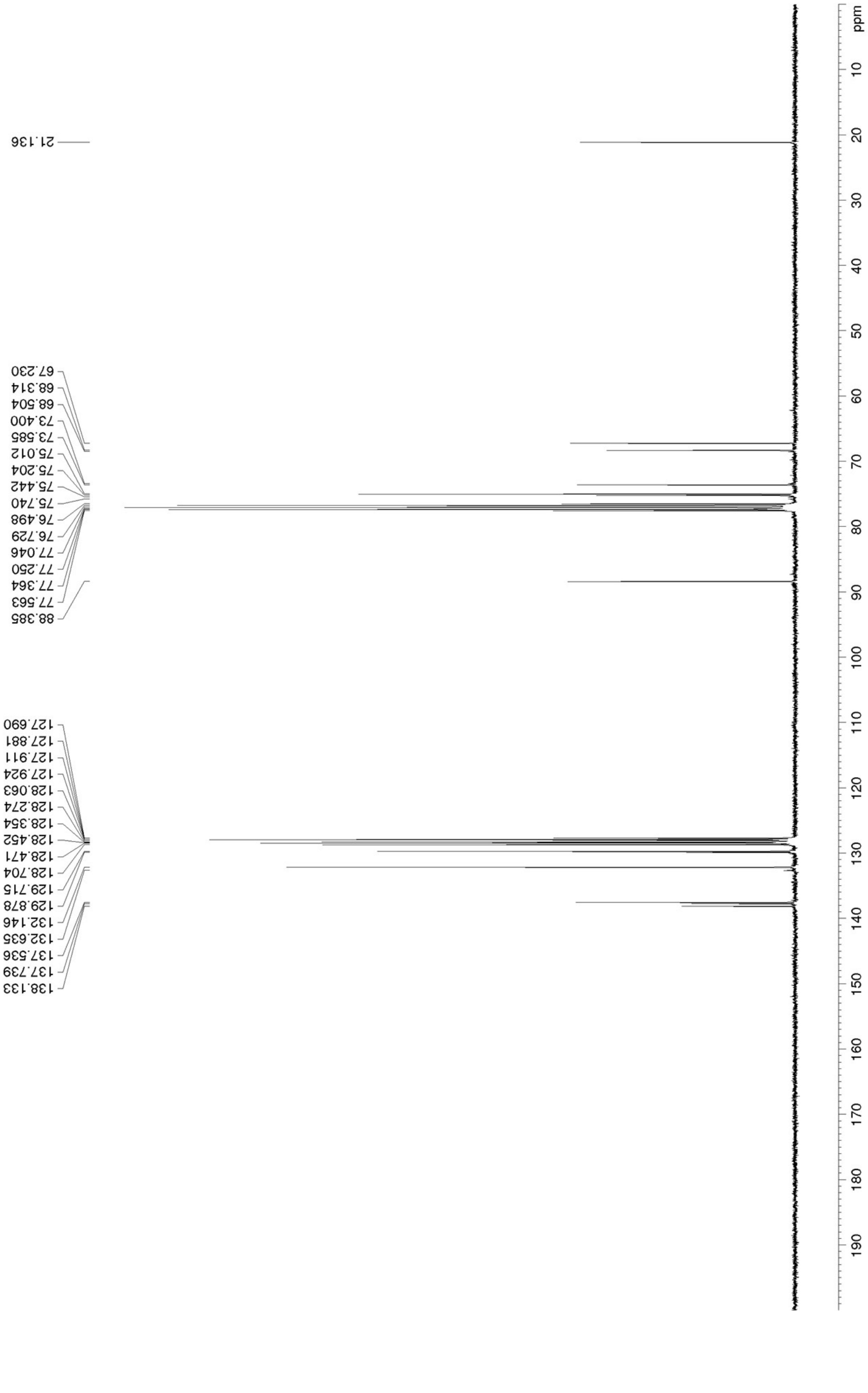

Compound **9** ^{1}H-NMR

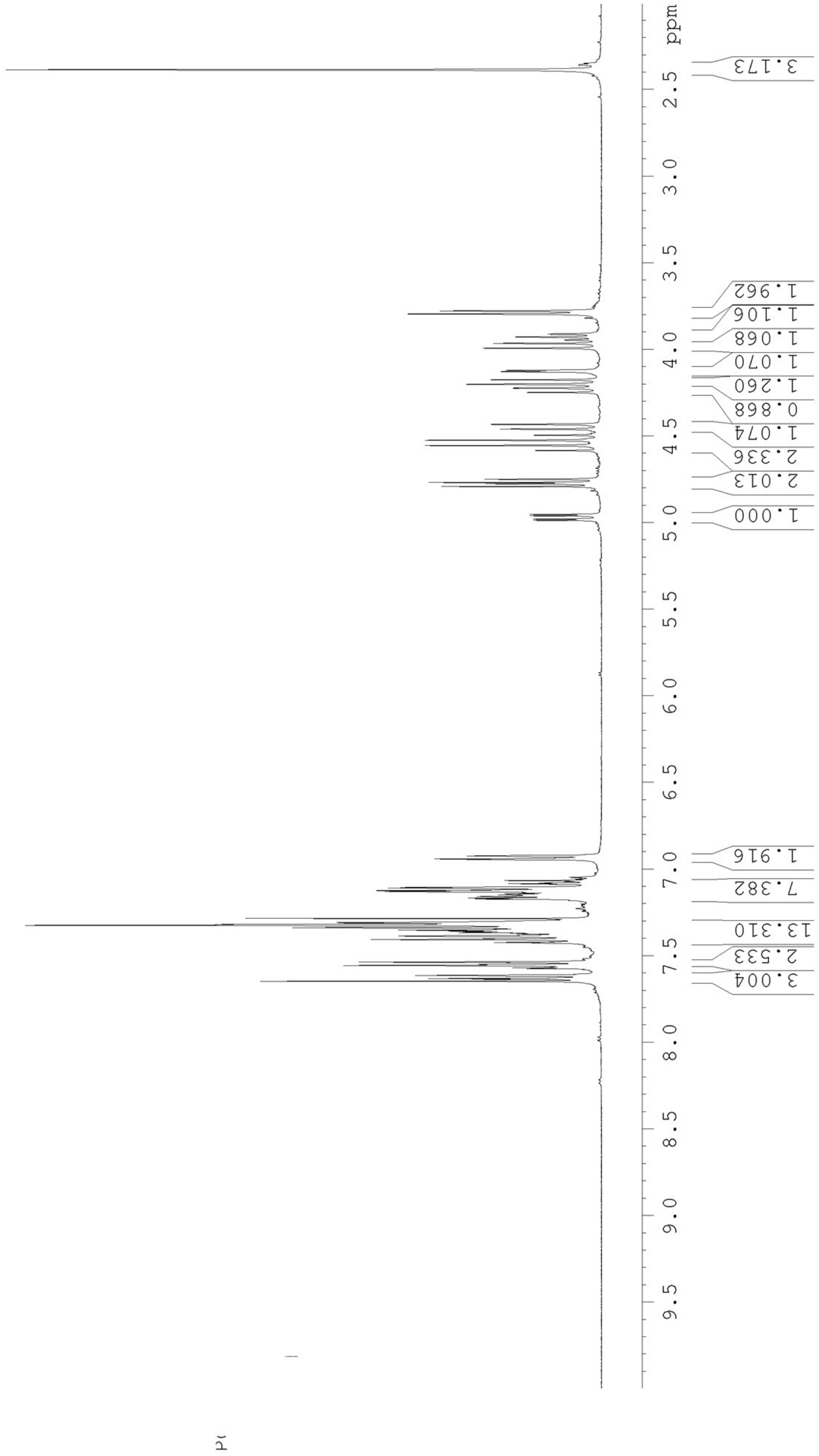

Compound **9** ^{13}C-NMR

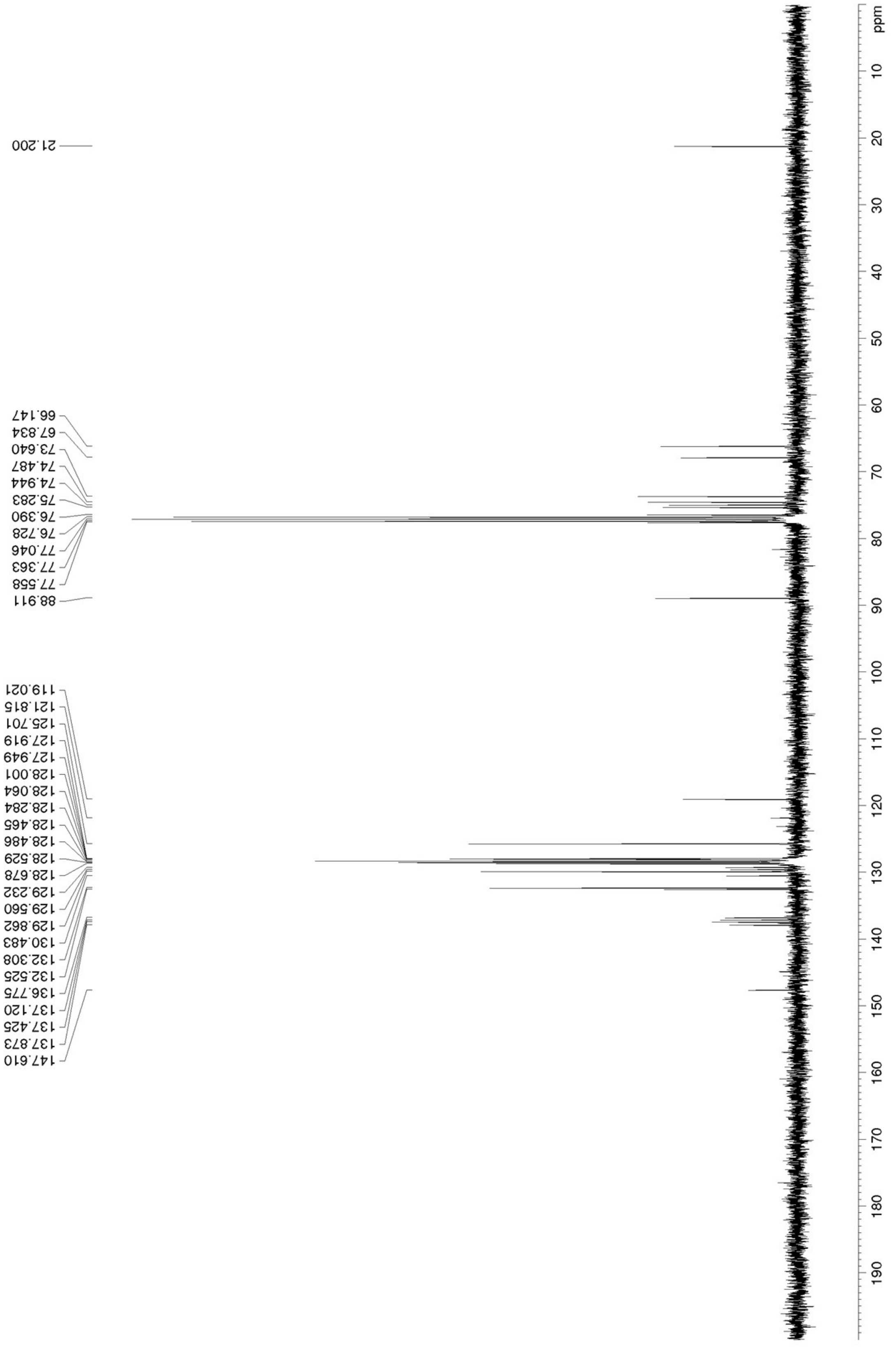

Compound **10** ^{1}H-NMR

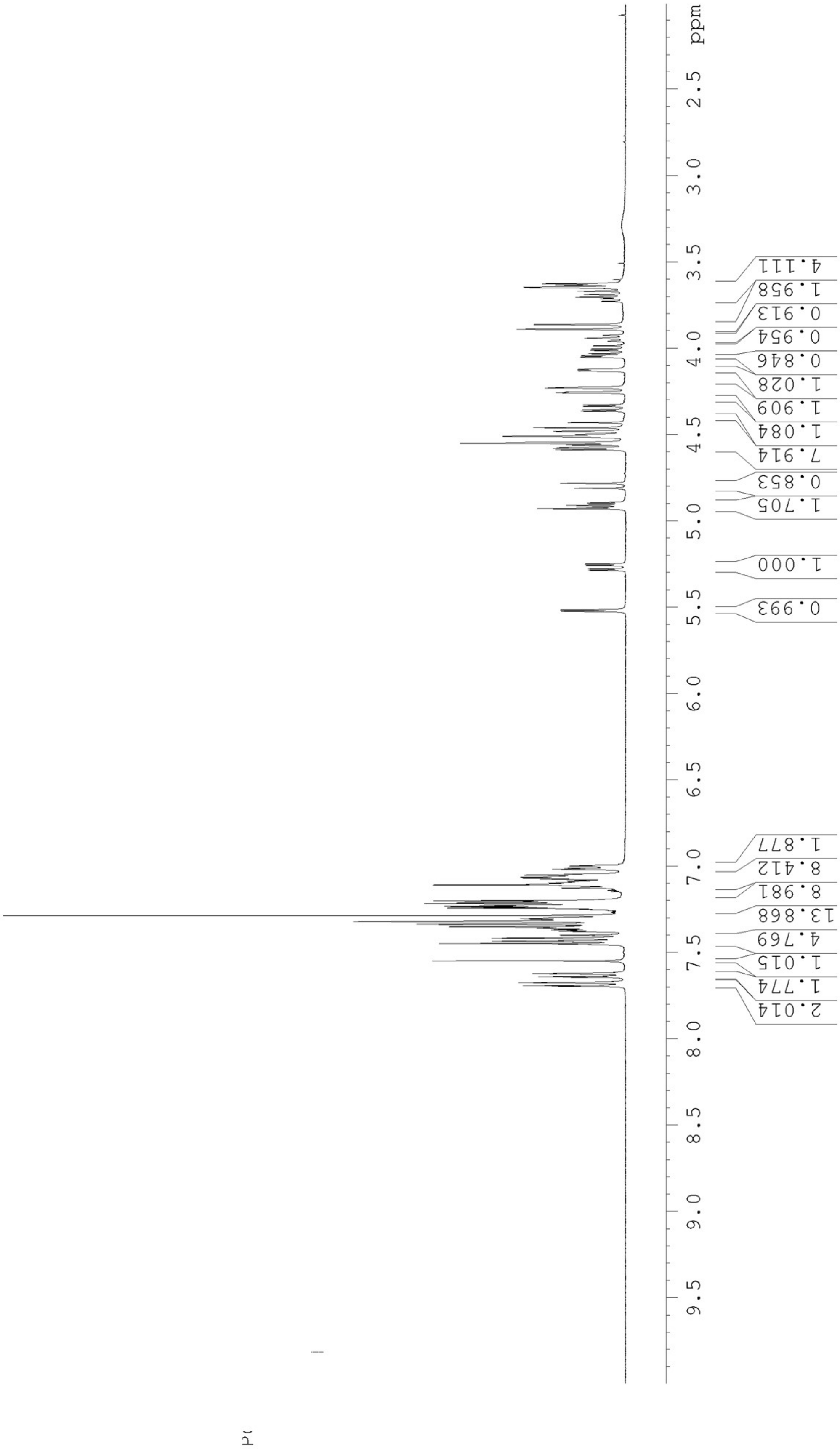

Compound **10** ^{13}C-NMR

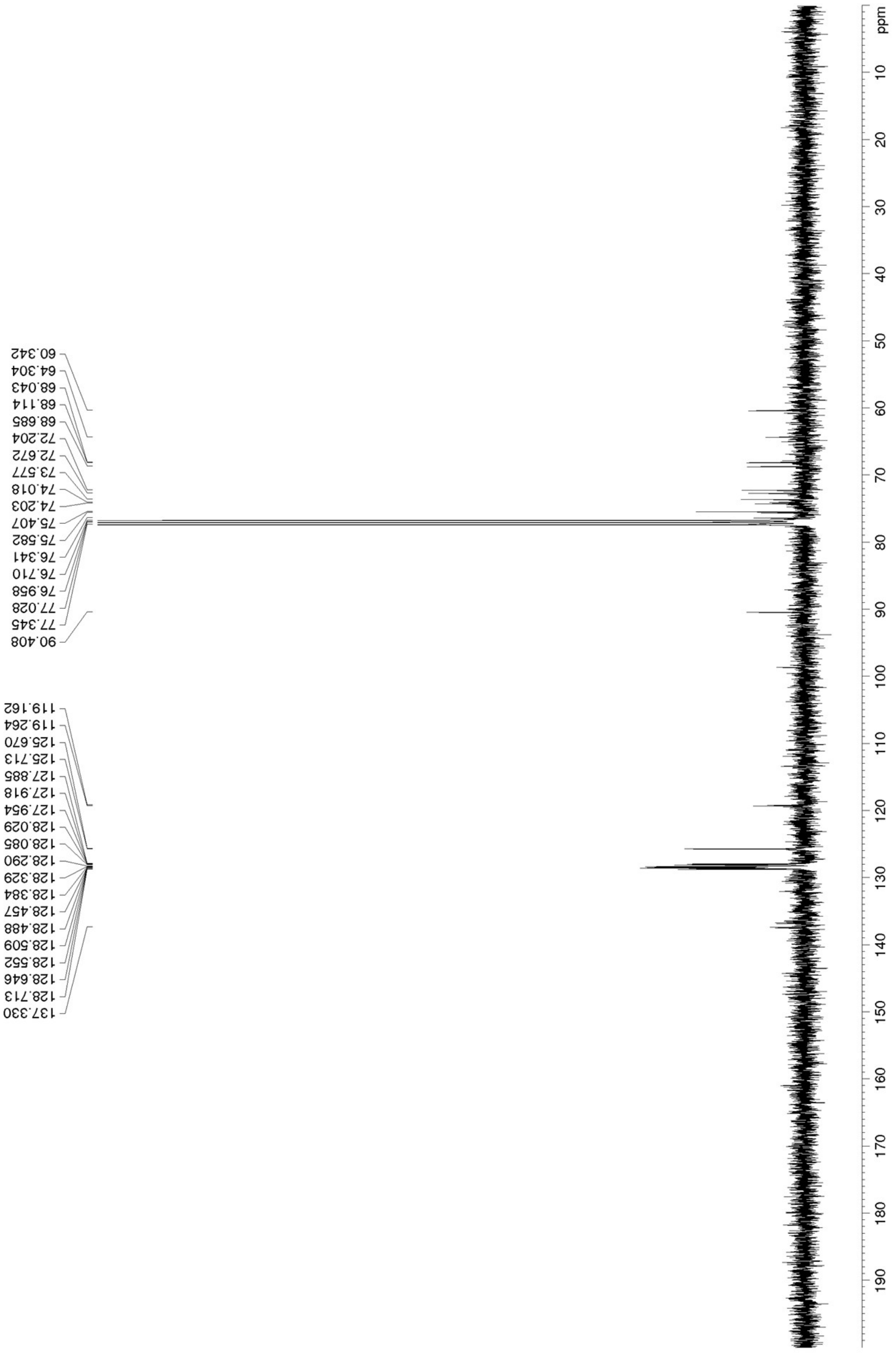

Compound **11** ^{1}H-NMR

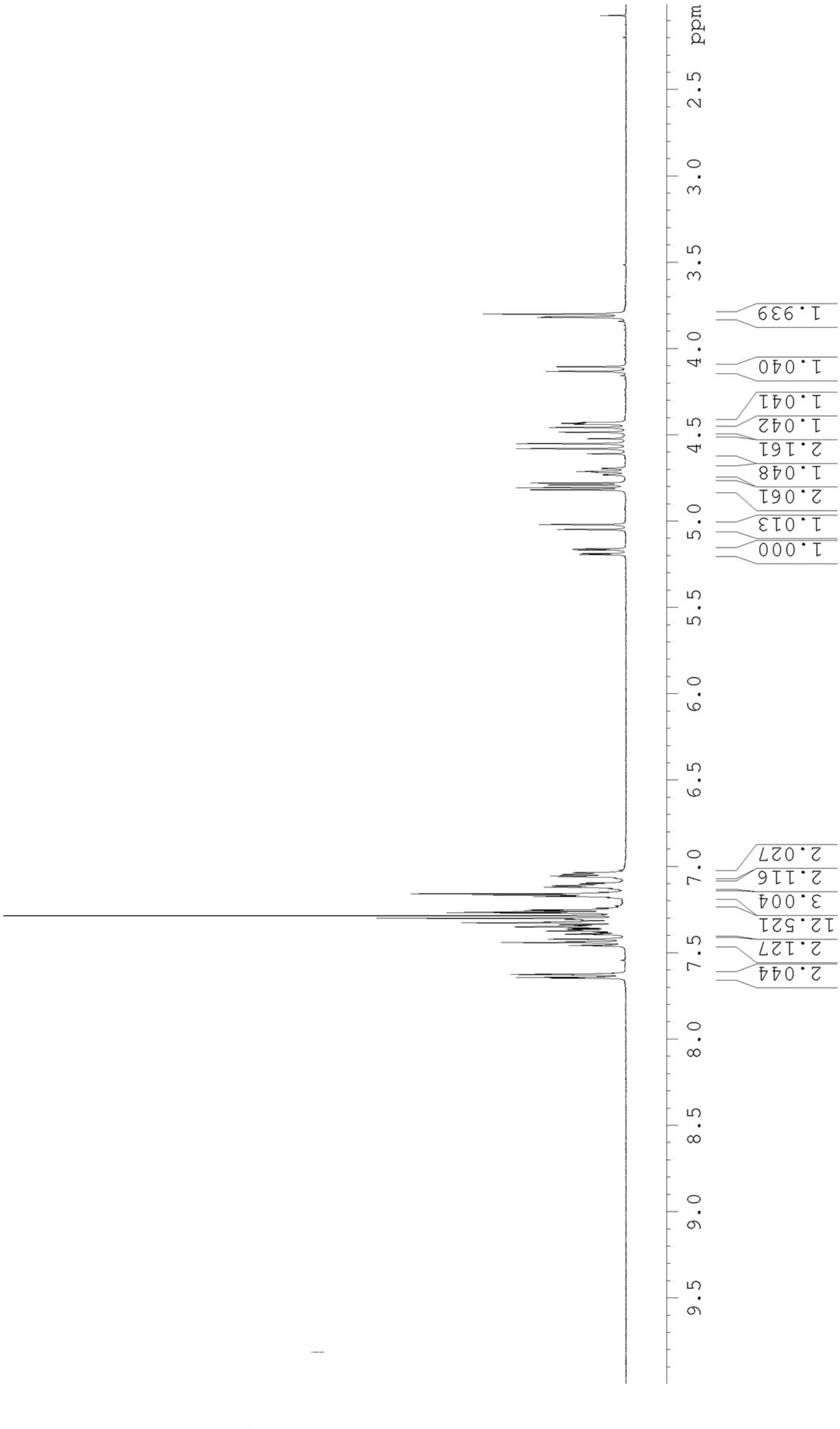

Compound **11** ^{13}C-NMR

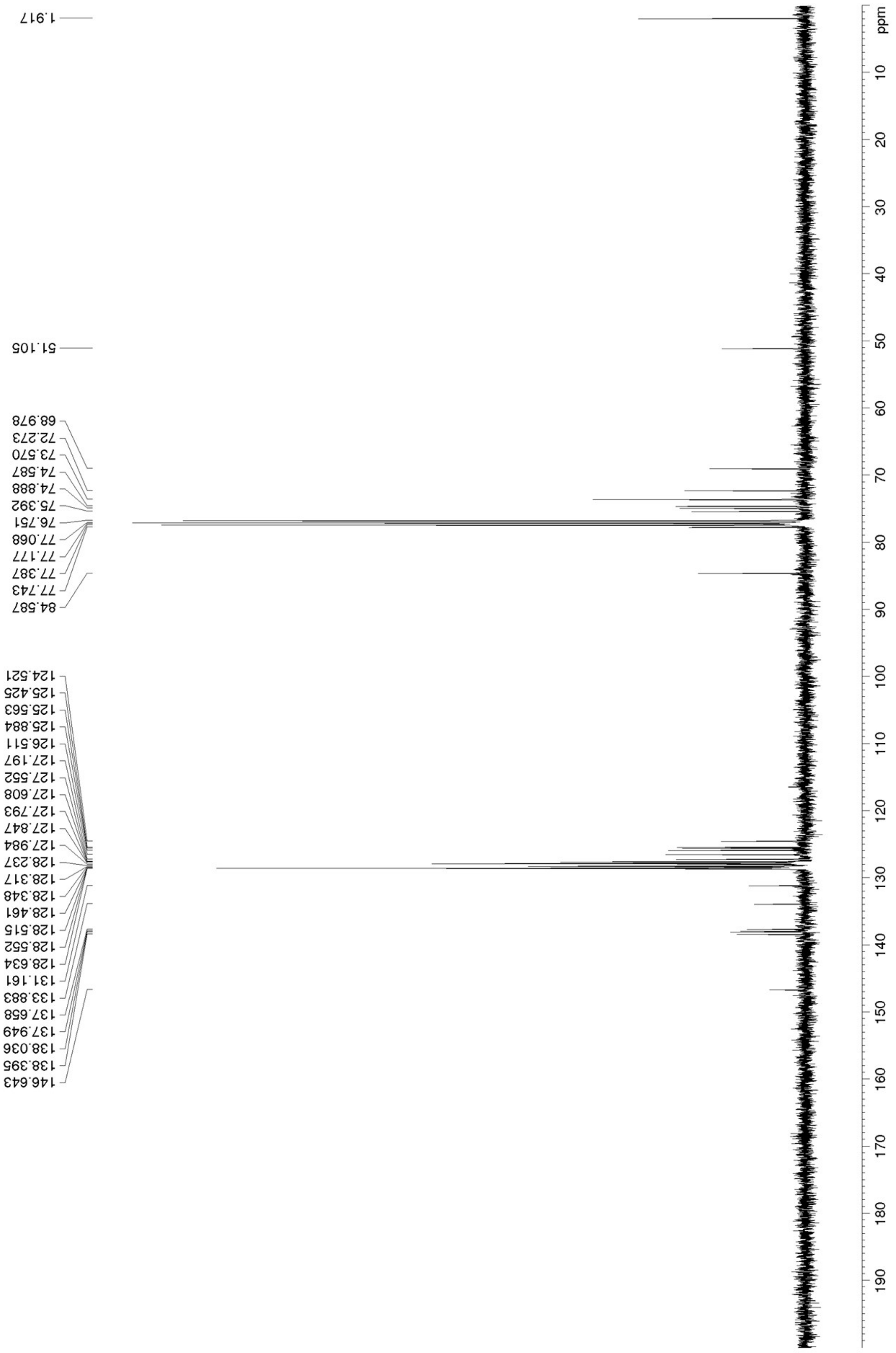

Compound **12** ^{1}H-NMR

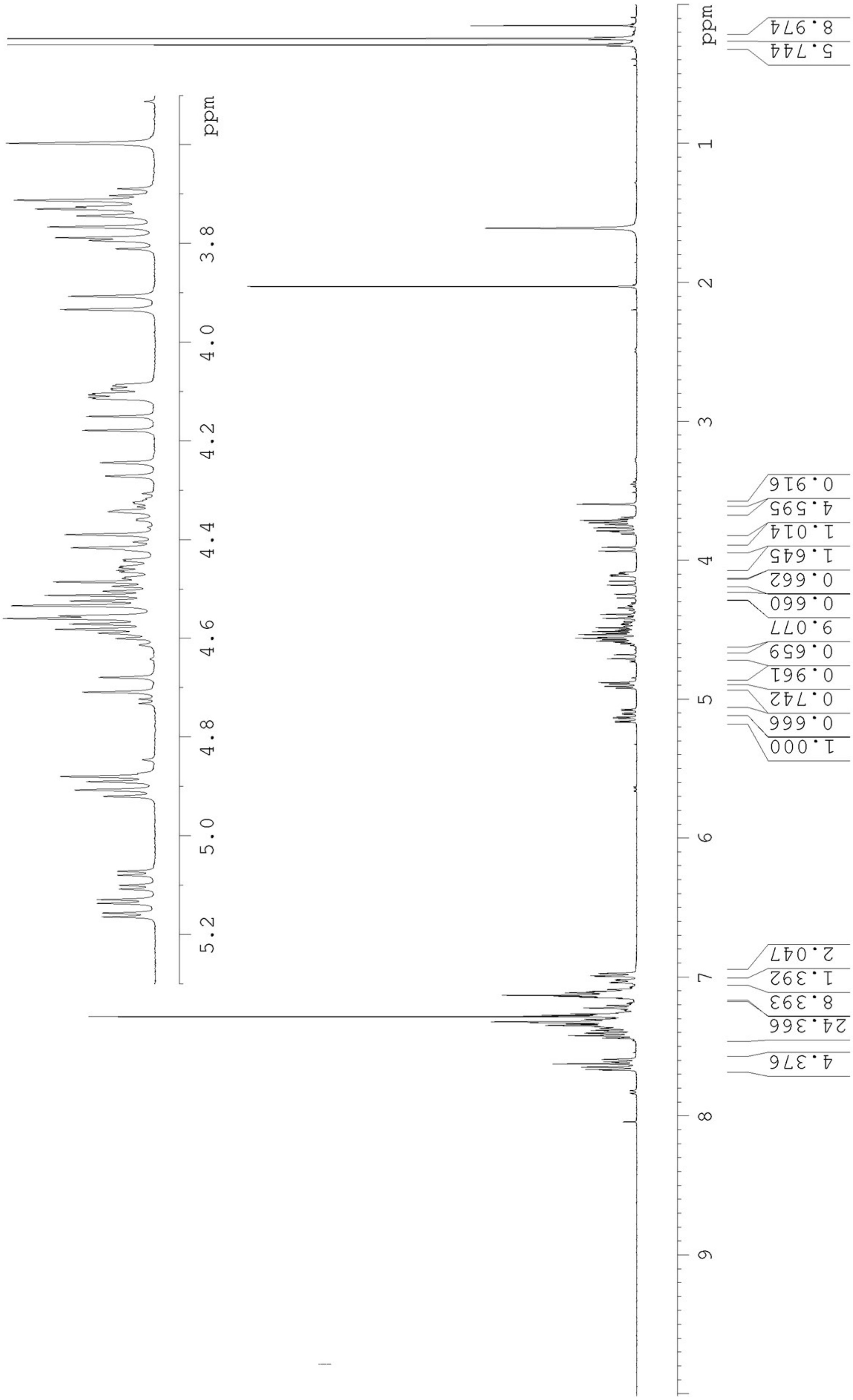

Compound **12** ^{13}C-NMR

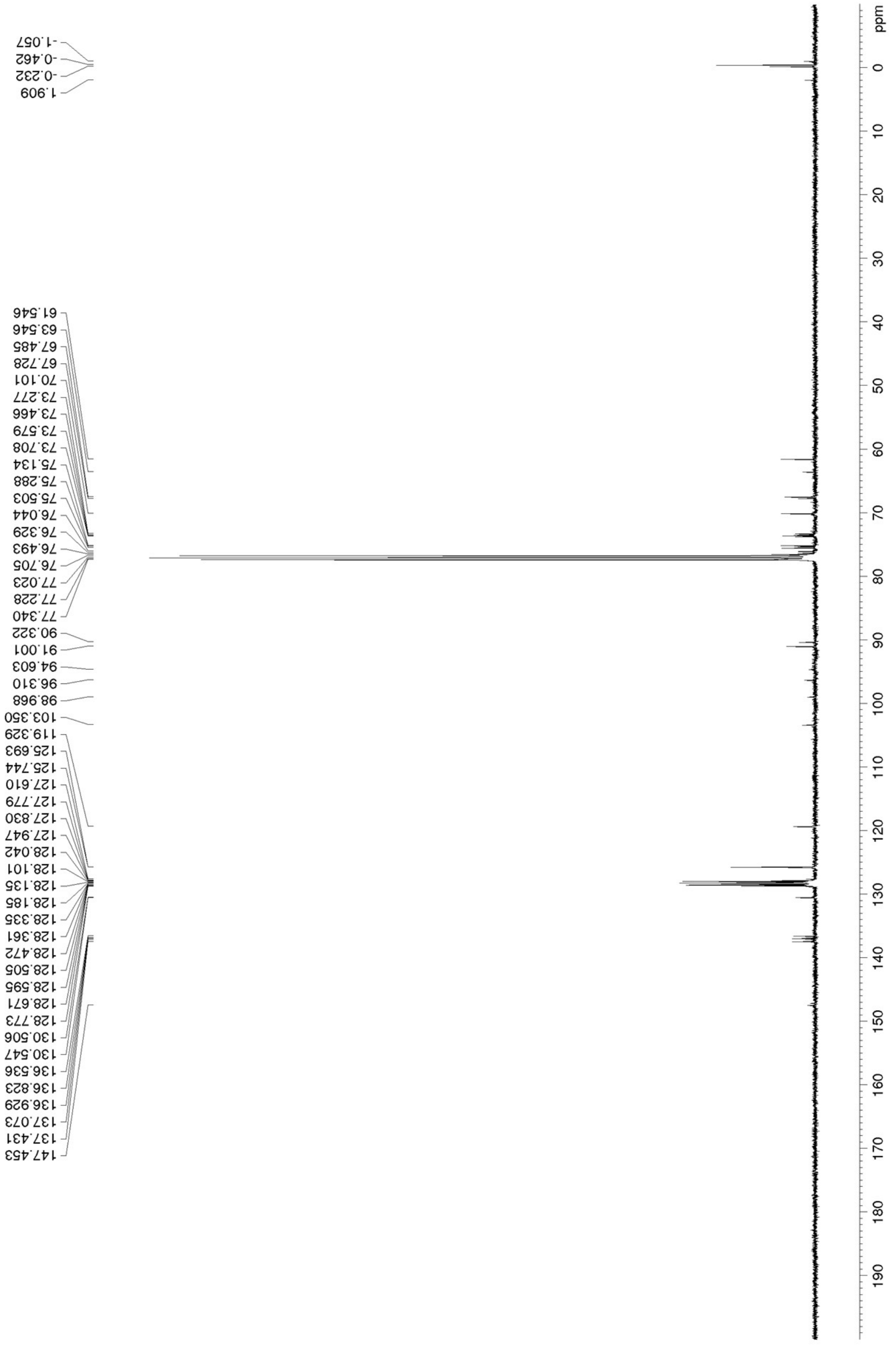

Compound **13** ^{1}H-NMR

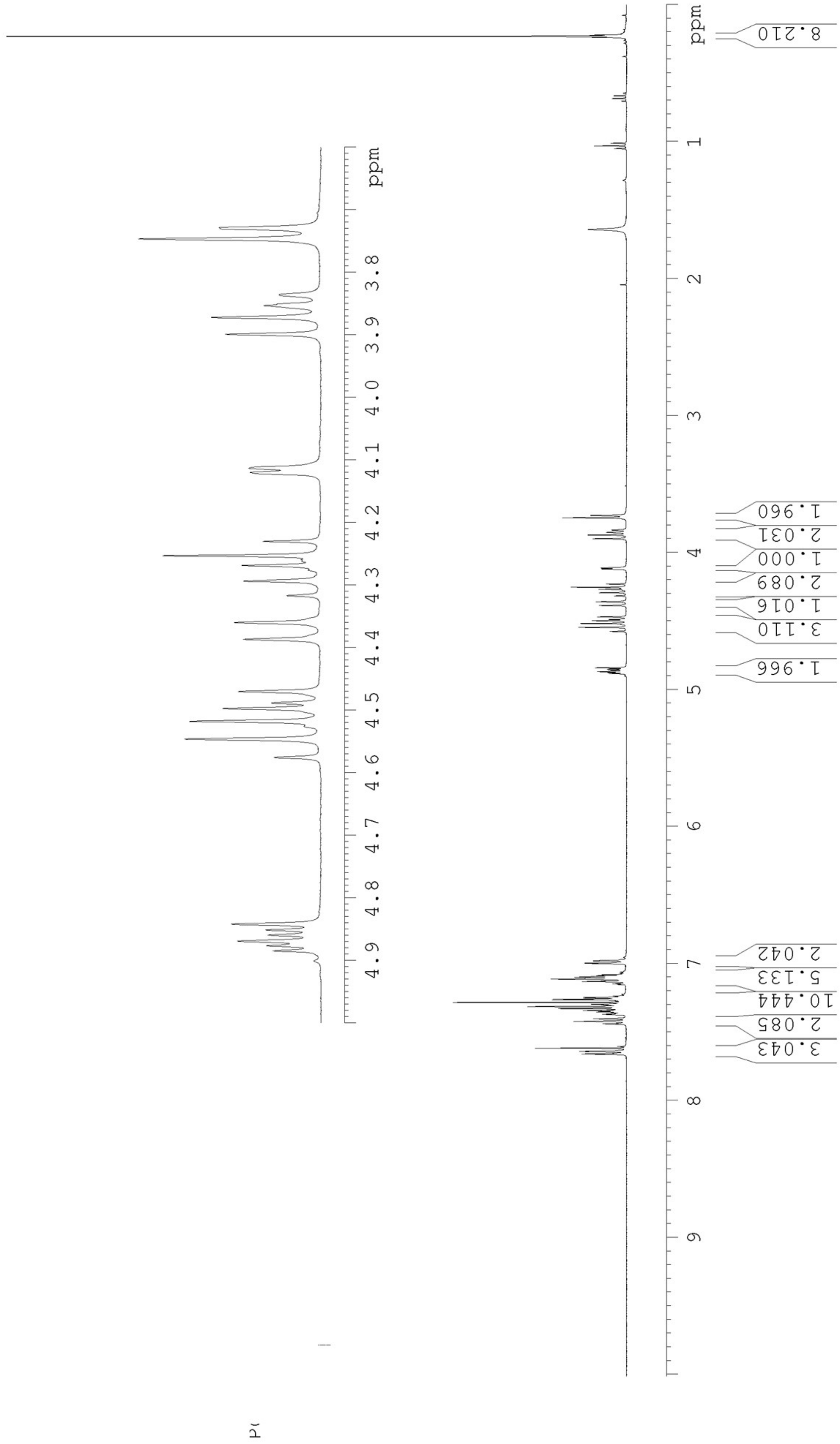

Compound **13** ^{13}C-NMR

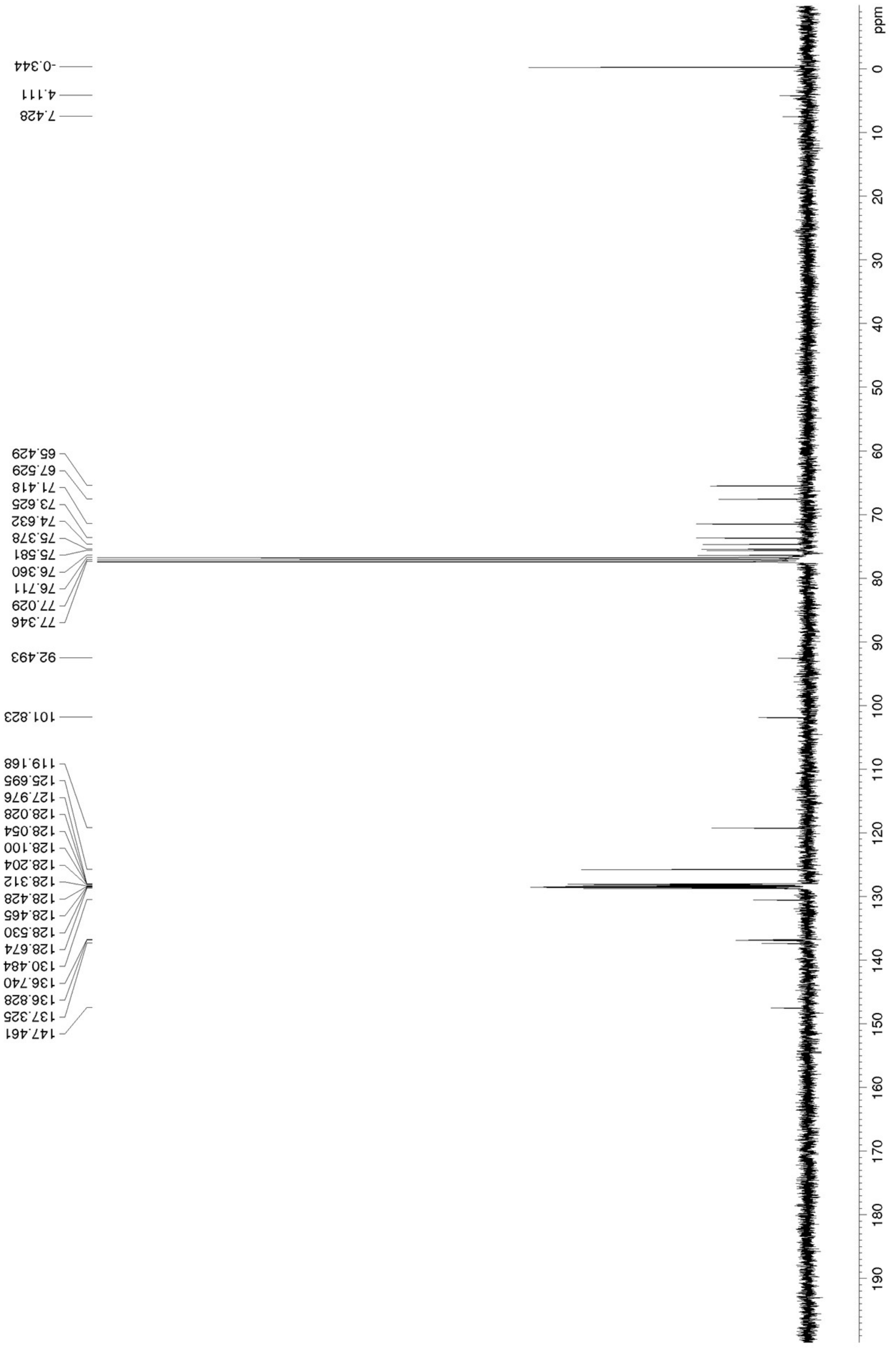

Compound **14** ^{1}H-NMR

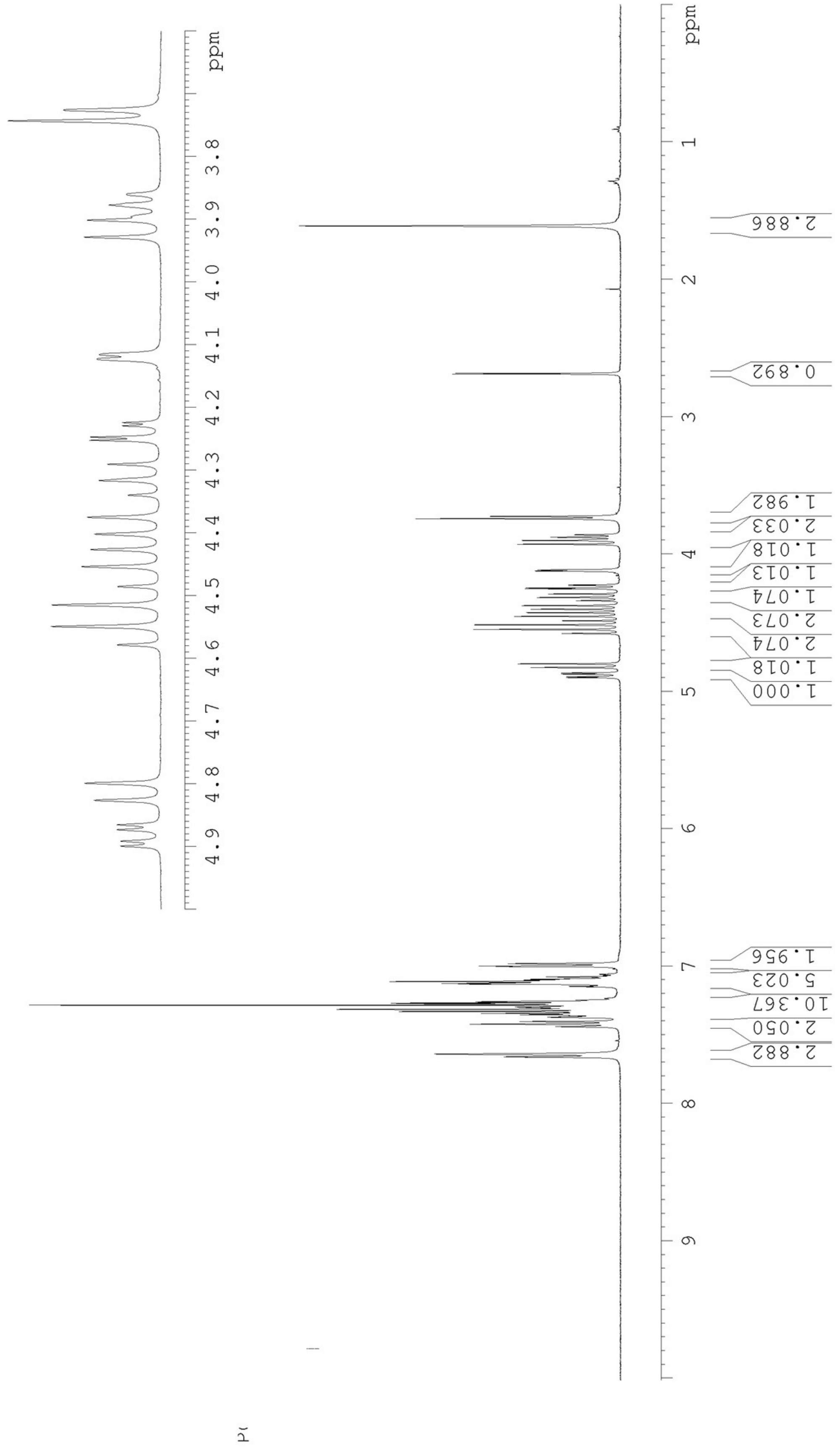

Compound **14** ^{13}C-NMR

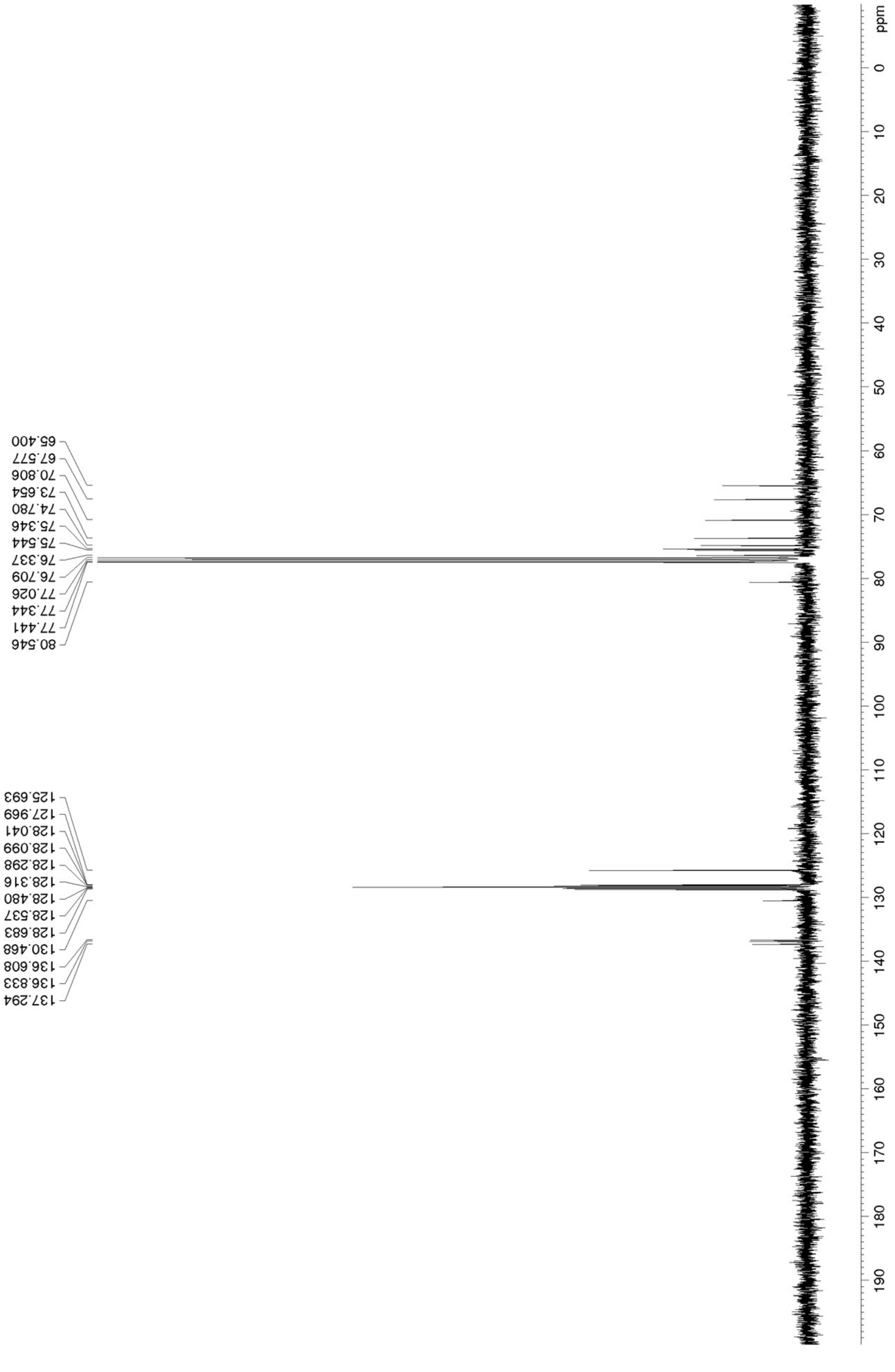

Compound **15a** ^{1}H-NMR

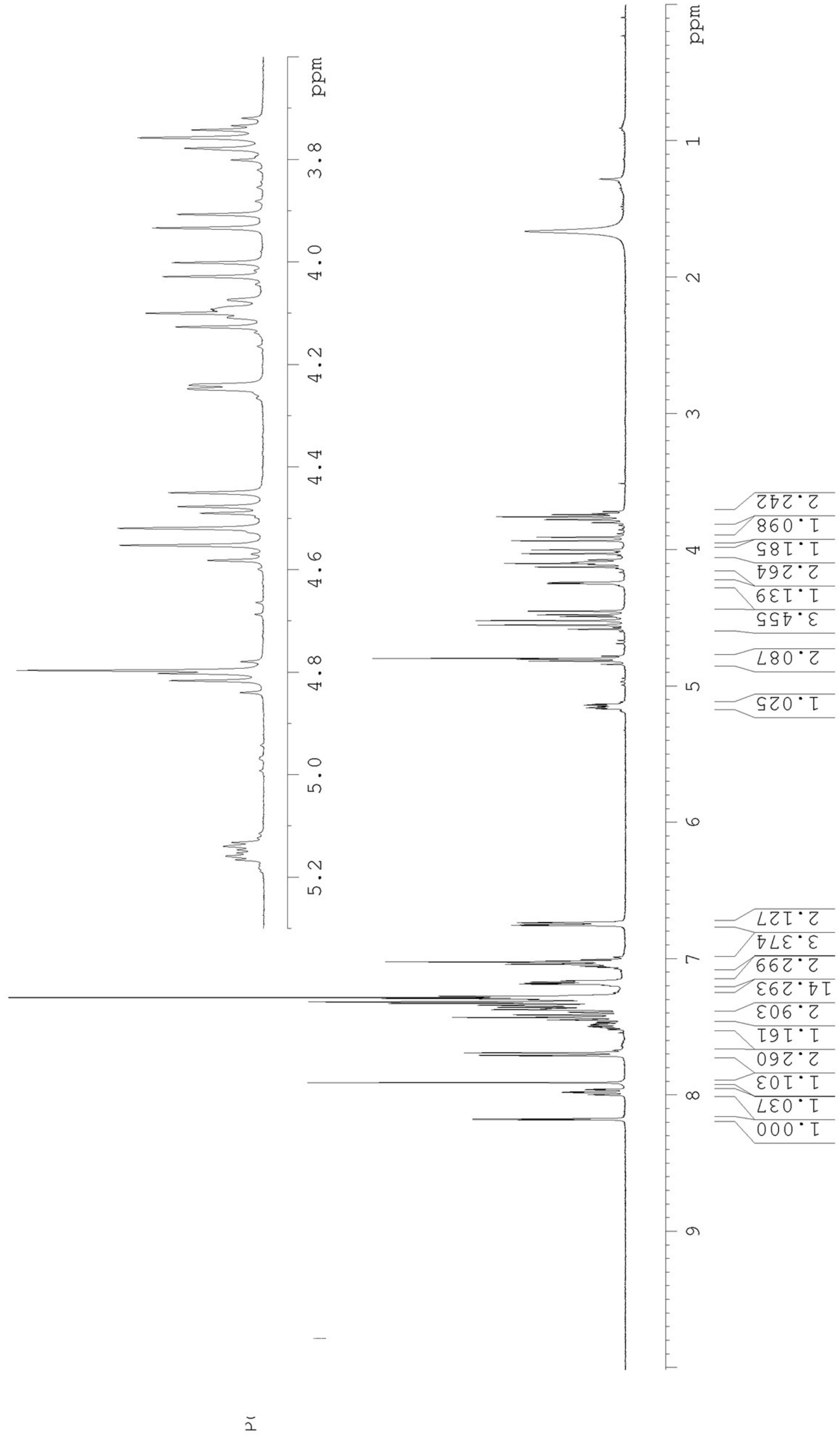

Compound **15a** ^{13}C-NMR

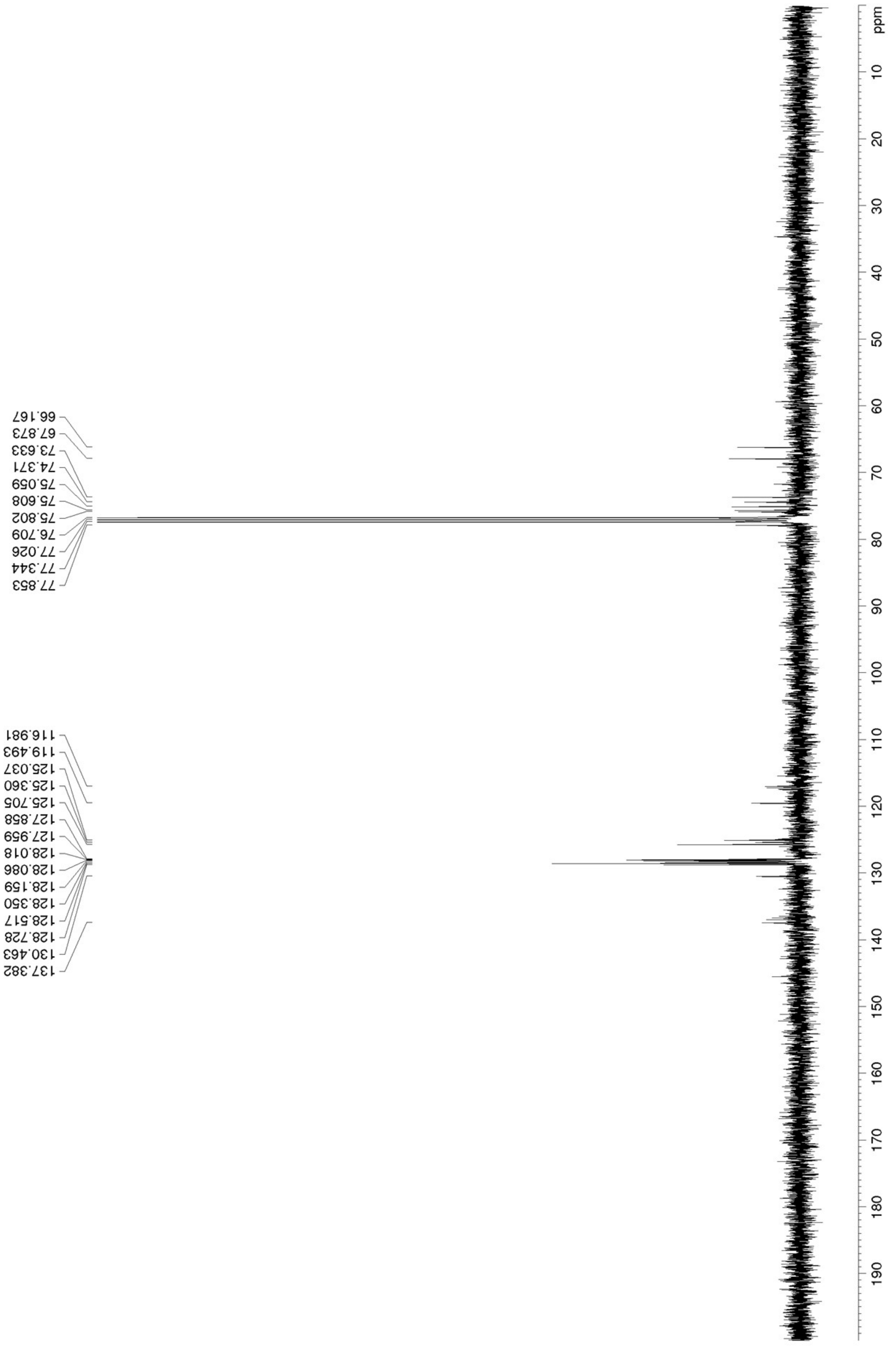

Compound **15b** ^{1}H-NMR

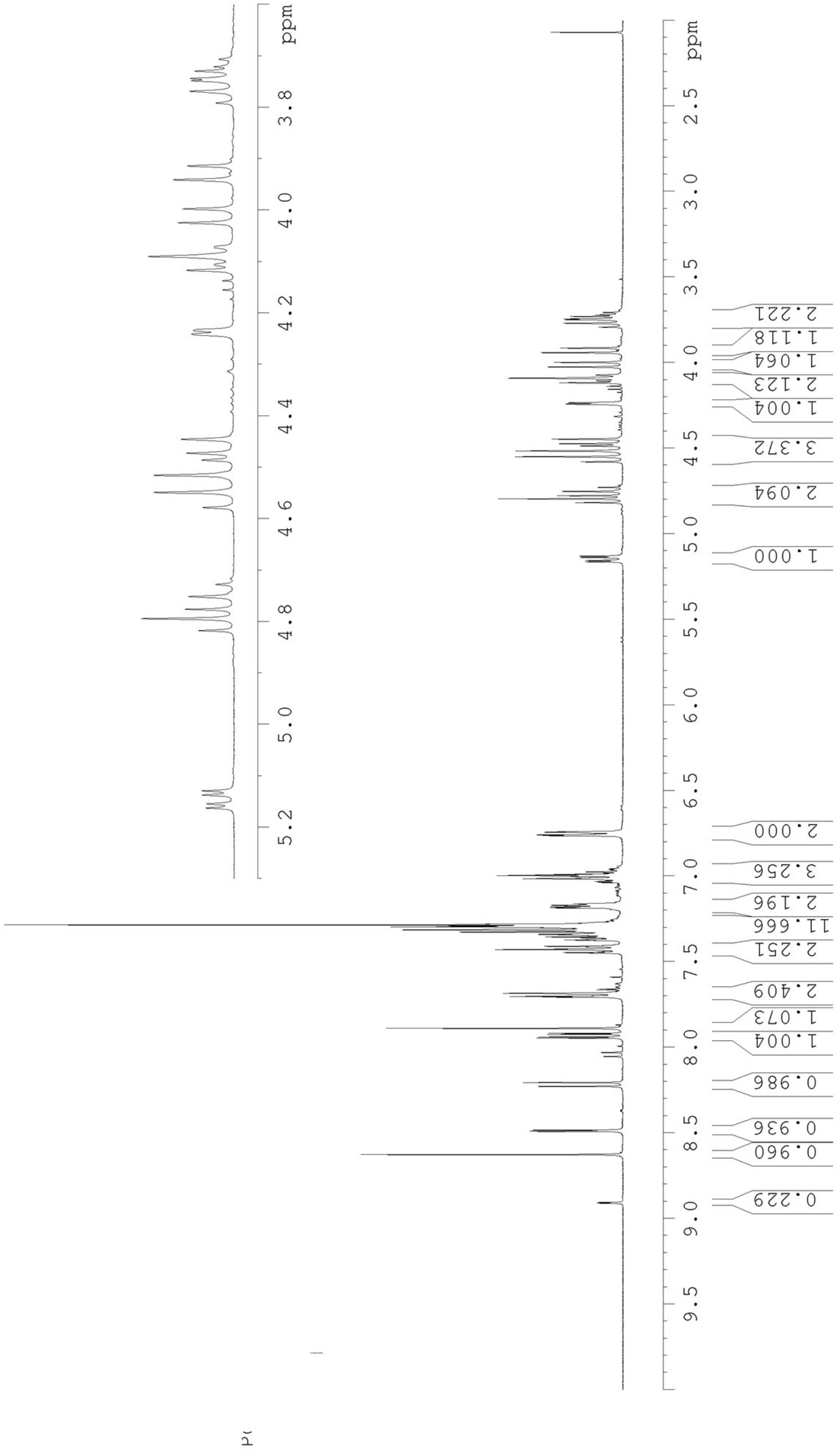

Compound **15b** ^{13}C-NMR

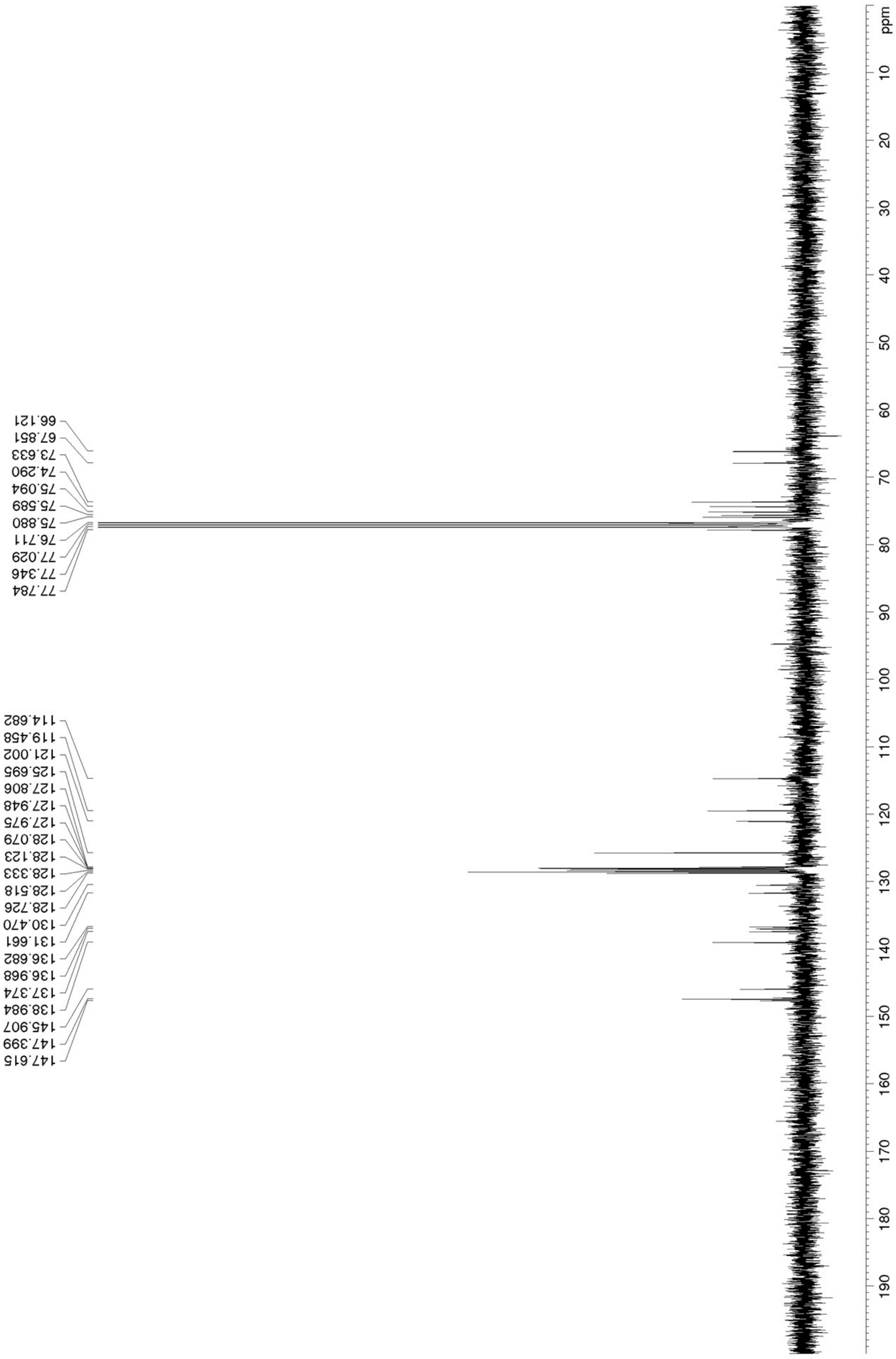

Compound **16a** ^{1}H-NMR

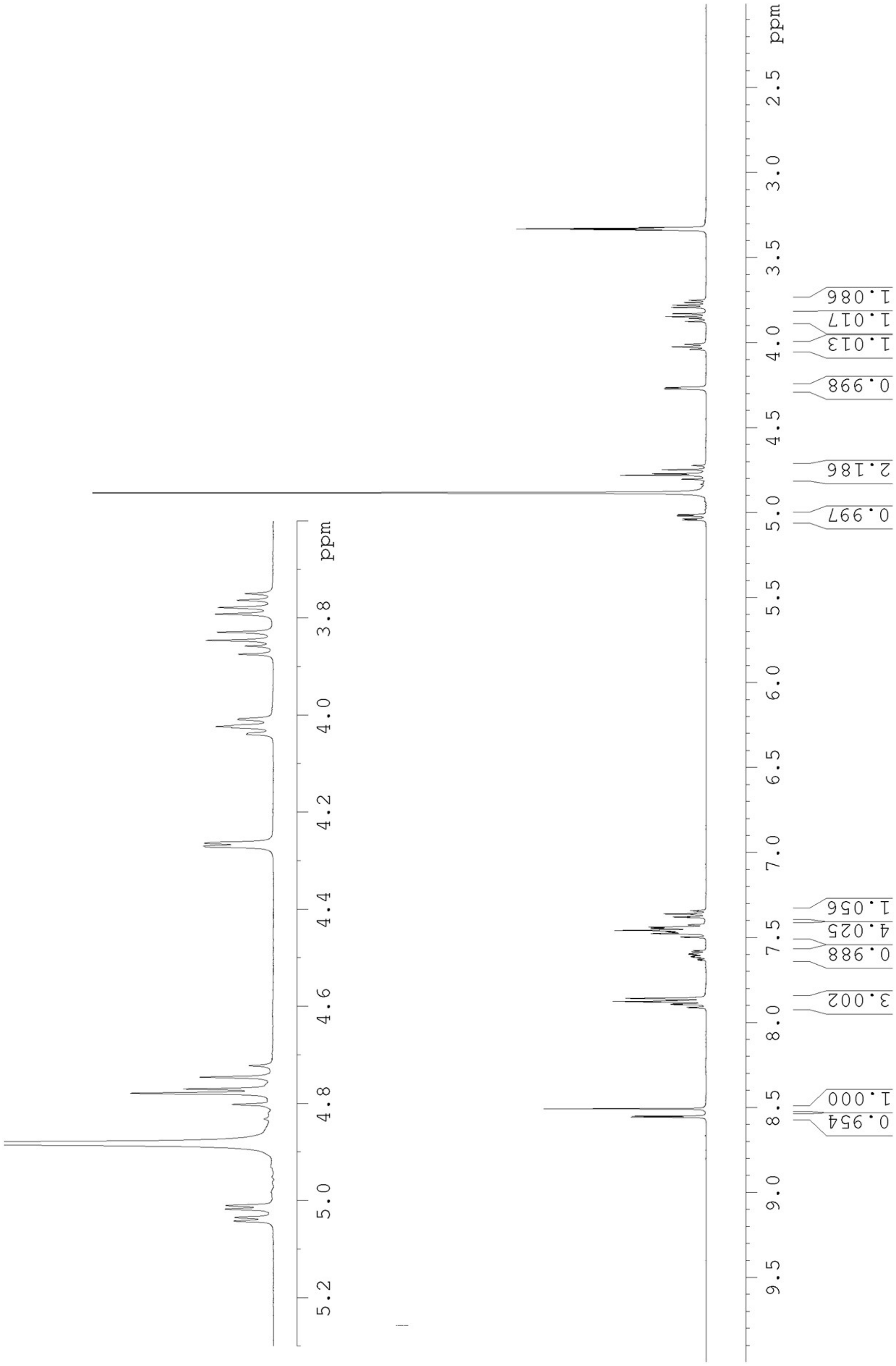

Compound **16a** ^{13}C-NMR

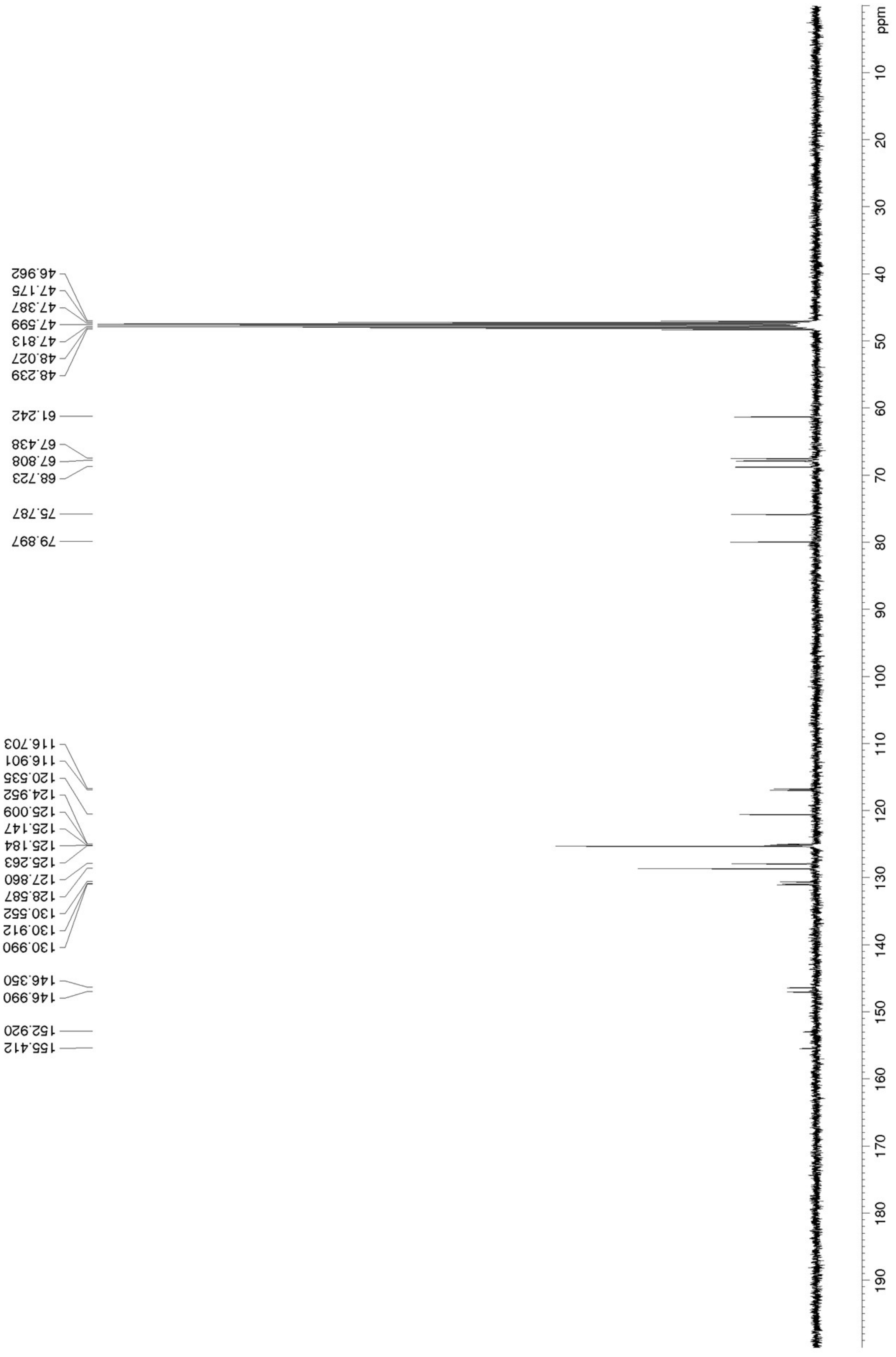

Compound **16b** ^{1}H-NMR

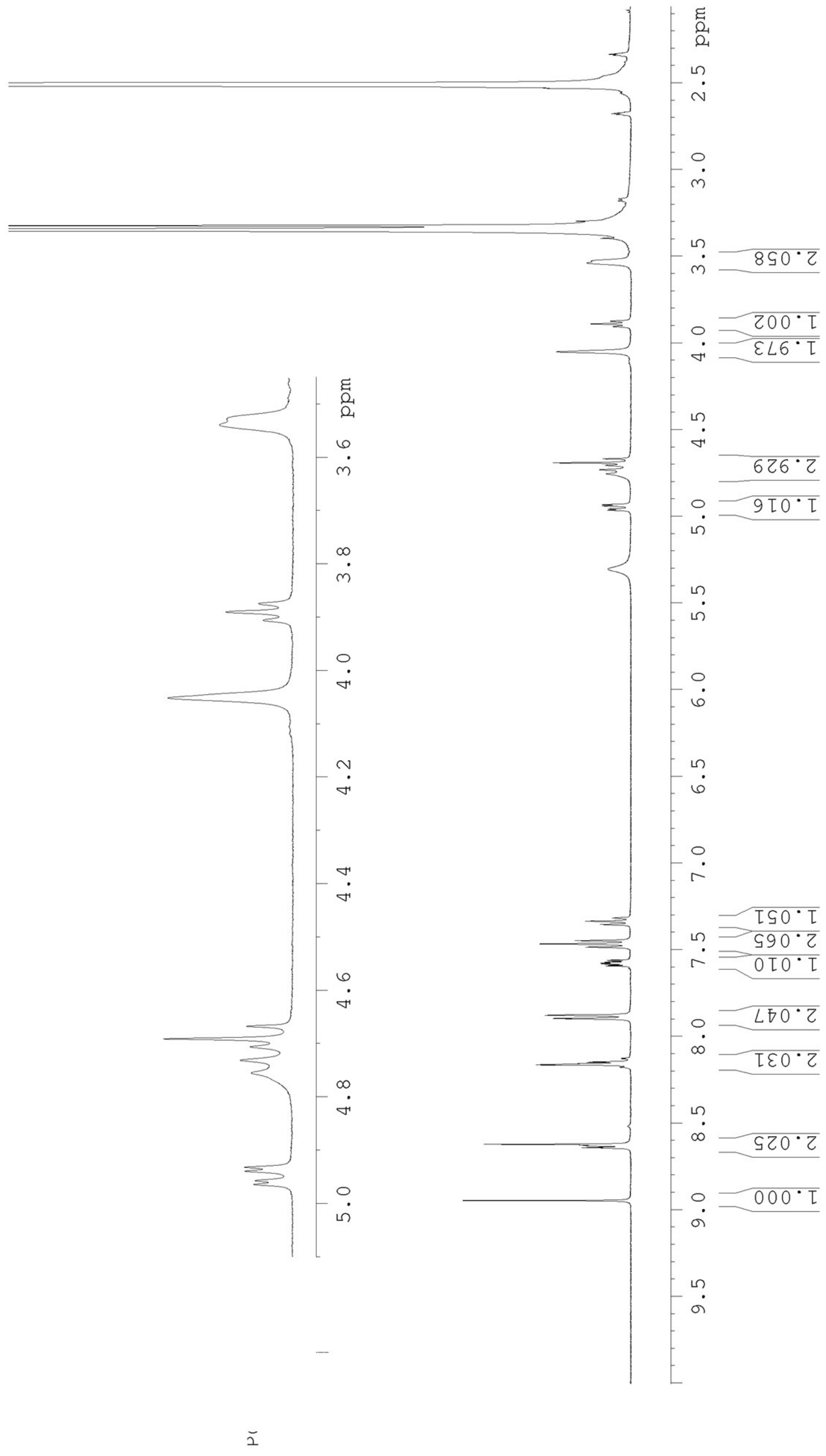

Compound **16b** ^{13}C-NMR

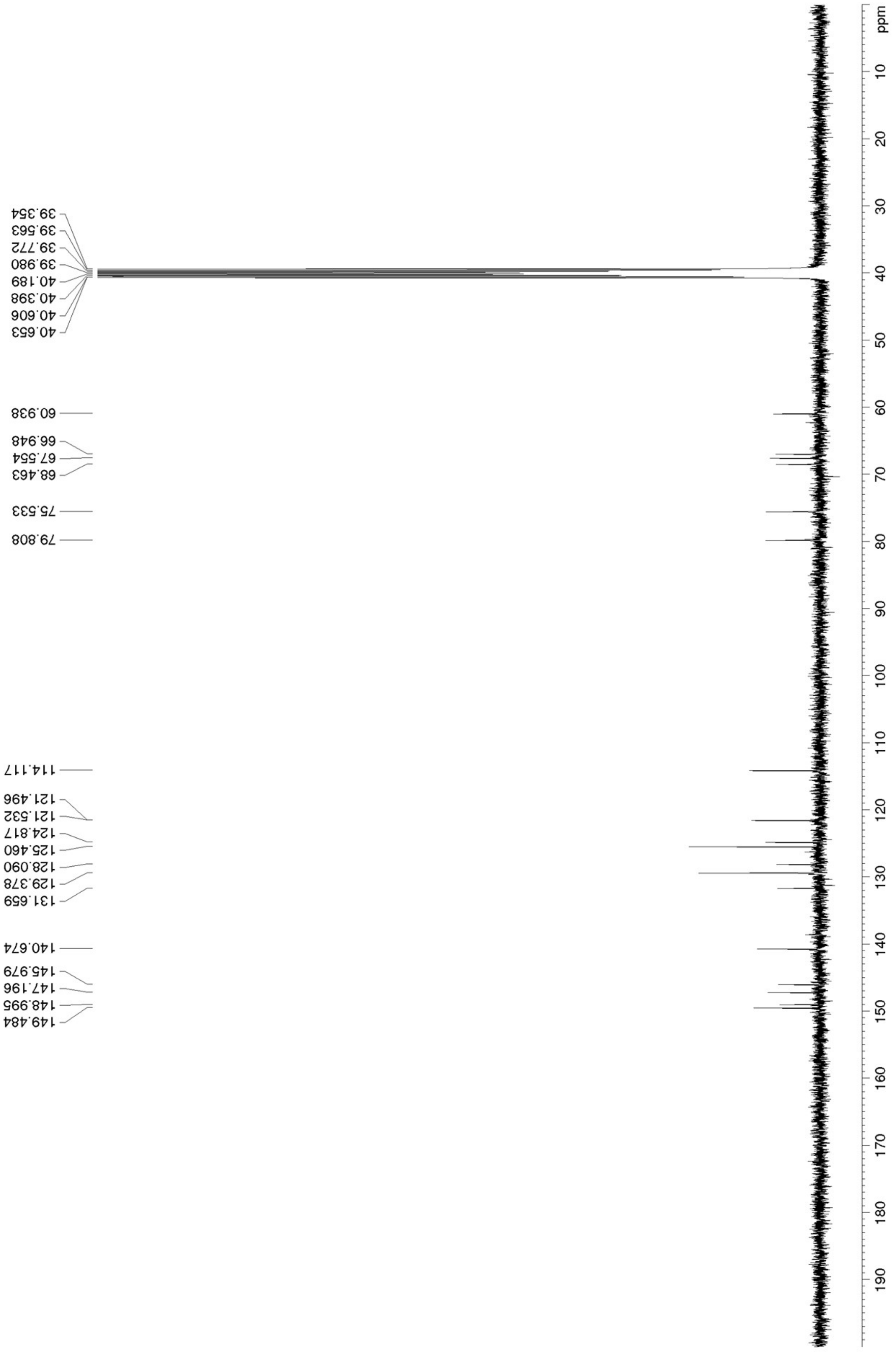

Compound **17** ^{1}H-NMR

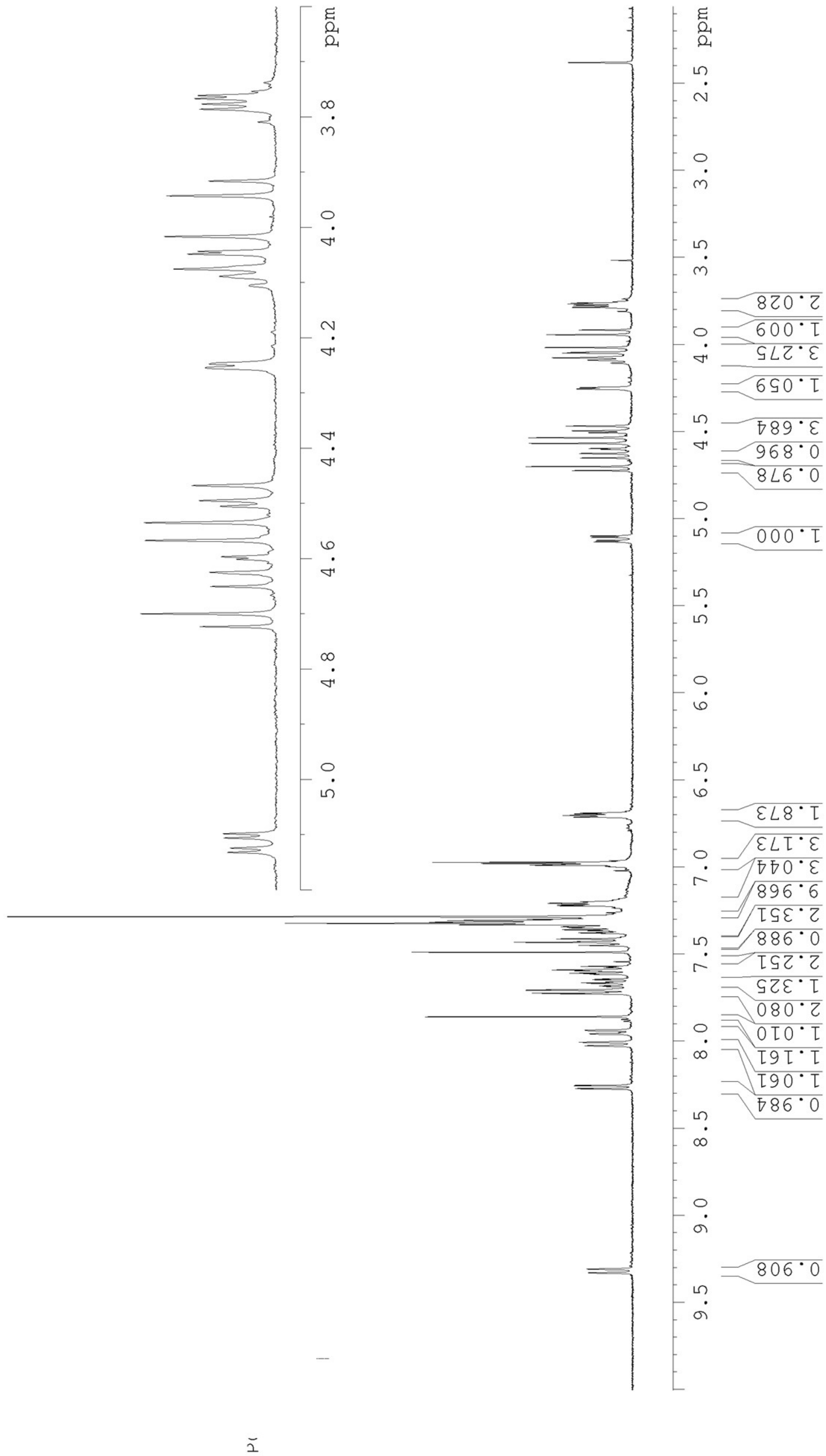

Compound **17** ^{13}C-NMR

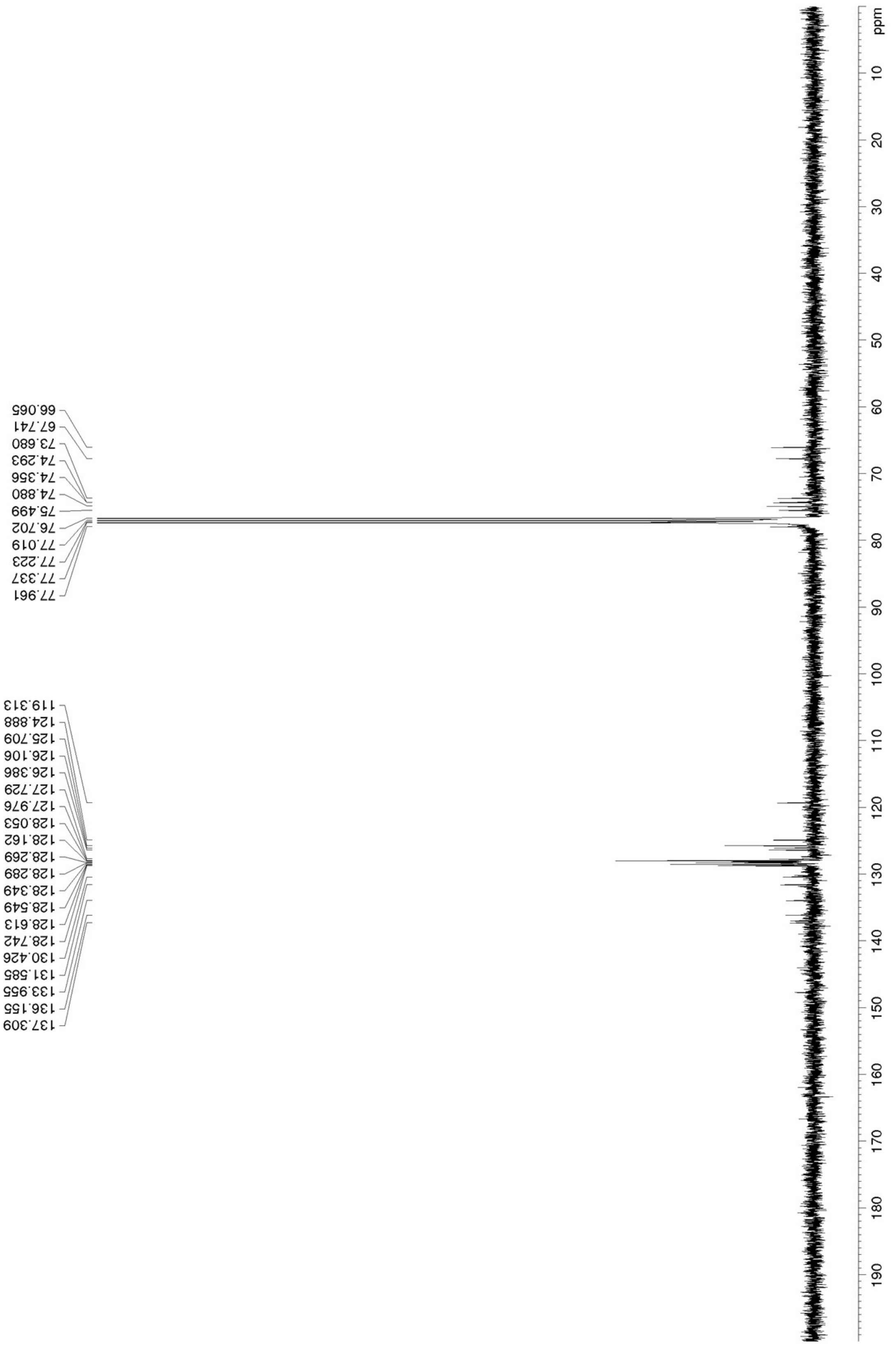

Compound **18** ^{1}H-NMR

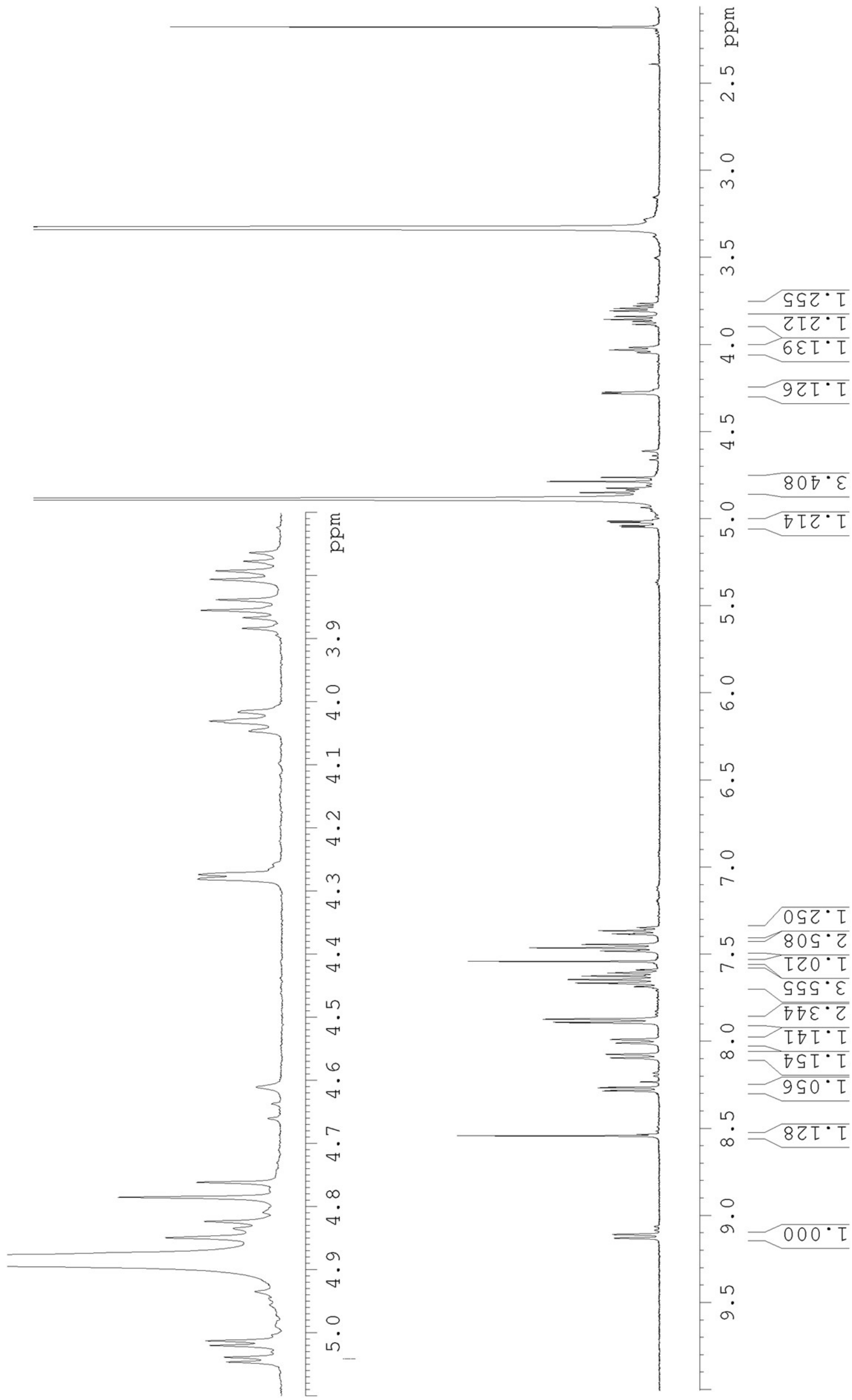

Compound **18** ^{13}C-NMR

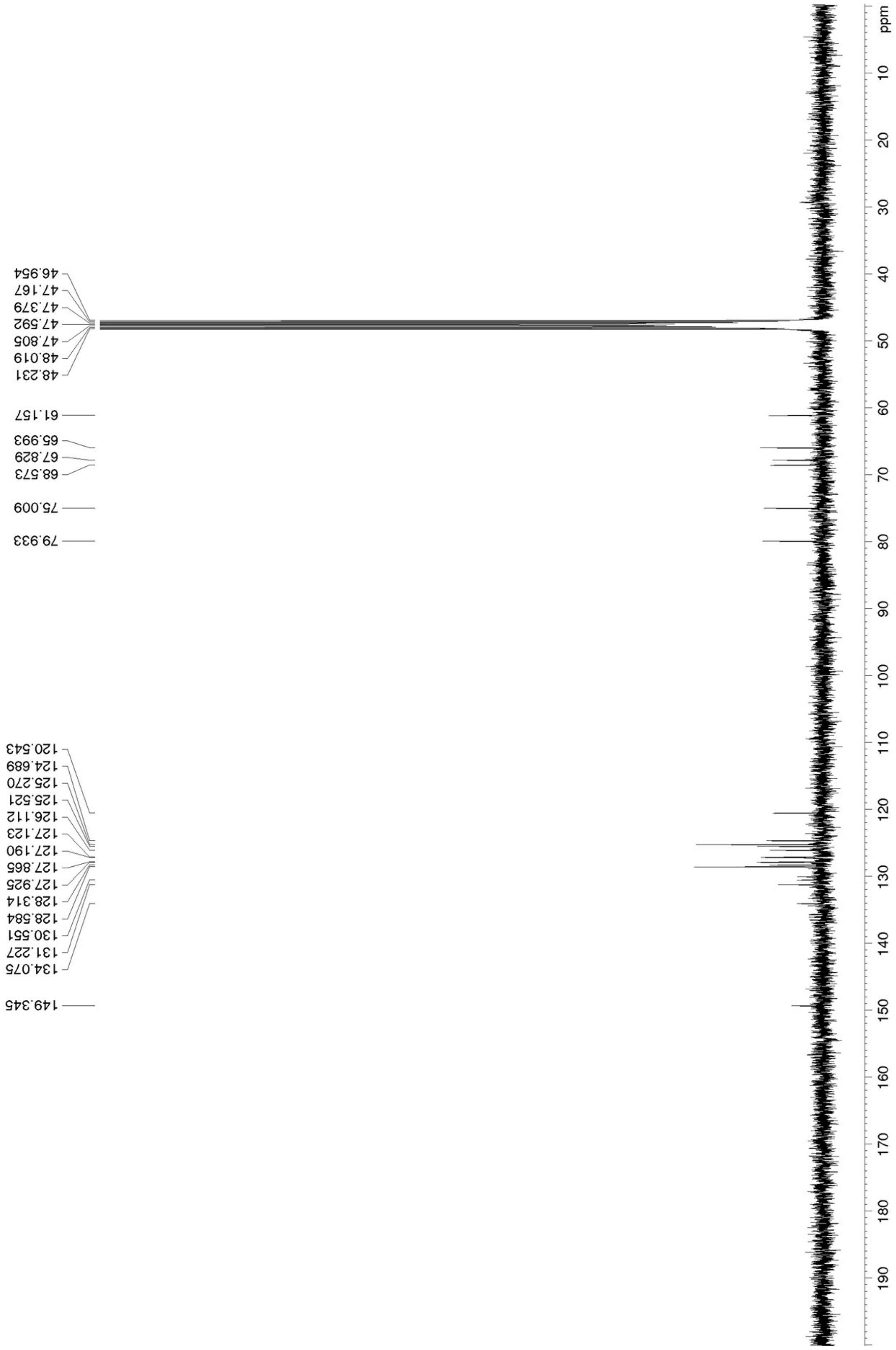

Compound **19** ^{1}H-NMR

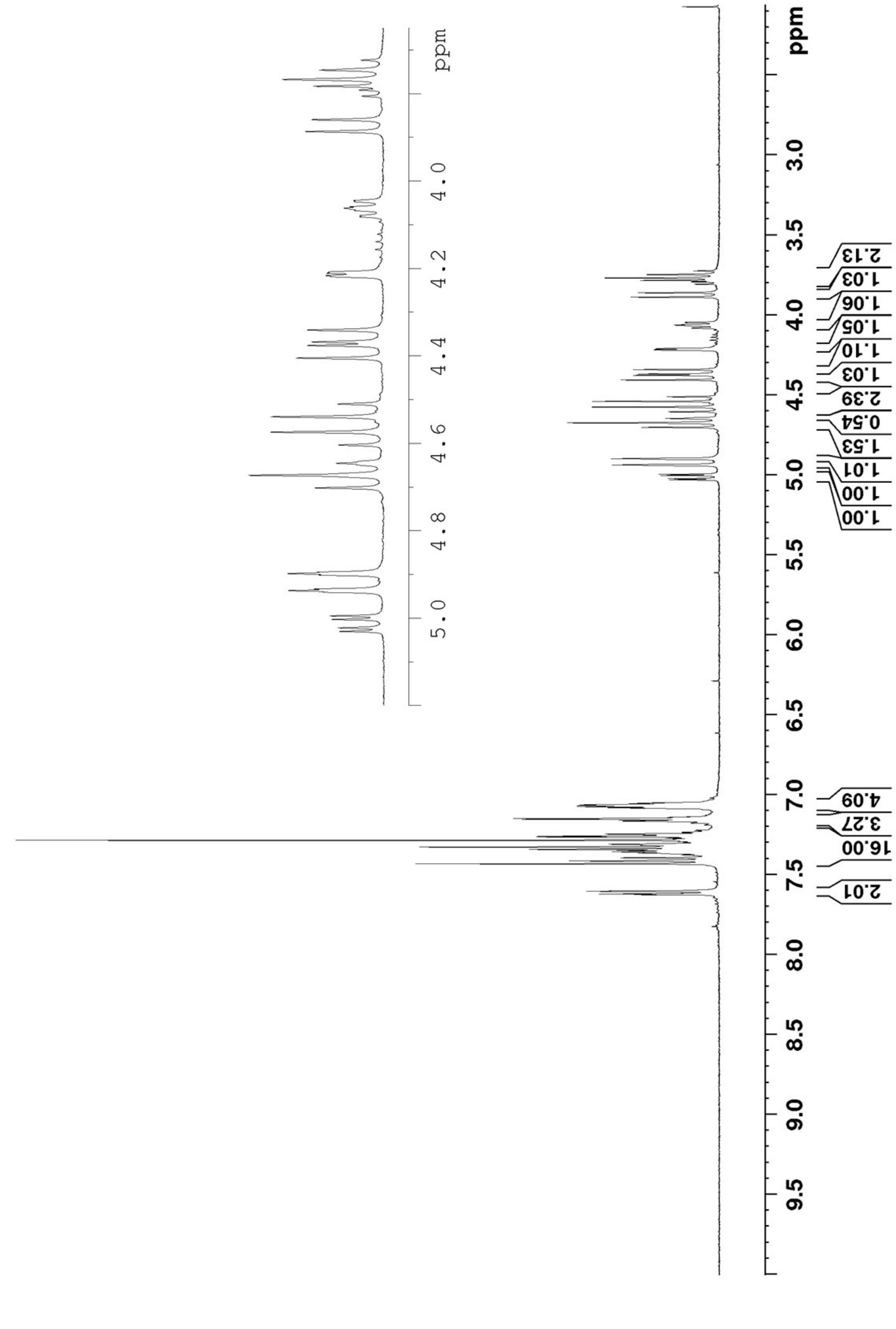

Compound **19** ^{13}C-NMR

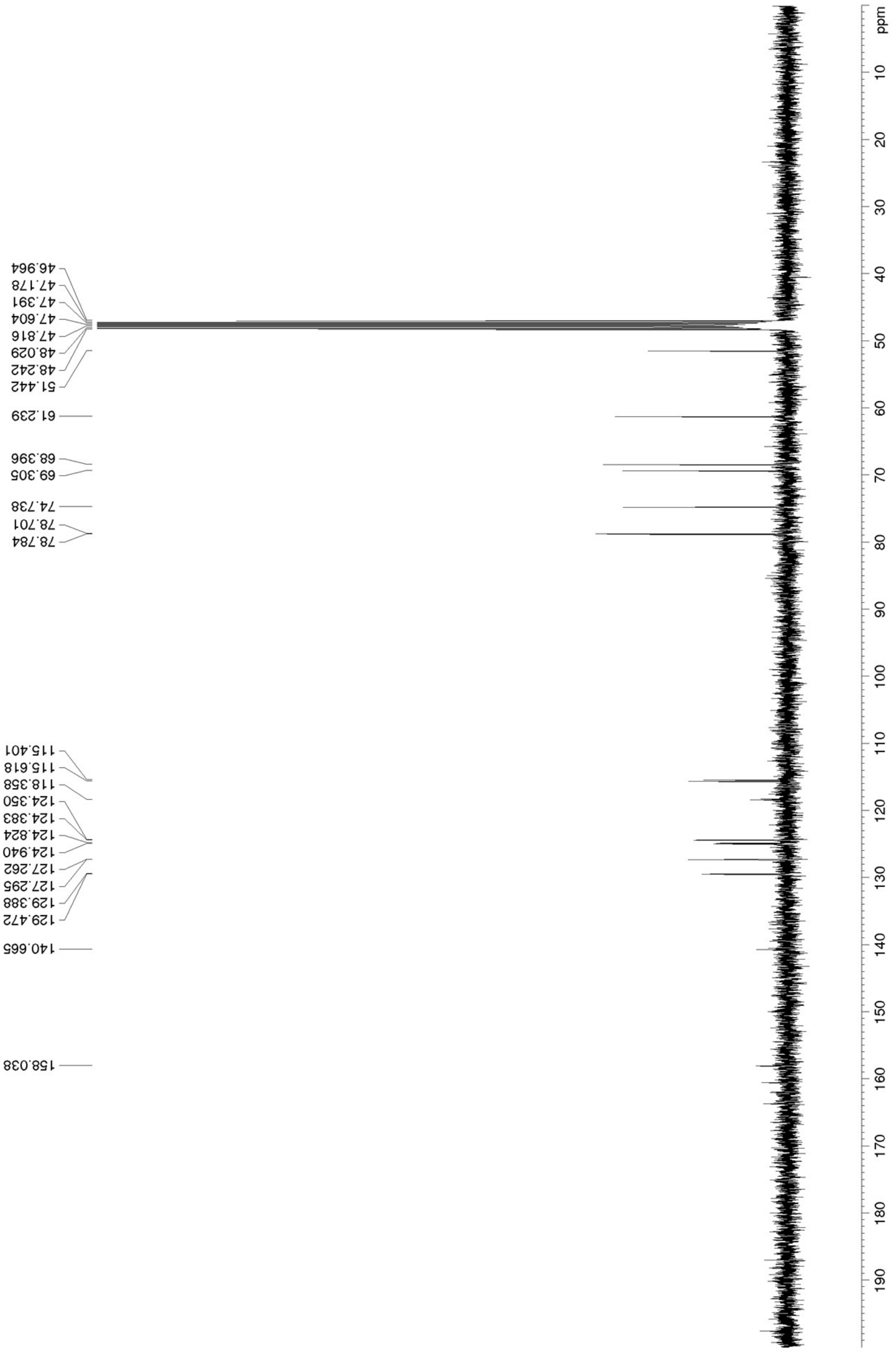

Compound **20** ^{1}H-NMR

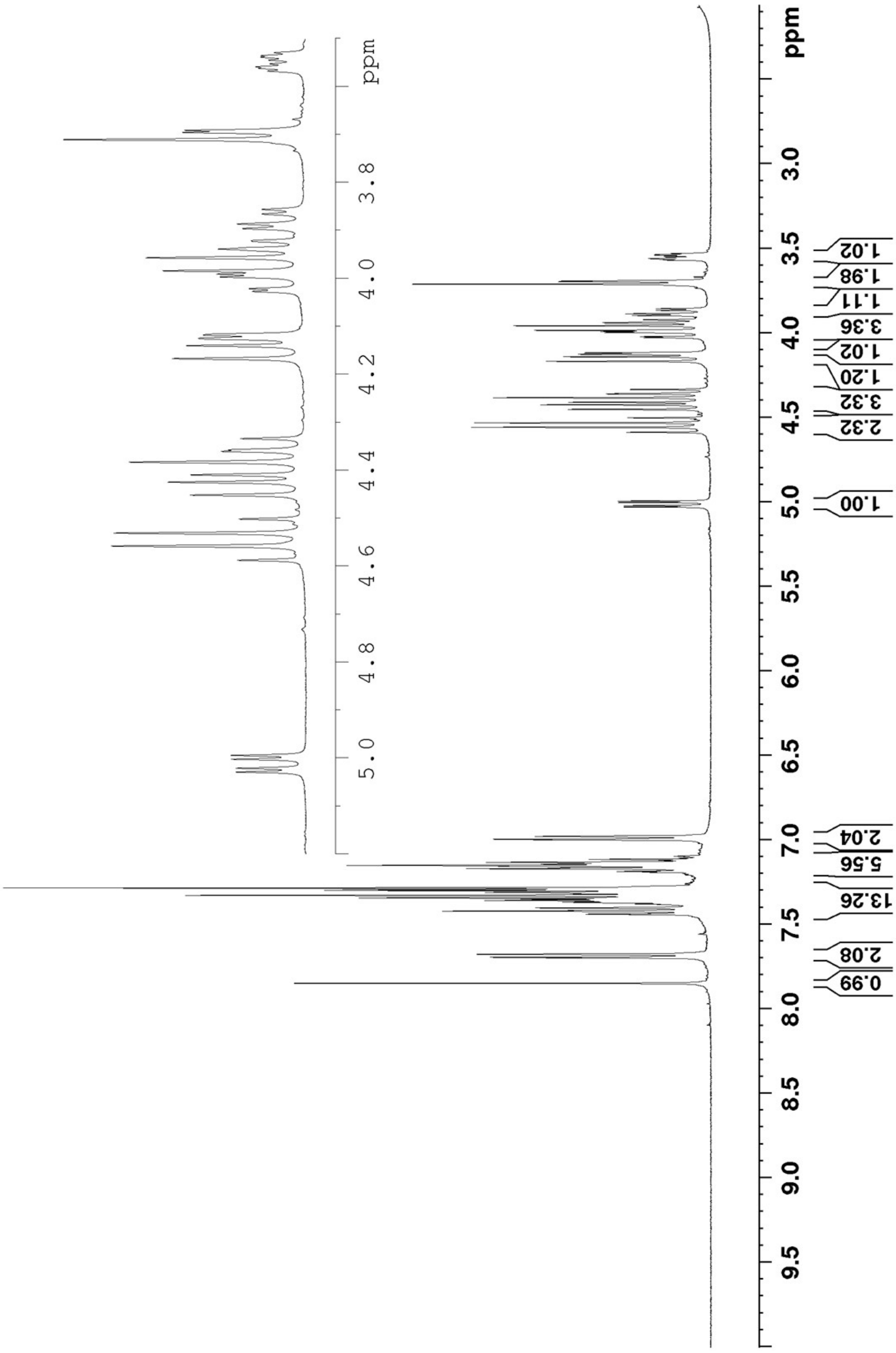

Compound **20** ^{13}C-NMR

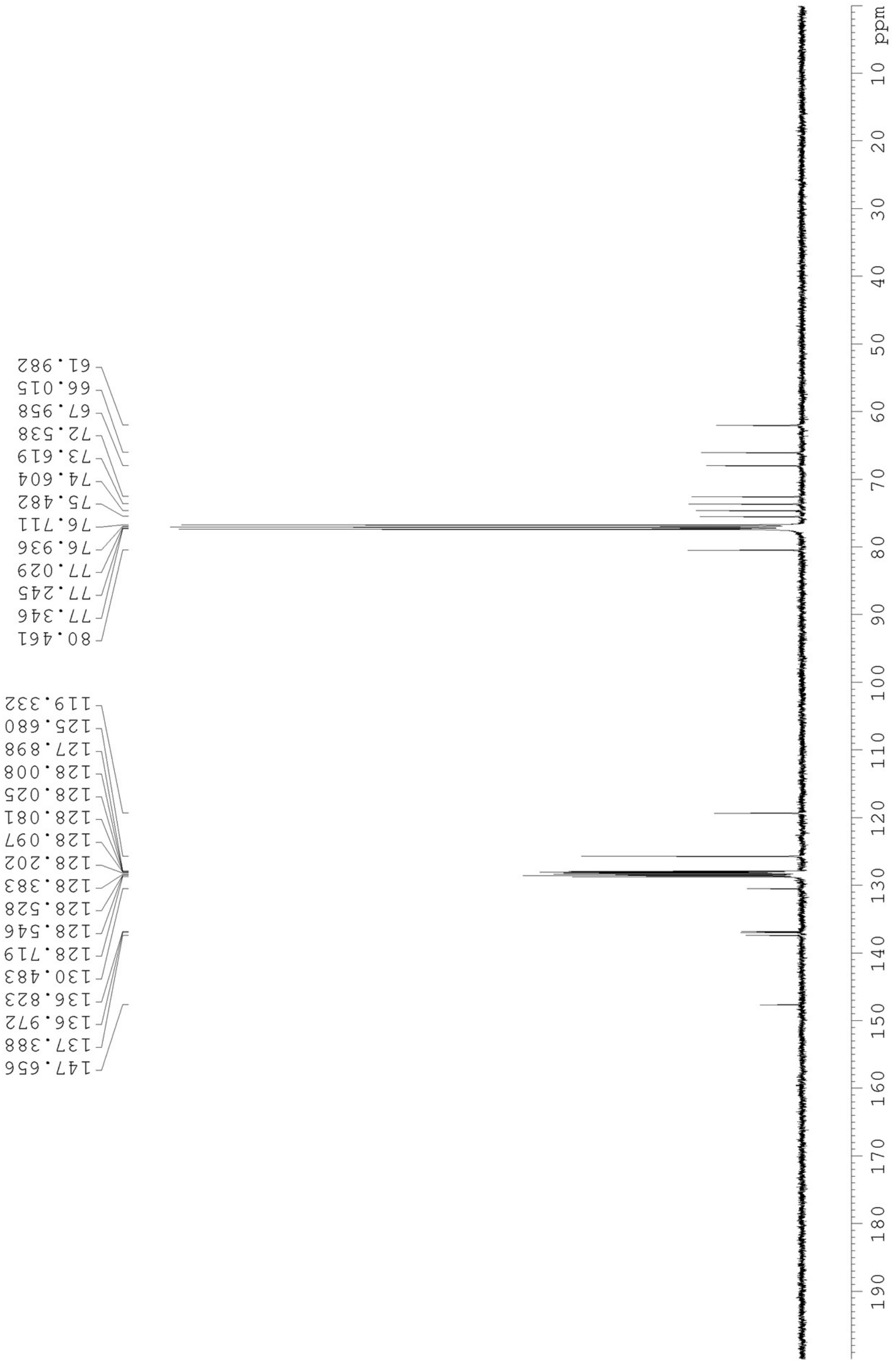

Compound **20** NOESY

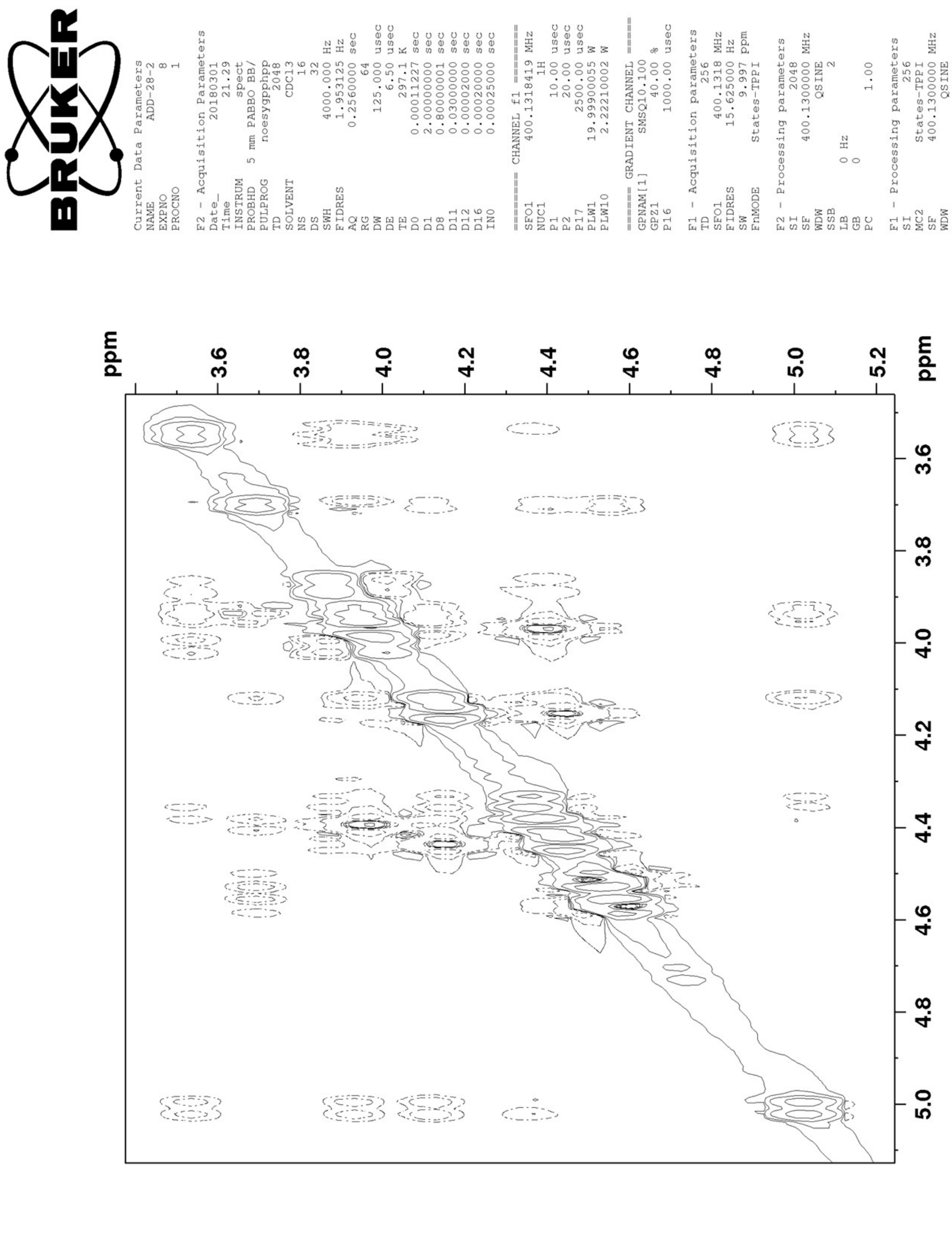

Compound **21** ^{1}H-NMR

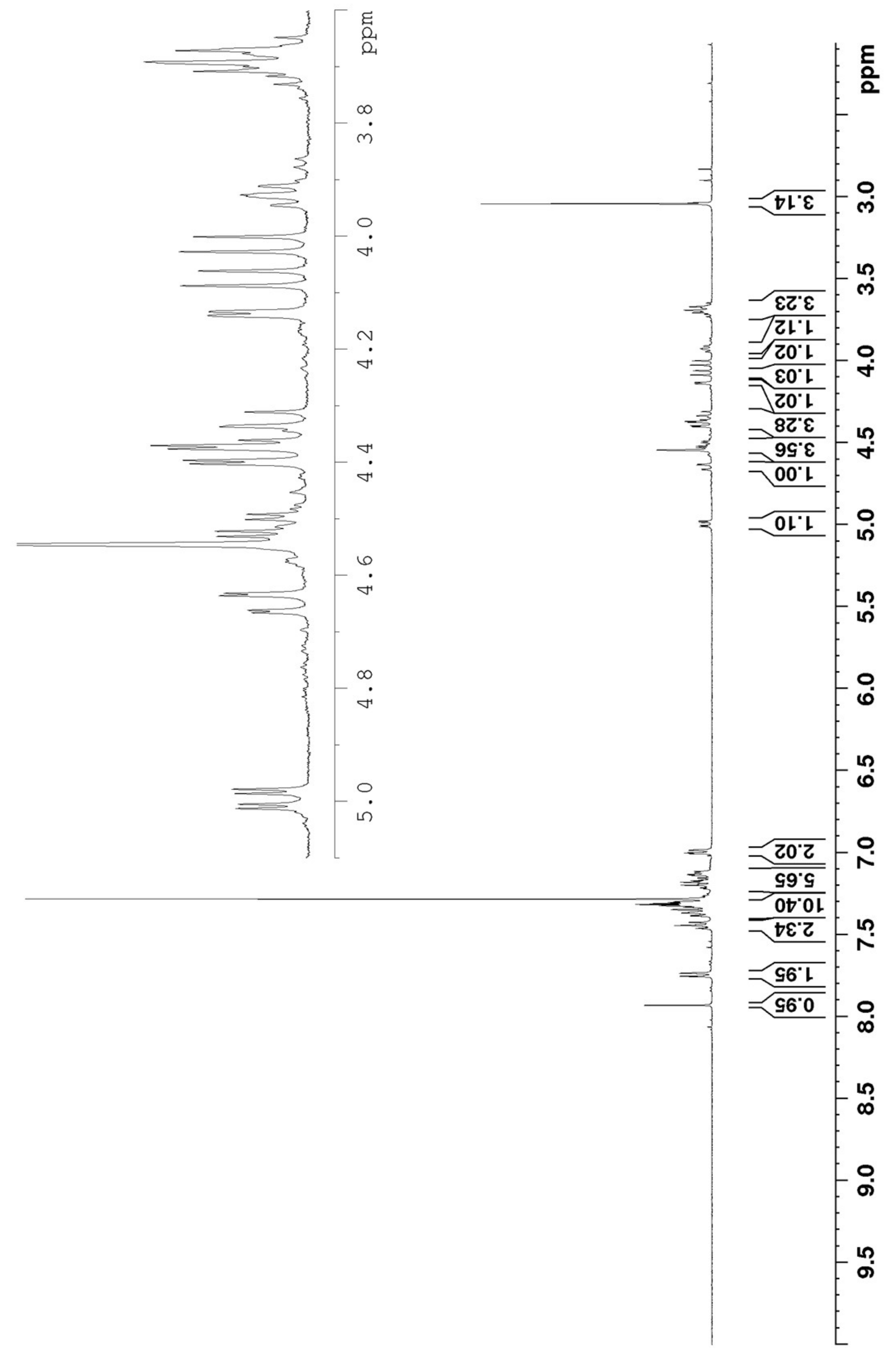

Compound **21** ^{13}C-NMR

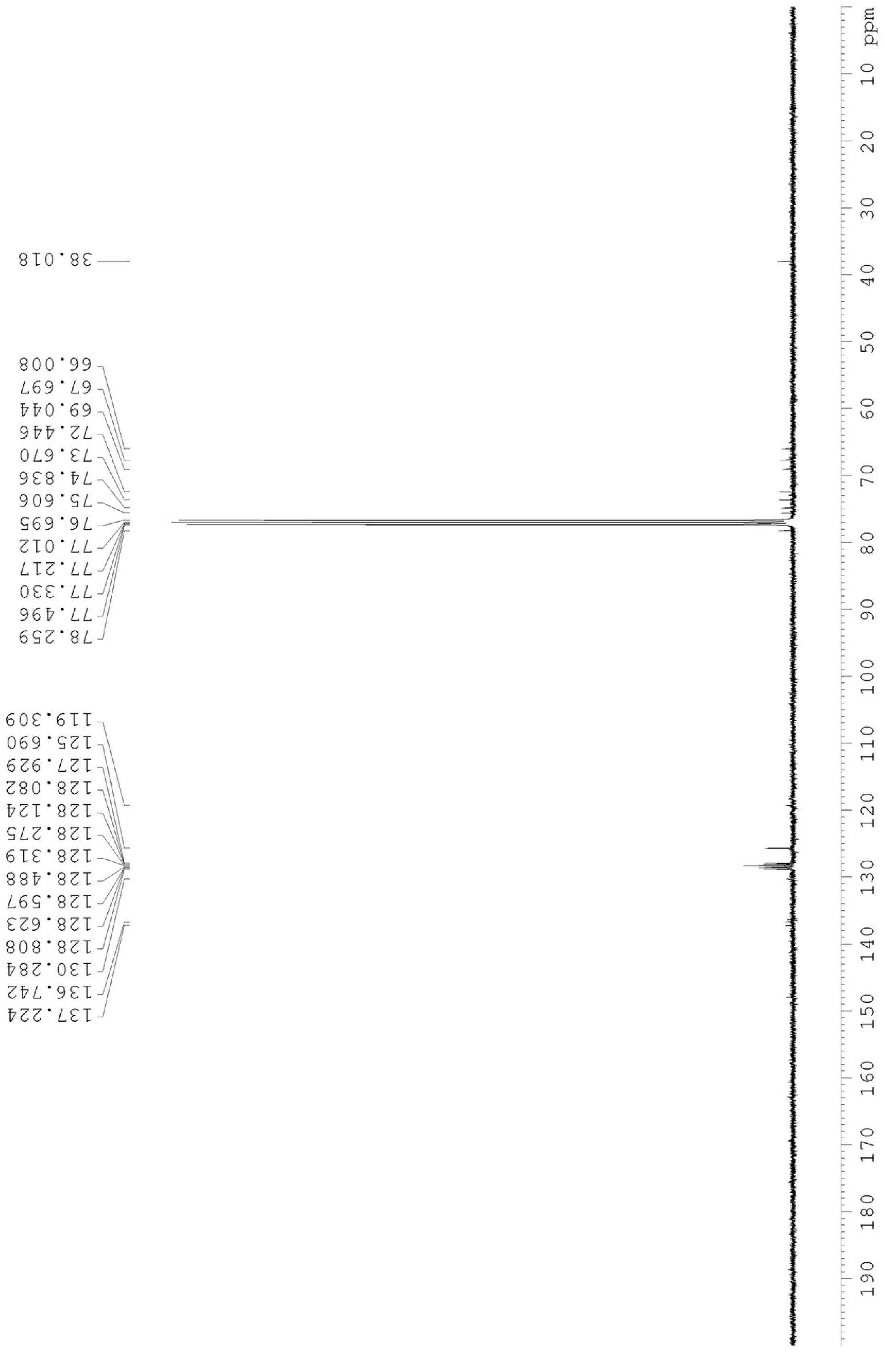

Compound **22** ^{1}H-NMR

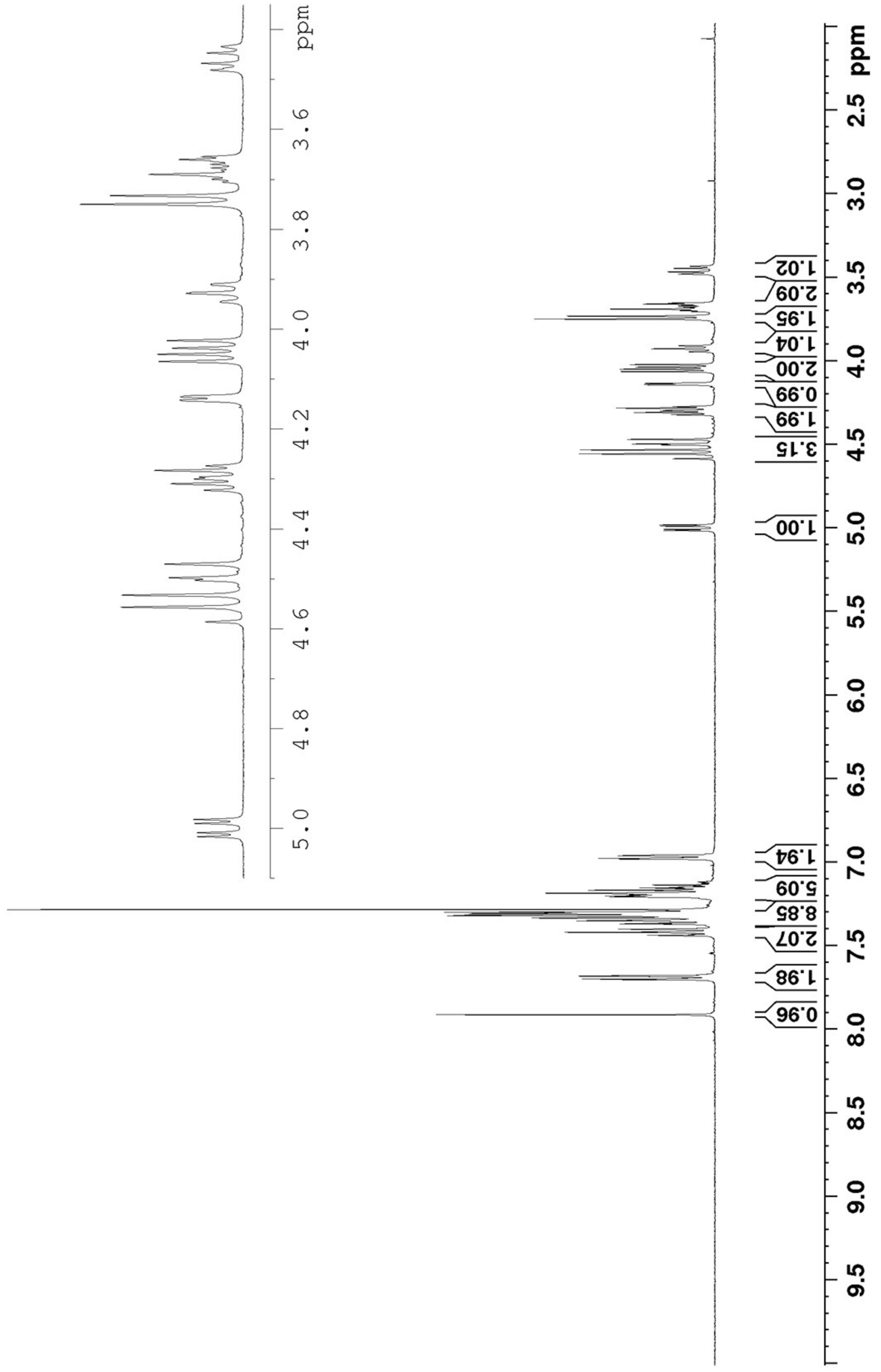

Compound **22** ^{13}C-NMR

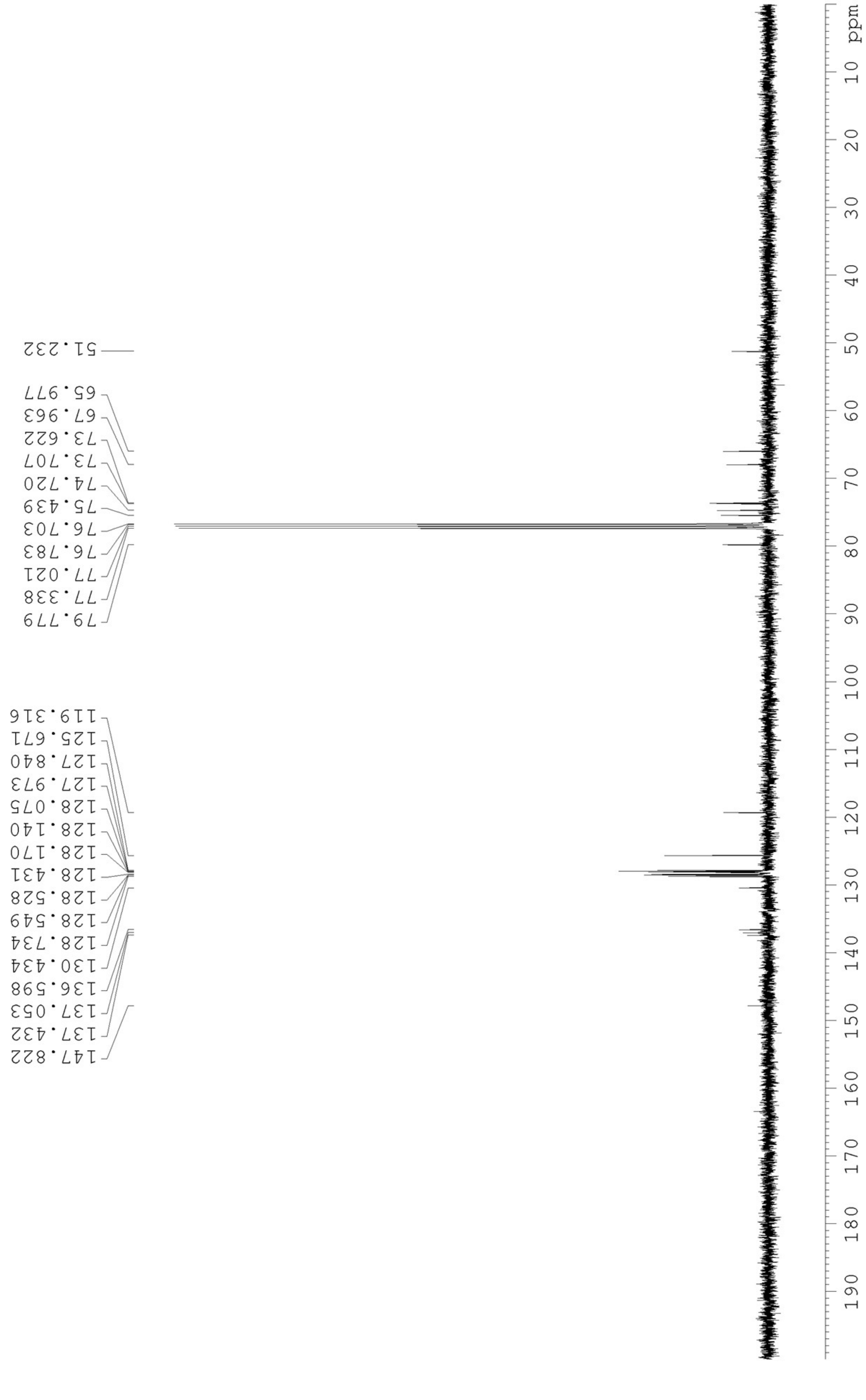

Compound **23a** ^{1}H-NMR

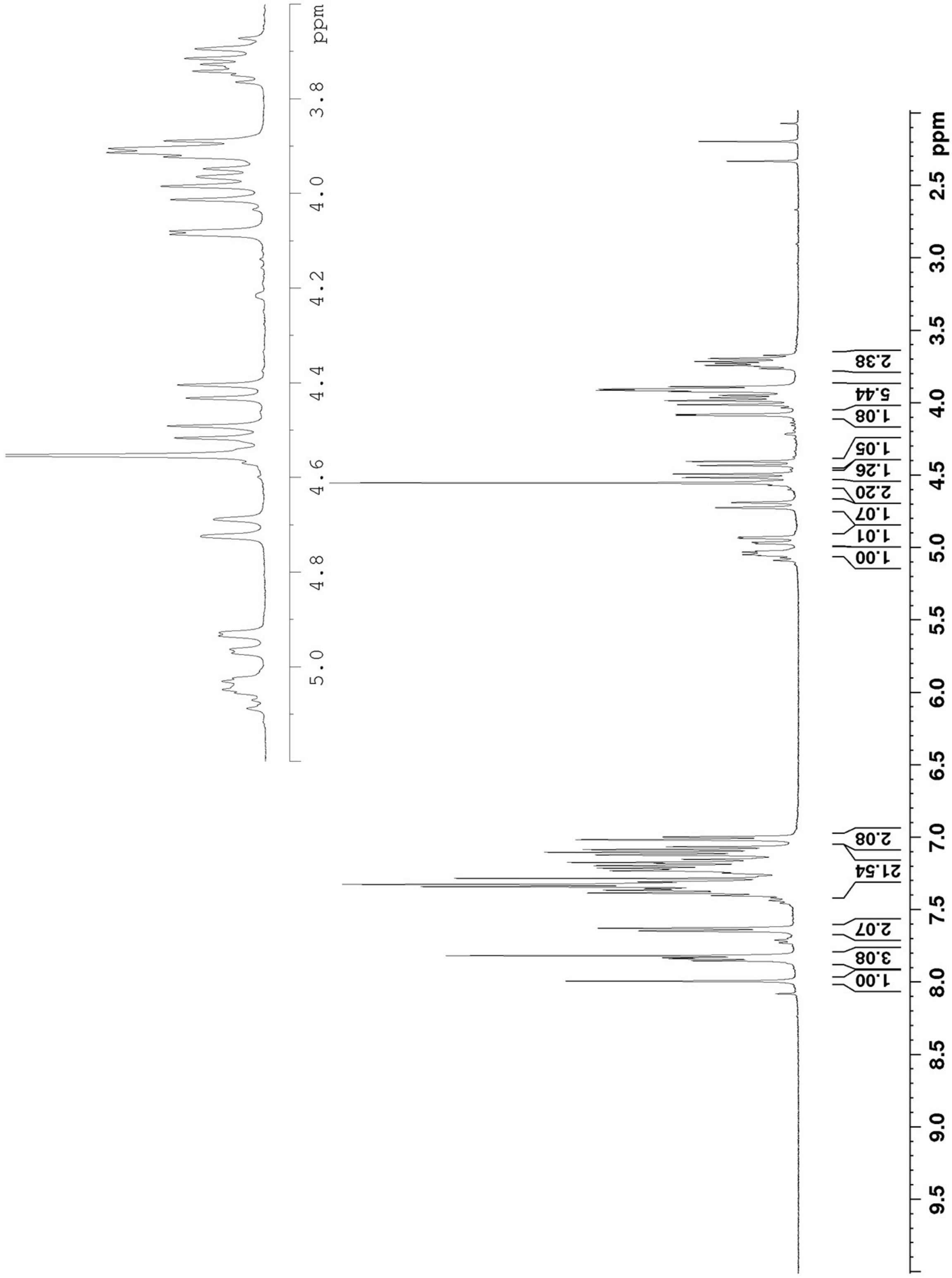

Compound **23a** ^{13}C-NMR

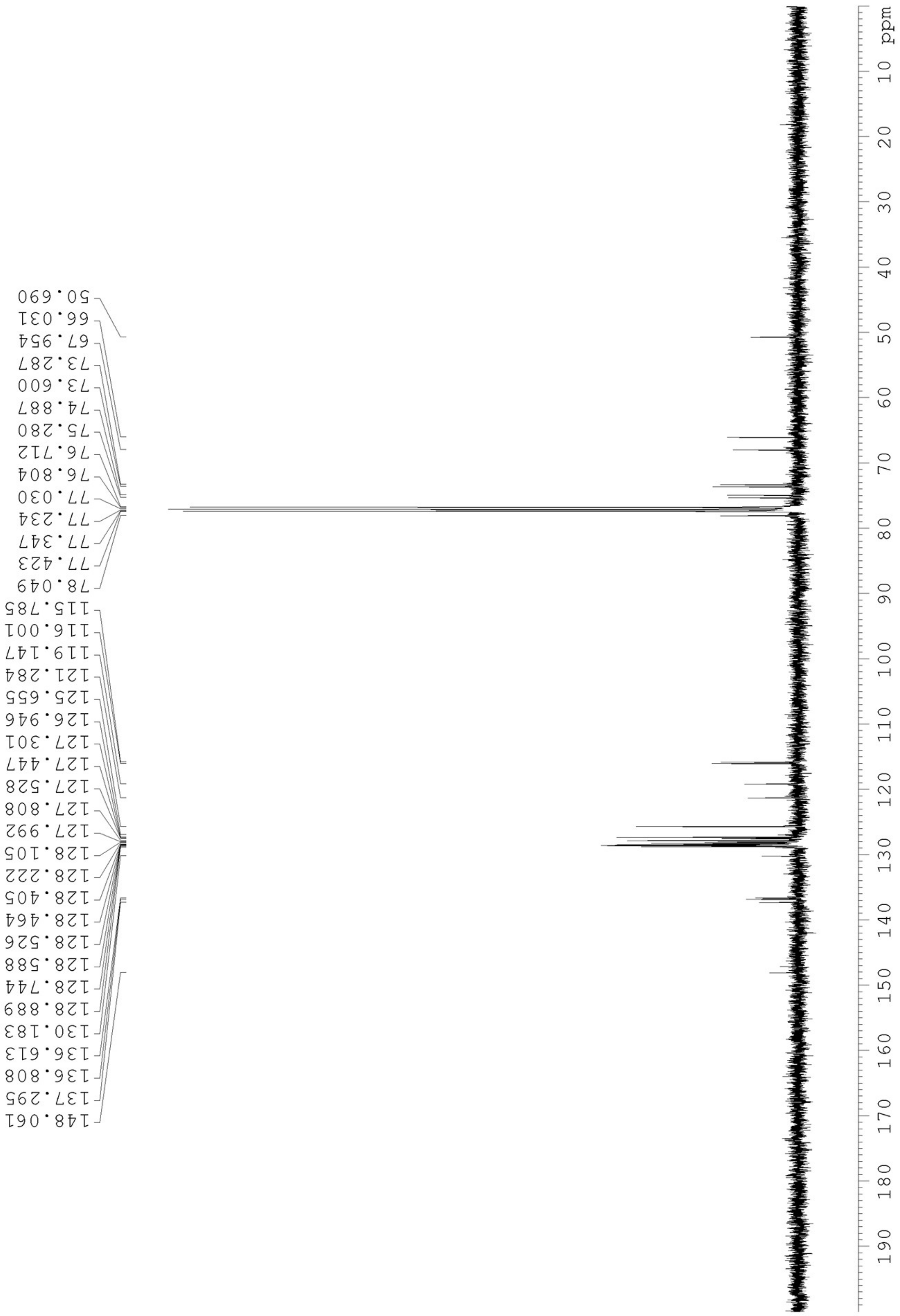

Compound **23b** ^{1}H-NMR

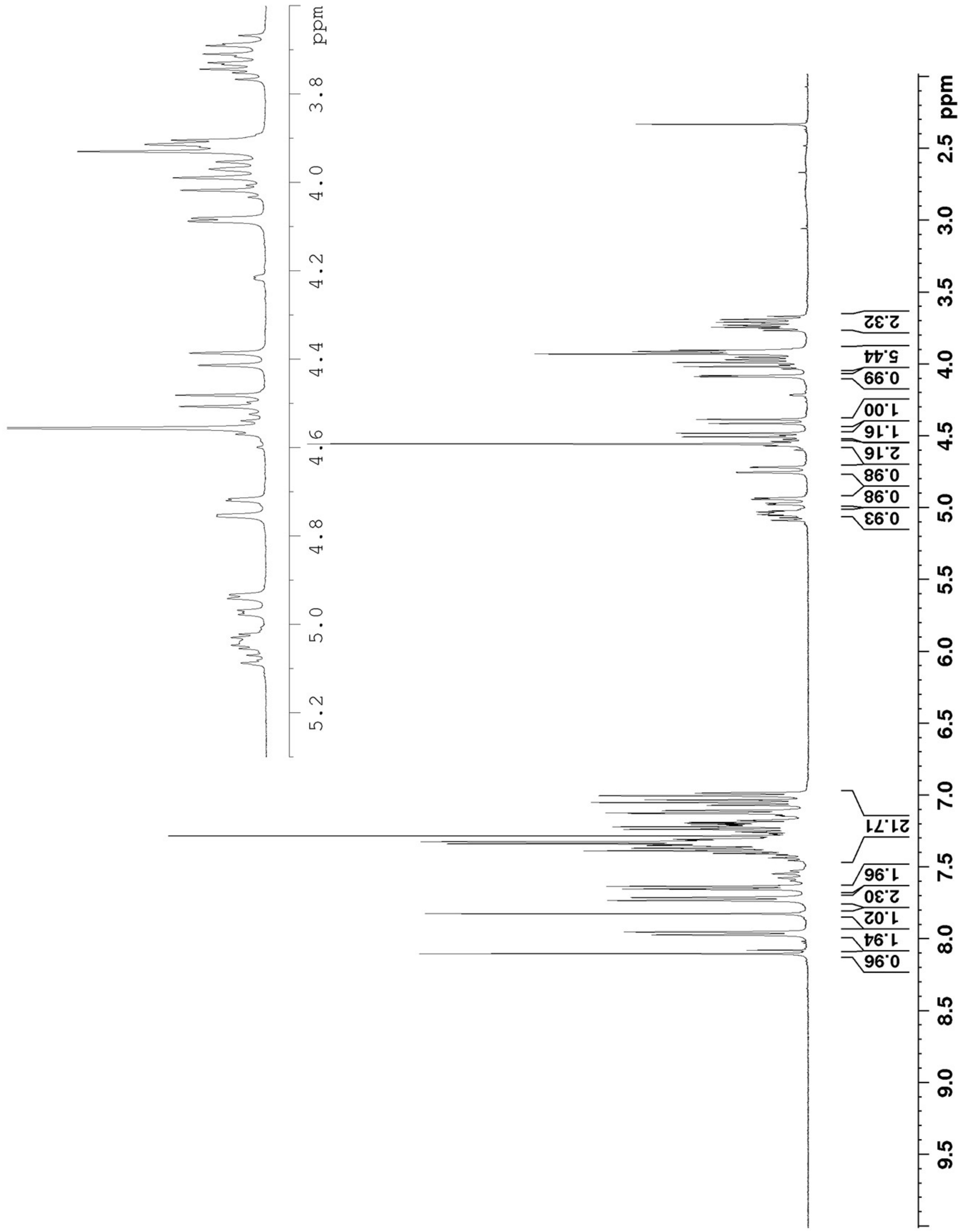

Compound **23b** ^{13}C-NMR

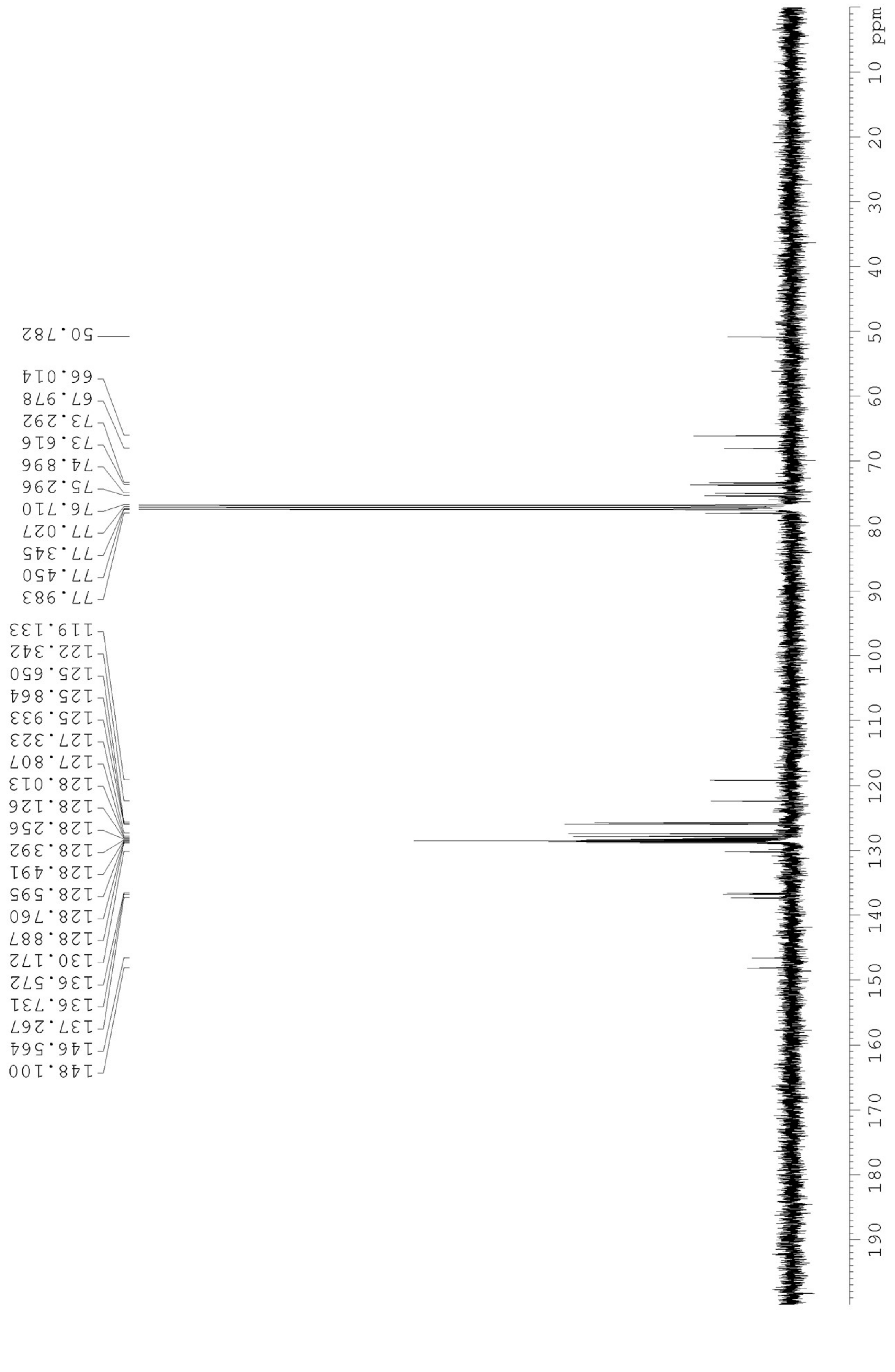

Compound **23c** ^{1}H-NMR

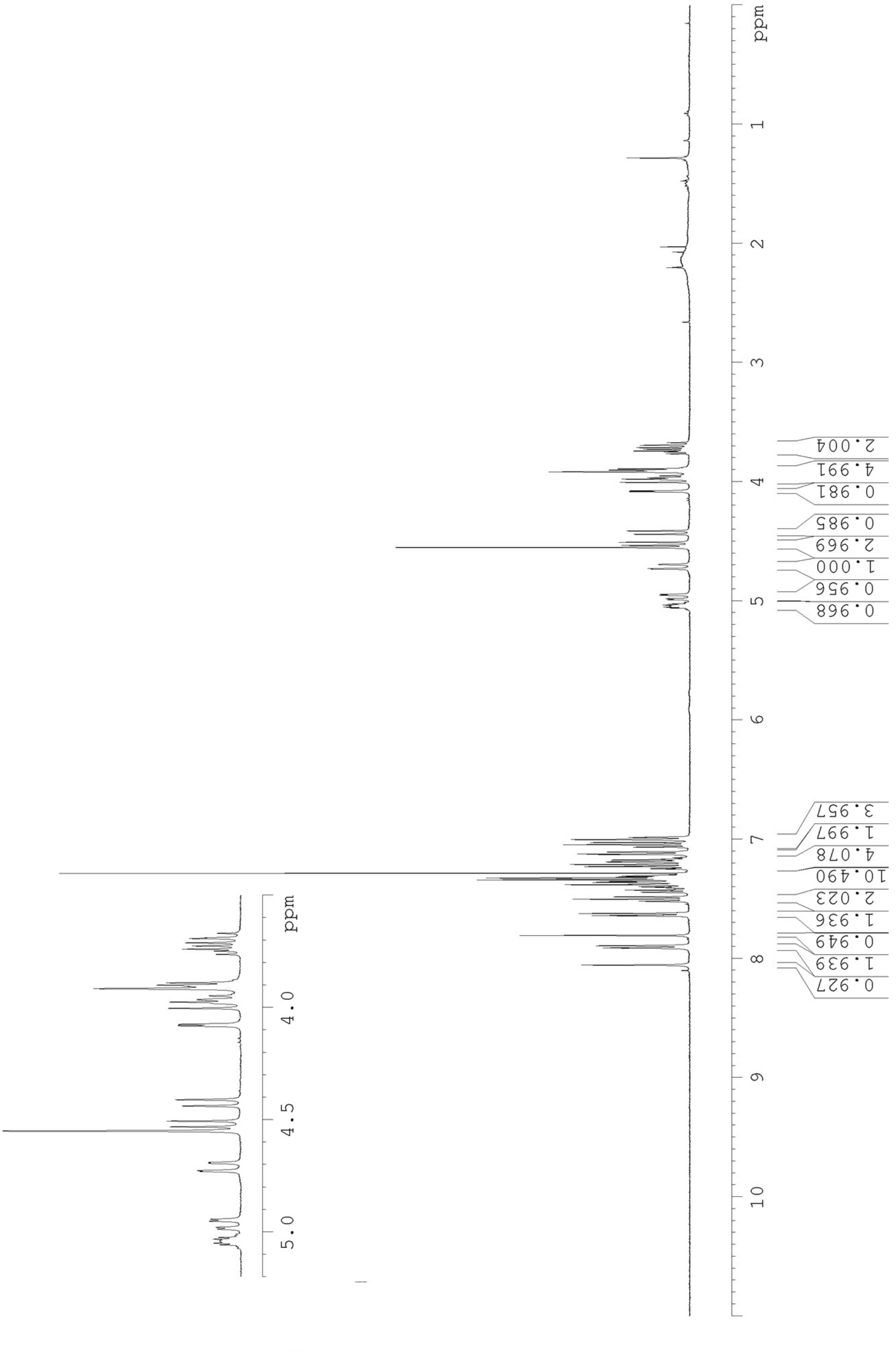

Compound **23c** ^{13}C-NMR

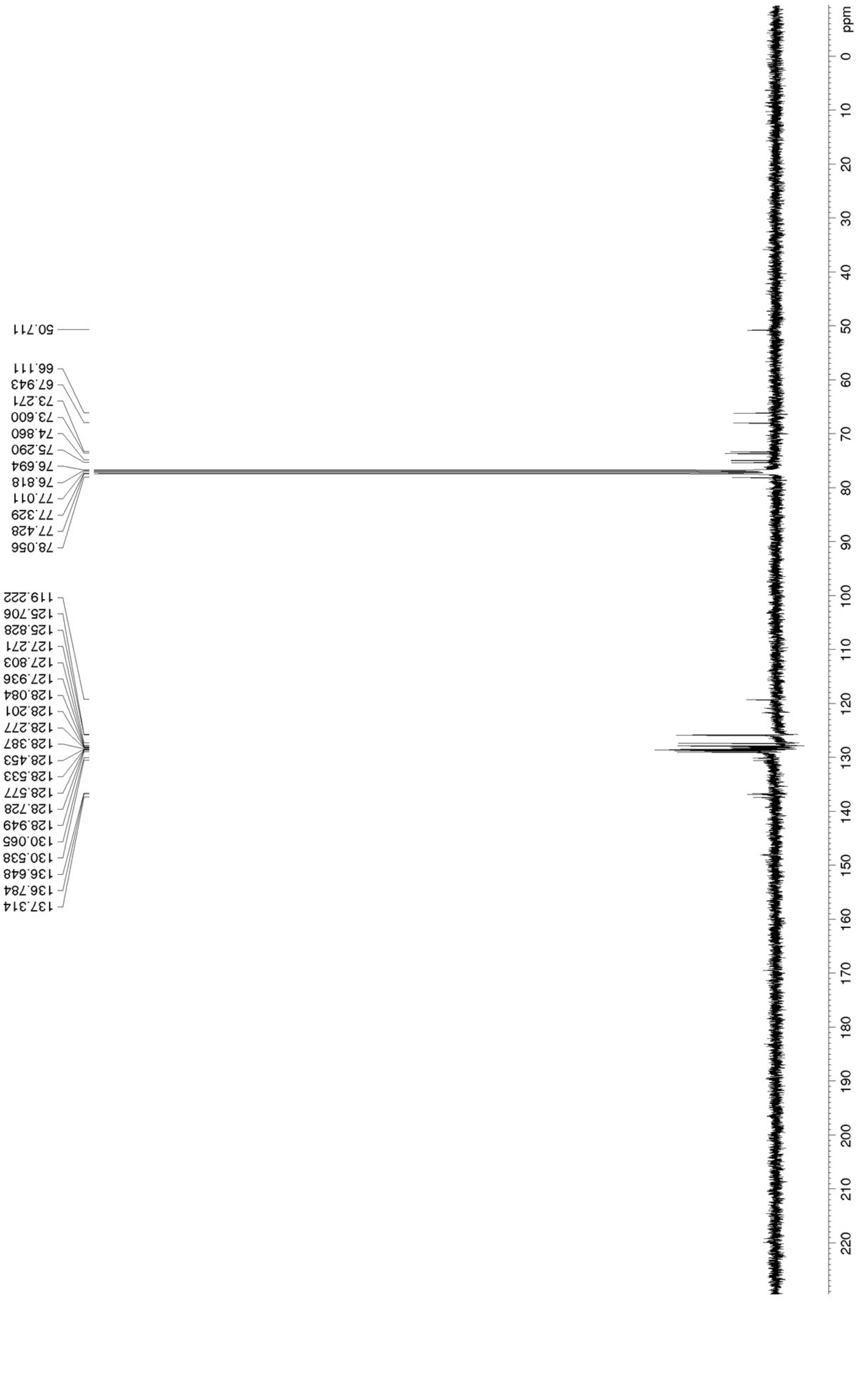

Compound **23d** ^{1}H-NMR

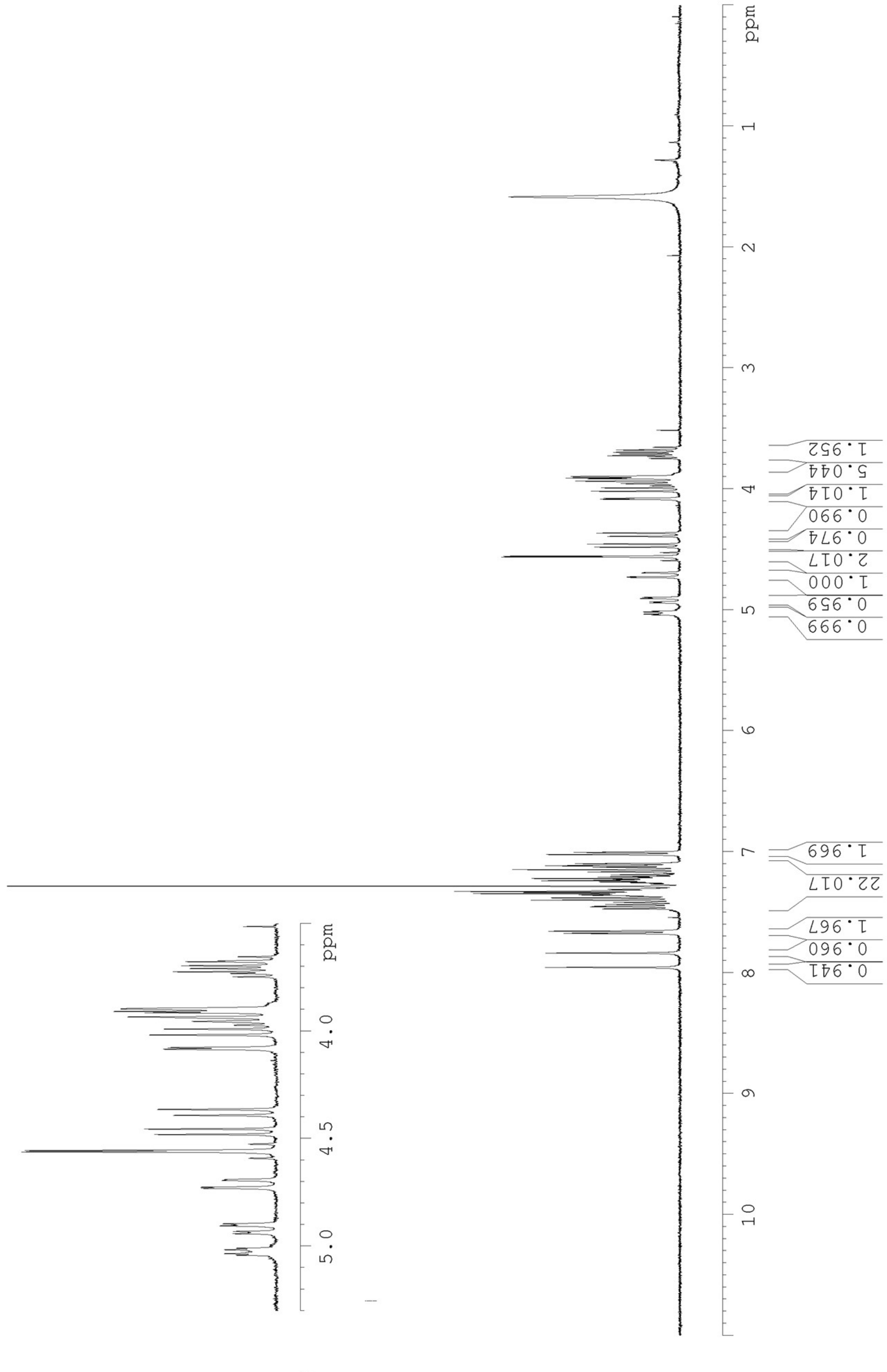

Compound **23d** ^{13}C-NMR

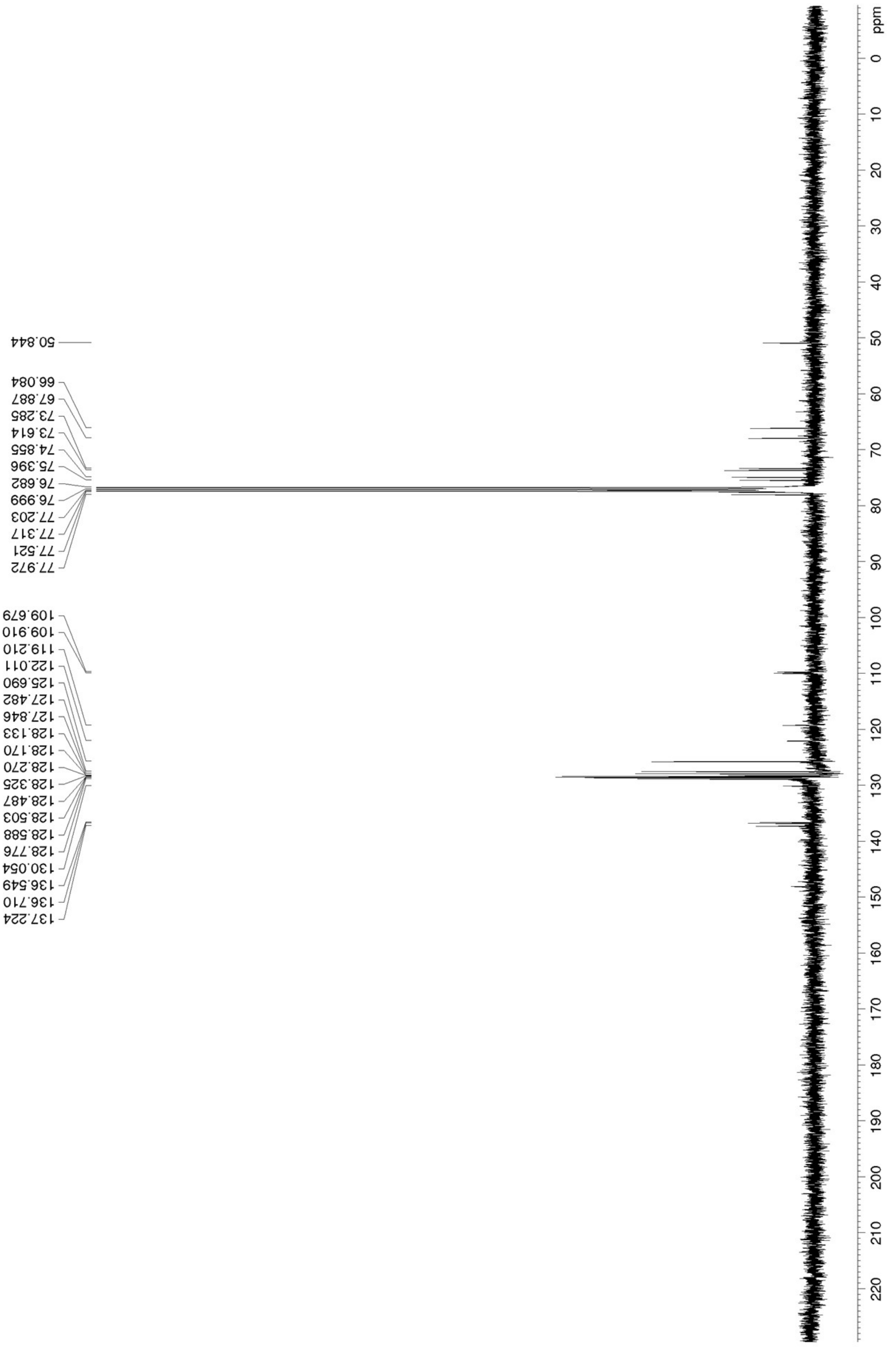

Compound **24a** ^{1}H-NMR

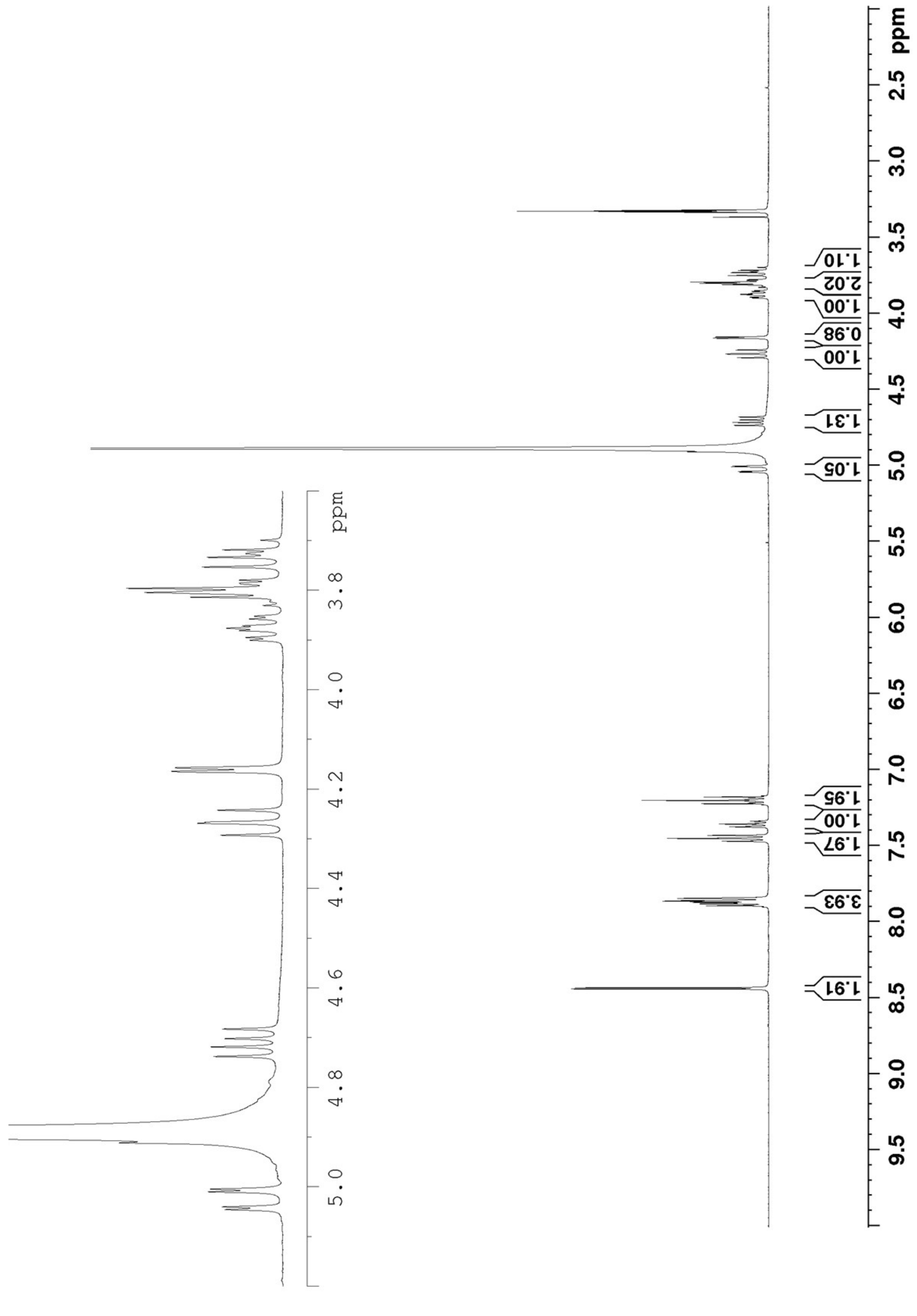

Compound **24a** ^{13}C-NMR

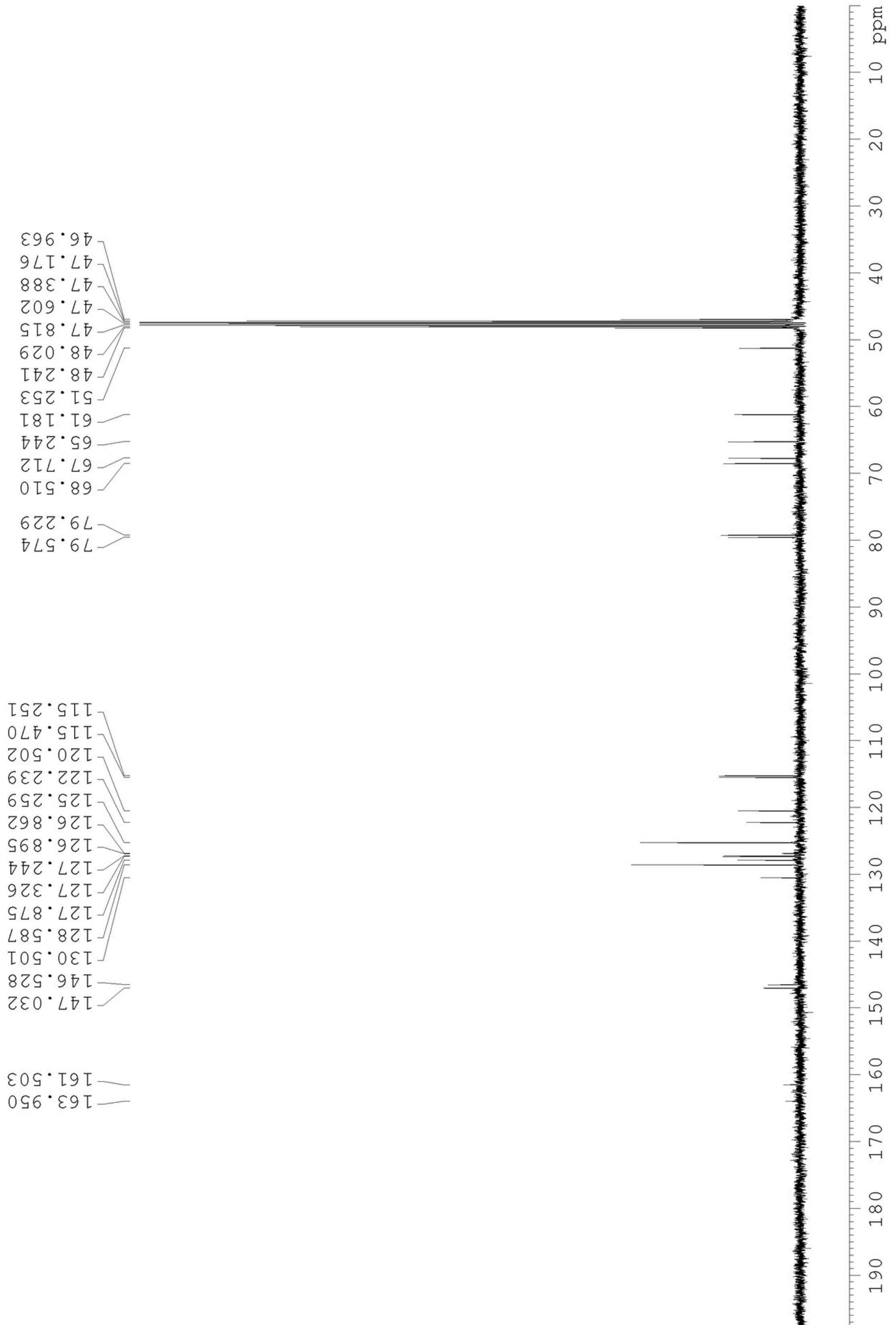

Compound **24b** ^{1}H-NMR

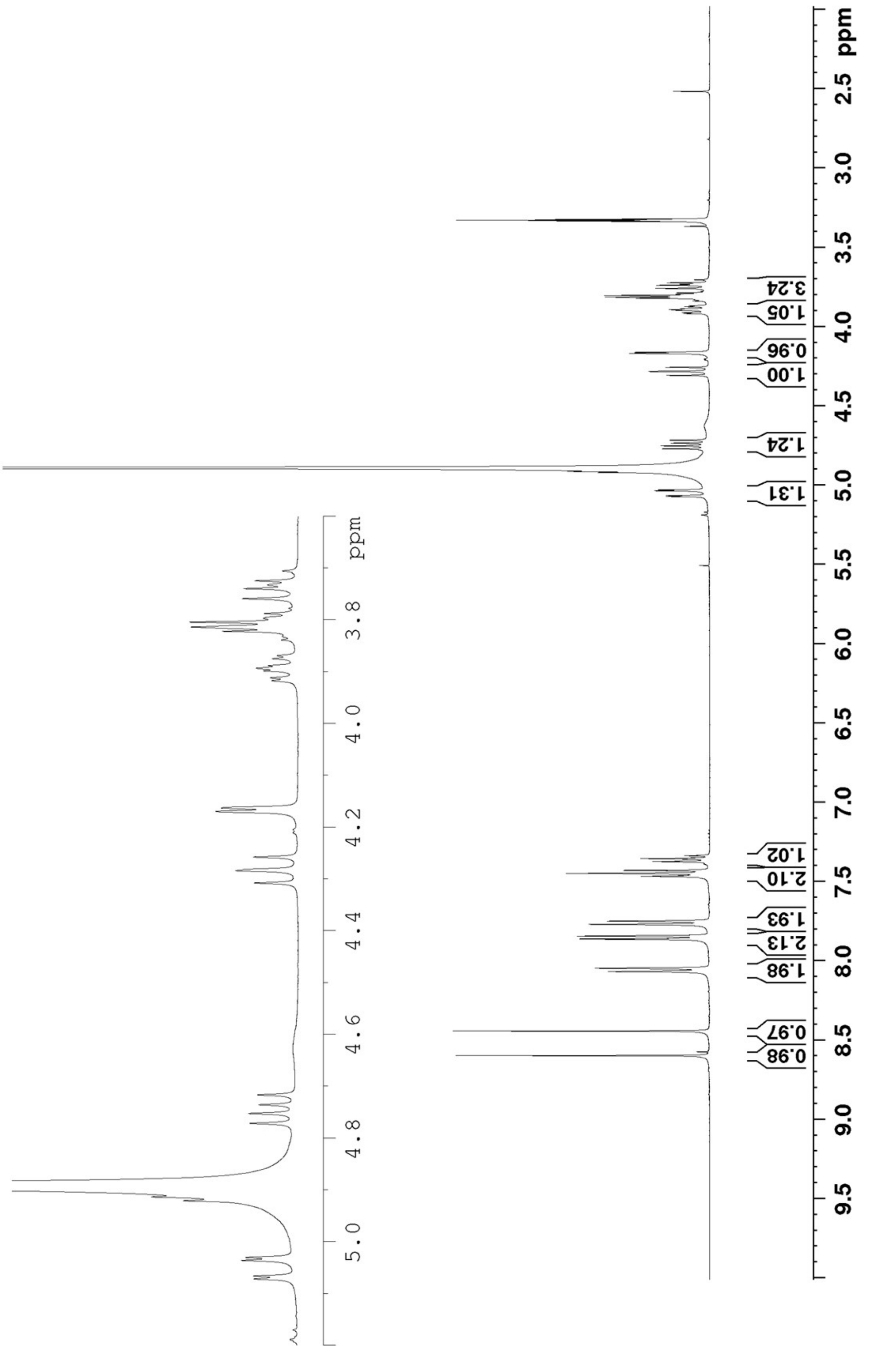

Compound **24b** ^{13}C-NMR

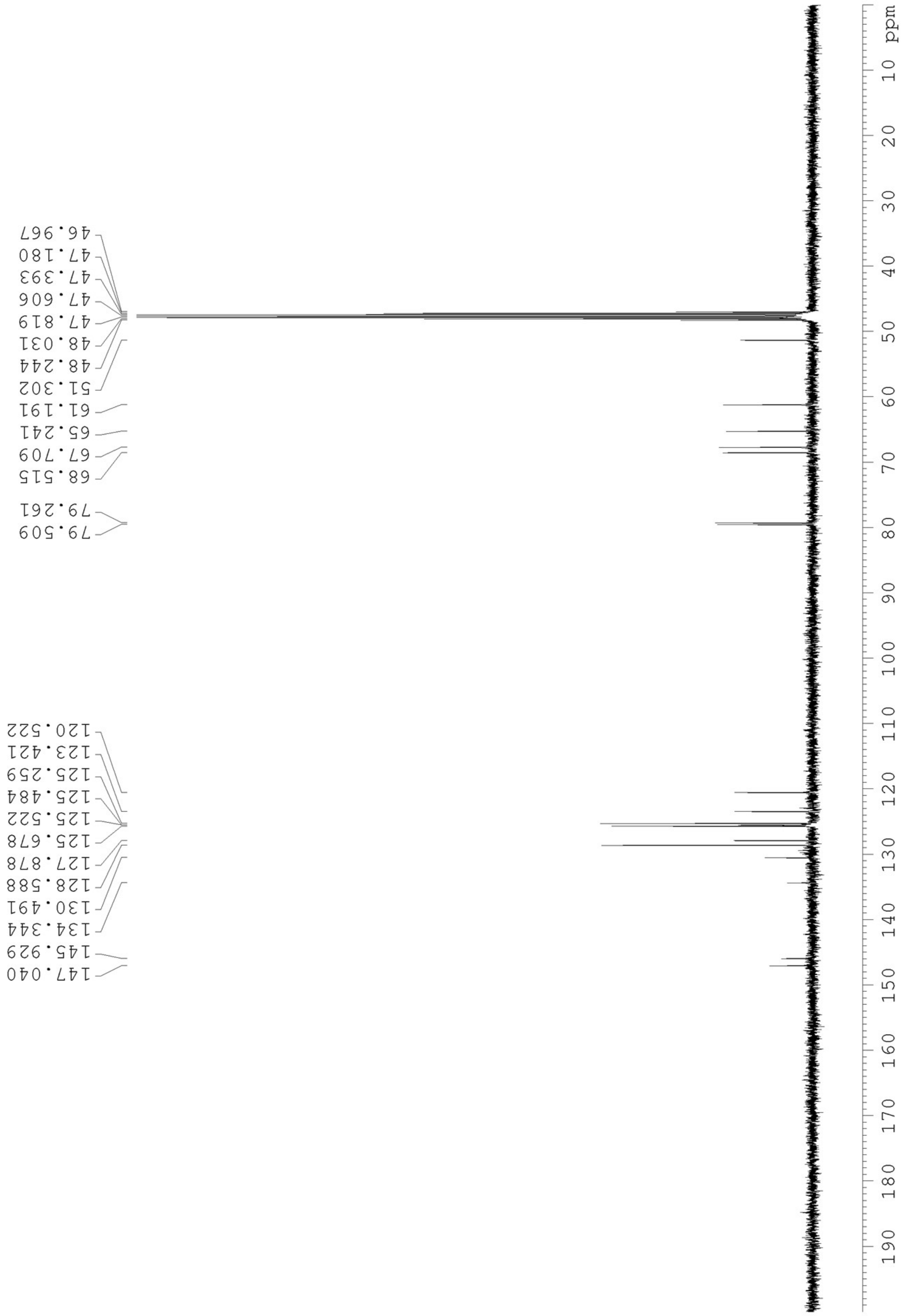

Compound **24c** ^{1}H-NMR

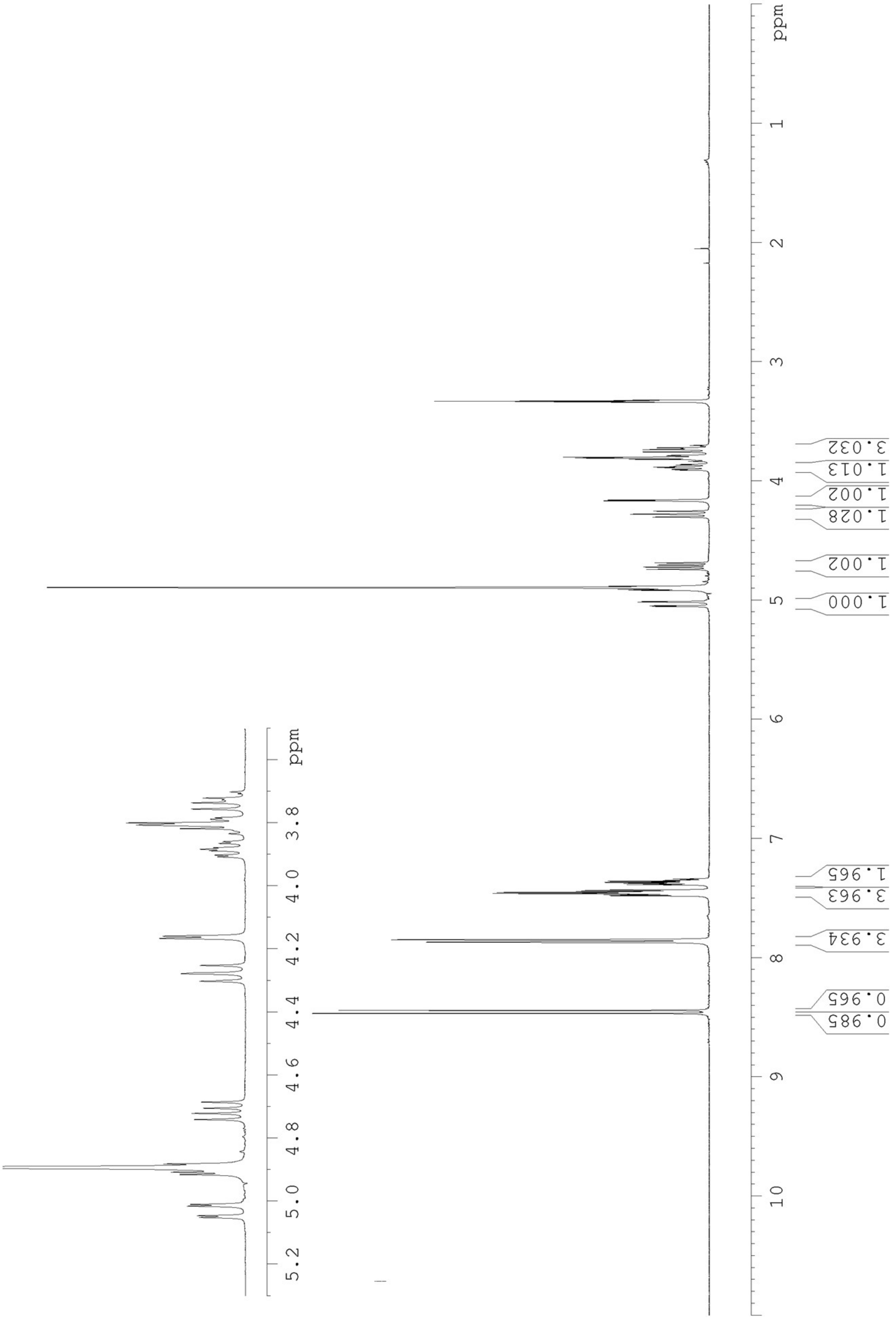

Compound **24c** ^{13}C-NMR

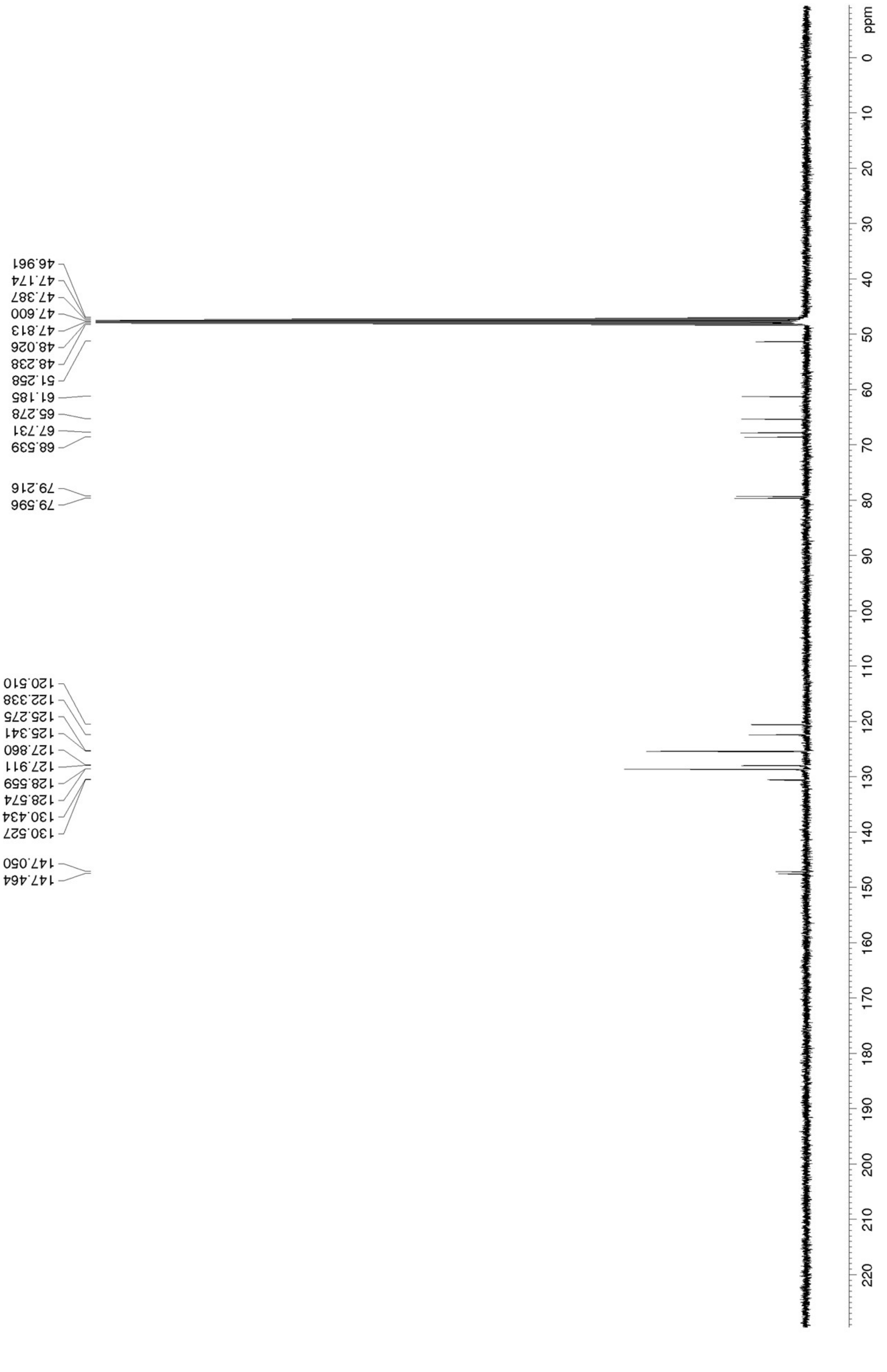

Compound **24d** ^{1}H-NMR

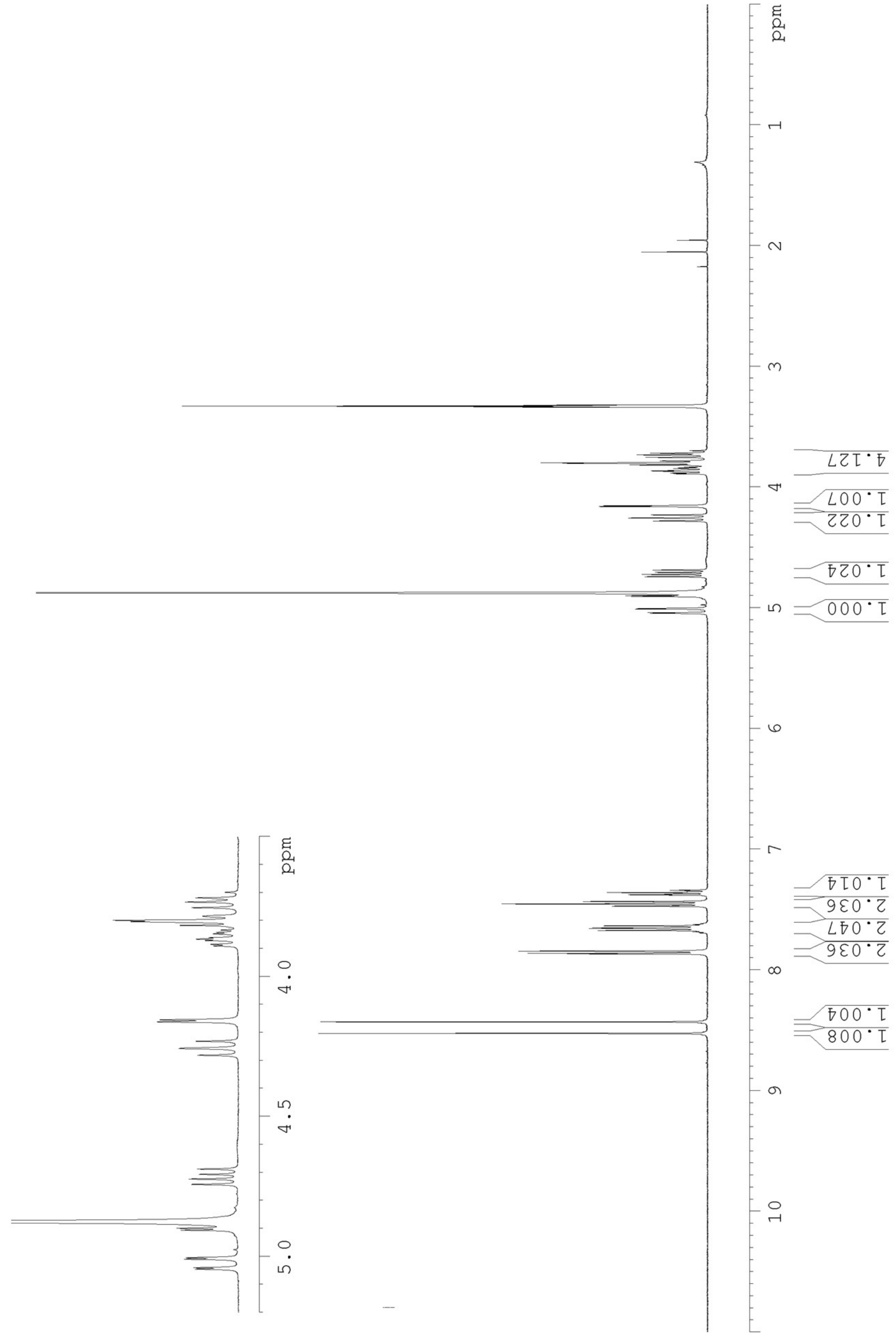

Compound **24c** ^{13}C-NMR

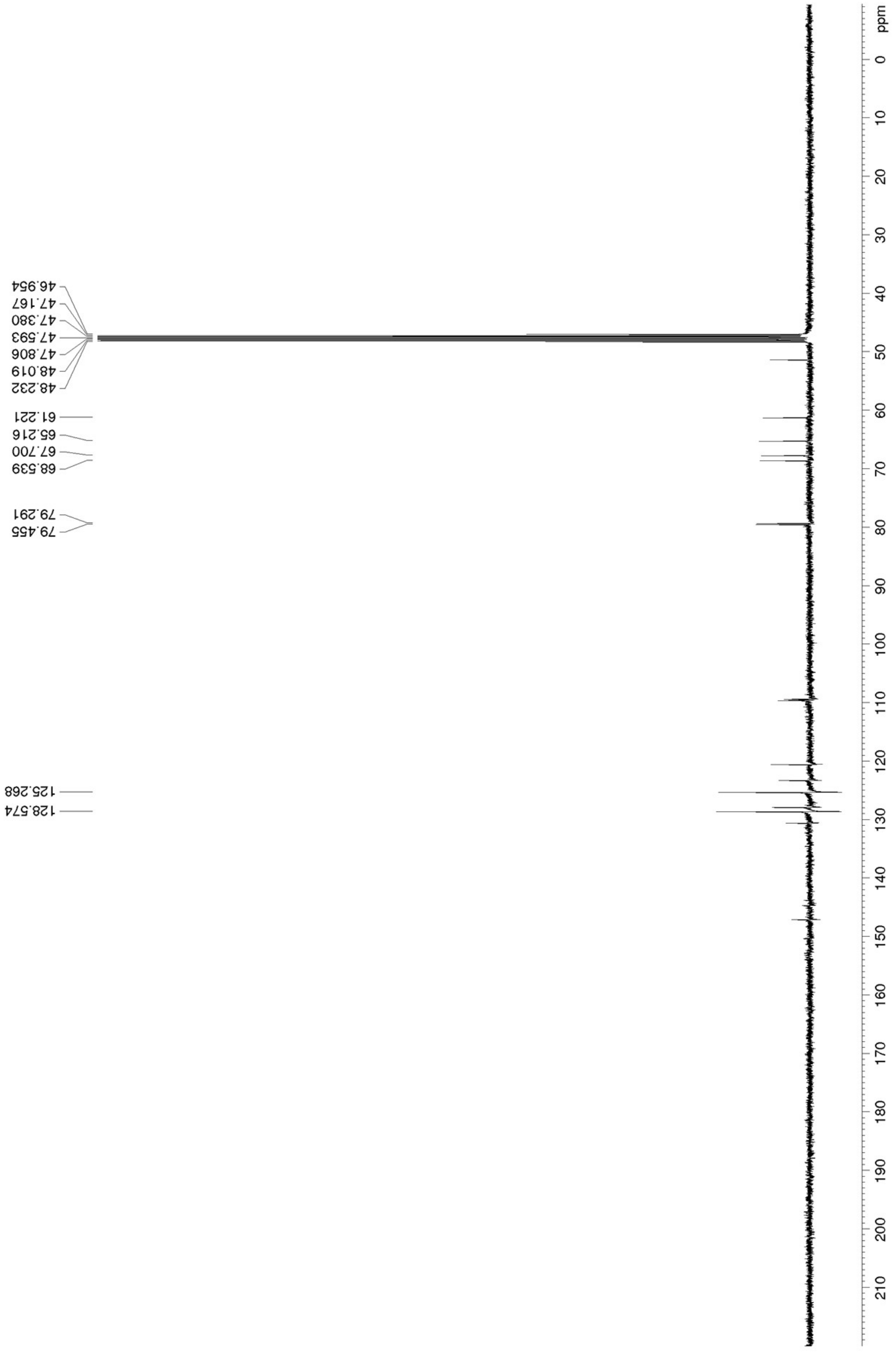

Compound **24a** ^{1}H-NMR in D_2O with 10% DMSO-d6

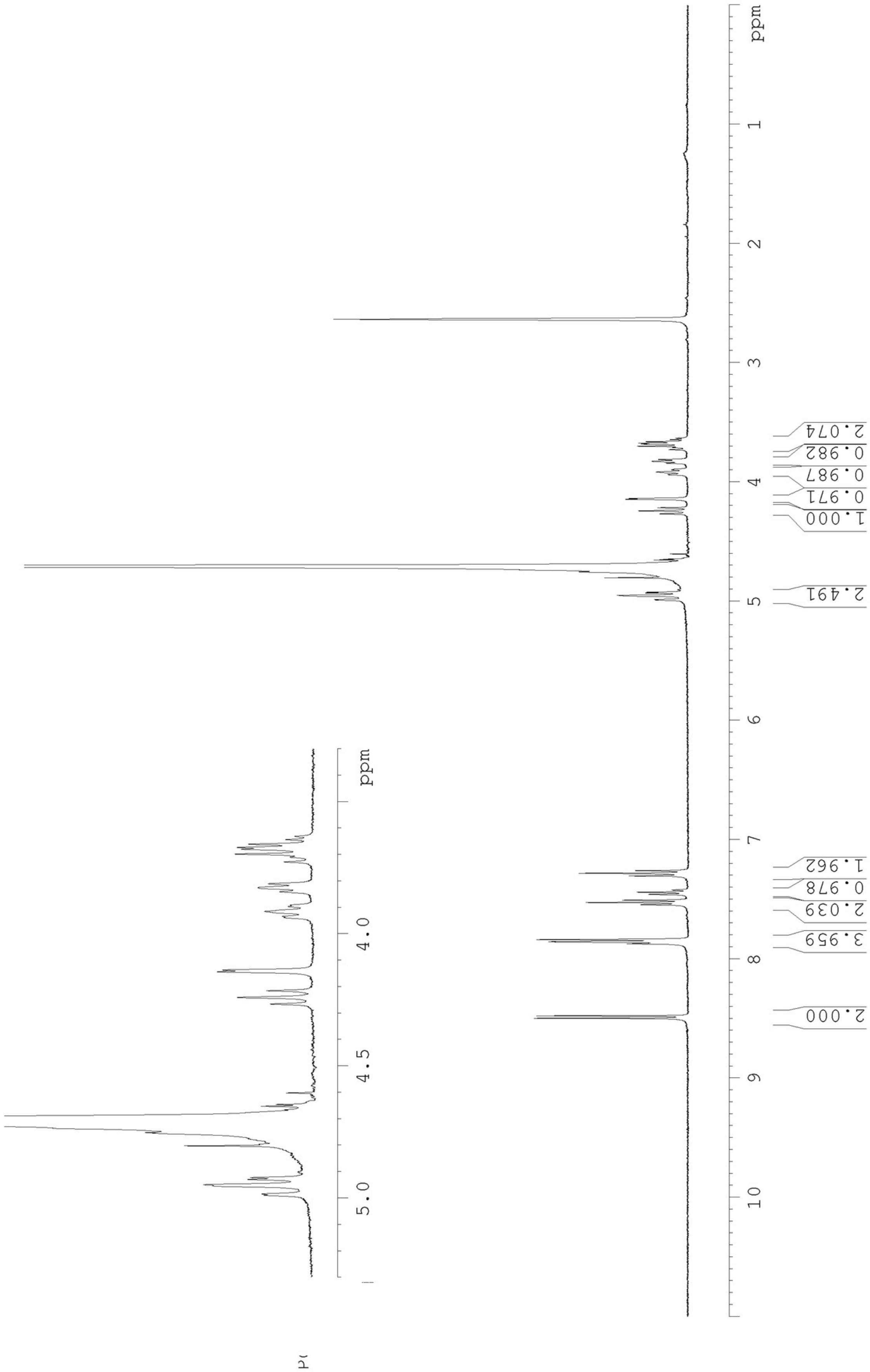

Compound **1c**

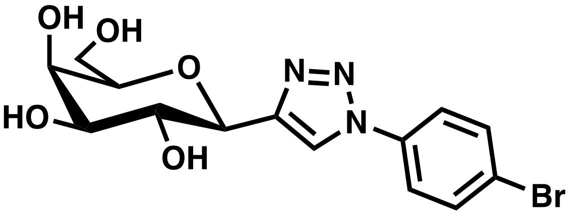
OH
OH
O
N=N
N
HO
OH
Br

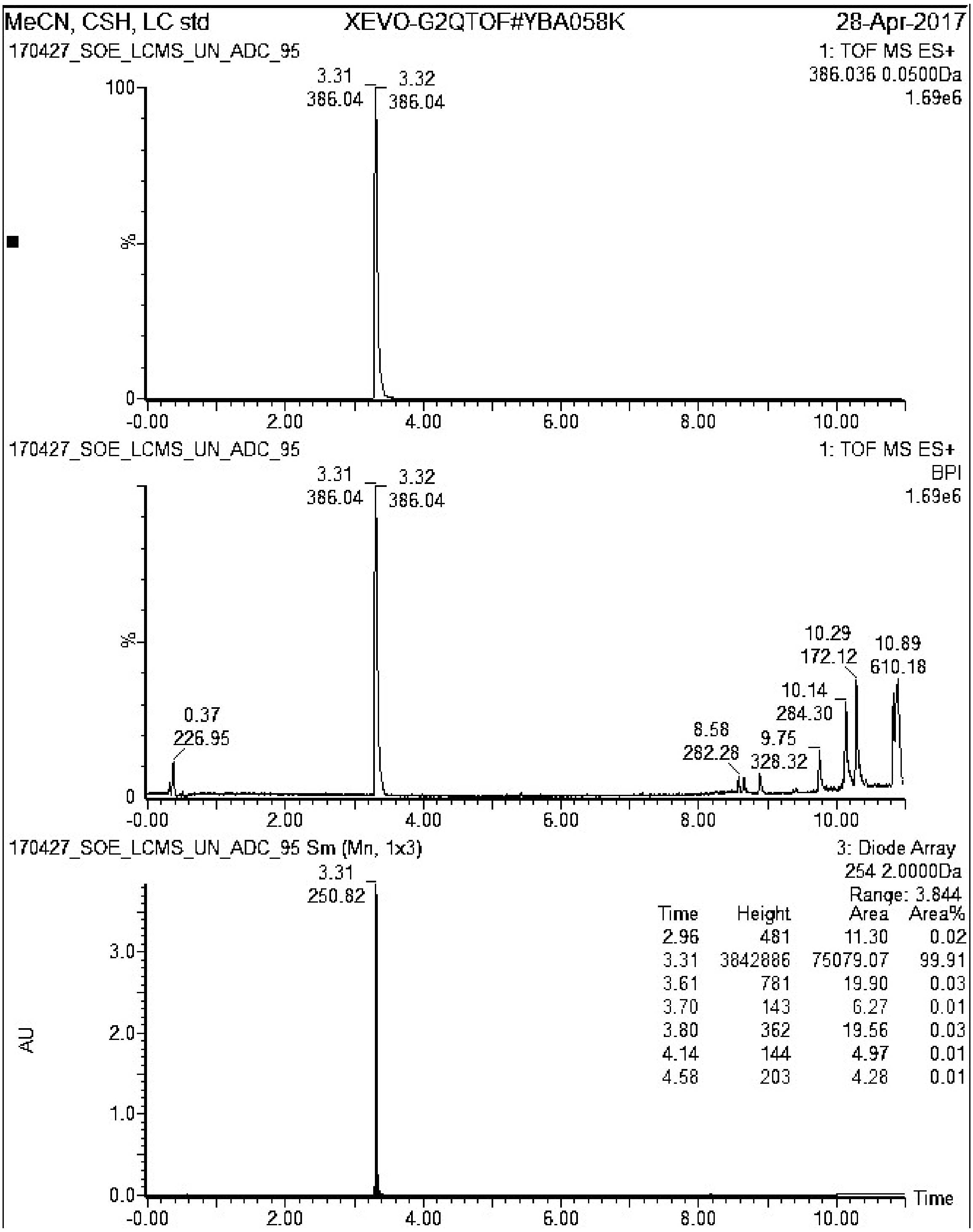
MeCN, CSH, LC std
XEVO-G2QTOF#YBA058K
28-Apr-2017
170427_SOE_LCMS_UN_ADC_95
1: TOF MS ES+
386.036 0.0500Da
1.69e6
3.31
386.04
3.32
386.04
170427_SOE_LCMS_UN_ADC_95
1: TOF MS ES+
BPI
1.69e6
3.31
386.04
3.32
386.04
0.37
226.95
8.58
282.28
9.75
328.32
10.14
284.30
10.29
172.12
10.89
610.18
170427_SOE_LCMS_UN_ADC_95 Sm (Mn, 1x3)
3: Diode Array
254 2.0000Da
Range: 3.844
3.31
250.82
Time Height Area Area%
2.96 481 11.30 0.02
3.31 3842886 75079.07 99.91
3.61 781 19.90 0.03
3.70 143 6.27 0.01
3.80 362 19.56 0.03
4.14 144 4.97 0.01
4.58 203 4.28 0.01
AU
Time

Compound **1d**

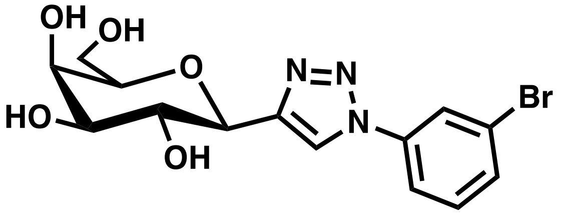
OH
OH
O
N=N
N
Br
HO
OH

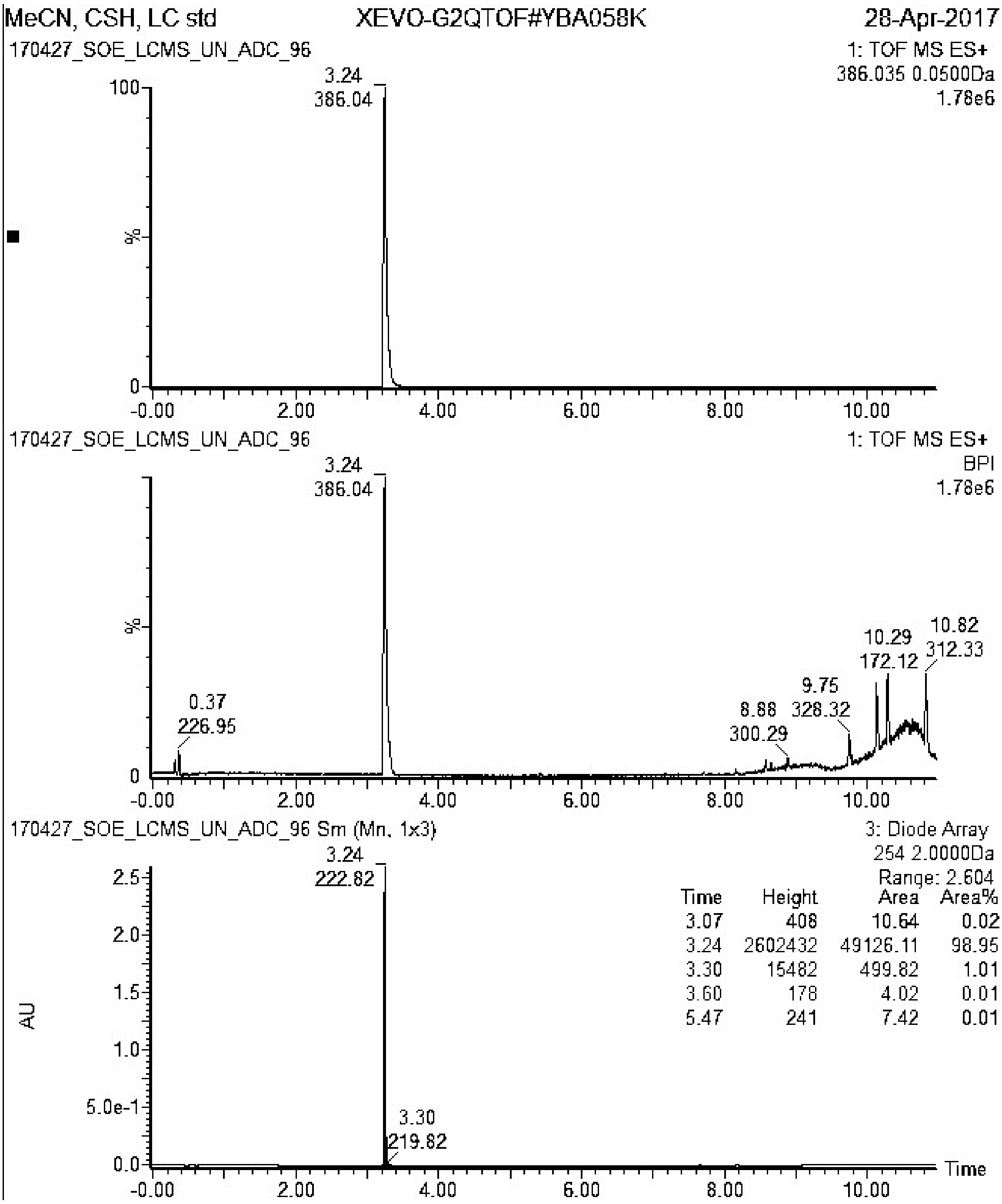
MeCN, CSH, LC std
XEVO-G2QTOF#YBA058K
28-Apr-2017
170427_SOE_LCMS_UN_ADC_96
1: TOF MS ES+
386.035 0.0500Da
1.78e6
3.24
386.04
100
%
0
-0.00
2.00
4.00
6.00
8.00
10.00
170427_SOE_LCMS_UN_ADC_96
1: TOF MS ES+
BPI
1.78e6
3.24
386.04
0.37
226.95
8.88
300.29
9.75
328.32
10.29
172.12
10.82
312.33
170427_SOE_LCMS_UN_ADC_96 Sm (Mn, 1x3)
3: Diode Array
254 2.0000Da
Range: 2.604
3.24
222.82
3.30
219.82
AU
2.5
2.0
1.5
1.0
5.0e-1
0.0
Time
Time Height Area Area%
3.07 408 10.64 0.02
3.24 2602432 49126.11 98.95
3.30 15482 499.82 1.01
3.60 178 4.02 0.01
5.47 241 7.42 0.01

Compound **1e**

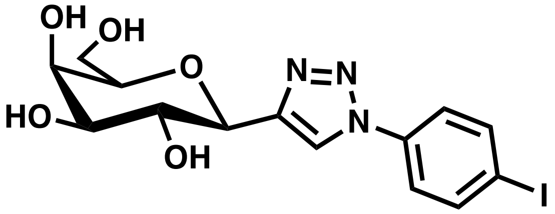

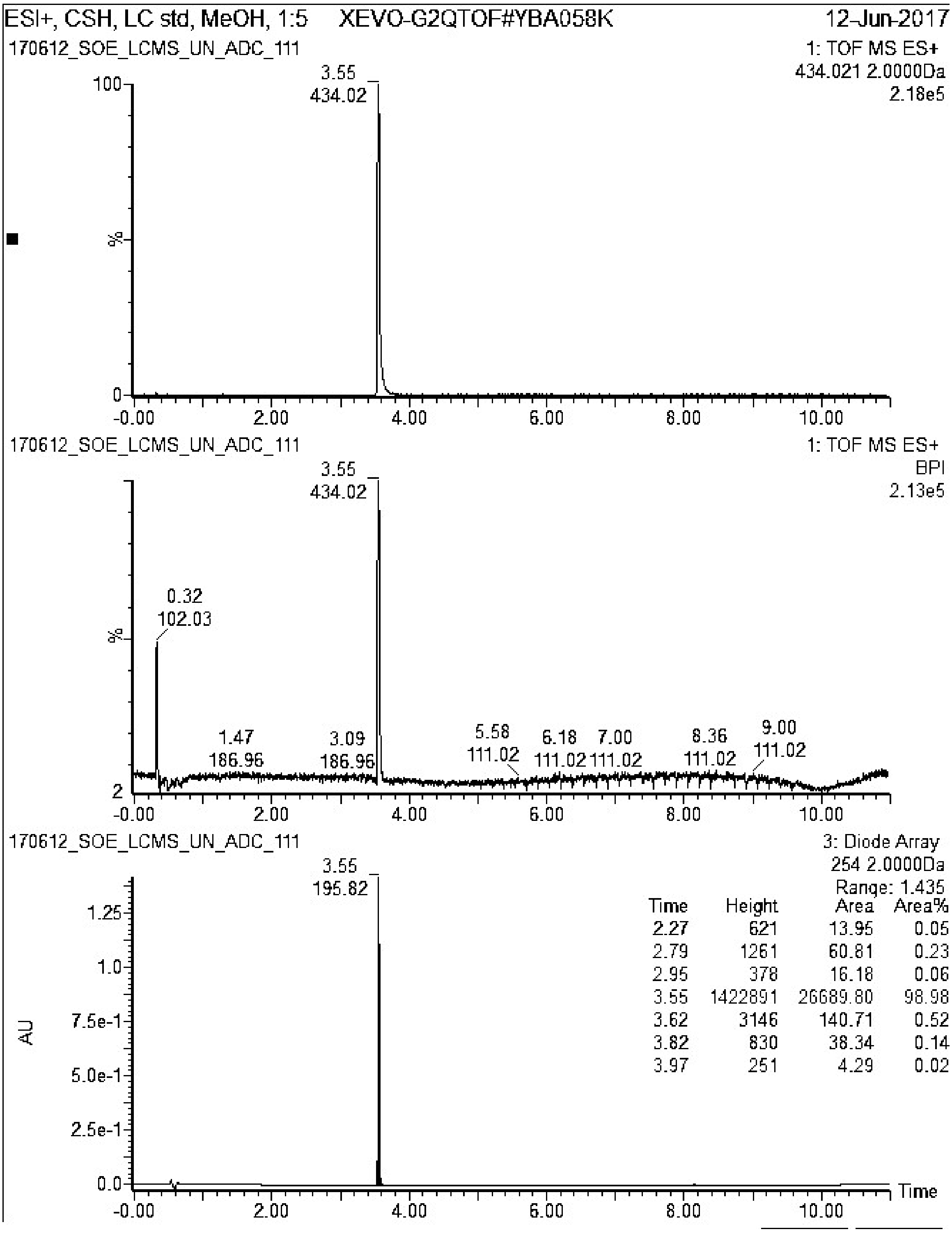

Time	Height	Area	Area%
2.27	621	13.95	0.05
2.79	1261	60.81	0.23
2.95	378	16.18	0.06
3.55	1422891	26689.80	98.98
3.62	3146	140.71	0.52
3.82	830	38.34	0.14
3.97	251	4.29	0.02

Compound **1f**

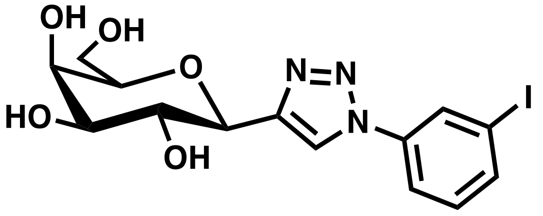

OH
OH
O
HO
OH
N=N
N
I

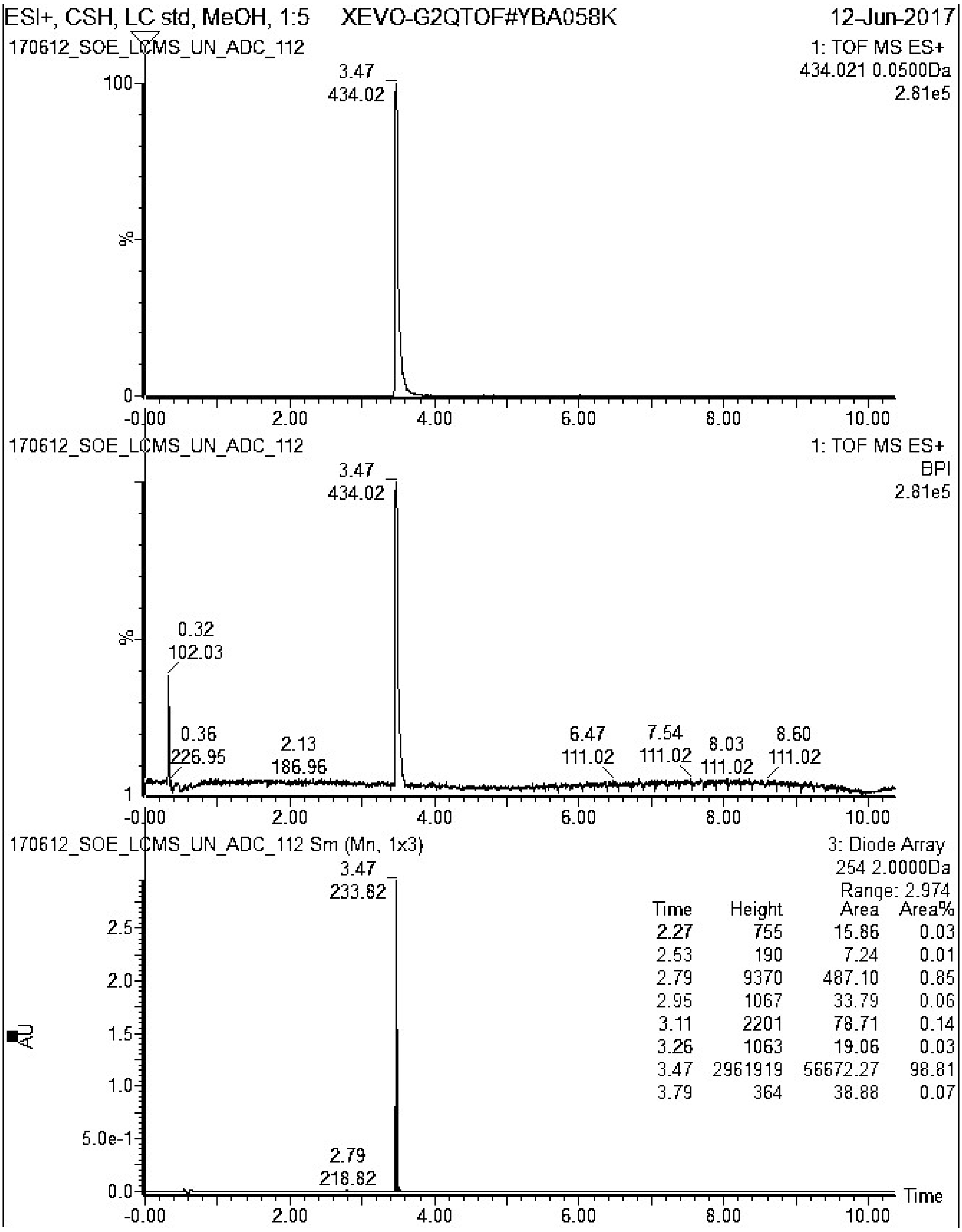

ESI+, CSH, LC std, MeOH, 1:5
XEVO-G2QTOF#YBA058K
12-Jun-2017
170612_SOE_LCMS_UN_ADC_112
1: TOF MS ES+
434.021 0.0500Da
2.81e5
3.47
434.02
100
%
0
-0.00
2.00
4.00
6.00
8.00
10.00
170612_SOE_LCMS_UN_ADC_112
1: TOF MS ES+
BPI
2.81e5
3.47
434.02
%
0.32
102.03
0.36
226.95
2.13
186.96
6.47
111.02
7.54
111.02
8.03
111.02
8.60
111.02
1
170612_SOE_LCMS_UN_ADC_112 Sm (Mn, 1x3)
3: Diode Array
254 2.0000Da
Range: 2.974
3.47
233.82
Time Height Area Area%
2.27 755 15.86 0.03
2.53 190 7.24 0.01
2.79 9370 487.10 0.85
2.95 1067 33.79 0.06
3.11 2201 78.71 0.14
3.26 1063 19.06 0.03
3.47 2961919 56672.27 98.81
3.79 364 38.88 0.07
2.5
2.0
1.5
1.0
5.0e-1
0.0
AU
2.79
218.82
Time

Compound **1g**

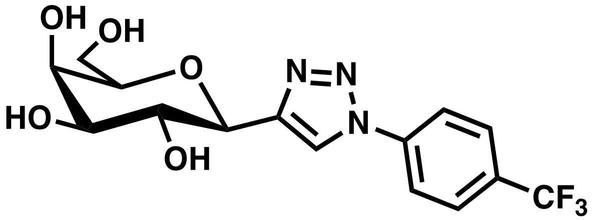

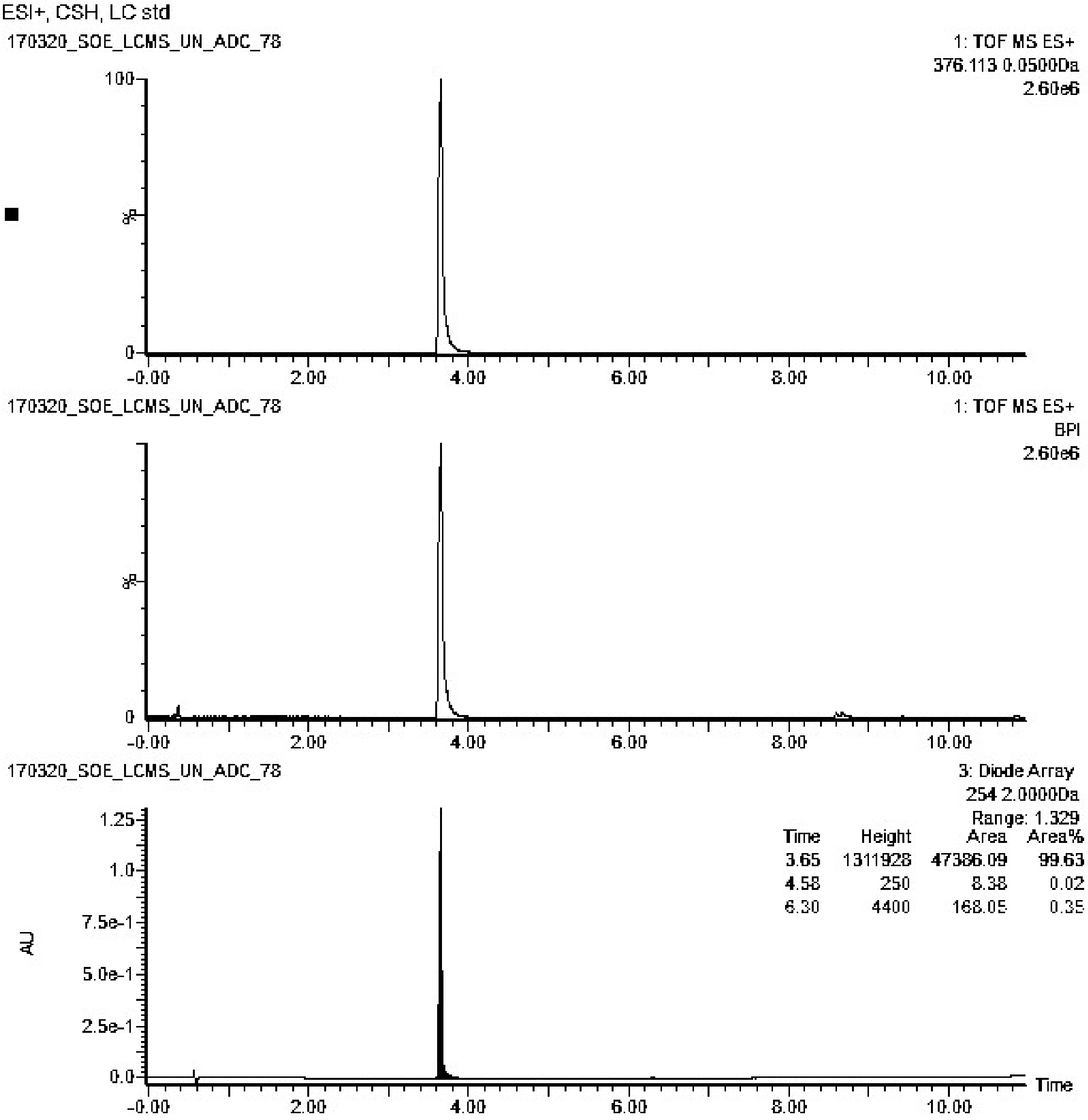
ESI+, CSH, LC std
170320_SOE_LCMS_UN_ADC_78
1: TOF MS ES+
376.113 0.0500Da
2.60e6
170320_SOE_LCMS_UN_ADC_78
1: TOF MS ES+
BPI
2.60e6
170320_SOE_LCMS_UN_ADC_78
3: Diode Array
254 2.0000Da
Range: 1.329
Time Height Area Area%
3.65 1311928 47386.09 99.63
4.58 250 8.38 0.02
6.30 4400 168.05 0.35
AU
Time

Compound **1h**

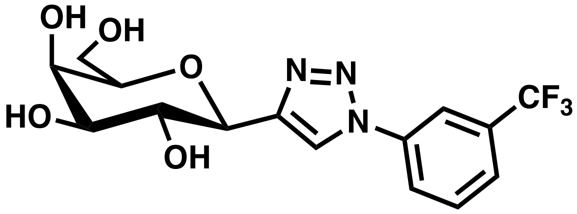
OH
OH
O
N=N
N
CF3
HO
OH

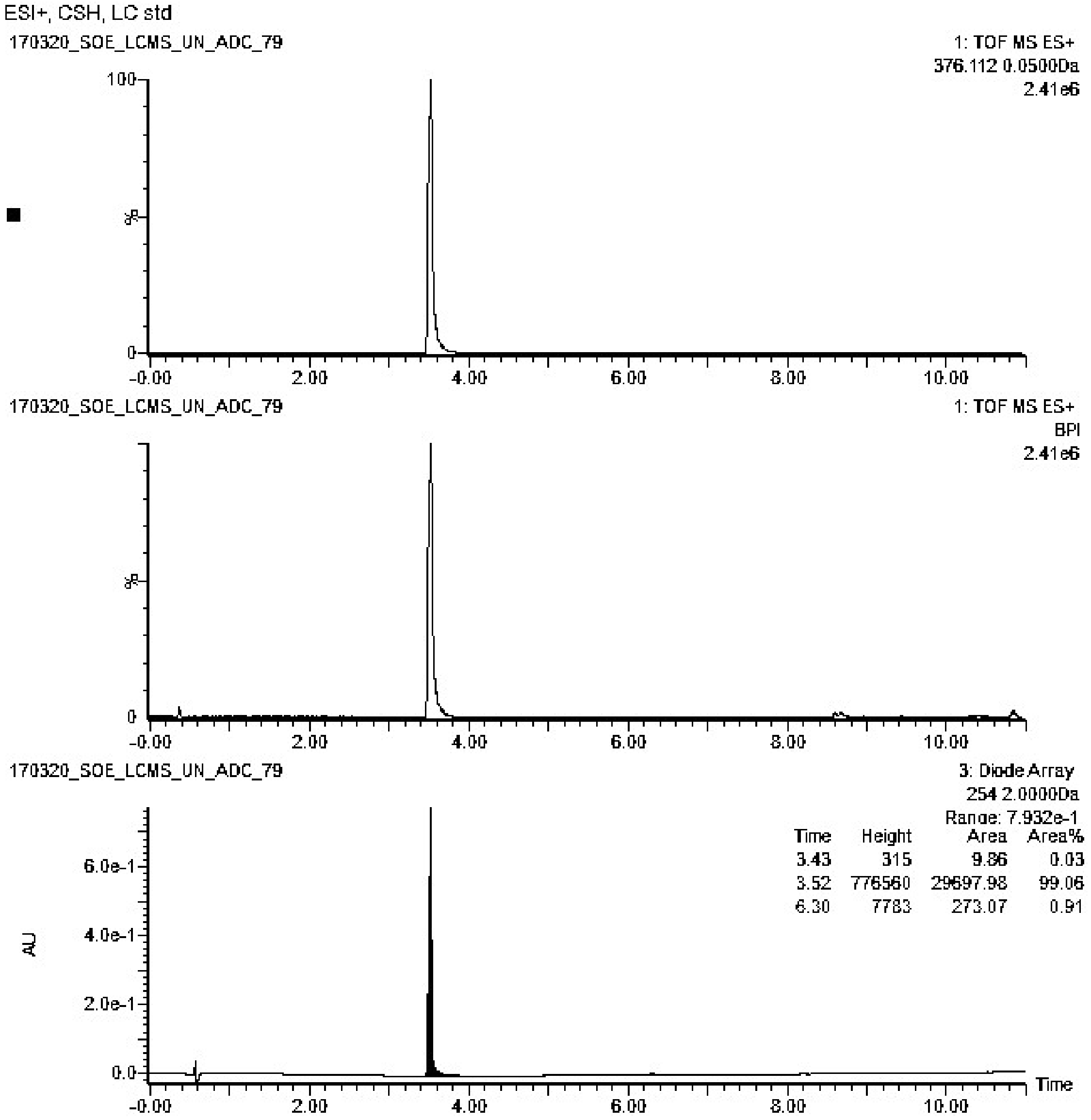
ESI+, CSH, LC std
170320_SOE_LCMS_UN_ADC_79
1: TOF MS ES+
376.112 0.0500Da
2.41e6
100
%
0
-0.00
2.00
4.00
6.00
8.00
10.00
170320_SOE_LCMS_UN_ADC_79
1: TOF MS ES+
BPI
2.41e6
%
0
-0.00
2.00
4.00
6.00
8.00
10.00
170320_SOE_LCMS_UN_ADC_79
3: Diode Array
254 2.0000Da
Range: 7.932e-1
Time Height Area Area%
3.43 315 9.86 0.03
3.52 776560 29697.98 99.06
6.30 7783 273.07 0.91
6.0e-1
4.0e-1
2.0e-1
0.0
AU
-0.00
2.00
4.00
6.00
8.00
10.00
Time

Compound **1i**

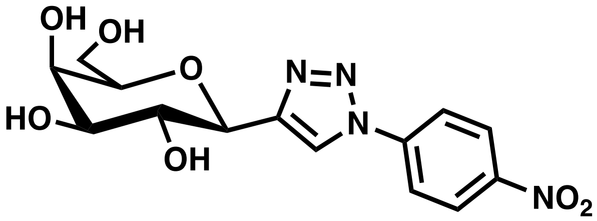
OH
OH
O
N=N
N
HO
OH
NO2

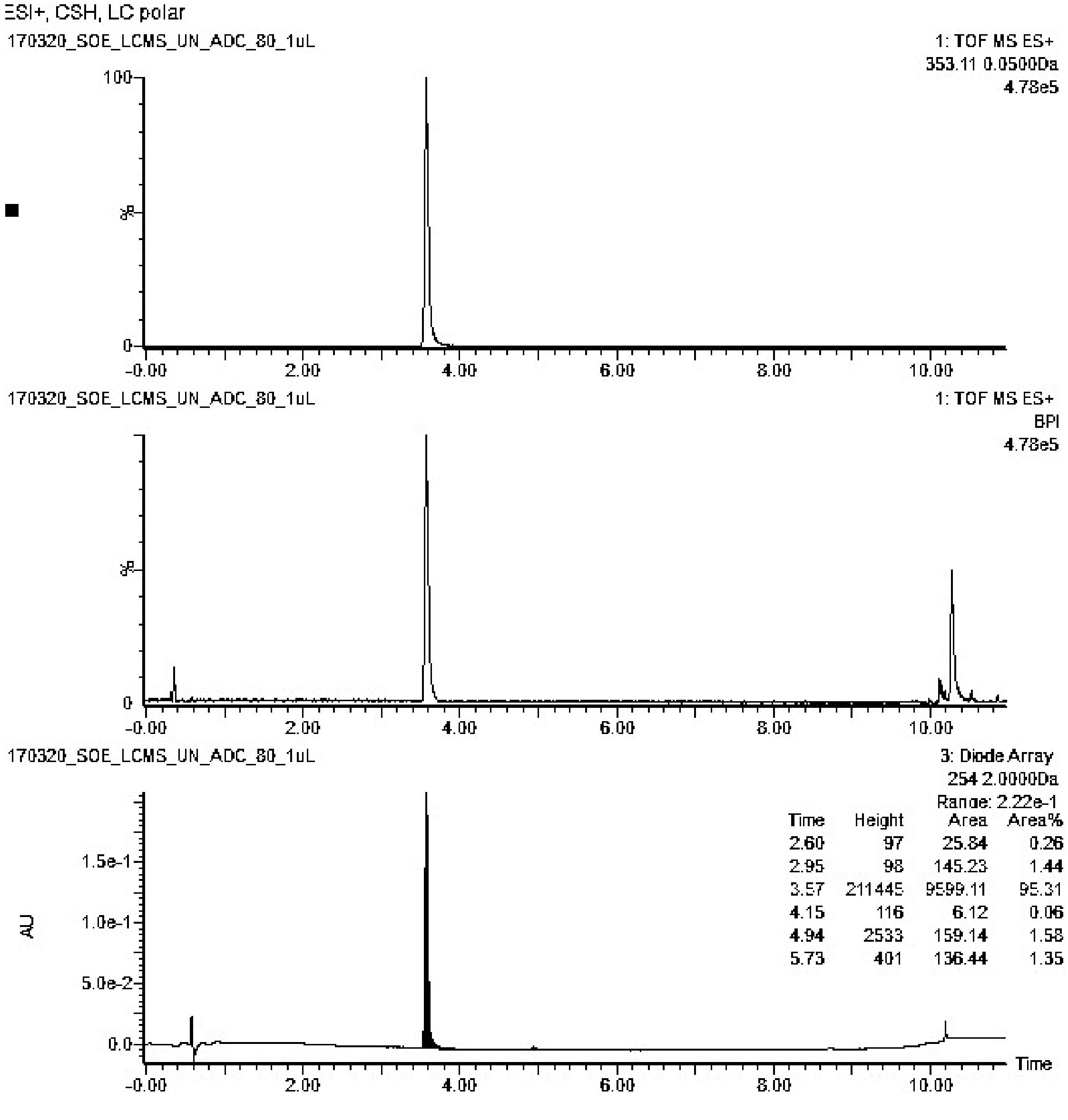
ESI+, CSH, LC polar
170320_SOE_LCMS_UN_ADC_80_1uL
1: TOF MS ES+
353.11 0.0500Da
4.78e5
100
%
0
-0.00 2.00 4.00 6.00 8.00 10.00
170320_SOE_LCMS_UN_ADC_80_1uL
1: TOF MS ES+
BPI
4.78e5
%
0
-0.00 2.00 4.00 6.00 8.00 10.00
170320_SOE_LCMS_UN_ADC_80_1uL
3: Diode Array
254 2.0000Da
Range: 2.22e-1
Time Height Area Area%
2.60 97 25.84 0.26
2.95 98 145.23 1.44
3.57 211445 9599.11 95.31
4.15 116 6.12 0.06
4.94 2533 159.14 1.58
5.73 401 136.44 1.35
AU
1.5e-1
1.0e-1
5.0e-2
0.0
Time
-0.00 2.00 4.00 6.00 8.00 10.00

Compound **1j**

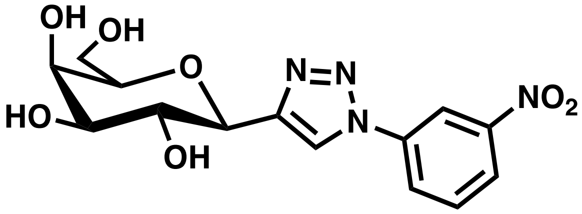

ESI+, CSH, LC polar

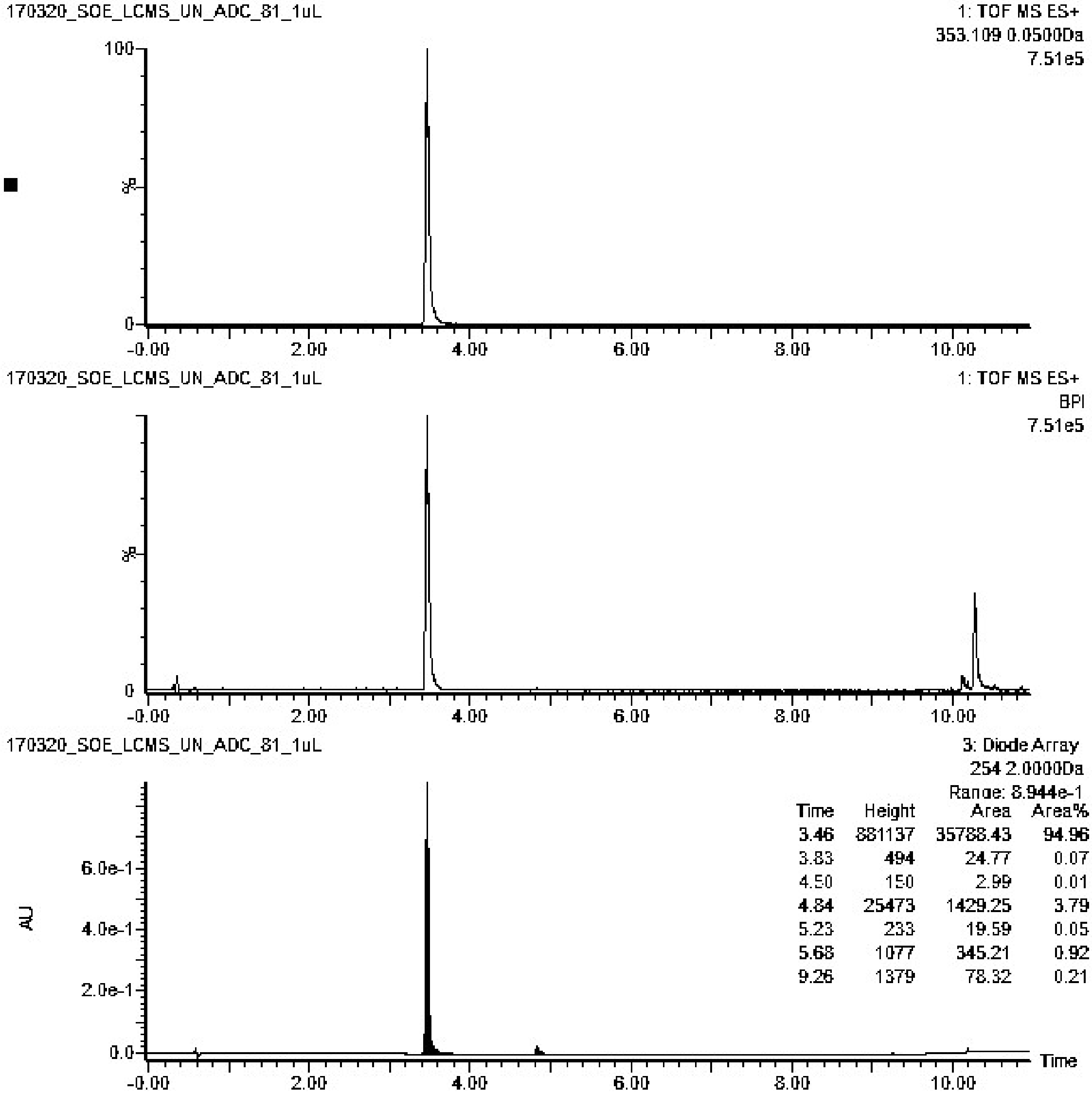

Compound **1k**

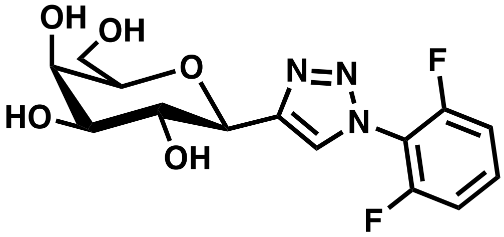

OH
OH
O
HO
OH
N=N
N
F
F

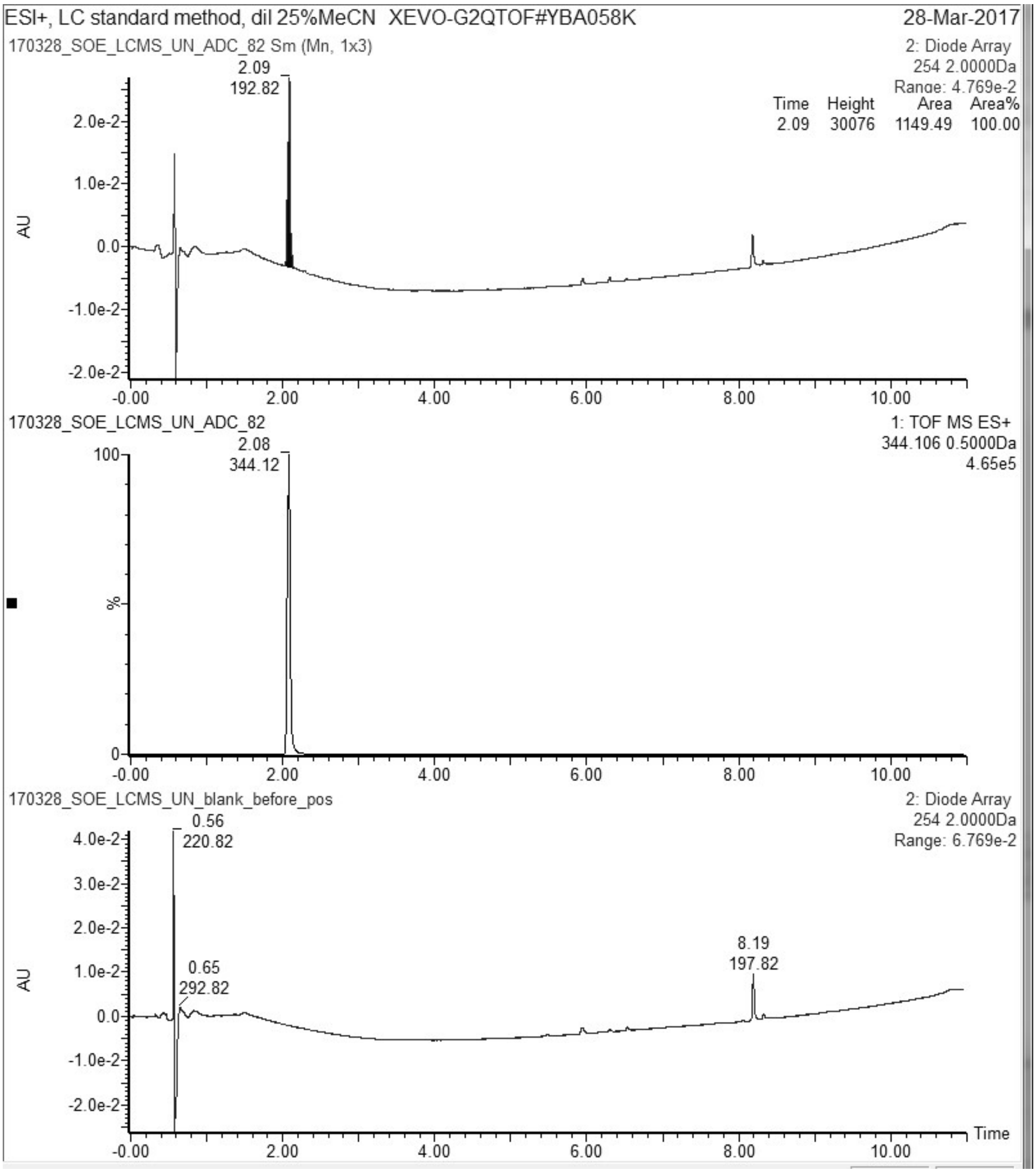

ESI+, LC standard method, dil 25%MeCN XEVO-G2QTOF#YBA058K
28-Mar-2017
170328_SOE_LCMS_UN_ADC_82 Sm (Mn, 1x3)
2: Diode Array
254 2.0000Da
Range: 4.769e-2
Time Height Area Area%
2.09 30076 1149.49 100.00
2.09
192.82
AU
170328_SOE_LCMS_UN_ADC_82
1: TOF MS ES+
344.106 0.5000Da
4.65e5
2.08
344.12
%
170328_SOE_LCMS_UN_blank_before_pos
2: Diode Array
254 2.0000Da
Range: 6.769e-2
0.56
220.82
0.65
292.82
8.19
197.82
Time

Compound **1l**

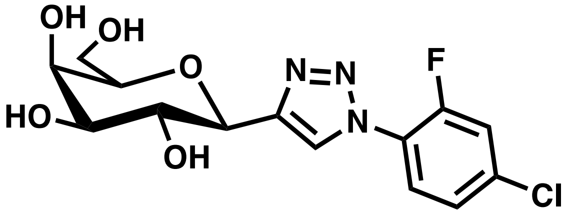

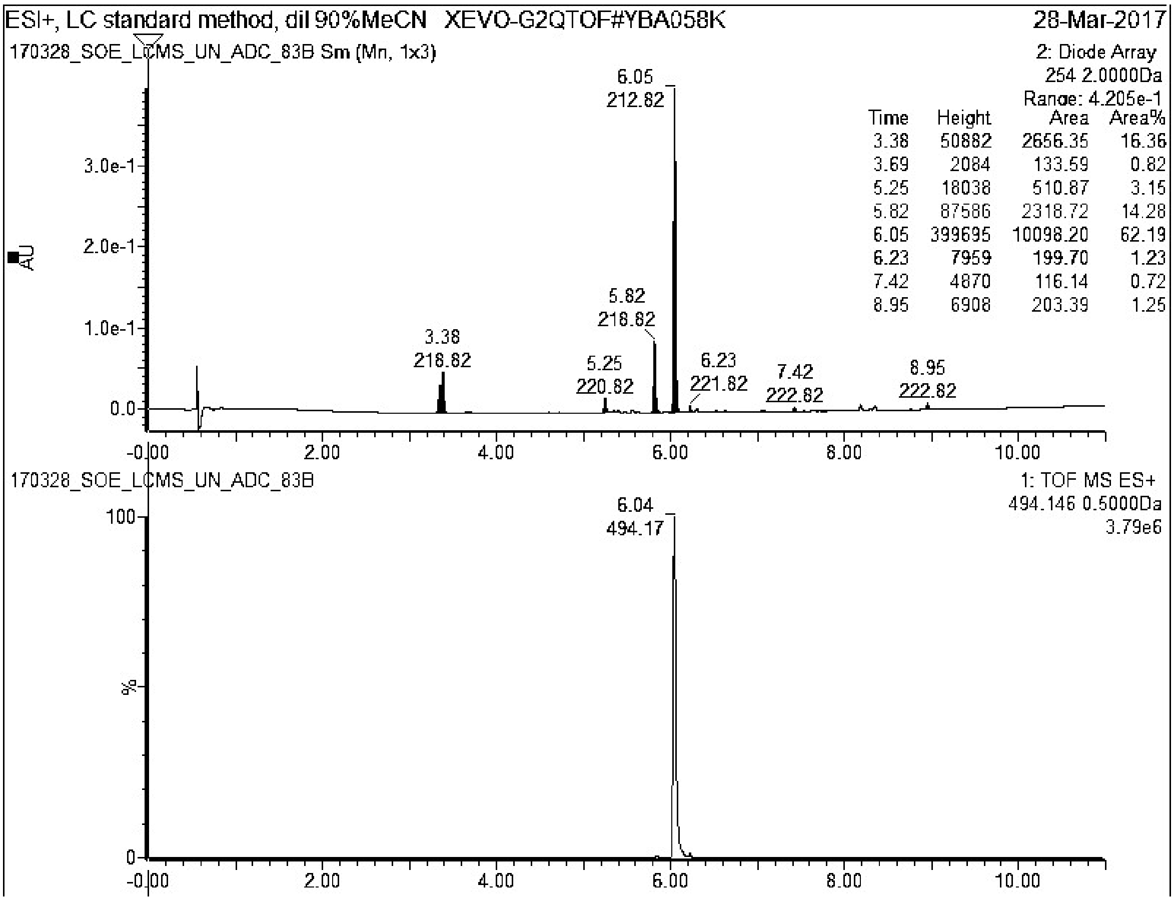

ESI+, LC standard method, dil 90%MeCN XEVO-G2QTOF#YBA058K
28-Mar-2017
170328_SOE_LCMS_UN_ADC_83B Sm (Mn, 1x3)
2: Diode Array
254 2.0000Da
Range: 4.205e-1
Time Height Area Area%
3.38 50882 2656.35 16.36
3.69 2084 133.59 0.82
5.25 18038 510.87 3.15
5.82 87586 2318.72 14.28
6.05 399695 10098.20 62.19
6.23 7959 199.70 1.23
7.42 4870 116.14 0.72
8.95 6908 203.39 1.25
AU
6.05
212.82
5.82
218.82
3.38
218.82
5.25
220.82
6.23
221.82
7.42
222.82
8.95
222.82
170328_SOE_LCMS_UN_ADC_83B
1: TOF MS ES+
494.146 0.5000Da
3.79e6
6.04
494.17
%

Compound **1m**

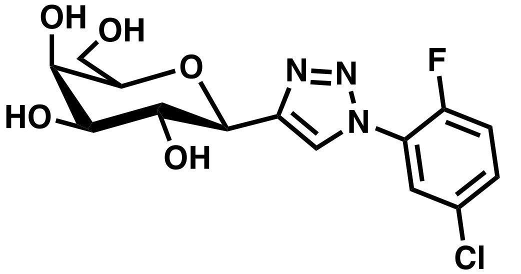

ESI+, CSH, LC std

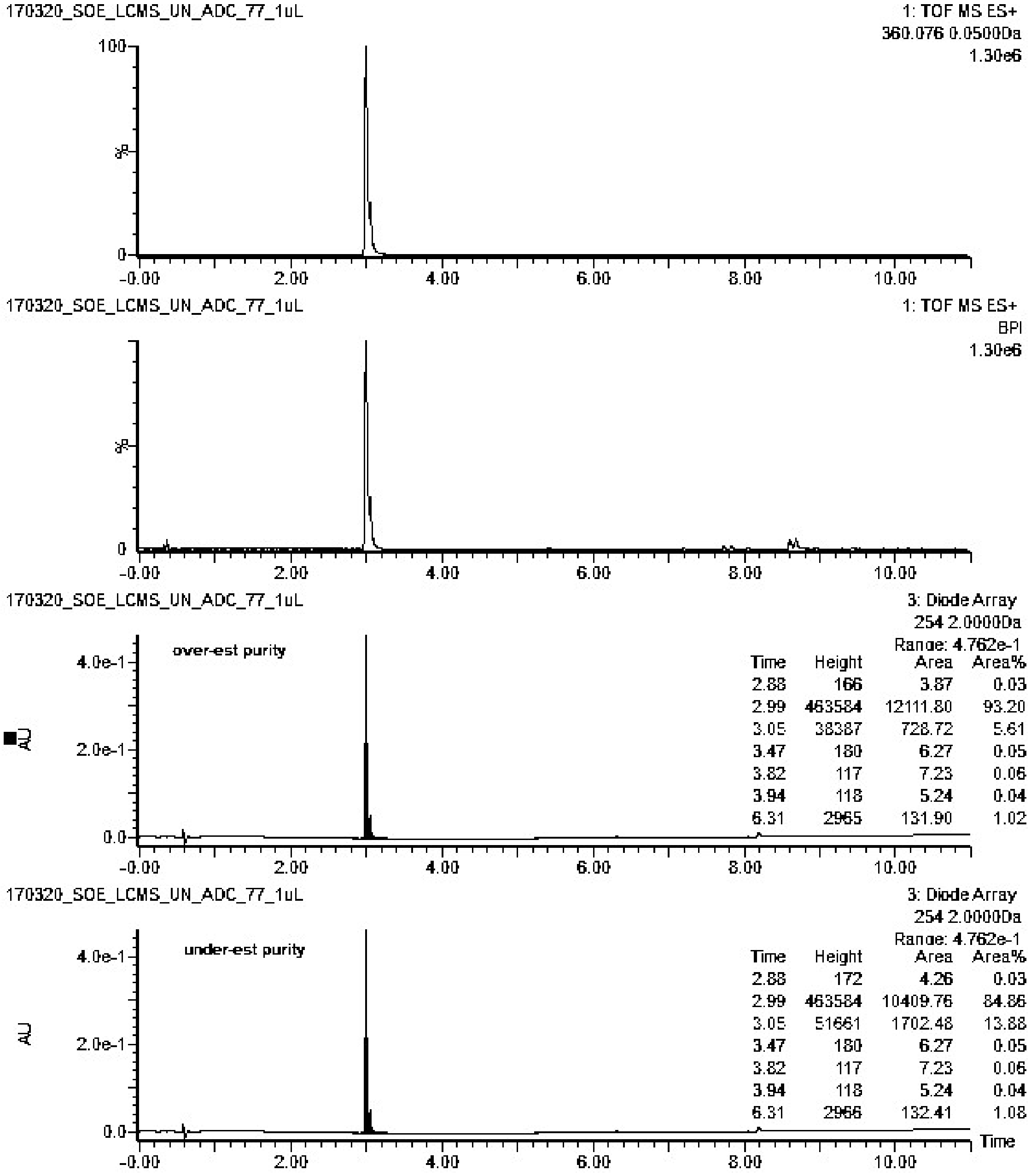

over-est purity

Time	Height	Area	Area%
2.88	166	3.87	0.03
2.99	463584	12111.80	93.20
3.05	38387	728.72	5.61
3.47	180	6.27	0.05
3.82	117	7.23	0.06
3.94	118	5.24	0.04
6.31	2965	131.90	1.02

under-est purity

Time	Height	Area	Area%
2.88	172	4.26	0.03
2.99	463584	10409.76	84.86
3.05	51661	1702.48	13.88
3.47	180	6.27	0.05
3.82	117	7.23	0.06
3.94	118	5.24	0.04
6.31	2966	132.41	1.08

Compound **1n**

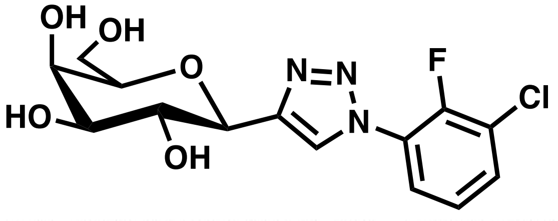

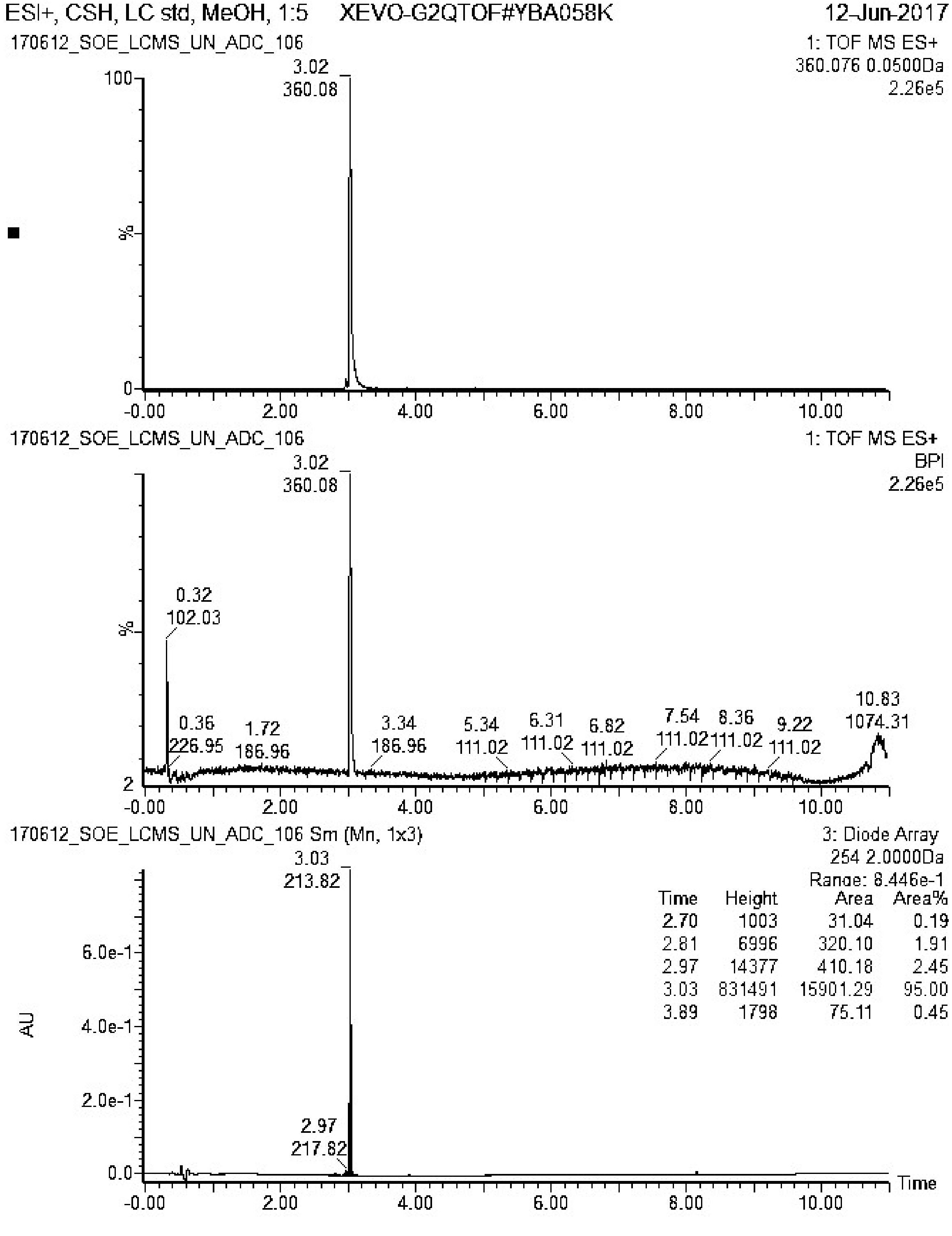

ESI+, CSH, LC std, MeOH, 1:5 XEVO-G2QTOF#YBA058K
12-Jun-2017
170612_SOE_LCMS_UN_ADC_106
1: TOF MS ES+
360.076 0.0500Da
2.26e5
3.02
360.08
170612_SOE_LCMS_UN_ADC_106
1: TOF MS ES+
BPI
2.26e5
0.32
102.03
0.36
226.95
1.72
186.96
3.34
186.96
5.34
111.02
6.31
111.02
6.82
111.02
7.54
111.02
8.36
111.02
9.22
111.02
10.83
1074.31
170612_SOE_LCMS_UN_ADC_106 Sm (Mn, 1x3)
3: Diode Array
254 2.0000Da
Range: 8.446e-1
3.03
213.82
2.97
217.82
Time Height Area Area%
2.70 1003 31.04 0.19
2.81 6996 320.10 1.91
2.97 14377 410.18 2.45
3.03 831491 15901.29 95.00
3.89 1798 75.11 0.45
AU
Time

Compound **1o**

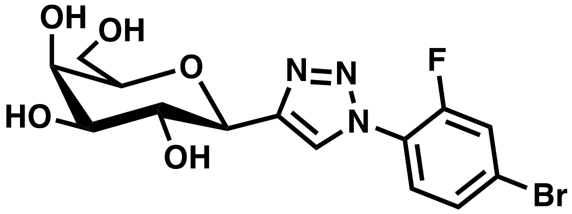
OH
OH
O
HO
OH
N=N
N
F
Br

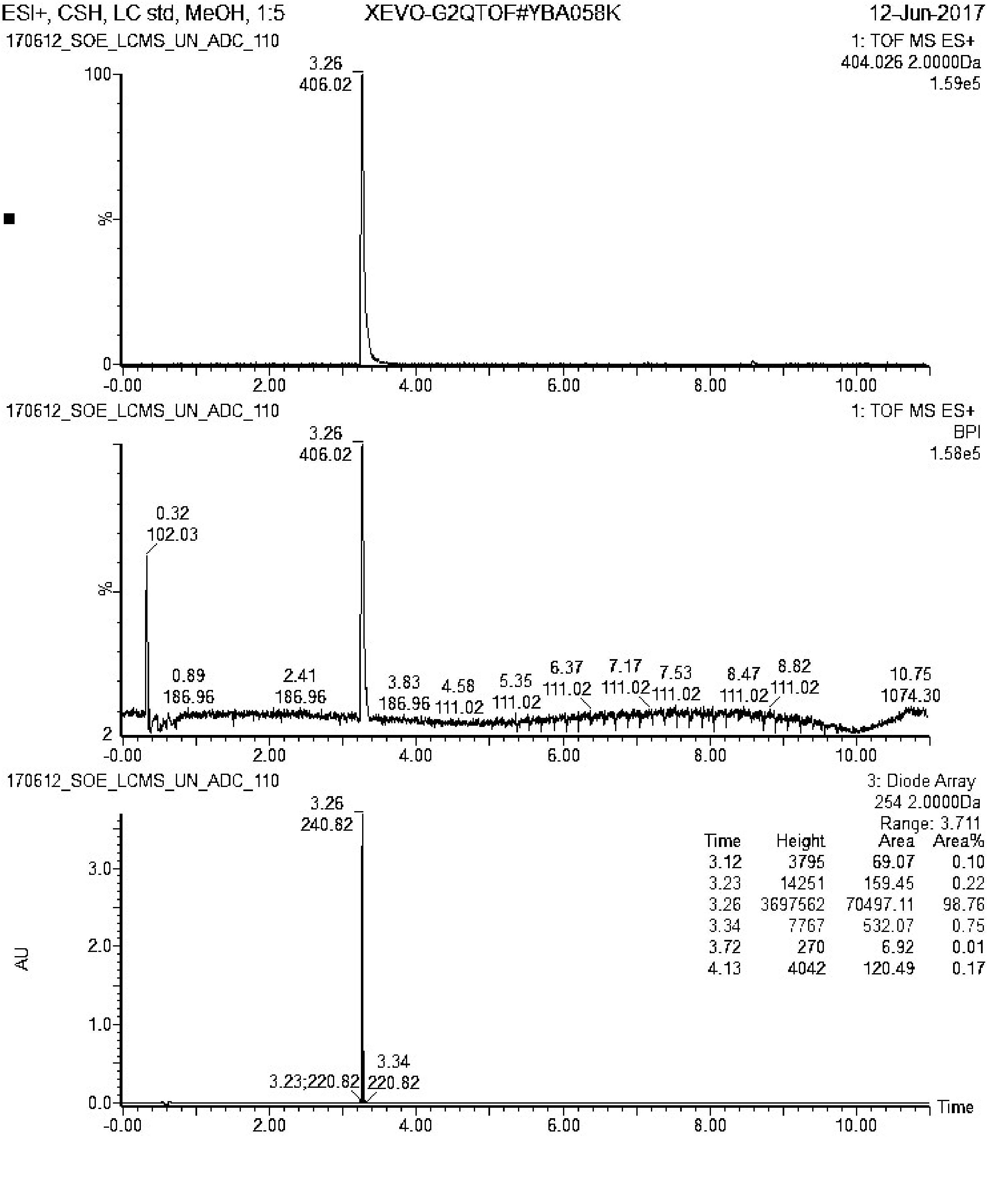
ESI+, CSH, LC std, MeOH, 1:5
XEVO-G2QTOF#YBA058K
12-Jun-2017
170612_SOE_LCMS_UN_ADC_110
1: TOF MS ES+
404.026 2.0000Da
1.59e5
3.26
406.02
100
%
0
-0.00
2.00
4.00
6.00
8.00
10.00
170612_SOE_LCMS_UN_ADC_110
1: TOF MS ES+
BPI
1.58e5
3.26
406.02
0.32
102.03
0.89
186.96
2.41
186.96
3.83
186.96
4.58
111.02
5.35
111.02
6.37
111.02
7.17
111.02
7.53
111.02
8.47
111.02
8.82
111.02
10.75
1074.30
2
170612_SOE_LCMS_UN_ADC_110
3: Diode Array
254 2.0000Da
Range: 3.711
3.26
240.82
Time Height Area Area%
3.12 3795 69.07 0.10
3.23 14251 159.45 0.22
3.26 3697562 70497.11 98.76
3.34 7767 532.07 0.75
3.72 270 6.92 0.01
4.13 4042 120.49 0.17
3.0
2.0
1.0
0.0
AU
3.23;220.82
3.34
220.82
Time

Compound **1p**

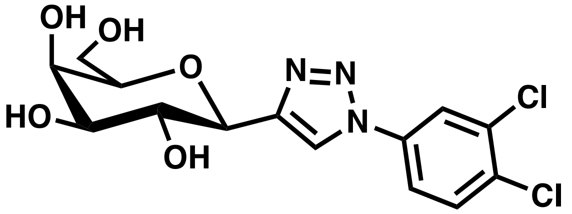

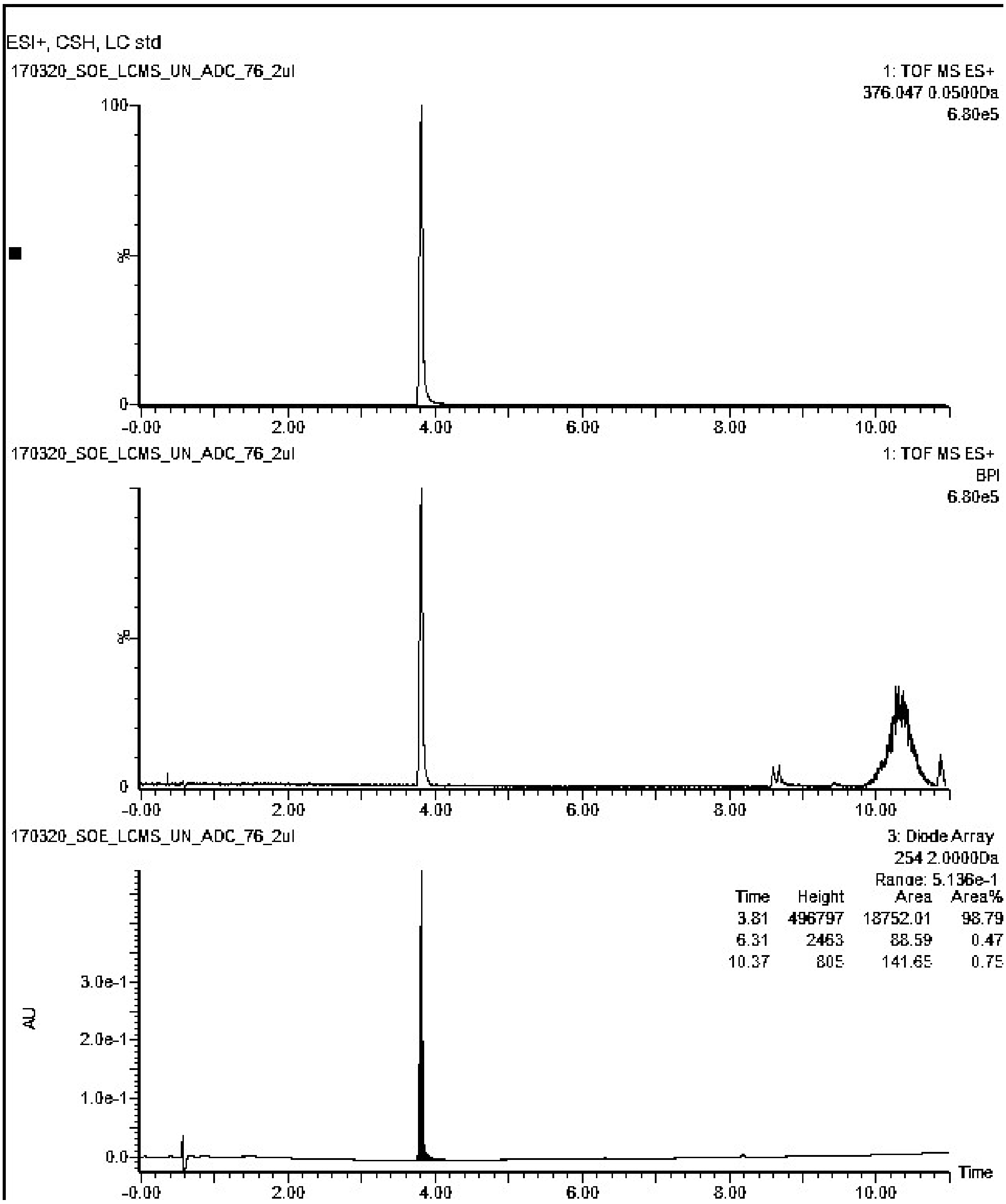
ESI+, CSH, LC std
170320_SOE_LCMS_UN_ADC_76_2ul
1: TOF MS ES+
376.047 0.0500Da
6.80e5
100
%
0
-0.00
2.00
4.00
6.00
8.00
10.00
170320_SOE_LCMS_UN_ADC_76_2ul
1: TOF MS ES+
BPI
6.80e5
%
0
170320_SOE_LCMS_UN_ADC_76_2ul
3: Diode Array
254 2.0000Da
Range: 5.136e-1
Time Height Area Area%
3.81 496797 18752.01 98.79
6.31 2463 88.59 0.47
10.37 805 141.65 0.75
AU
3.0e-1
2.0e-1
1.0e-1
0.0
Time

Compound **1q**

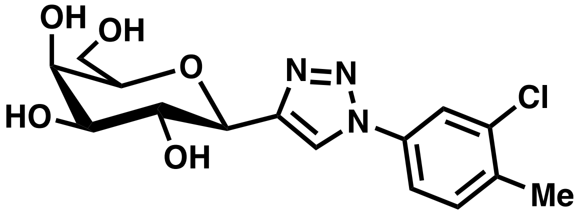
OH
OH
O
HO
OH
N=N
N
Cl
Me

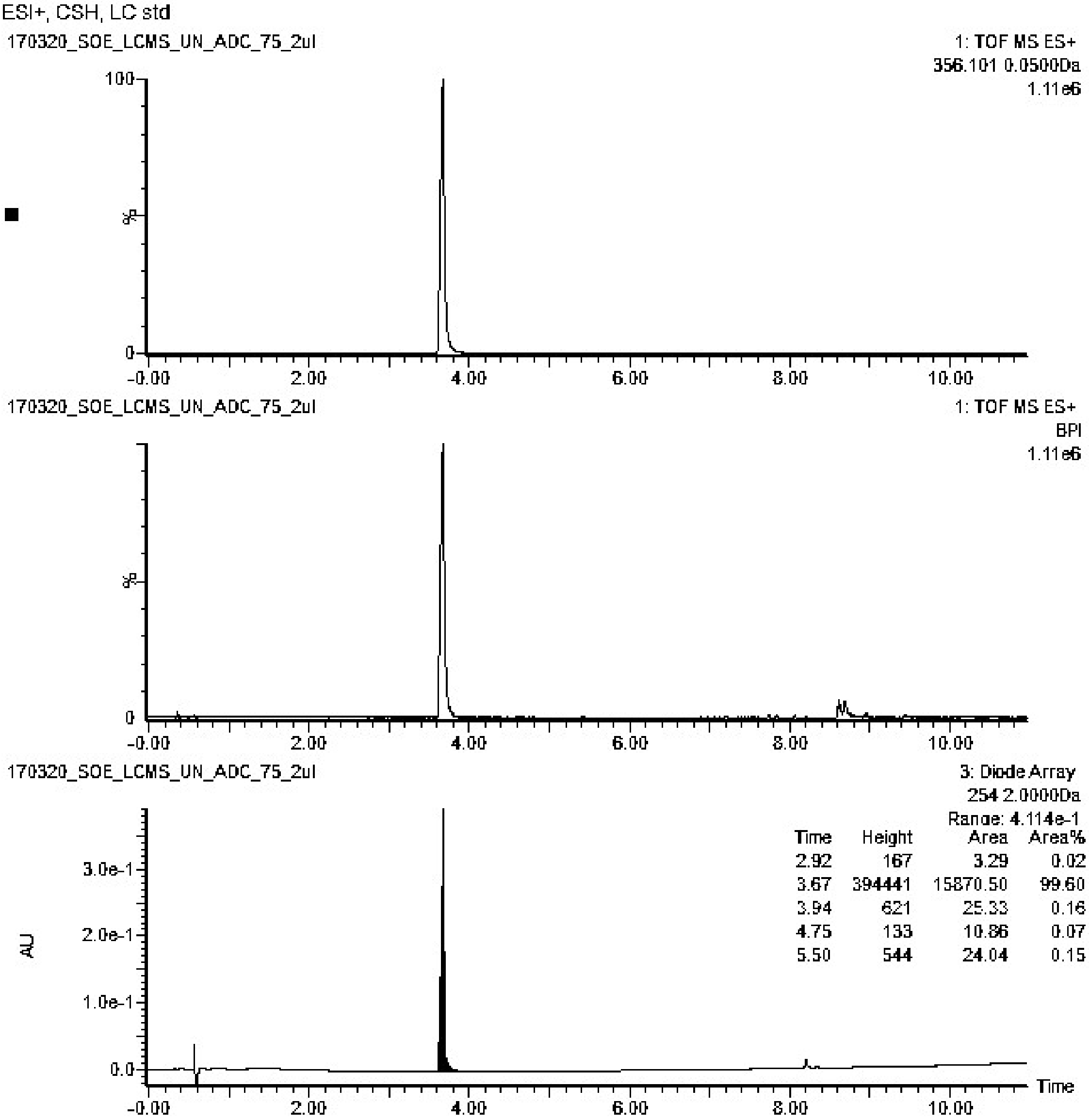
ESI+, CSH, LC std
170320_SOE_LCMS_UN_ADC_75_2ul
1: TOF MS ES+
356.101 0.0500Da
1.11e6
100
%
0
-0.00
2.00
4.00
6.00
8.00
10.00
170320_SOE_LCMS_UN_ADC_75_2ul
1: TOF MS ES+
BPI
1.11e6
%
0
170320_SOE_LCMS_UN_ADC_75_2ul
3: Diode Array
254 2.0000Da
Range: 4.114e-1
Time Height Area Area%
2.92 167 3.29 0.02
3.67 394441 15870.50 99.60
3.94 621 25.33 0.16
4.75 133 10.86 0.07
5.50 544 24.04 0.15
3.0e-1
2.0e-1
1.0e-1
0.0
AU
Time

Compound **1r**

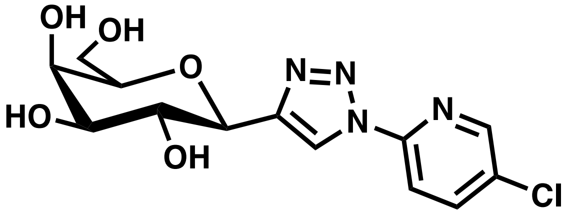
OH
OH
O
N=N
N
N
HO
OH
Cl

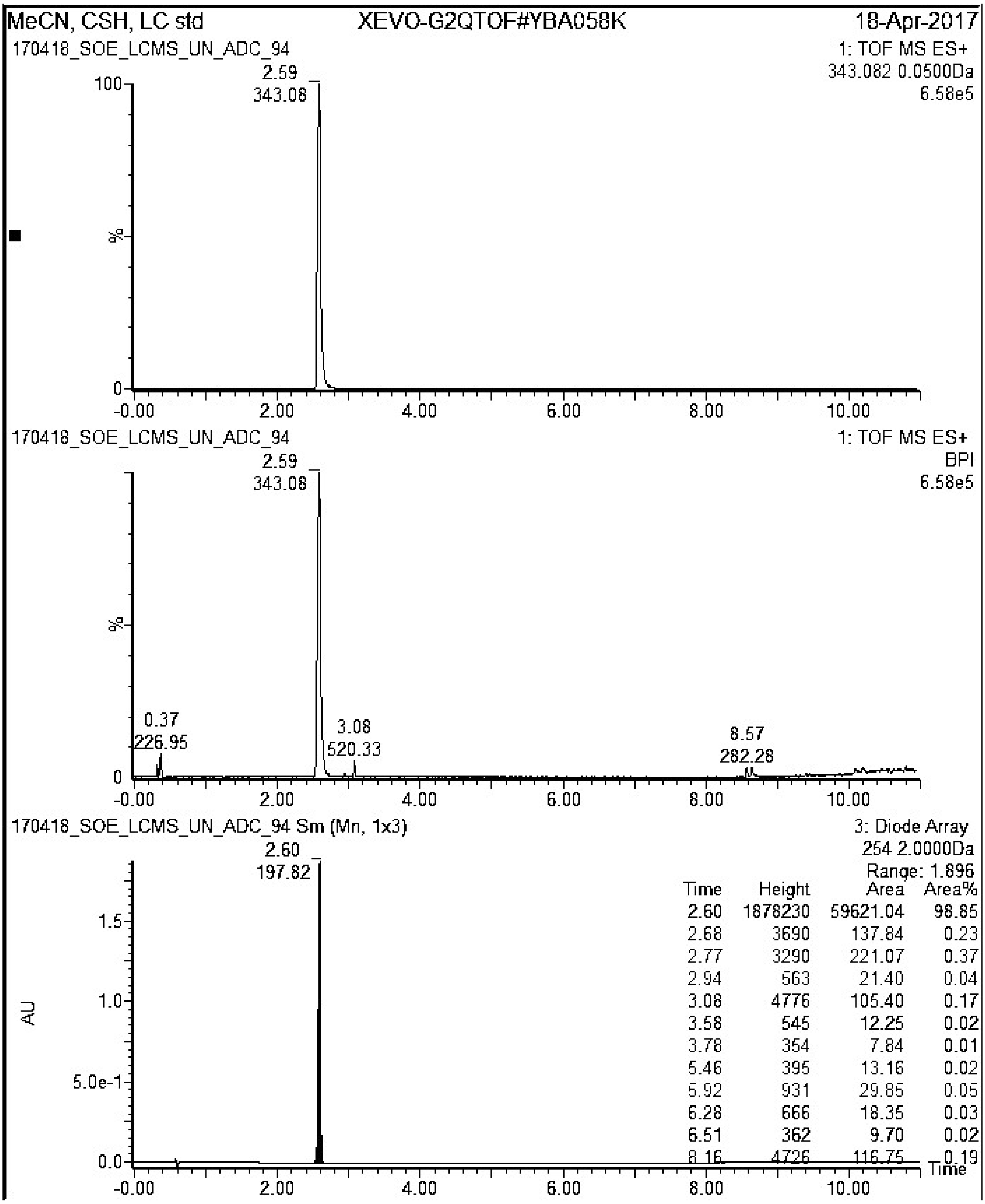
MeCN, CSH, LC std
XEVO-G2QTOF#YBA058K
18-Apr-2017
170418_SOE_LCMS_UN_ADC_94
1: TOF MS ES+
343.082 0.0500Da
6.58e5
2.59
343.08
%
170418_SOE_LCMS_UN_ADC_94
1: TOF MS ES+
BPI
6.58e5
2.59
343.08
0.37
226.95
3.08
520.33
8.57
282.28
170418_SOE_LCMS_UN_ADC_94 Sm (Mn, 1x3)
3: Diode Array
254 2.0000Da
Range: 1.896
2.60
197.82
AU
Time Height Area Area%
2.60 1878230 59621.04 98.85
2.68 3690 137.84 0.23
2.77 3290 221.07 0.37
2.94 563 21.40 0.04
3.08 4776 105.40 0.17
3.58 545 12.25 0.02
3.78 354 7.84 0.01
5.46 395 13.16 0.02
5.92 931 29.85 0.05
6.28 666 18.35 0.03
6.51 362 9.70 0.02
8.16 4726 116.75 0.19
Time
-0.00 2.00 4.00 6.00 8.00 10.00

Compound **1s**

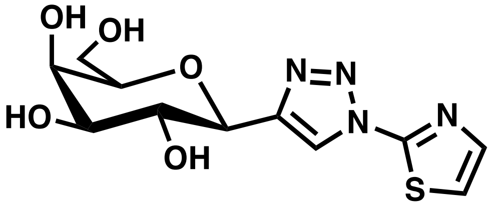

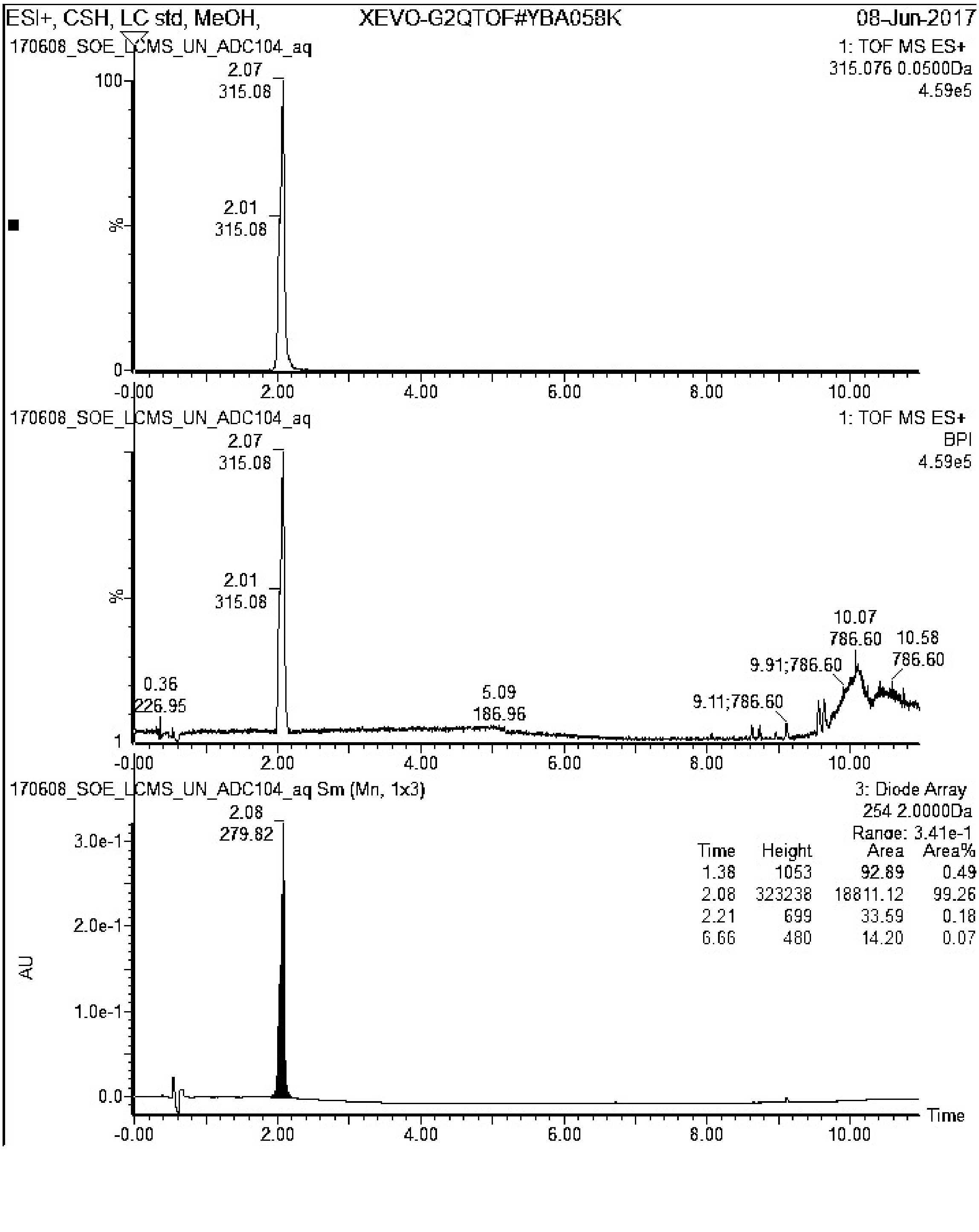

Time	Height	Area	Area%
1.38	1053	92.89	0.49
2.08	323238	18811.12	99.26
2.21	699	33.59	0.18
6.66	480	14.20	0.07

Compound **1t**

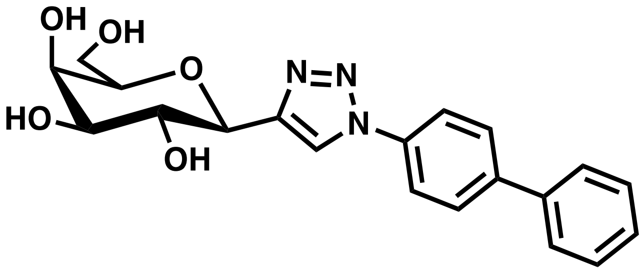
OH
OH
O
N=N
N
HO
OH

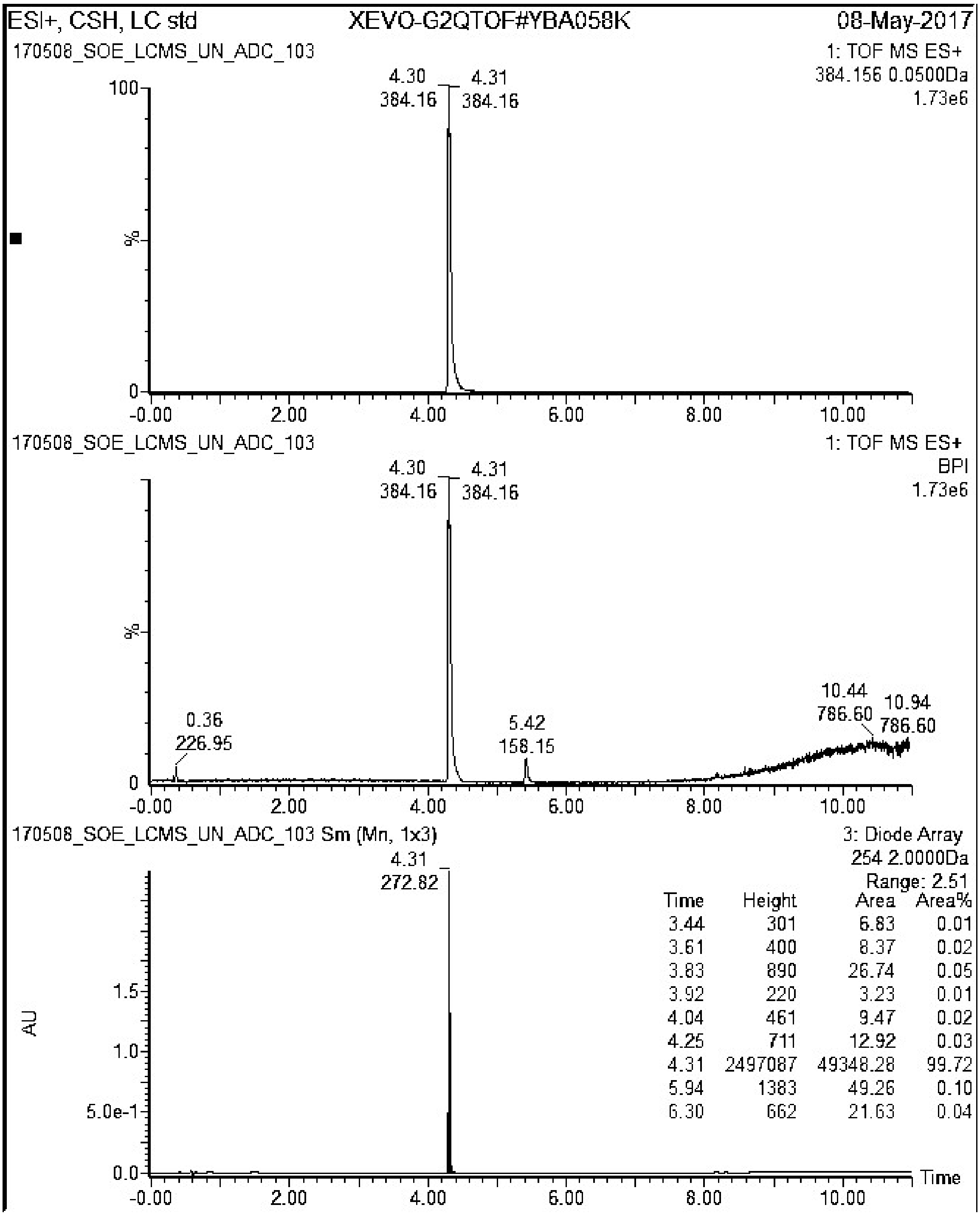
ESI+, CSH, LC std
XEVO-G2QTOF#YBA058K
08-May-2017
170508_SOE_LCMS_UN_ADC_103
1: TOF MS ES+
384.156 0.0500Da
1.73e6
4.30
384.16
4.31
384.16
170508_SOE_LCMS_UN_ADC_103
1: TOF MS ES+
BPI
1.73e6
0.36
226.95
5.42
158.15
10.44
786.60
10.94
786.60
170508_SOE_LCMS_UN_ADC_103 Sm (Mn, 1x3)
3: Diode Array
254 2.0000Da
Range: 2.51
4.31
272.82
Time Height Area Area%
3.44 301 6.83 0.01
3.61 400 8.37 0.02
3.83 890 26.74 0.05
3.92 220 3.23 0.01
4.04 461 9.47 0.02
4.25 711 12.92 0.03
4.31 2497087 49348.28 99.72
5.94 1383 49.26 0.10
6.30 662 21.63 0.04
AU
Time

Compound **1u**

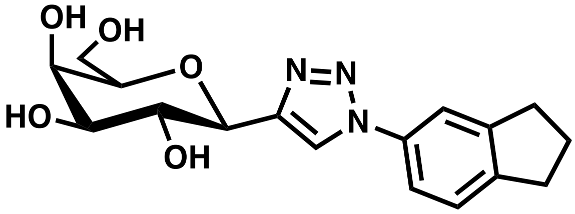

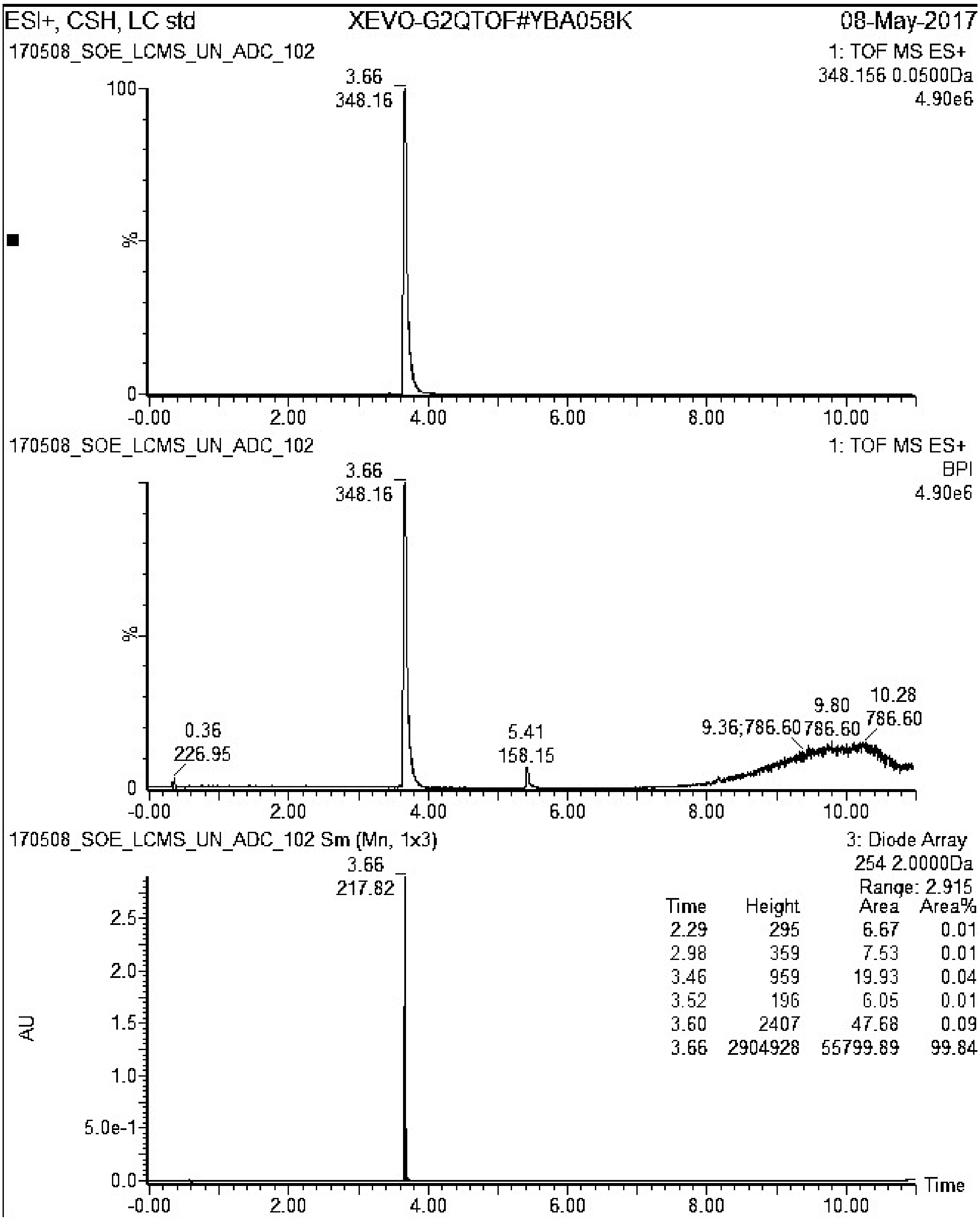
ESI+, CSH, LC std
XEVO-G2QTOF#YBA058K
08-May-2017
170508_SOE_LCMS_UN_ADC_102
1: TOF MS ES+
348.156 0.0500Da
4.90e6
3.66
348.16
170508_SOE_LCMS_UN_ADC_102
1: TOF MS ES+
BPI
4.90e6
3.66
348.16
0.36
226.95
5.41
158.15
9.36;786.60
9.80
786.60
10.28
786.60
170508_SOE_LCMS_UN_ADC_102 Sm (Mn, 1x3)
3: Diode Array
254 2.0000Da
Range: 2.915
3.66
217.82
Time Height Area Area%
2.29 295 6.67 0.01
2.98 359 7.53 0.01
3.46 959 19.93 0.04
3.52 196 6.05 0.01
3.60 2407 47.68 0.09
3.66 2904928 55799.89 99.84
AU
Time

Compound **1v**

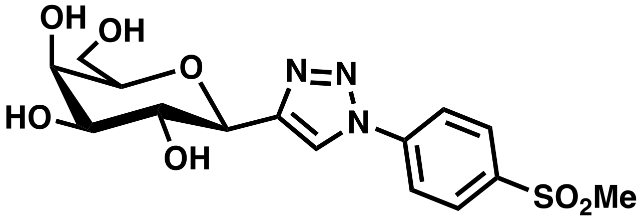
OH
OH
O
N=N
N
HO
OH
SO_2Me

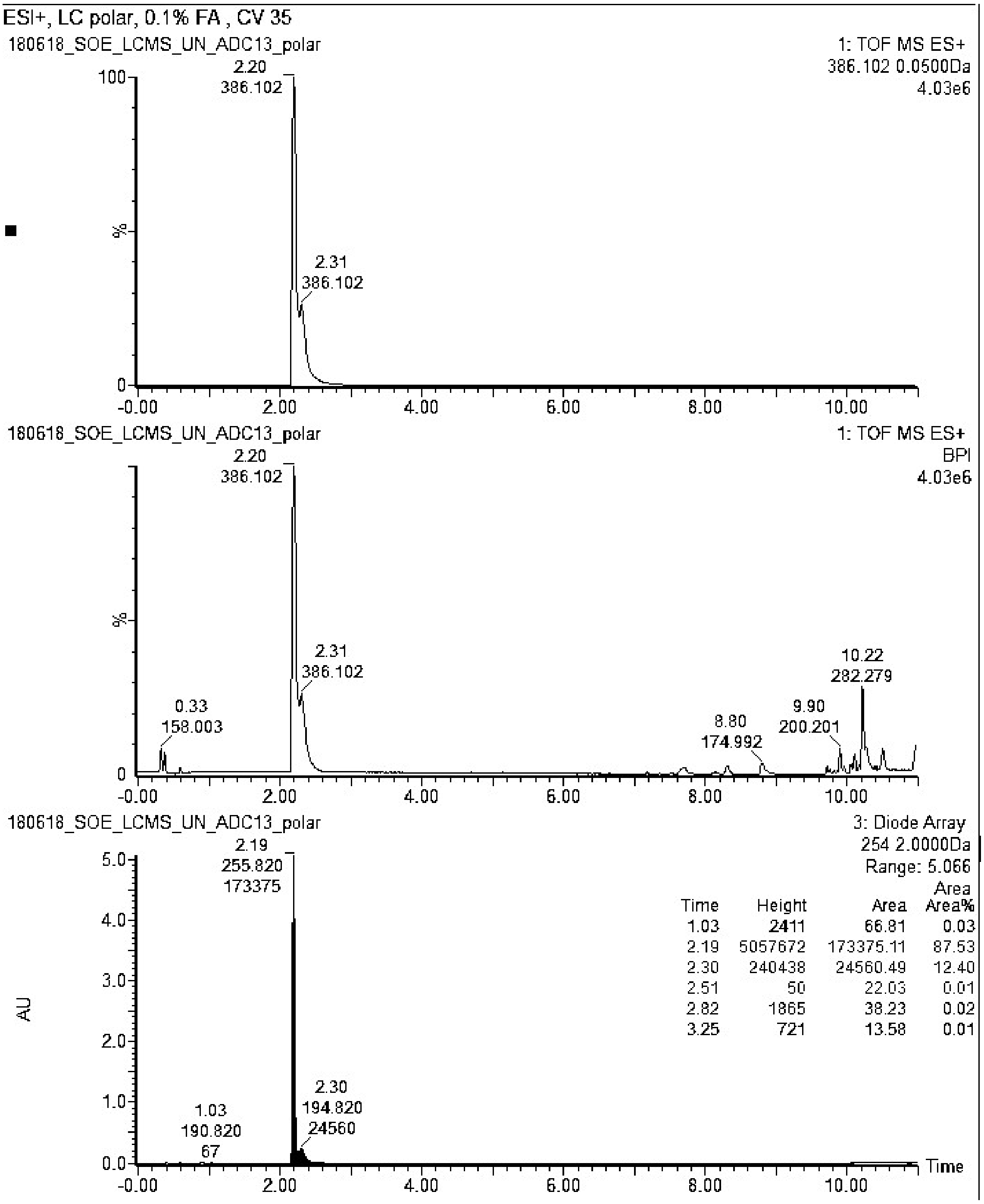
ESI+, LC polar, 0.1% FA , CV 35
180618_SOE_LCMS_UN_ADC13_polar
1: TOF MS ES+
386.102 0.0500Da
4.03e6
2.20
386.102
2.31
386.102
100
%
0
-0.00
2.00
4.00
6.00
8.00
10.00
180618_SOE_LCMS_UN_ADC13_polar
1: TOF MS ES+
BPI
4.03e6
2.20
386.102
2.31
386.102
0.33
158.003
8.80
174.992
9.90
200.201
10.22
282.279
%
0
-0.00
2.00
4.00
6.00
8.00
10.00
180618_SOE_LCMS_UN_ADC13_polar
3: Diode Array
254 2.0000Da
Range: 5.066
2.19
255.820
173375
2.30
194.820
24560
1.03
190.820
67
Time Height Area Area Area%
1.03 2411 66.81 0.03
2.19 5057672 173375.11 87.53
2.30 240438 24560.49 12.40
2.51 50 22.03 0.01
2.82 1865 38.23 0.02
3.25 721 13.58 0.01
AU
5.0
4.0
3.0
2.0
1.0
0.0
-0.00
2.00
4.00
6.00
8.00
10.00
Time

Compound **1w**

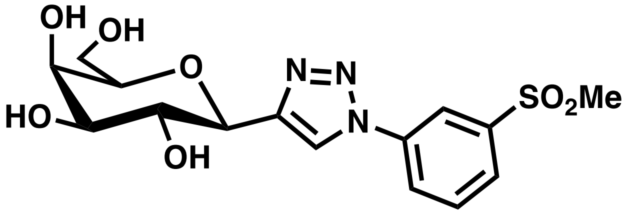
OH
OH
O
N=N
N
SO2Me
HO
OH

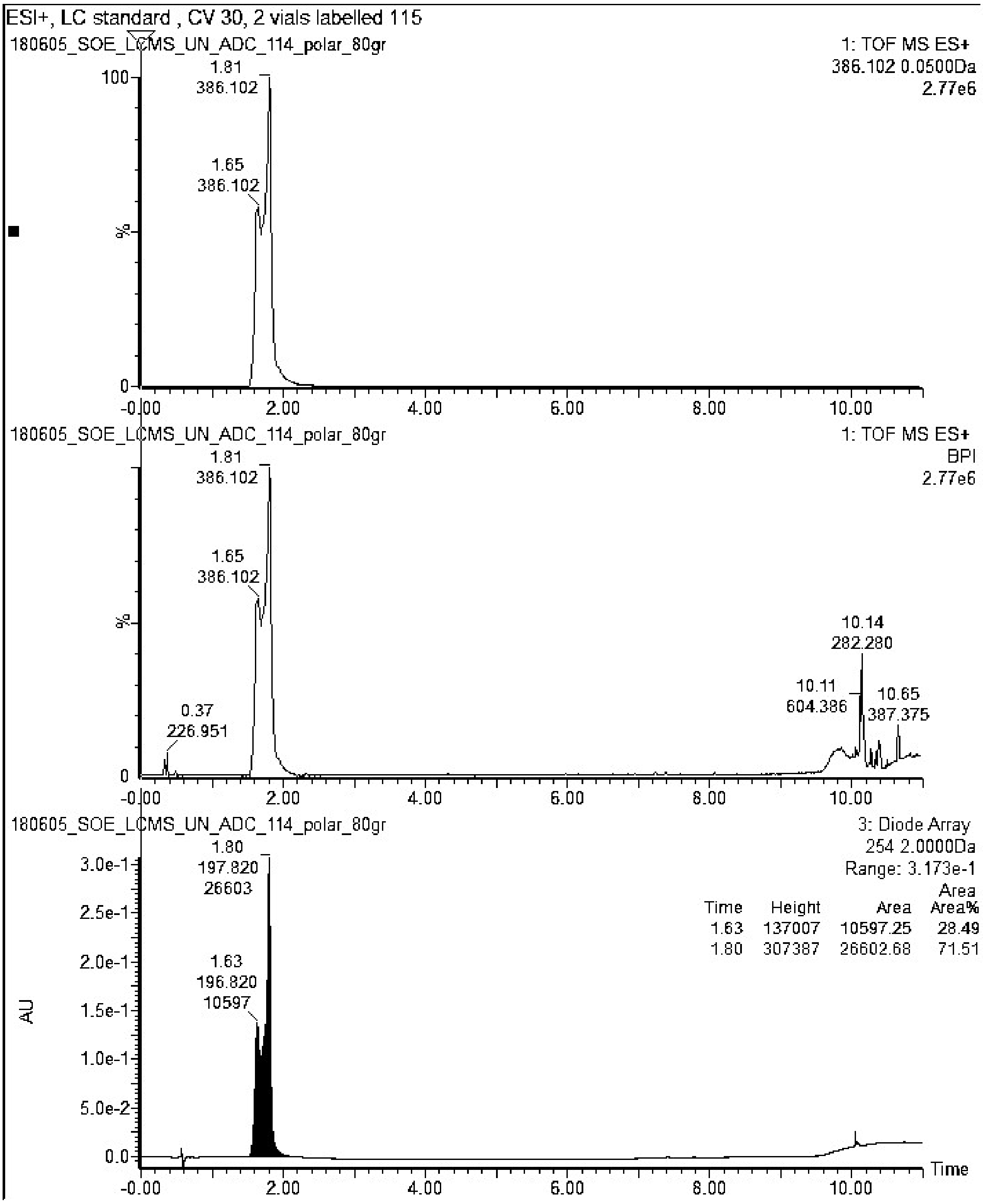
ESI+, LC standard , CV 30, 2 vials labelled 115
180605_SOE_LCMS_UN_ADC_114_polar_80gr
1: TOF MS ES+
386.102 0.0500Da
2.77e6
1.81
386.102
1.65
386.102
100
%
0
-0.00
2.00
4.00
6.00
8.00
10.00
180605_SOE_LCMS_UN_ADC_114_polar_80gr
1: TOF MS ES+
BPI
2.77e6
1.81
386.102
1.65
386.102
0.37
226.951
10.14
282.280
10.11
604.386
10.65
387.375
180605_SOE_LCMS_UN_ADC_114_polar_80gr
3: Diode Array
254 2.0000Da
Range: 3.173e-1
1.80
197.820
26603
1.63
196.820
10597
Time Height Area Area%
1.63 137007 10597.25 28.49
1.80 307387 26602.68 71.51
3.0e-1
2.5e-1
2.0e-1
1.5e-1
1.0e-1
5.0e-2
0.0
AU
Time

Compound **1x**

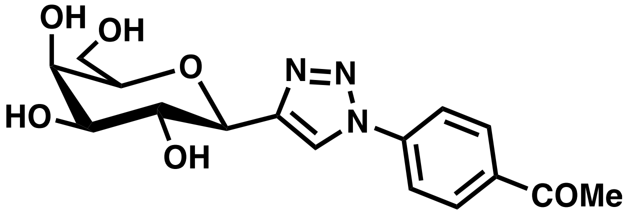

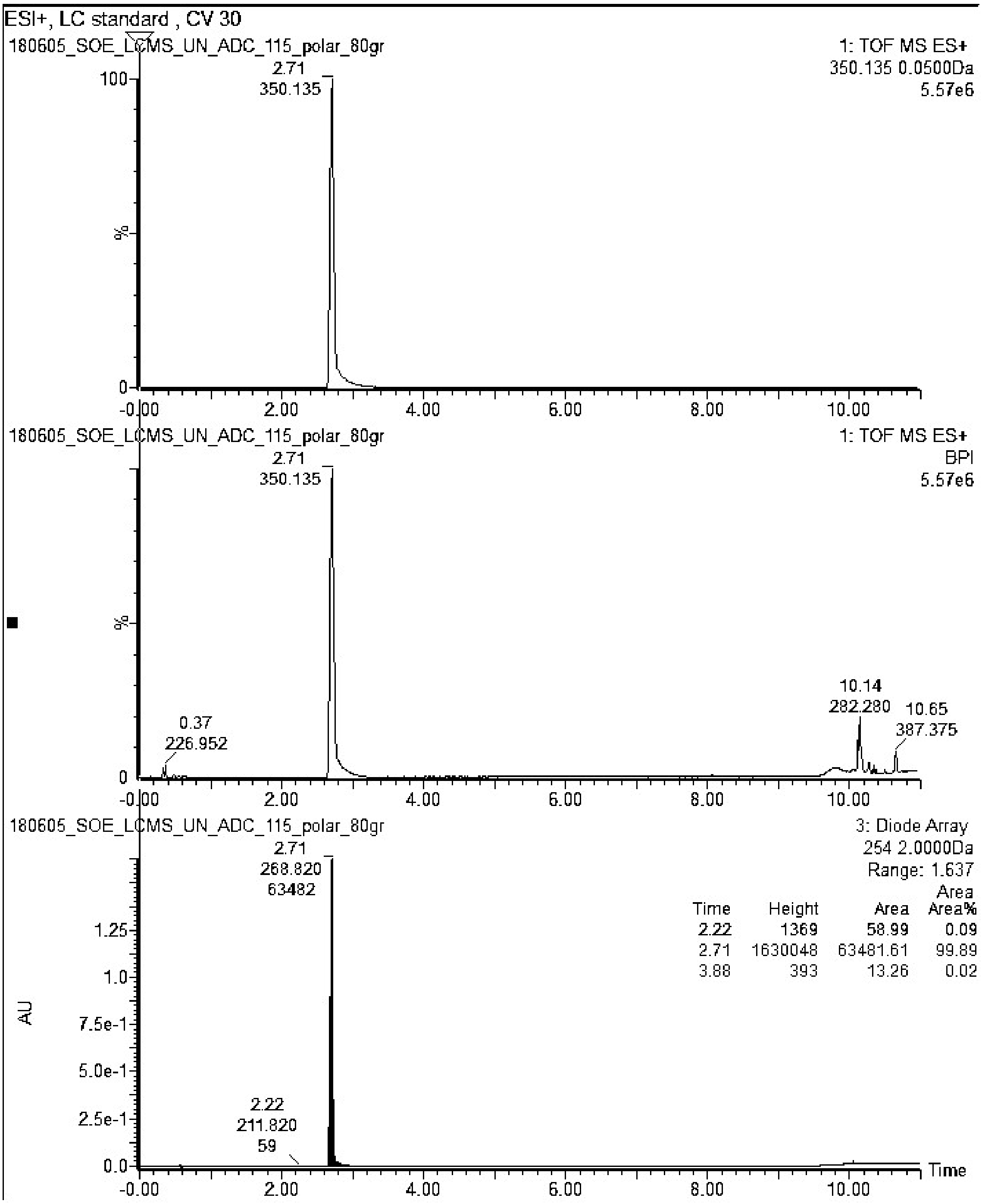
ESI+, LC standard , CV 30
180605_SOE_LCMS_UN_ADC_115_polar_80gr
1: TOF MS ES+
350.135 0.0500Da
5.57e6
2.71
350.135
180605_SOE_LCMS_UN_ADC_115_polar_80gr
1: TOF MS ES+
BPI
5.57e6
2.71
350.135
0.37
226.952
10.14
282.280
10.65
387.375
180605_SOE_LCMS_UN_ADC_115_polar_80gr
3: Diode Array
254 2.0000Da
Range: 1.637
2.71
268.820
63482
2.22
211.820
59
Time Height Area Area%
2.22 1369 58.99 0.09
2.71 1630048 63481.61 99.89
3.88 393 13.26 0.02
AU
%
Time

Compound **1y**

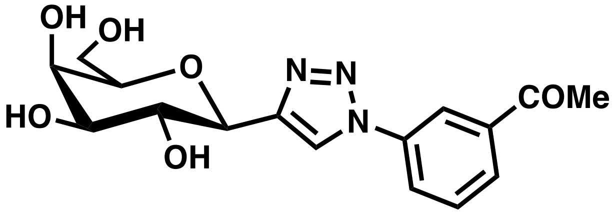

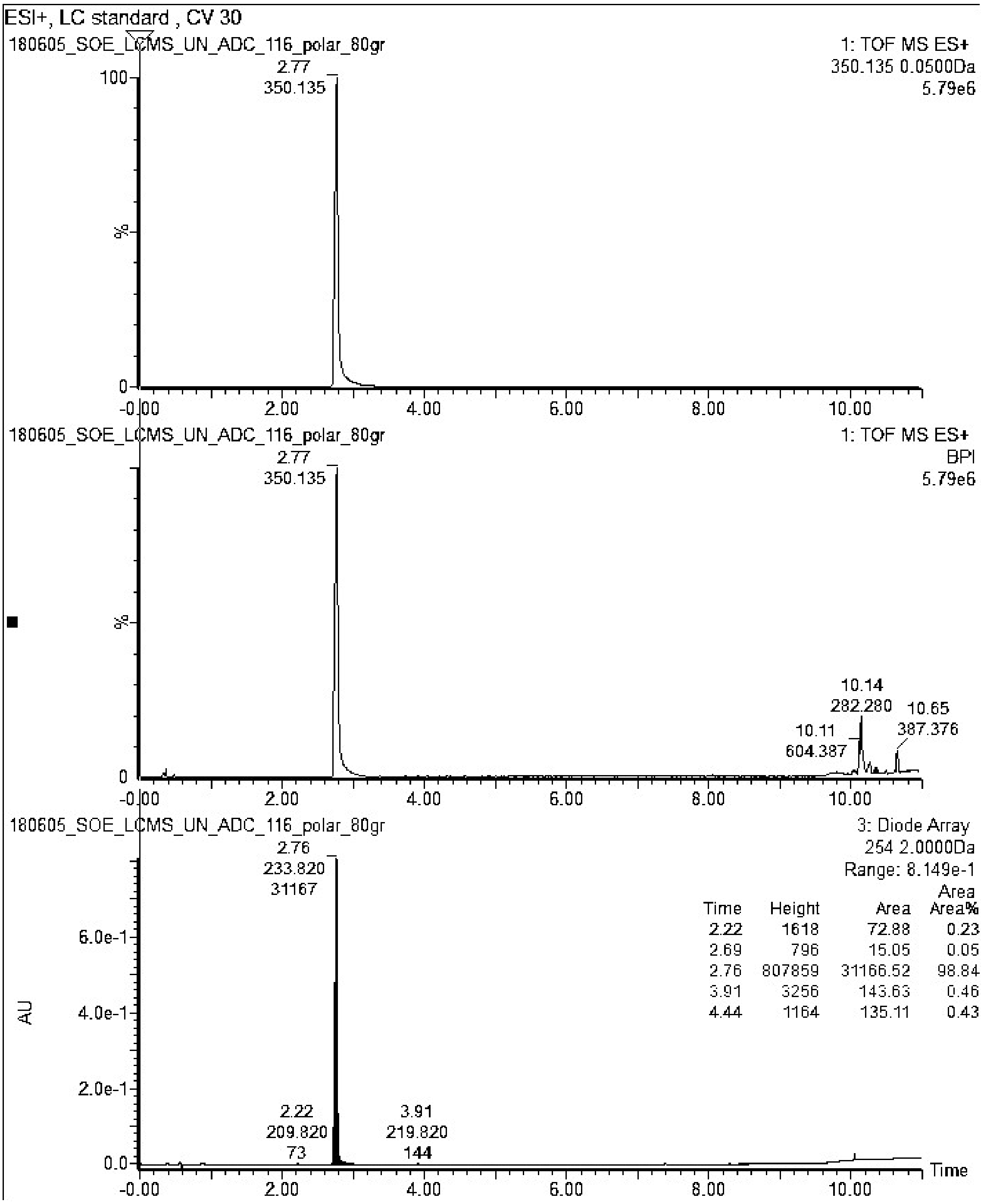

Time	Height	Area	Area Area%
2.22	1618	72.88	0.23
2.69	796	15.05	0.05
2.76	807859	31166.52	98.84
3.91	3256	143.63	0.46
4.44	1164	135.11	0.43

Compound **1z**

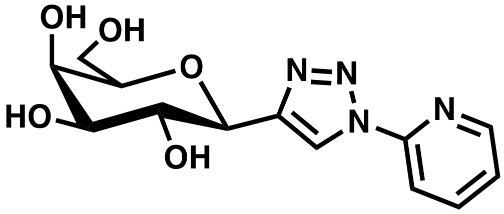

OH
OH
O
N=N
N
N
HO
OH

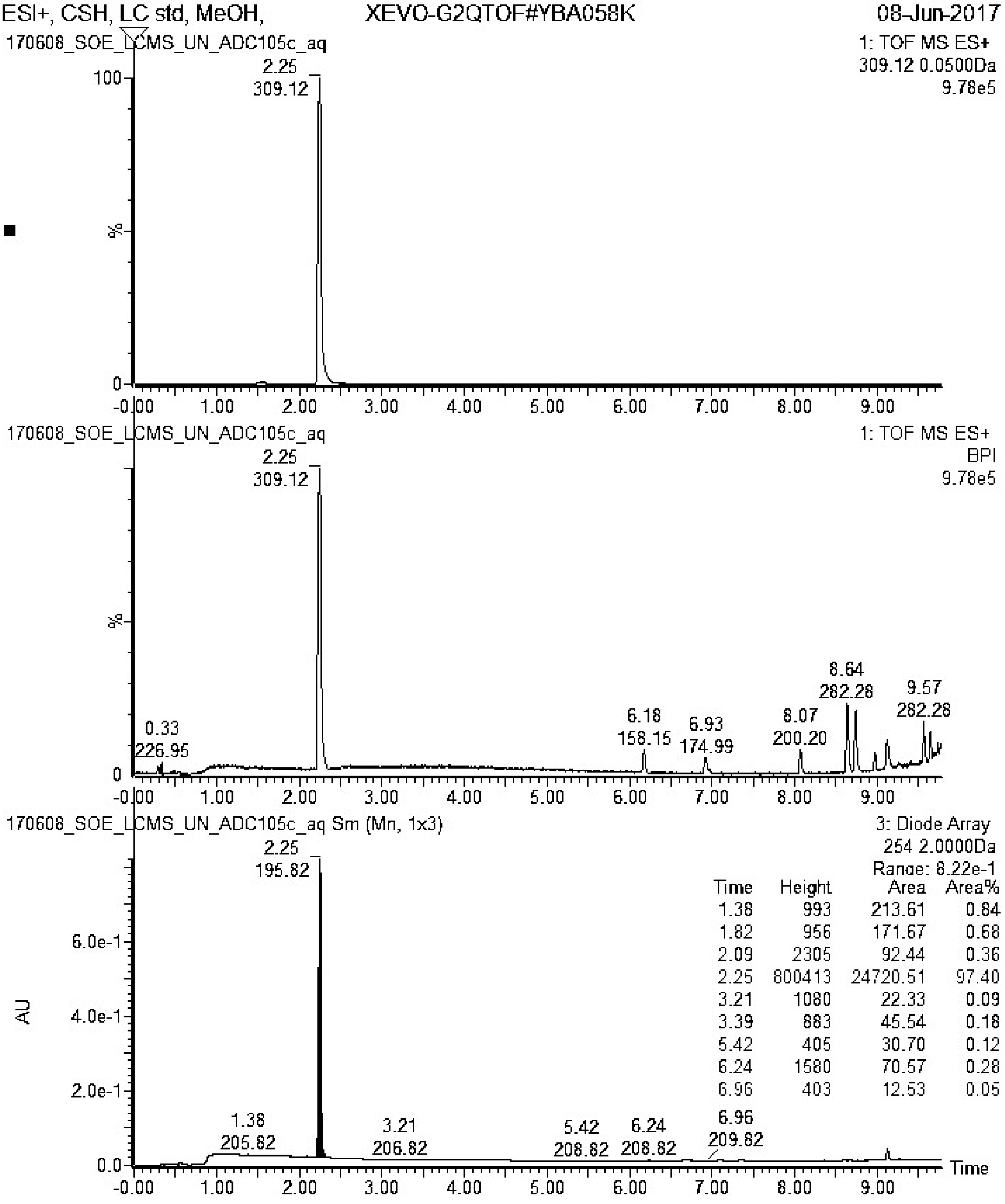

ESI+, CSH, LC std, MeOH,
XEVO-G2QTOF#YBA058K
08-Jun-2017
170608_SOE_LCMS_UN_ADC105c_aq
1: TOF MS ES+
309.12 0.0500Da
9.78e5
2.25
309.12
170608_SOE_LCMS_UN_ADC105c_aq
1: TOF MS ES+
BPI
9.78e5
0.33
226.95
6.18
158.15
6.93
174.99
8.07
200.20
8.64
282.28
9.57
282.28
170608_SOE_LCMS_UN_ADC105c_aq Sm (Mn, 1x3)
3: Diode Array
254 2.0000Da
Range: 8.22e-1
2.25
195.82
1.38
205.82
3.21
206.82
5.42
208.82
6.24
208.82
6.96
209.82
AU
%
Time
Time Height Area Area%
1.38 993 213.61 0.84
1.82 956 171.67 0.68
2.09 2305 92.44 0.36
2.25 800413 24720.51 97.40
3.21 1080 22.33 0.09
3.39 883 45.54 0.18
5.42 405 30.70 0.12
6.24 1580 70.57 0.28
6.96 403 12.53 0.05

Compound **16a**

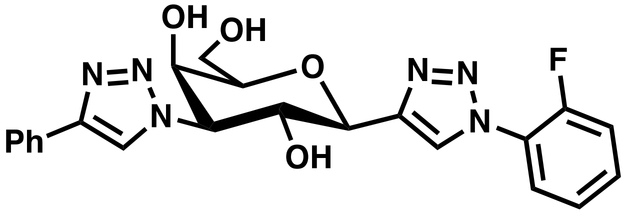

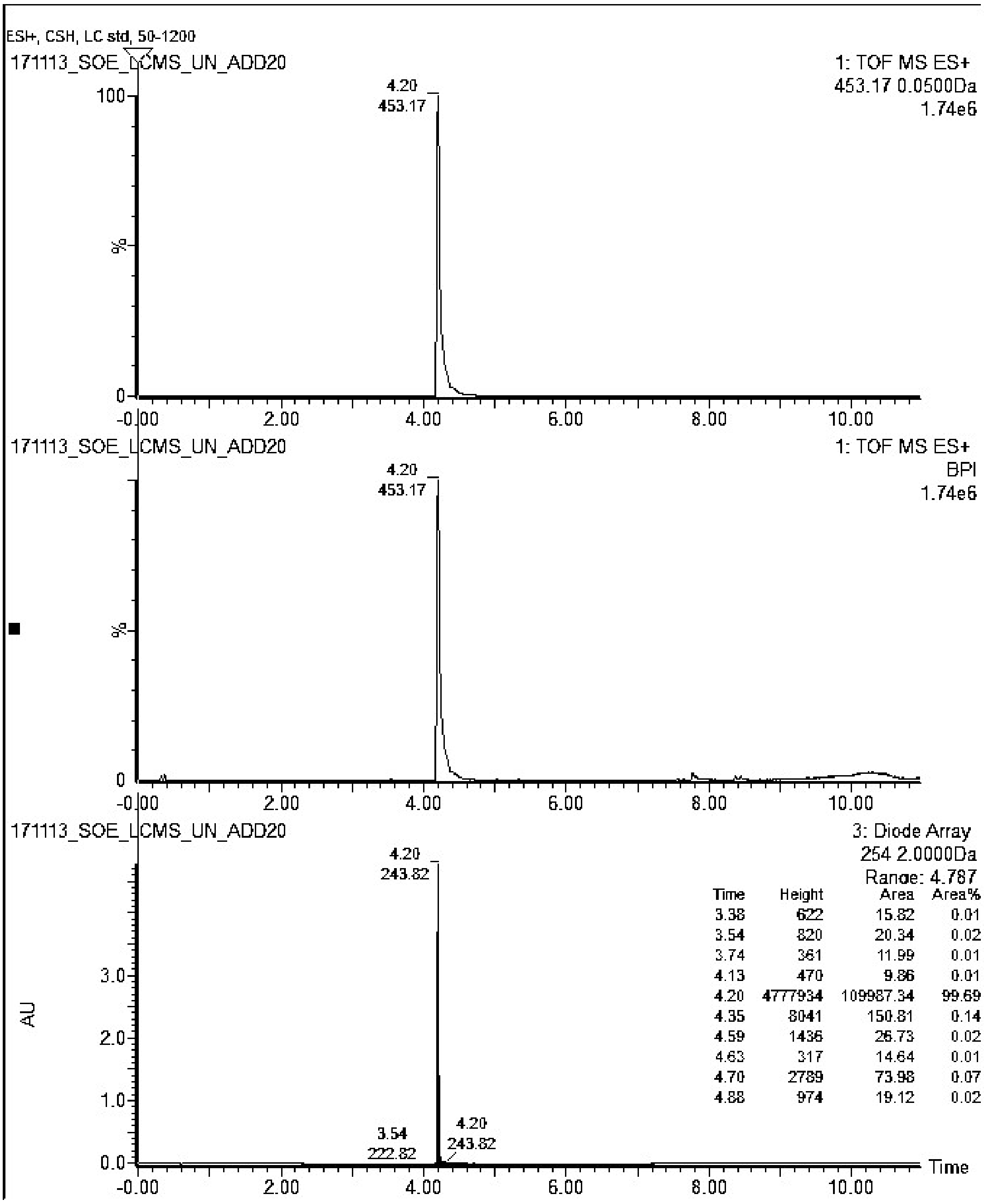

Compound **16b**

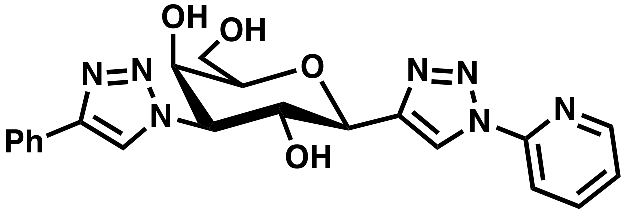

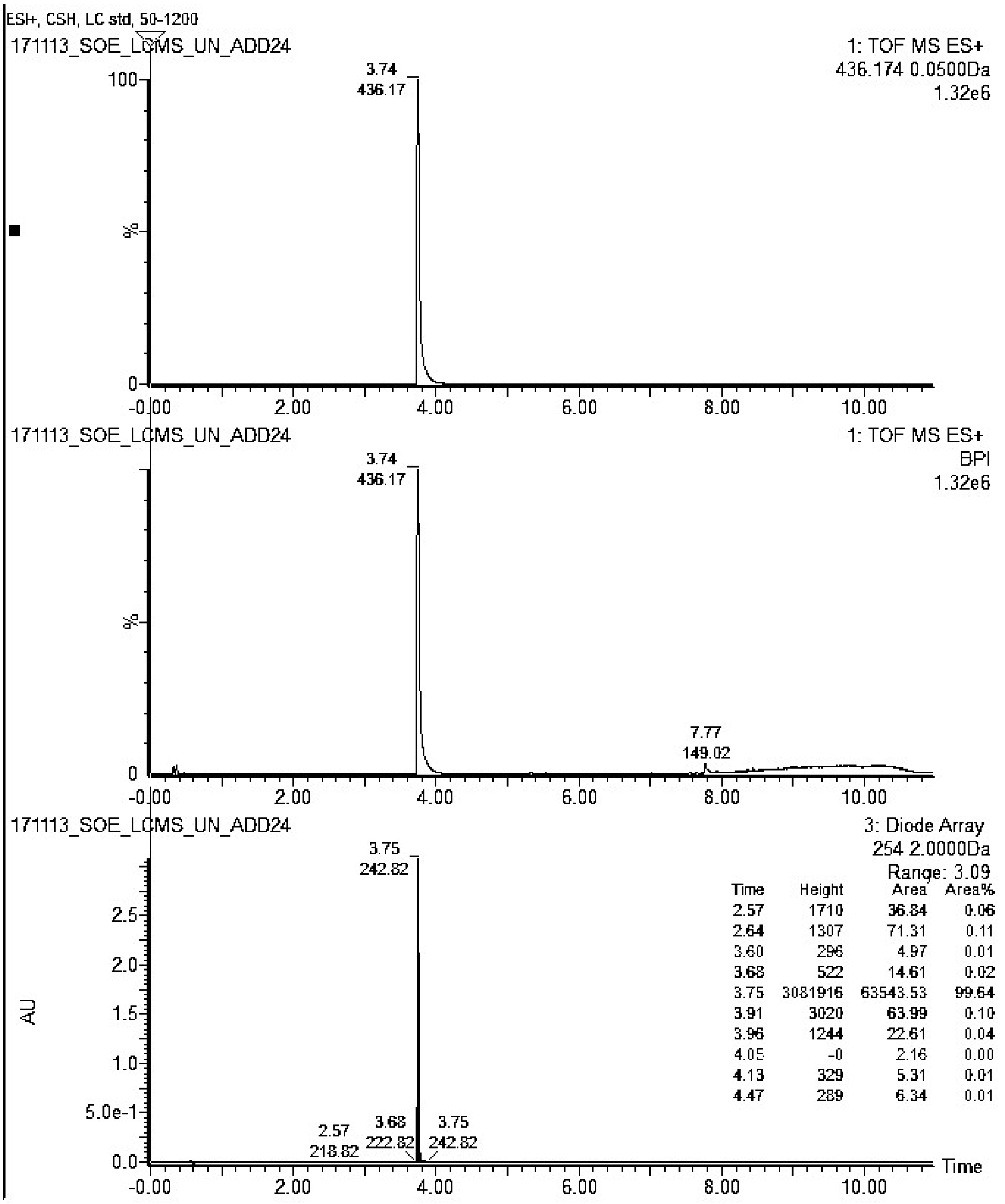

Compound **18**

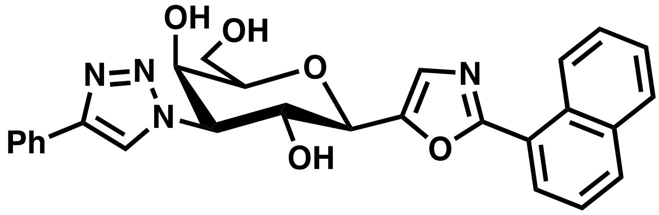
OH
OH
N=N
N
O
N
Ph
OH
O

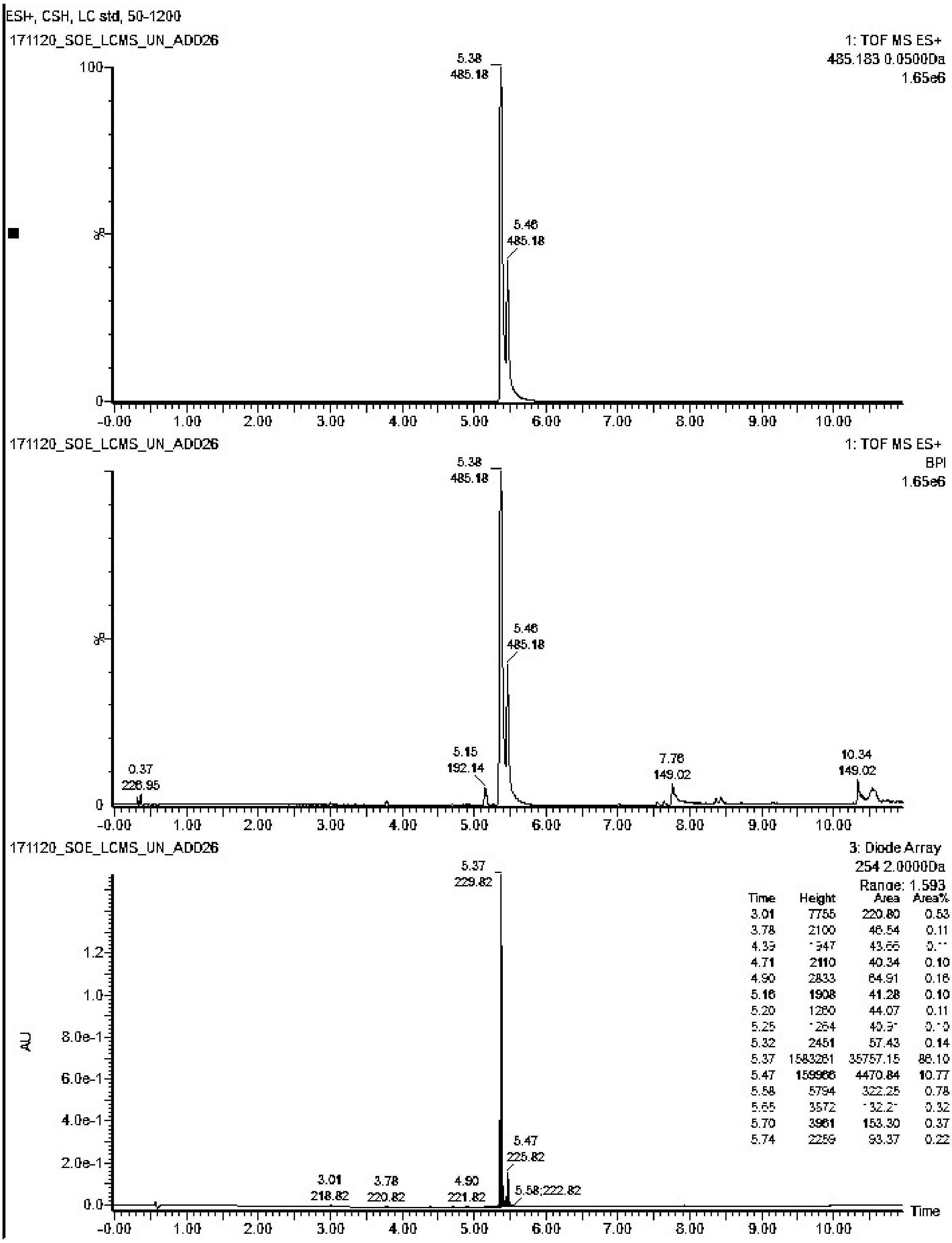
ESI+, CSH, LC std, 50-1200
171120_SOE_LCMS_UN_AD026
1: TOF MS ES+
485.183 0.0500Da
1.65e6
5.38
485.18
5.46
485.18
100
%
0
-0.00 1.00 2.00 3.00 4.00 5.00 6.00 7.00 8.00 9.00 10.00
171120_SOE_LCMS_UN_AD026
1: TOF MS ES+
BPI
1.65e6
5.38
485.18
5.46
485.18
0.37
228.95
5.15
192.14
7.78
149.02
10.34
149.02
%
0
-0.00 1.00 2.00 3.00 4.00 5.00 6.00 7.00 8.00 9.00 10.00
171120_SOE_LCMS_UN_AD026
3: Diode Array
254 2.0000Da
Range: 1.593
Time Height Area Area%
3.01 7755 220.80 0.53
3.78 2100 46.54 0.11
4.39 43.66
4.71 2110 40.34 0.10
4.90 2833 64.91 0.16
5.16 1908 41.28 0.10
5.20 1260 44.07 0.11
5.25
5.32 2451 57.43 0.14
5.37 1583281 35757.15 88.10
5.47 159966 4470.84 10.77
5.58 5794 322.25 0.78
5.65 3572 0.32
5.70 3961 153.30 0.37
5.74 2259 93.37 0.22
5.37
229.82
5.47
225.82
3.01
218.82
3.78
220.82
4.90
221.82
5.58;222.82
AU
1.2
1.0
8.0e-1
6.0e-1
4.0e-1
2.0e-1
0.0
Time
-0.00 1.00 2.00 3.00 4.00 5.00 6.00 7.00 8.00 9.00 10.00

Compound **24a**

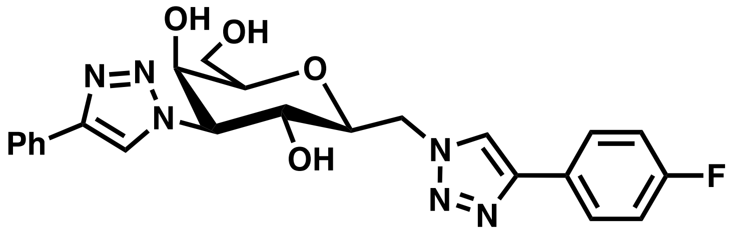

ESI+, LC standard

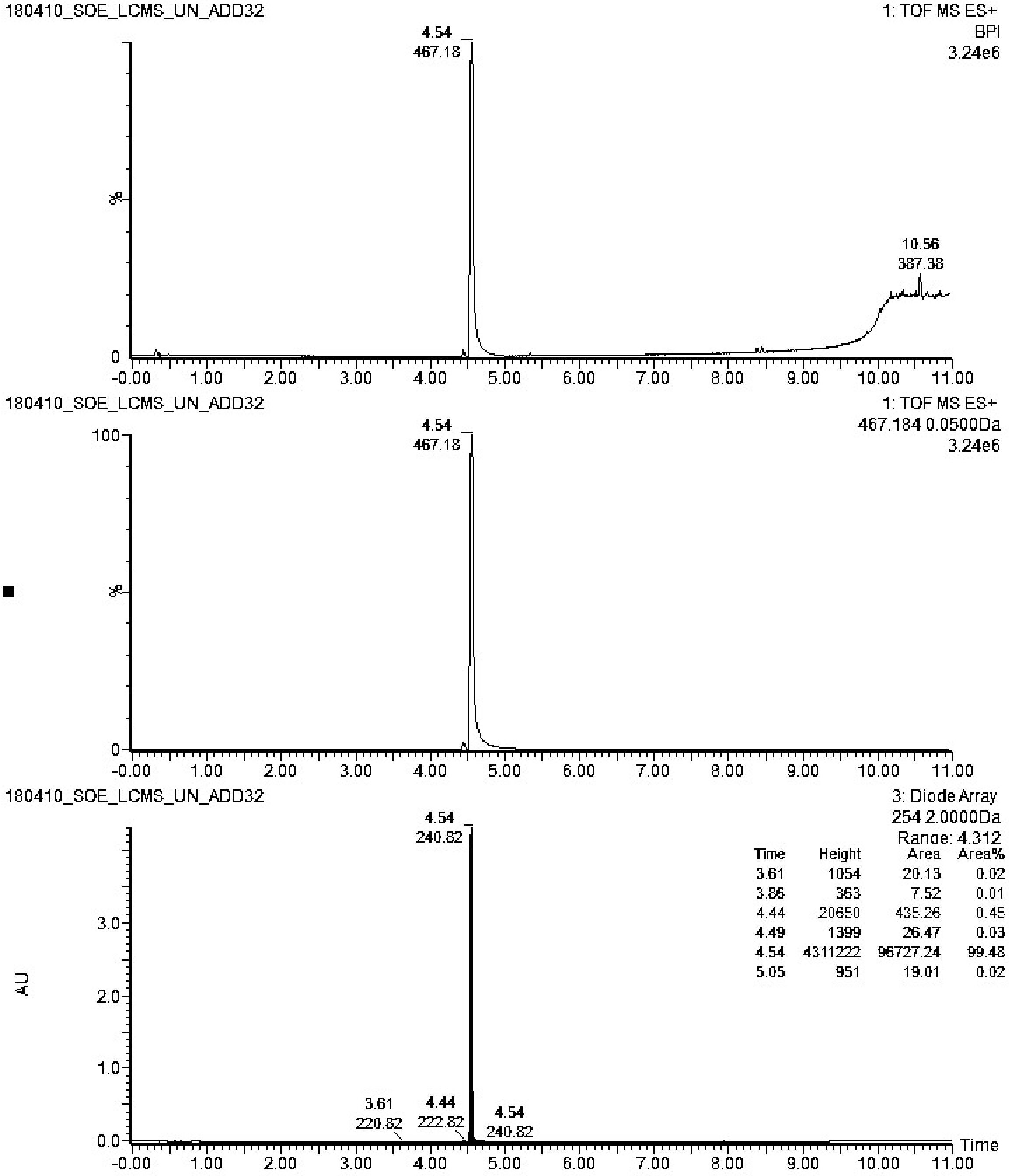

Compound **24b**

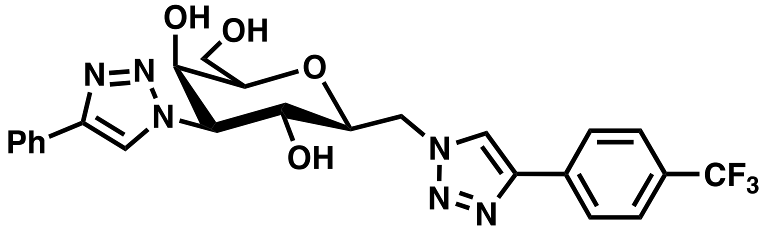

ESI+, LC standard

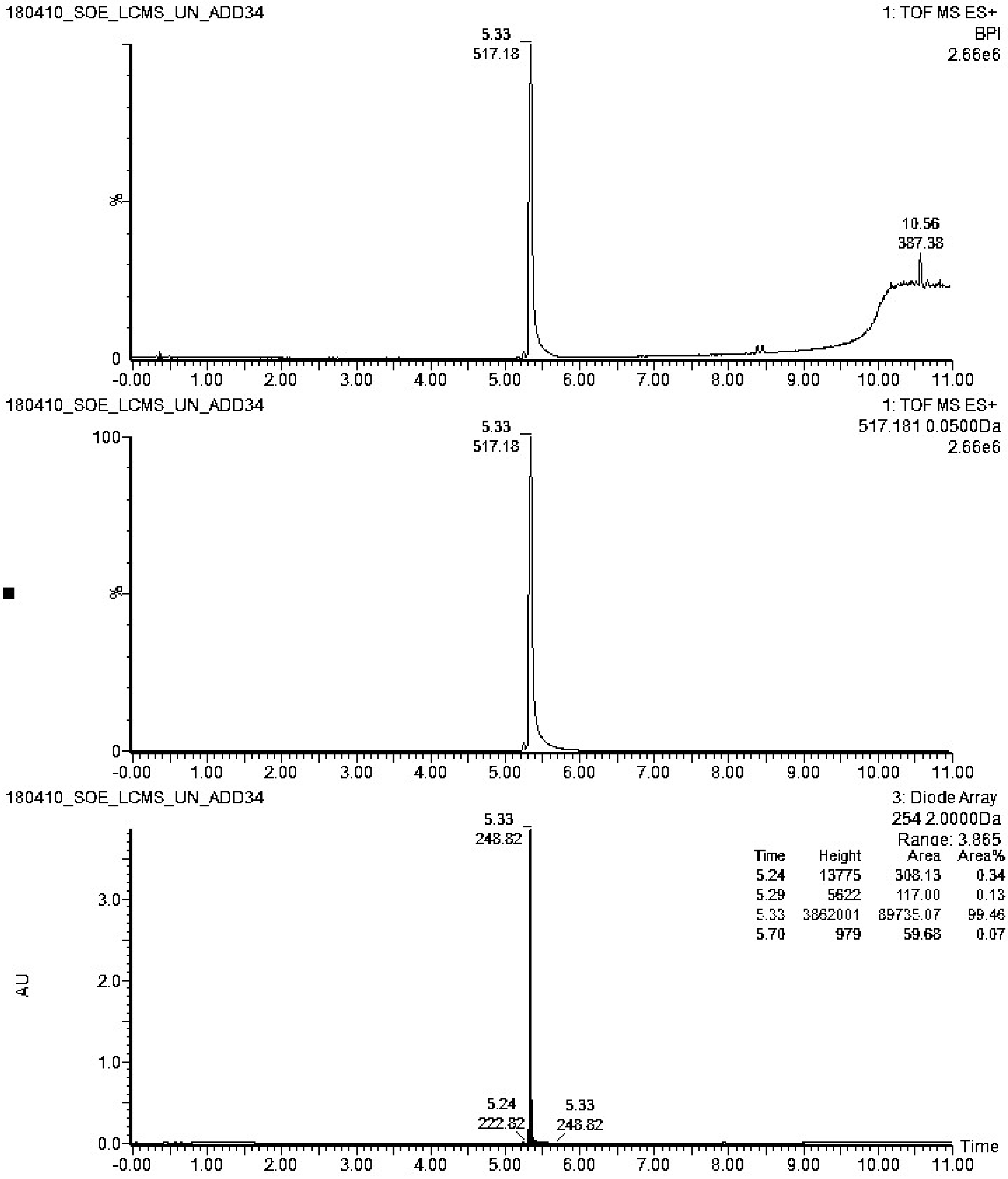

Compound **24c**

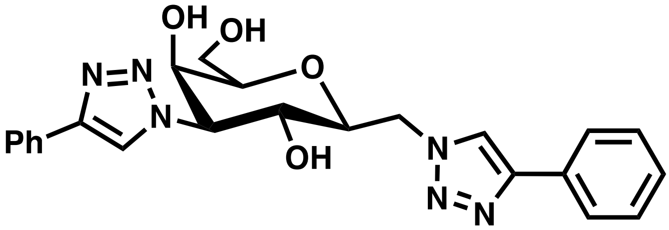

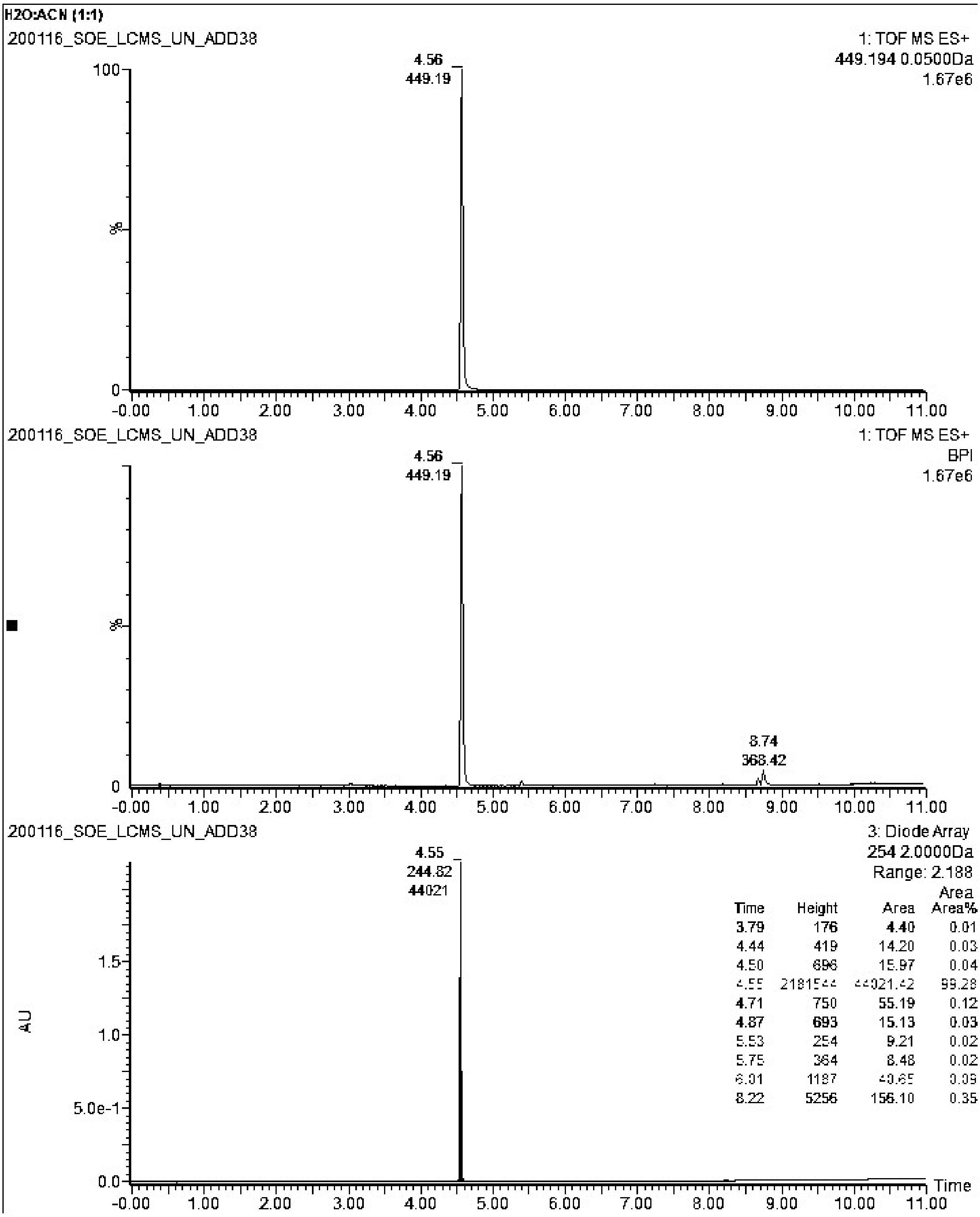

Time	Height	Area	Area Area%
3.79	176	4.40	0.01
4.44	419	14.20	0.03
4.50	696	15.97	0.04
4.55	2181544	44021.42	99.28
4.71	750	55.19	0.12
4.87	693	15.13	0.03
5.53	254	9.21	0.02
5.75	364	8.48	0.02
6.01	1187	40.65	0.09
8.22	5256	156.10	0.35

Compound **24d**

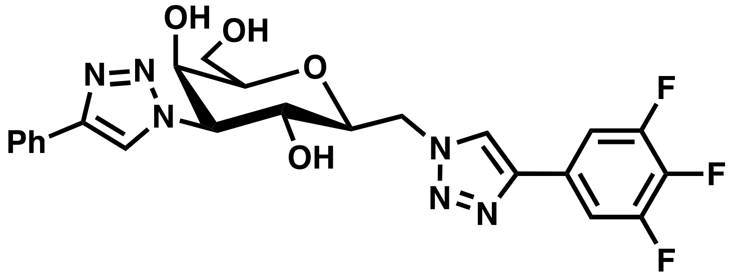

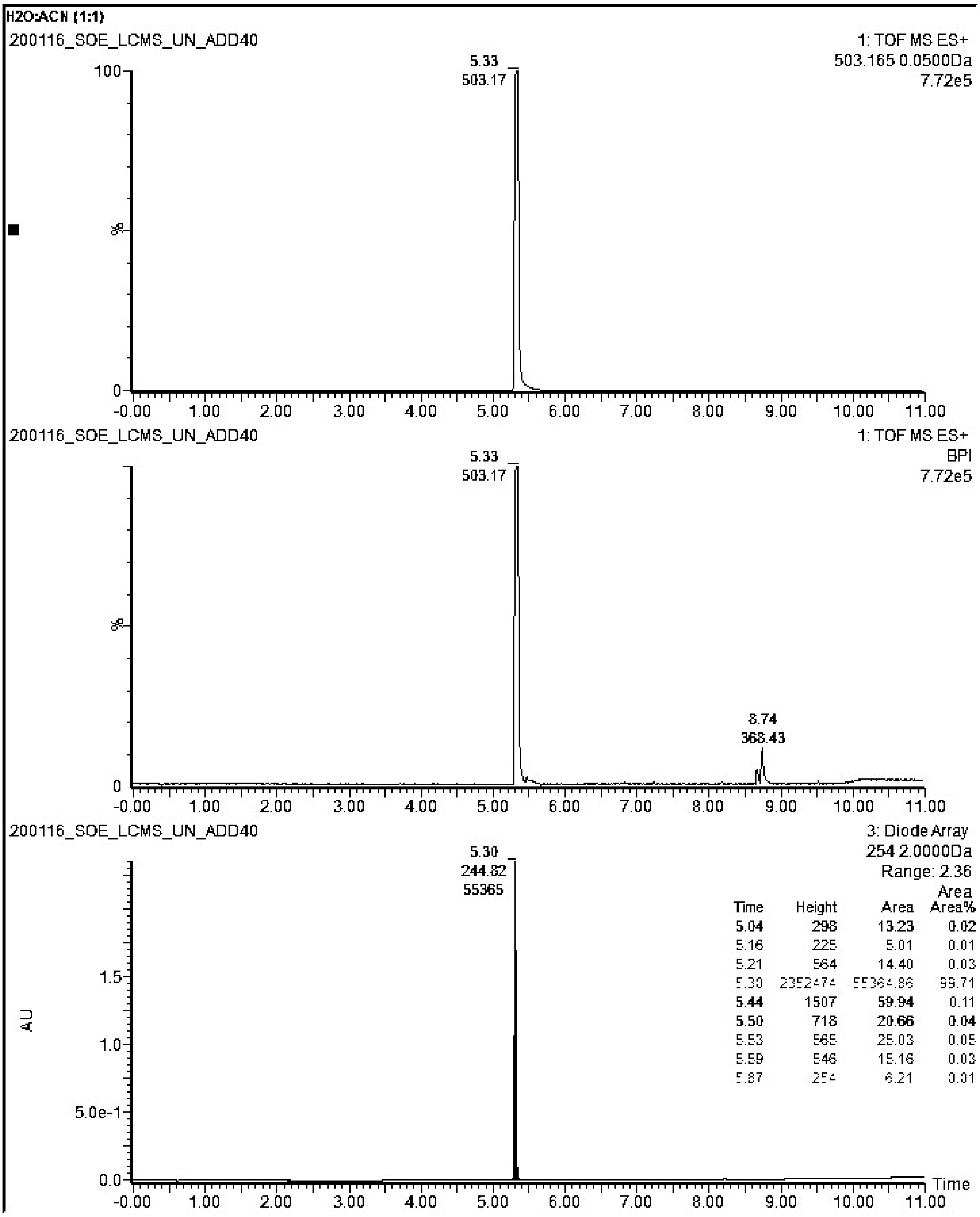

Table S1. Crystallographic data and refinement statistics of galectin-4C in complex with **24a**. All values in parenthesis describe the statistics in the highest outer shell.

	Galectin-4C in complex with **24a**
Crystal system, space group	Monoclinic, $P2_1$
Unit Cell parameters (Å)	a = 45.70, b = 128.56, c = 45.89,<br>α = 90.0°, β = 101.2°, γ = 90.0°
Resolution	45.01 – 2.28 (2.36-2.28)
Total number of Observations	80041 (7503)
Total number of Unique reflections	23306 (2251)
CC (1/2)	0.992 (0.766)
Completeness (%)	98.6 (98.5)
I/σ(I)	6.8 (2.3)
R_{merge} (%)	10.0 (44.0)
**Refinement**	
Resolution (Å)	36.77 – 2.28
Total number of unique observations	23261
R_{cryst} (%)	19.70
R_{free} (%)	26.70
**No. of Atoms**	
Protein	4343
Ligand	68
Water molecules	75
**Average B factors (Å²)**	
Protein	38.9
Ligand	41.7
Water molecules	35.0
**RMS deviations**	
Bond Lengths (Å)	0.010
Bond Angles (°)	1.692
**Ramachandran plot statistics**	
Favoured (%)	96.86
Allowed (%)	2.77
Outliers (%)	0.37
PDB ID	6WAB
	Galectin-4C in complex with **24a**
Crystal system, space group	Monoclinic, $P2_1$
